# Multi-Omics Analysis in β-Thalassemia Using an *HBB* Gene-Knockout Human Erythroid Progenitor Cell Model

**DOI:** 10.3390/ijms23052807

**Published:** 2022-03-04

**Authors:** Guoqiang Zhou, Haokun Zhang, Anning Lin, Zhen Wu, Ting Li, Xumin Zhang, Hongyan Chen, Daru Lu

**Affiliations:** 1 State Key Laboratory of Genetic Engineering, MOE Engineering Research Center of Gene Technology, School of Life Sciences, Fudan University, Shanghai 200438, China; 18110700067@fudan.edu.cn (G.Z.); zhanghaokun666@hotmail.com (H.Z.); 19210700038@fudan.edu.cn (A.L.); 18110700083@fudan.edu.cn (Z.W.); xumin_zhang@fudan.edu.cn (X.Z.); 2 Human Phenome Institute, Fudan University, Shanghai 200438, China; 21112030008@m.fudan.edu.cn; 3 NHC Key Laboratory of Birth Defects and Reproductive Health, Chongqing Key Laboratory of Birth Defects and Reproductive Health, Chongqing Population and Family Planning, Science and Technology Research Institute, Chongqing 404100, China

**Keywords:** omics study, *HBB*, *HBG1/2*, HIF1 pathway, stress erythropoiesis

## Abstract

β-thalassemia is a hematologic disease that may be associated with significant morbidity and mortality. Increased expression of *HBG1/2* can ameliorate the severity of β-thalassemia. Compared to the unaffected population, some β-thalassemia patients display elevated *HBG1/2* expression levels in their red blood cells. However, the magnitude of up-regulation does not reach the threshold of self-healing, and thus, the molecular mechanism underlying *HBG1/2* expression in the context of *HBB*-deficiency requires further elucidation. Here, we performed a multi-omics study examining chromatin accessibility, transcriptome, proteome, and phosphorylation patterns in the *HBB* homozygous knockout of the HUDEP2 cell line (HBB-KO). We found that up-regulation of *HBG1/2* in HBB-KO cells was not induced by the H3K4me3-mediated genetic compensation response. Deletion of *HBB* in human erythroid progenitor cells resulted in increased ROS levels and production of oxidative stress, which led to an increased rate of apoptosis. Furthermore, in response to oxidative stress, slower cell cycle progression and proliferation were observed. In addition, stress erythropoiesis was initiated leading to increased intracellular *HBG1/2* expression. This molecular model was also validated in the single-cell transcriptome of hematopoietic stem cells from β-hemoglobinopathy patients. These findings further the understanding of *HBG1/2* gene regulatory networks and provide novel clinical insights into β-thalassemia phenotypic diversity.

## 1. Introduction

The human body generates approximately two million red blood cells (RBCs) every second through a process called erythropoiesis, which usually occurs in the bone marrow. RBCs circulate in the blood for approximately 120 days to deliver oxygen to tissues via a tetrameric hemoglobin protein [1]. Fetal hemoglobin (HbF, α_2_γ_2_) is comprised of two copies of γ-globin (*HBG1/2*) and two copies of α-globin (*HBA*), while adult hemoglobin (HbA, α_2_β_2_) is comprised of two copies of α-globin (*HBA*) and two copies of β-globin (*HBB*) [2,3]. The *HBG1/2* gene is primarily expressed during development, but silenced soon after birth and replaced by *HBB* expression [2,4].

β-thalassemia is a hereditary hematological disease caused by over 300 mutations in the *HBB* gene [5]. Among β-thalassemia patients, there is a sub-population who simultaneously exhibit the hereditary persistence of fetal hemoglobin (HPFH) [2,6]. HPFH-related mutations in the *HBB* gene cluster or point mutations in the *HBG1/2* gene lead to excessive *HBG1/2* persisting in adult RBCs (more than 20% of total globin). HPFH patients with β-thalassemia mutations do not show symptoms such as anemia, suggesting that reactivation of the silently expressed *HBG1/2* gene in adult RBCs can treat β-thalassemia [2,3,7].

The balance between production and destruction of RBCs contributes to erythroid homeostasis to ensure adequate oxygen delivery to tissues [8]. Usually, erythropoiesis occurs in the bone marrow and produces RBCs at a constant rate. However, when this process is blocked, an alternative pathway, known as stress erythropoiesis, is activated [9]. Stress erythropoiesis can rapidly generate a large number of new RBCs for maintaining tissue oxygenation. Considerable evidence suggests that stress erythropoiesis is similar to fetal erythropoiesis. During stress erythropoiesis, stress erythroid progenitors express HbF upon differentiation, whereas in adult steady-state erythropoiesis, erythroid progenitors primarily express HbA [9,10,11]. In addition, hypoxia-inducible factor HIF1 can promote stress erythropoiesis through coordinated cell type-specific hypoxic responses [12,13,14]. Compared to the unaffected population, some β-thalassemia patients display elevated *HBG1/2* expression in their RBCs [7,15]. *HBG1/2* expression is also up-regulated in the RBCs of *HBB*-deficient *Macaca fascicularis* monkeys [16]. *HBG1/2* has also been shown to be up-regulated after genome editing of *HBB* in CD34^+^ adult-mobilized hematopoietic stem and progenitor cells (HSPCs) [17,18].

Since one of the processes associated with erythrocyte maturation is enucleation, important genetic regulation must take place before this event [19,20]. Here, we generated a human umbilical cord blood-derived erythroid progenitor (HUDEP2) cell line with homozygous knockout of *HBB* to elucidate the molecular mechanisms underlying the up-regulation of *HBG1/2* expression induced by *HBB* deletion. A CRISPR/Cas9 approach was used to detect potential off-target sites in HBB-KO cells, although no off-target effects were found. We constructed plasmids containing a series of nonsense mutations that produced truncated HBB mutant proteins. The plasmids were transfected into HEK-293T cells chemically and K562 cells via electroporation. We found that none of these plasmids could induce *HBG1/2* expression. CUT&Tag and qPCR experiments demonstrated that there were no differences in H3K4me3 enrichment in the *HBG1/2* promoter region. Taken together, our findings demonstrated that up-regulation of *HBG1/2* in HUDEP2 HBB-KO cells was not caused by the H3K4me3-mediated genetic compensation response (GCR).

ATAC-seq [21], RNA-seq [22], TMT-based Proteomics [23], and TMT-based Phosphoproteomics [24] analyses indicated that *HBB* deletion was associated with enhanced erythroid differentiation, the production of oxidative stress, and slower cell cycle progression. We next sought to determine the molecular mechanism underlying the up-regulation of *HBG1/2* expression in response to *HBB* deletion. We showed that the loss of *HBB* in human erythroid progenitor cells led to an increase in ROS production and oxidative stress, and a subsequent increase in the rate of apoptosis. In addition, in response to this oxidative stress, cell cycle progression and proliferation were slowed down, and stress erythropoiesis was initiated, leading to the up-regulation of intracellular *HBG1/2* expression. This molecular model has also been validated in the single-cell transcriptome of hematopoietic stem cells (HSCs) from β-hemoglobinopathy patients. These data provide mechanistic insights into the long-observed but poorly understood phenomenon of up-regulated *HBG1/2* expression in the context of *HBB* deletion.

## 2. Results

### 2.1. The H3K4me3-Mediated GCR Does Not Induce Up-Regulation of HBG1/2 Expression in the Context of HBB-KO

HUDEP2 cells are a commonly used tool for studying globin regulation [4,25,26,27]. We constructed a cellular model of *HBB* homozygous knockout in HUDEP2 cells, hereafter referred to as HBB-KO. RT-qPCR analysis revealed that *HBG1/2* transcription was increased ~150 times, while flow cytometry indicated that the HbF content was increased ~264 times in HBB-KO cells compared to HUDEP2 WT cells (Figure S1). In order to determine whether the elevated *HBG1/2* expression observed in HBB-KO cells was due to the CRISPR off-target effect, we searched for potential off-target sites from the guide RNAs (gRNAs) used in the construction of HUDEP2 HBB-KO using the CCTop website [28]. The top six sites were PCR-amplified, then Sanger sequenced to examine the off-target effects. No off-target cleavage was observed (Figure S2, Table S1).

Next, we examined the cells for GCR, a phenomenon in which an organism with a gene mutation does not develop the expected abnormal gene expression due to compensatory actions of another gene, which functionally compensates for the loss-of-function gene by restoring more normal physiological function [29]. GCR can be induced by introducing an exogenously constructed truncated nonsense mutation plasmid into the cells [30]. Using a series of *HBB*-truncated nonsense mutants (Figure S3A), we demonstrated by RT-qPCR analysis that none of the *HBB*-truncated nonsense mutant plasmids induced up-regulation of *HBG1/2* expression relative to the control (HBB-WT) in HEK-293T (Figure S3B) and K562 cells (Figure S3C). The widely recognized molecular mechanism of GCR involves the binding of shortened RNA (produced by gene mutations) to proteins such as UPF3A and components of the COMPASS complex, which then bind to the promoter region of another gene homologous to the mutant gene. The combination causes H3K4me3 enrichment of the homologous gene promoter, thereby promoting upregulation of the related gene. CUT&Tag (anti-H3K4me3-antibody) in WT and HBB-KO cells revealed no significant differences in H3K4me3-enriched signals in the *HBG1/2* gene promoter region (Figure S3D–F). Specifically, H3K4me3 signals were not detected in the *HBG1* promoter region, and while H3K4me3 signals were observed in the *HBG2* promoter region, no significant differences were found in the binding region (Figure S3G). qPCR verified that there were no significant differences in H3K4me3 enrichment in the *HBG2* promoter (Figure S3H). Thus, our findings suggest that H3K4me3-mediated GCR does not induce up-regulation of *HBG1/2* expression in the context of HBB-KO.

### 2.2. Chromatin Accessibility Analysis Suggests That Erythroid Differentiation Is Enhanced in HBB-KO Cells

Three biological replicates (rep 1–3) were prepared for each sample (WT and HBB-KO) to analyze chromatin accessibility by ATAC-seq. Spearman correlation analysis found that the ATAC-seq data had high reproducibility (Figure S4A). TSS-heat map analysis revealed that there were no significant differences between the WT and the HBB-KO groups (Figure S4C), indicating that *HBB* knockout did not drastically change chromatin accessibility. However, subtle differences in chromatin accessibility were found by comparing the overall reads in the WT and HBB-KO groups (Figure S4B). ATAC-seq track analysis revealed that the *HBB* signal was decreased, and the *HBG1/2* signal was increased in the HBB-KO cells compared to the WT cells (Figure S5), consistent with the RT-qPCR data (Figure S1). No differences in the *BCL11A* and *ZBTB7A* signals in the HBB-KO cells relative to the WT cells were observed by ATAC-seq track analysis (Figure S6A,B). Our RT-qPCR data also showed that *BCL11A* and *ZBTB7A* expression levels were not significantly different between the WT and HBB-KO cells (Figure S6C). Therefore, elevated *HBG1/2* expression was not regulated by BCL11A and ZBTB7A in the context of HBB-KO.

**Figure 1:**
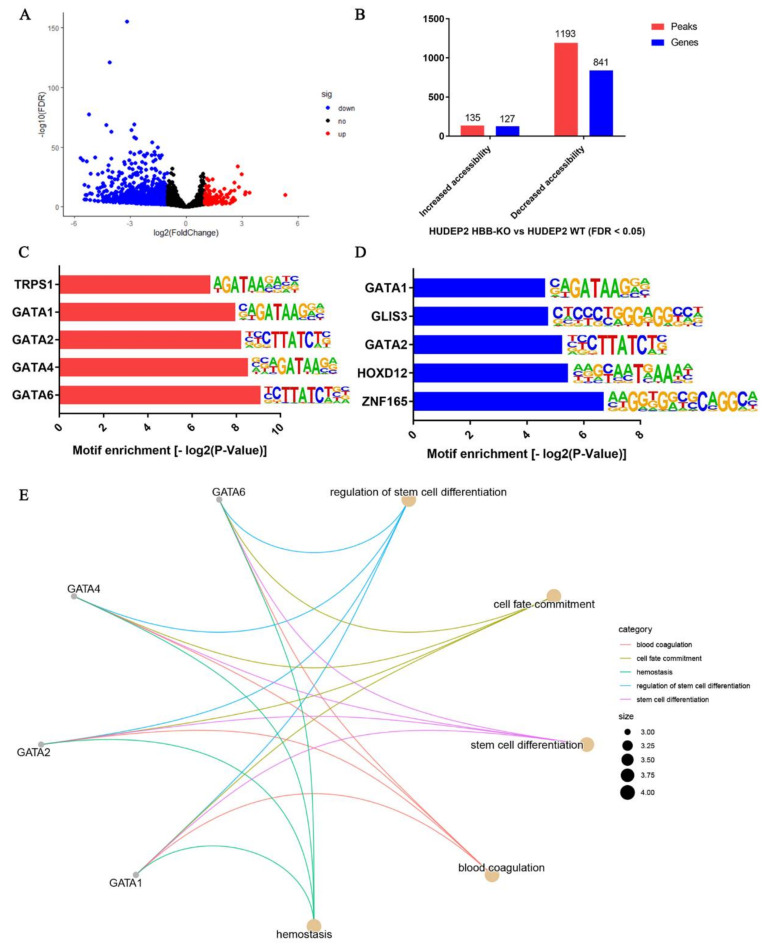
ATAC-seq analysis of HUDEP2 HBB-KO and HUDEP2 cells. (**A**) Volcano plots analysis of ATAC-seq peaks. (**B**) Chromatin accessibility analysis of ATAC-seq. (**C**) TFs corresponding to increase in accessibility sites. (**D**) TFs corresponding to decrease in accessibility sites. (**E**) Biological processes analysis of TFs corresponding to differential accessibility sites.

To characterize the subtle differences in chromatin accessibility, we performed differential chromatin accessibility analysis. The volcano plot (FDR ≤ 0.05, Fold change ≥ 2 or Fold change ≤ 0.5) showed that the increased accessibility peaks were 135, corresponding to 127 genes, while the decreased accessibility peaks were 1193, corresponding to 841 genes in the HBB-KO cells relative to the WT cells (Figure 1A,B, Tables S2 and S3). HOMER enrichment analysis revealed that the transcription factors corresponding to differentially accessibility sites were TRS1, GATA1, GATA2, GATA4, GATA6, GLIS3, HOXD12, and ZNF165 (Figure 1C,D). These transcription factors were mainly enriched in the biological processes of stem cell differentiation and cell fate determination (Figure 1E). Thus, the loss of *HBB* gene function may directly affect the differentiation process of erythroid progenitor cells.

### 2.3. Transcriptome Analysis Indicates That Oxidative Stress May Have Occurred in HBB-KO Cells

**Figure 2:**
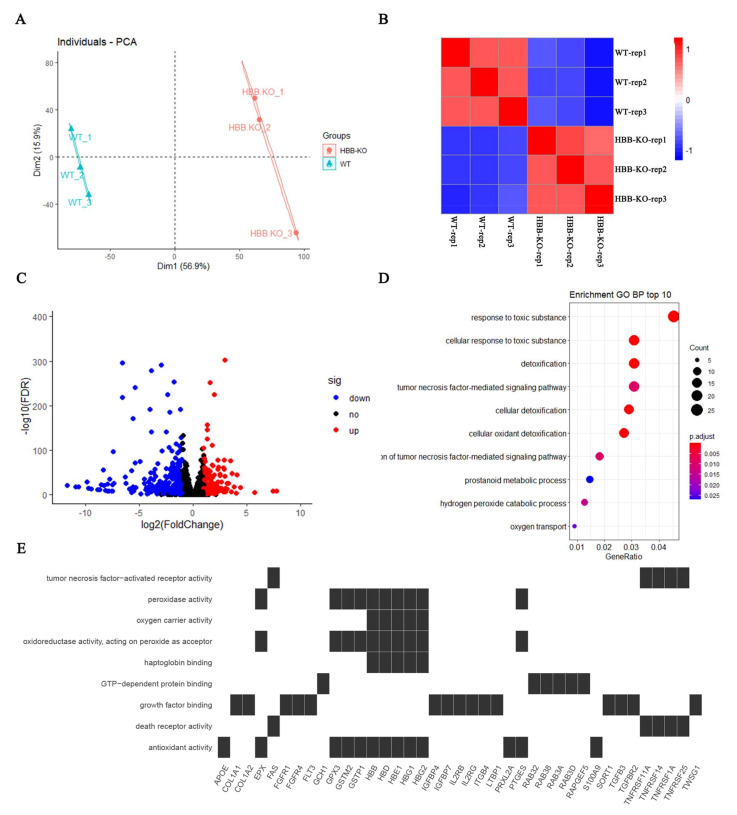
RNA-seq analysis of HUDEP2 HBB-KO and HUDEP2 cells. (**A**) The principal component analysis (PCA) of HUDEP2 HBB-KO (n = 3) and HUDEP2 cells (n = 3). (**B**) Spearman correlation analysis in HUDEP2 cells (n = 3) and HUDEP2 HBB-KO cells (n = 3). (**C**) Volcano plots analysis of RNA-seq. (**D**) Biological processes analysis of DEGs. (**E**) Molecular functions analysis of DEGs.

In order to determine the molecular networks in the context of HBB-KO, we performed RNA-seq in WT and HBB-KO cells. PCA and Spearman correlation analysis showed that the biological replicates of WT/HBB-KO had good reproducibility (Figure 2A,B). The volcano plot (FDR ≤ 0.05, Fold change ≥ 2 or Fold change ≤ 0.5) showed that there were 200 up-regulated and 470 down-regulated genes in the HBB-KO group compared to the WT group (Figure 2C, Table S4). The heatmap revealed that *HBG1/2* was significantly up-regulated and *HBB* was significantly down-regulated in HBB-KO cells, which verified the biological accuracy of our RNA-seq data set (Figure S7). Gene Ontology analysis found that the molecular functions of DEGs were mostly concentrated in anti-oxidation, oxygen transport and cell death (Figure 2E), while biological processes were mostly concentrated in oxidation and detoxification in the cell, and hydrogen peroxide metabolism (Figure 2D). These results suggest that oxidative stress may have occurred in the HBB-KO cells.

### 2.4. Proteome Analysis Reveals That the HIF-1 Pathway Is Activated in HBB-KO Cells

**Figure 3:**
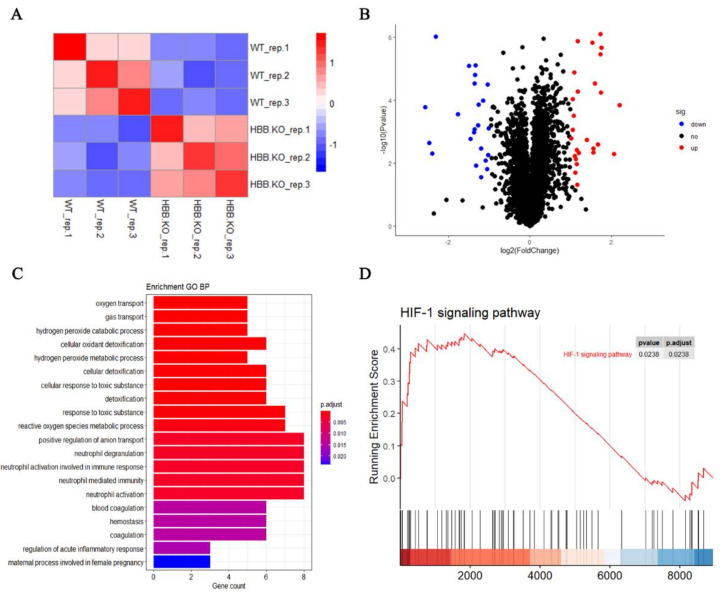
Proteome analysis of HUDEP2 HBB-KO and HUDEP2 cells. (**A**) Spearman correlation analysis in HUDEP2 cells (n = 3) and HUDEP2 HBB-KO cells (n = 3). (**B**) Volcano plots analysis of Proteome. (**C**) Biological processes analysis of DEPs. (**D**) GSEA analysis of HIF1-pathway.

Proteomic analysis provides information about the role of proteins in biological events. Here, we identified 112,881 peptides corresponding to 9042 proteins. Using the TMT-labeled quantitative approach, we obtained quantitative information for 8952 of these 9042 proteins in WT and HBB-KO cells (Table S5). Spearman correlation analysis showed that the biological replicates of WT/HBB had good reproducibility (Figure 3A). The volcano plot showed that there were 27 up-regulated and 23 down-regulated proteins in the HBB-KO group compared to the WT group (Figure 3B). Heatmap analysis revealed that *HBG1/2* was significantly up-regulated and *HBB* was significantly down-regulated in HBB-KO cells (Figure S8), which verified the biological accuracy of our RNA-seq data (Figure S7) and indicated that the proteome data was consistent with the transcriptome data in HBB-KO cells. GO analysis found that the DEPs were mostly enriched in biological processes such as hydrogen peroxide metabolism, intracellular anti-oxidation, and cell homeostasis (Figure 3C). These findings are similar to the results obtained by transcriptome data analysis (Figure 2D). In addition, using GSEA analysis we found that the HIF1 signaling pathway was activated in HBB-KO cells (Figure 3D).

### 2.5. Phosphoproteome Analysis Indicates That Cell Cycle Progression May Be Slower in HBB-KO Cells

**Figure 4:**
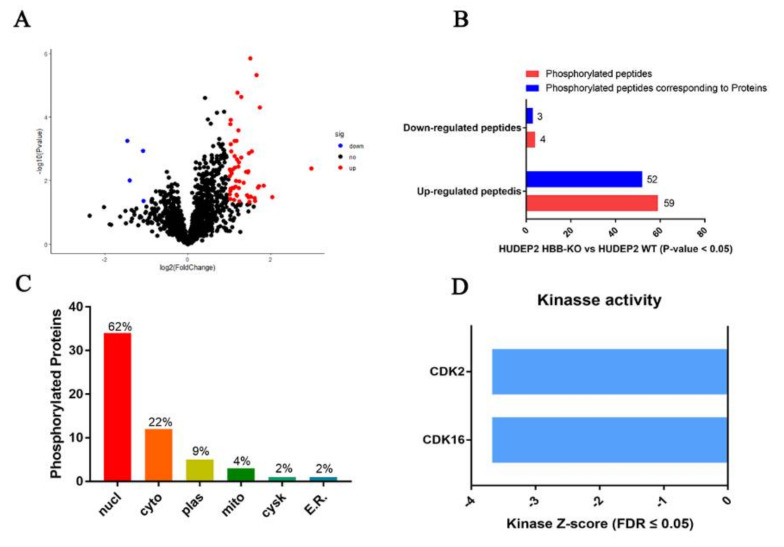
Phosphorylated pattern analysis of HUDEP2 HBB-KO and HUDEP2 cells. (**A**) Volcano plots analysis of Phosphorylated pattern. (**B**) Phosphorylated peptides analysis. (**C**) Subcellular localization analysis of phosphorylated peptides. (**D**) Kinase activity analysis.

Phosphorylated proteomics analysis is used to identify differentially expressed phosphorylated peptides. We found that 59 phosphorylated peptides corresponding to 52 non-redundant proteins were up-regulated, while four phosphorylated peptides corresponding to four non-redundant proteins were down-regulated in HBB-KO cells compared to WT cells (Figure 4A,B, Table S6). In order to understand the biological function of the phosphorylated peptides’ corresponding proteins, we conducted subcellular localization analysis via the WolF PSORT website [31]. We found that 62% of proteins were located in the nucleus (nucl), 22% in the cytoplasmic matrix (cyto), 9% in the cell membrane (plas), 4% in the mitochondria (mito), 2% on the cytoplasmic skeleton (cysk), and 2% on the ribosome (E.R.) (Figure 4C). We analyzed the kinase activity based on the differentially expressed phosphorylated peptides through the KSEP website [32], and found that the cycle-dependent kinases CDK2 and CDK16 (FDR ≤ 0.05) were decreased in the HBB-KO group (Figure 4D). Thus, cell cycle progression may be slower in HBB-KO cells.

### 2.6. Stress Erythropoiesis Increases HBG1/2 Expression in Response to Loss of HBB

**Figure 5:**
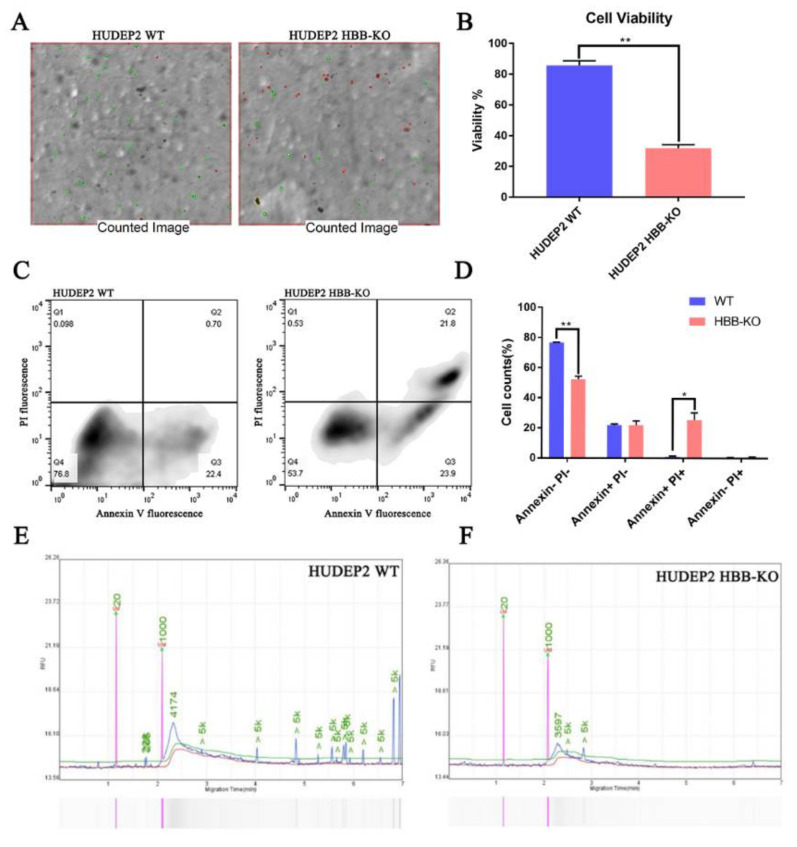
Cell viability and apoptosis analysis of HUDEP2 HBB-KO and HUDEP2 cells. (**A**) Cell viability analysis. (**B**) Statistics analysis of cell viability. (**C**) Cell apoptosis analysis. (**D**) Statistics analysis of Cell apoptosis. (**E**) Capillary electrophoresis analysis of HUDEP2 WT cells. (**F**) Capillary electrophoresis analysis of HUDEP2 HBB-KO cells. * *p* < 0.01, ** *p* < 0.001.

**Figure 6:**
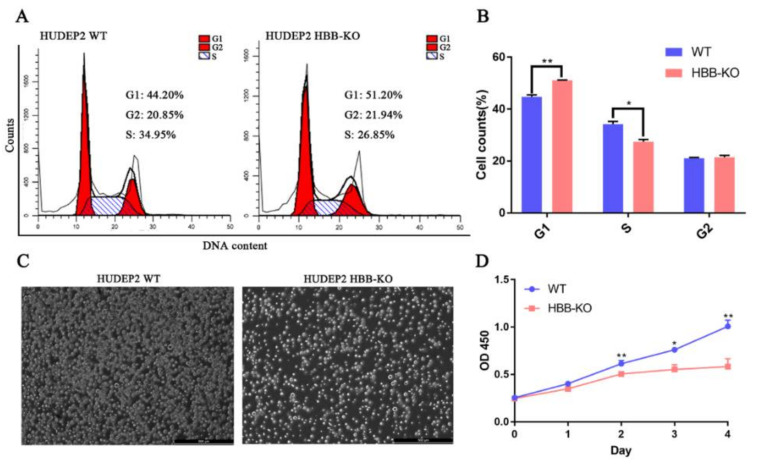
Cell cycle and Cell proliferation analysis of HUDEP2 HBB-KO and HUDEP2 cells. (**A**) Cell cycle analysis. (**B**) Statistics analysis of cell cycle. (**C**) Pellets of HUDEP2 HBB-KO and HUDEP2 WT cells after 48 h of culture. (**D**) CCK-8 assays in HUDEP2 HBB-KO and HUDEP2 WT cells. Values are the means ± SD. * *p* < 0.01, ** *p* < 0.001.

**Figure 7:**
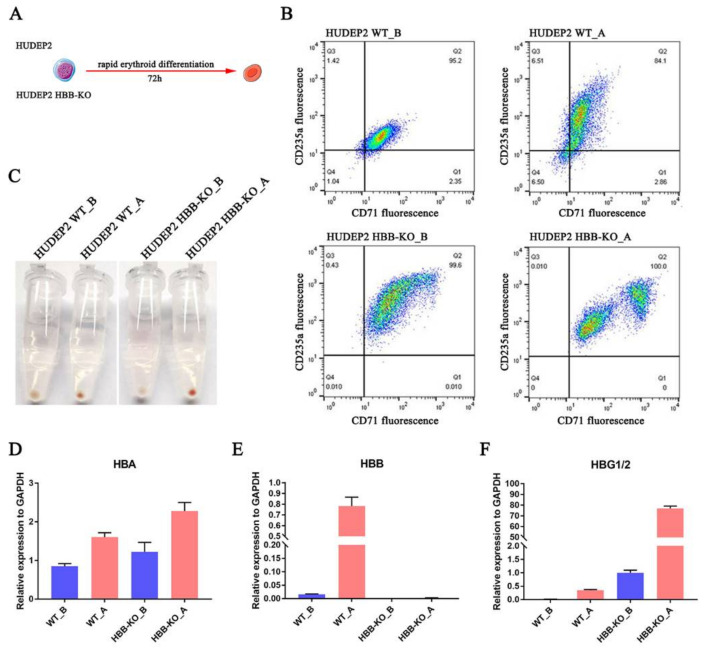
Erythroid differentiation analysis of HUDEP2 WT and HUDEP2 HBB-KO cells. (**A**) Schematic diagram of experimental design. (**B**) Representative flow cytometry dot plots in HUDEP2 HBB KO and HUDEP2 cells stained for CD71 and CD235a. The data are presented as mean of three biological replicates. (**C**) Pellets of HUDEP2 HBB-KO and HUDEP2 WT cells after erythroid differentiation. (**D**) RT-qPCR assays to determine *HBA* expression. (**E**) RT-qPCR assays to determine *HBB* expression. (**F**) RT-qPCR assays to determine *HBG1/2* expression.

Based on our multi-omics study, we performed a series of experiments to confirm the molecular model that *HBB* deletion induces up-regulation of *HBG1/2* expression. We found that ROS content was up-regulated approximately 1.5 times in HBB-KO cells compared with WT cells (Figure S9A), and that the cell viability of HBB-KO cells was approximately 2.7 times lower than WT cells (Figure 5A,B) through DCFH probe and trypan blue staining, respectively. Flow cytometry and capillary electrophoresis assays revealed that the apoptotic rate was increased in HBB-KO cells compared to WT cells (Figure 5C–F). Flow cytometry and CCK8 assays indicated that cell cycle progression and cell proliferation were slower in HBB-KO cells (Figure 6A–D). *HIF1α* expression was shown to be up-regulated in HBB-KO cells compared to WT cells (Figure S9B) by RT-qPCR. In addition, we conducted an erythroid differentiation experiment on WT and HBB-KO cells by collecting samples before (WT_B; HBB-KO_B) and after (WT_A; HBB-KO_A) erythroid differentiation (Figure 7A,C). We found that the fluorescence intensity of CD71 and CD235a was higher in the HBB-KO_B group than in the WT_B group, indicating that the number of erythroid cells in the HBB-KO group was higher than in the WT group. Cells in the HBB-KO_A group were clearly divided into two groups, indicating that some cells had undergone erythroid differentiation (Figure 7B). RT-qPCR analysis showed that after erythroid differentiation, γ-globin expression in the HBB-KO_A group was up-regulated by approximately 160 times compared with the WT_A group (Figure 7D–F). Other erythroid differentiation marker genes including *KLF1, KLF3, FOG1, AHSP, NFE2, EPOR* and *GFI1B* were also up-regulated (Figure S10), indicating that knocking out *HBB* in erythroid progenitor cells induced stress erythropoiesis. Thus, our findings indicated that deletion of *HBB* in human erythroid progenitor cells led to increased ROS production and oxidative stress, which resulted in an increase in the rate of apoptosis. In response to oxidative stress, cell cycle progression and cell proliferation were also slowed down, and stress erythropoiesis was initiated resulting in elevated intracellular *HBG1/2* expression levels. Furthermore, we analyzed the previously reported scRNA-seq data of CD34+-positive HSCs in normal people, β-thalassemia patients, and sickle anemia patients [33] (GEO database: GSE133181). We found that the proportion of erythroid cells in CD34+-positive HSCs in β-thalassemia and sickle anemia patients (8% and 10%) was slightly higher than in normal CD34+-positive HSCs (7.5%) (Figure S11). These findings suggested that HSCs in patients with β-thalassemia and sickle anemia may have partially undergone erythroid differentiation, which is consistent with our model of molecular regulation.

## 3. Discussion

Fetal globin expression during cell-intrinsic stress is a long-observed but poorly understood phenomenon. Here, we constructed an HUDEP2 cell line with a homozygous knockout of the *HBB* gene [34]. Four levels of high-throughput analyses were performed including chromatin accessibility, transcriptome, whole proteome, and phosphorylated proteome analyses. Through the analysis of multi-omics data and preliminary verification by subsequent experiments, we propose that loss of *HBB* gene function in human erythroid progenitor cells leads to increased ROS production and oxidative stress, which causes an increase in the apoptosis rate. At the same time, in response to elevated oxidative stress, cell proliferation slows down, and erythroid differentiation is activated, which ultimately upregulates γ-globin expression (Figure S12). This molecular model has also been partially validated using single-cell transcriptome data of HSCs from patients with β-hemoglobinopathy. Subsequent studies should further verify the role of HIF1-mediated stress erythropoiesis in the context of HBB-KO. Our findings contribute to furthering understanding of the molecular mechanism underlying the up-regulation of *HBG1/2* expression induced by *HBB* deletion, as well as providing some insights into potential treatment strategies for β-thalassemia.

## 4. Materials and Methods

### 4.1. Site-Specific Mutagenesis

A series of nonsense mutant truncated plasmids of the *HBB* gene were constructed using the KOD-Plus-Mutagenesis Kit (TOYOBO, Shanghai, China) according to the manufacturer’s recommendations. Plasmids were transfected into HEK-293T cells using Lipofectamine 3000 (Invitrogen, Waltham, MA, USA) and into K562 cells using the P3 primer cell 96-well NucleofectorTM Kit (LONZA, Basel, Switzerland) according to the manufacturer’s instructions. After 12 h of transfection, the transfection medium was replaced with a complete culture medium. After 48 h of culture, the cells were collected.

### 4.2. RT-qPCR Assays

Cells were homogenized using TRIzol (Thermo Fisher Scientific, Waltham, MA, USA), and RNA was extracted using the RNeasy Mini Kit (Qiagen, Venlo, The Netherlands). Reverse transcription was performed using HiScript III RT SuperMix for qPCR (+gDNA wiper) (Vazyme, Nanjing, China). RT-qPCR reactions were performed using SYBR^®^ Green Realtime PCR Master Mix (TOYOBO, Shanghai, China). The Ct values for genes of interest were normalized to GAPDH, and expressions of genes are represented as 2-[△Ct] or 2-[△△Ct] for fold change under control conditions. All the primers used for qRT-PCR are listed in Table S7.

### 4.3. Cell Culture and Erythroid Differentiation

K562 cells were maintained in RPMI Medium 1640 Thermo Fisher Scientific, Waltham, MA, USA), while HEK-293T cells were maintained in DMEM Medium (Thermo Fisher Scientific, Waltham, MA, USA) supplemented with 10% fetal bovine serum (Thermo Fisher Scientific, Waltham, MA, USA) in a humidified atmosphere of 5% CO_2_ at 37 ℃. HUDEP2 cells were cultured as described previously [34]. Briefly, StemSpan SFEM (Stemcell Technologies, Vancouver, British Columbia, Canada) was supplemented with dexamethasone (1 μM) (Novoprotein, Shanghai, China), doxycycline (1 μg/mL) (Novoprotein, Shanghai, China), erythropoietin (3 units/mL) (Novoprotein, Shanghai, China), human SCF (100 ng/mL) (Novoprotein, Shanghai, China), and 1% penicillin/streptomycin (Thermo Fisher Scientific, Waltham, MA, USA) in a humidified atmosphere of 5% CO_2_ at 37 ℃. For the erythroid differentiation culture system, HUDEP2 cells were induced as described previously [34]. Briefly, HUDEP2 cells were cultured in IMDM (Sigma, Kawasaki, Japan) containing 10% α-tocopherol (20 ng/mL) (Sigma, Kawasaki, Japan), linoleic acid (4 ng/mL) (Sigma, Kawasaki, Japan), cholesterol (200 ng/mL) (Sigma, Kawasaki, Japan), sodium selenite (2 ng/mL) (Sigma, Kawasaki, Japan), holo-transferrin (200 mg/mL) (Sigma, Kawasaki, Japan), human insulin (10 mg/mL) (Sigma, Kawasaki, Japan), ethanolamine (10 mM) (Sigma, Kawasaki, Japan), 2-ME (0.1 mM) (Sigma, Kawasaki, Japan), D-mannitol (14.57 mg/mL) (Sigma, Kawasaki, Japan), mifepristone (an antagonist of glucocorticoid receptor, 1 mM) (Sigma, Kawasaki, Japan) and EPO (5 IU/mL) (Novoprotein, Shanghai, China).

### 4.4. RNA-Seq

RNA was extracted from HUDEP2 HBB-KO cells (n = 3) or HUDEP2 WT cells (n = 3) using TRIzol. Sequencing libraries were prepared with an NEBNext^®^ Ultra™ RNA Library Prep Kit (Illumina, San Diego, CA, USA) Raw data were trimmed to remove adapters and low-quality reads using Trimmomatic [35]. Clean reads were aligned to the human reference *Homo sapiens* GRCh38/hg38 with Hisat2 [36]. Transcript quantification was performed using DESeq2 [37]. Differentially expressed genes (DEGs) were analyzed by FPKM [38] using fold changes (FDR ≤ 0.05, Fold change ≥ 2 or Fold change ≤ 0.5). Principal component analysis (PCA) was performed using factoextra [39] in R packages. Heat map analysis was performed using pheatmap [40] in R packages. Gene Ontology was performed using clusterprofiler [41] in R packages.

### 4.5. CUT&Tag and ATAC-Seq

CUT&Tag was performed using antibodies against histone H3k4me3 (Abcam, Cambridge, UK) in HUDEP2 HBB-KO cells (n = 3) or HUDEP2 WT cells (n = 3) with the NovoNGS^®^ CUT&Tag 3.0 High-Sensitivity Kit (for Illumina^®^) (Novoprotein, Shanghai, China) according to the manufacturer’s recommendations. ATAC-seq was performed following the manufacturer’s instructions (Novoprotein, Shanghai, China). A sequencing library was prepared using NEBNext Ultra II DNA Library Prep Kit for Illumina (NEB, Ipswich, MA, USA) following the manufacturer’s instructions. Paired-end 150 bp reads were generated on an Illumina NextSeq500. FastQC (a quality control tool for high throughput sequence data: http://www.bioinformatics.babraham.ac.uk/projects/fastqc/, accessed on 15 November 2021) was used for initial quality control of reads. All samples were processed according to the ENCODE guidelines for unreplicated transcription factor ChIP-seq analysis. Genomic track figures of all sample peaks were modified from the visualizations on the UCSC Genome Browser (http://genome.ucsc.edu/, accessed on 15 November 2021).

### 4.6. Proteome and Phosphoproteome Analysis

Proteins were digested with trypsin (Sigma, Kawasaki, Japan) utilizing the FASP method [42]. Peptide mixtures were labeled with 6-plex TMT reagents [23] according to the manufacturer’s protocol. Labelled peptides were separated by HPLC. Phosphopeptide enrichment was performed as previously described [43]. Briefly, peptide fractionations were mixed, lyophilized and enriched by TiO_2_ affinity chromatography. LC-MS/MS analysis was performed using an Orbitrap Exploris 480 mass spectrometer coupled with a nanoflow EASY-nLC 1000 system. A two-column system was adopted for all analyses. The mobile phases were 0.1% formic acid in H_2_O (Solvent A) and 0.1% formic acid in ACN (Solvent B). The peptide separation was performed as follows: 2–5% B for 2 min, 5–28% B for 98 min, 28–35% B for 5 min, 35–90% B for 2 min, and 90% B for 13 min at a flow rate of 200 nL/min. Data-dependent analysis was employed in MS analysis. The 15 most abundant ions in each MS scan were automatically selected and fragmented in the HCD mode. Raw data were processed by Proteome Discover (Version 1.4, Thermo Fisher Scientific, Waltham, MA, USA) and matched against the *Homo sapiens* database (UP000005640) scoring through the Mascot server (Version 2.3, Matrix Science, London, UK). Differentially expressed proteins (DEPs) were analyzed using fold changes (FDR ≤ 0.05, Fold change ≥ 2 or Fold change ≤ 0.5). PCA was performed using factoextra [39] in R packages. Heat maps were analyzed using pheatmap [40] in R packages. Gene Ontology and gene set enrichment analysis was performed using clusterprofiler [41] in R packages. Analysis of kinase activity was carried out using the KSEA website [32]. Protein subcellular localization was analyzed by WoLF PSORT [31].

### 4.7. Cytobiology

Cell viability was measured by trypan blue staining following the manufacturer’s instructions (Abcam, Cambridge, UK). The cell cycle assay was performed as previously described [44]. Briefly, HUDEP2 HBB-KO and HUDEP2 WT cells were washed with PBS, resuspended in PBS containing Triton X-100 (3‰) (Sigma, Kawasaki, Japan), propidium iodide (10 mg/mL) Sigma, Kawasaki, Japan, and Rnase A (50 μg/mL) (Novoprotein, Shanghai, China), then incubated in the dark for 15 min. The cell cycle was analyzed by flow cytometry (BD Biosciences, San Jose, CA, USA). Cell proliferation was assessed using the Cell Counting Kit-8 (CCK-8) (Dojindo Laboratories, Kumamoto, Japan) following the manufacturer’s instructions. Cell apoptosis was measured using the Dead Cell Apoptosis Kit with Annexin V Alexa Fluor™ 488 & Propidium Iodide (PI) (Thermo Fisher Scientific, Waltham, MA, USA) following the manufacturer’s instructions. The production of ROS in HUDEP2 HBB-KO and HUDEP2 WT cells was measured with a ROS detection kit (Beyotime, Shanghai, China) following the manufacturer’s instructions.

## Data Availability

Data are available via ProteomeXchange with identifier PXD030182.

## Supplementary Materials

The supporting information can be downloaded at: https://www.mdpi.com/article/10.3390/ijms23052807/s1.
