# Supplementary material for: Multi-Omics Analysis in β-Thalassemia Using an HBB Gene-Knockout Human Erythroid Progenitor Cell Model
Source: Int J Mol Sci. 2022 Mar 4;23(5):2807. doi: 10.3390/ijms23052807 (PMC8911073; doi:10.3390/ijms23052807)
Supplement: Supplementary file 1 [file ijms-23-02807-s001.zip › ijms-1524028-supplementary.pdf]

International Journal of
*Molecular Sciences*

MDPI

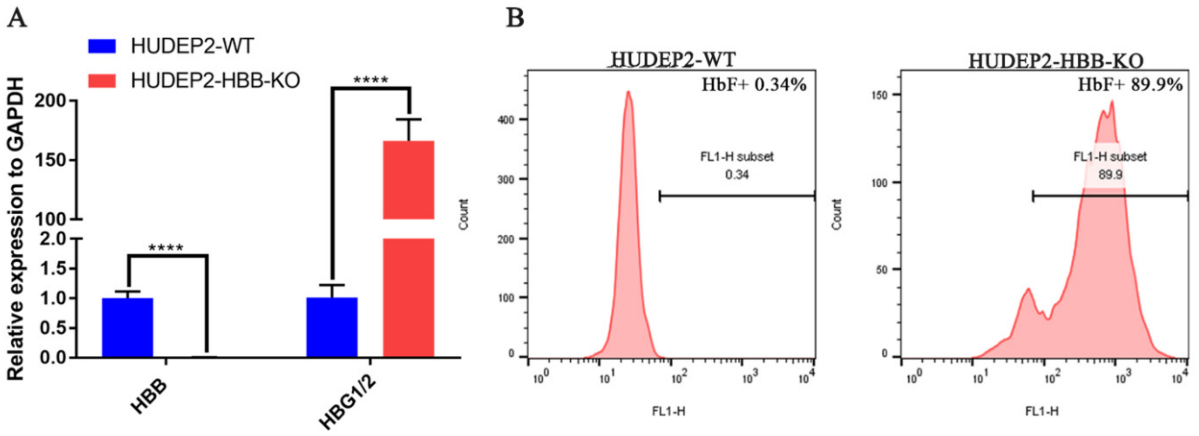


Figure S1. HBG1/2 and HbF expression in HUDEP2 HBB-KO cells. **(A)** RT-qPCR assays to determine the mRNA levels of HBB and HBG1/2 in HUDEP2 HBB-KO and HUDEP2 cells. The data are presented as mean ± SD of three biological replicates ****P<0.0001 from unpaired student t tests.**(B)** Representative flow cytometry dot plots in HUDEP2 HBB KO and HUDEP2 cells stained for fetal hemoglobin. The data are presented as mean of three biological replicates.

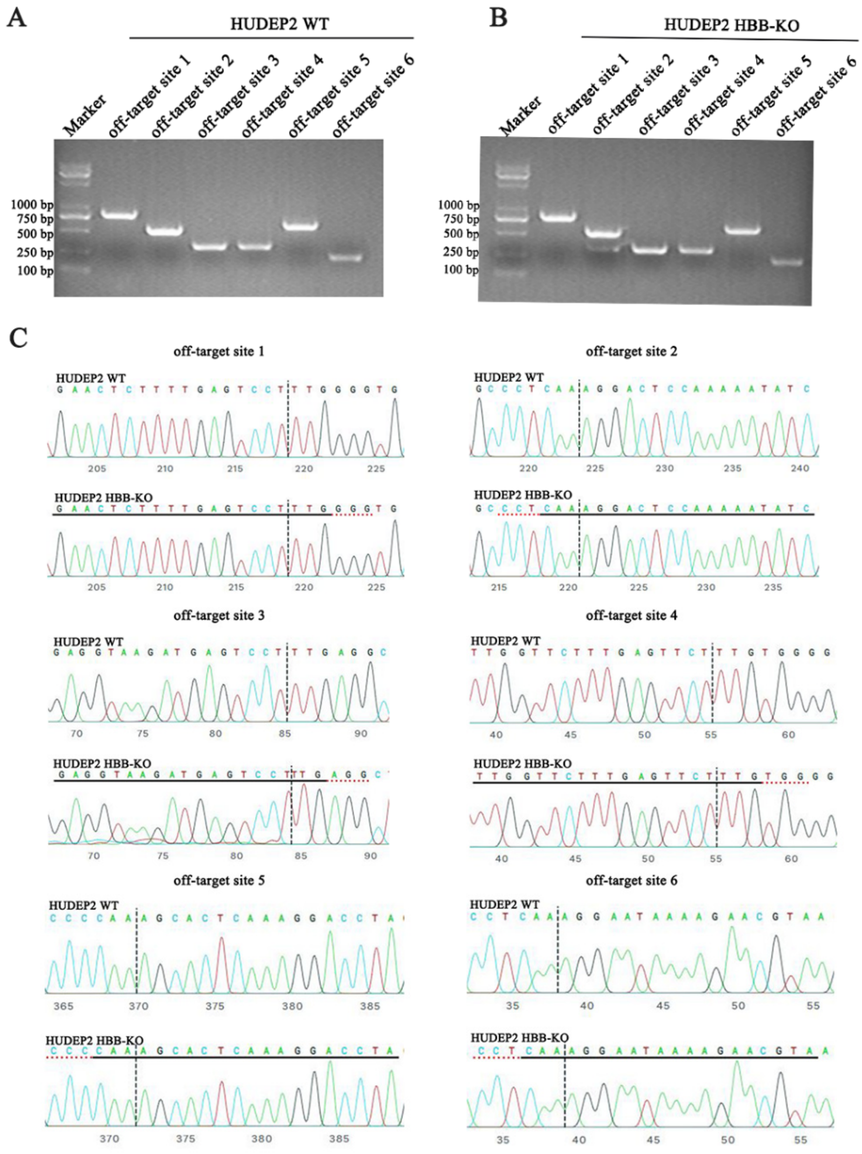

Figure S2. the detection of CRISPR off-target effect.**(A)** PCR amplified the potential off-target sites of the gRNAs used in the construction of HUDEP2 HBB-KO cells. **(B)** Sanger-seq of the potential off-target sites of the gRNAs used in the construction of HUDEP2 HBB-KO cells.

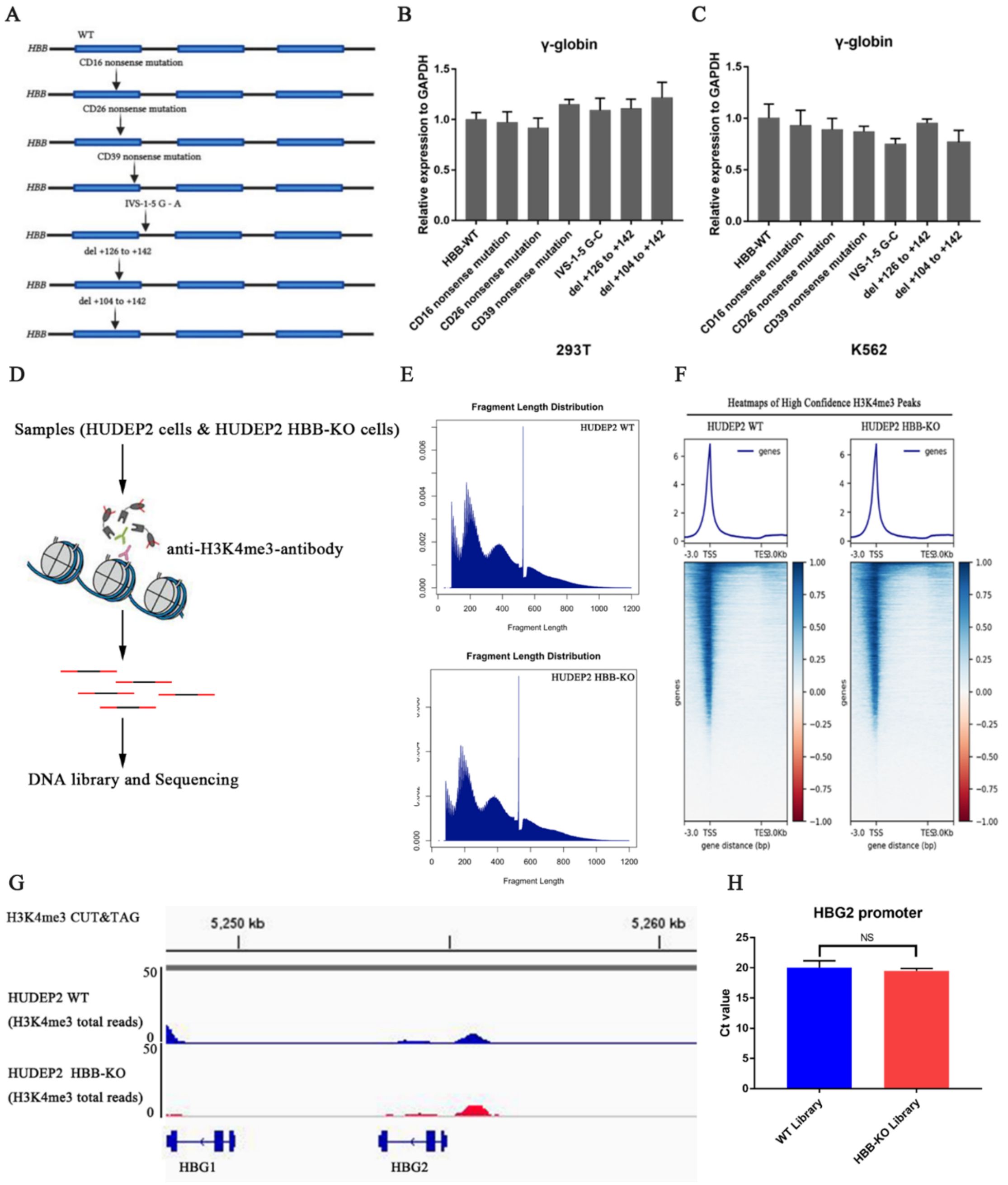


Figure S3. the detection of Genetic compensation response. **(A)** Schematic diagram of experimental design. **(B)** RT-qPCR assays to determine the γ-globin expression that was induced by HBB truncated nonsense mutant in 293T cells. **(C)** RT-qPCR assays to determine the γ-globin expression that was induced by HBB truncated nonsense mutant in K562 cells. **(D)** Schematic diagram of CUT&Tag assay. **(E)** Fragment length distribution of CUT&Tag Library. **(F)** Heatmap of H3K4me3 CUT&Tag. **(G)** Representative H3K4me3 CUT&Tag track in *HBG1/2* gene locus. **(H)** qPCR detected the H3K4me3 binding site in *HBG1/2* gene locus.

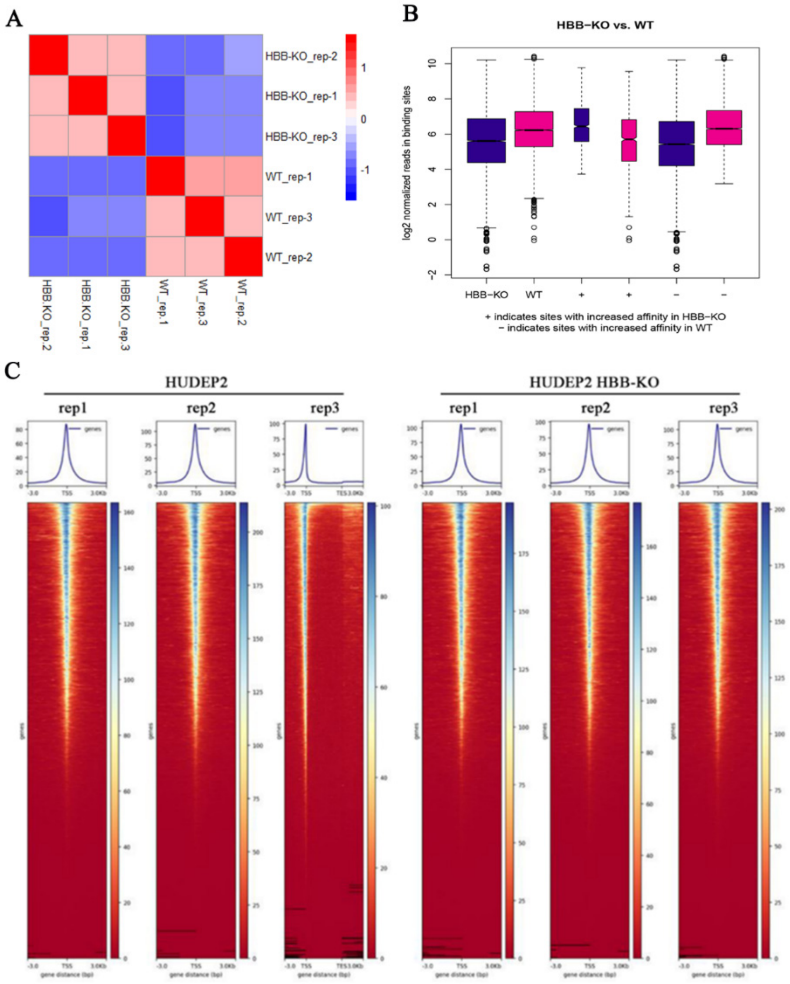


Figure S4. Quality control analysis of ATAC-seq. **(A)** Spearman correlation analysis in HUDEP2 cells (n=3) and HUDEP2 HBB-KO cells (n=3). **(B)** Total reads analysis of ATAC-seq. **(C)** TSS-heat map analysis in HUDEP2 cells(n=3) and HUDEP2 HBB-KO cells (n=3).

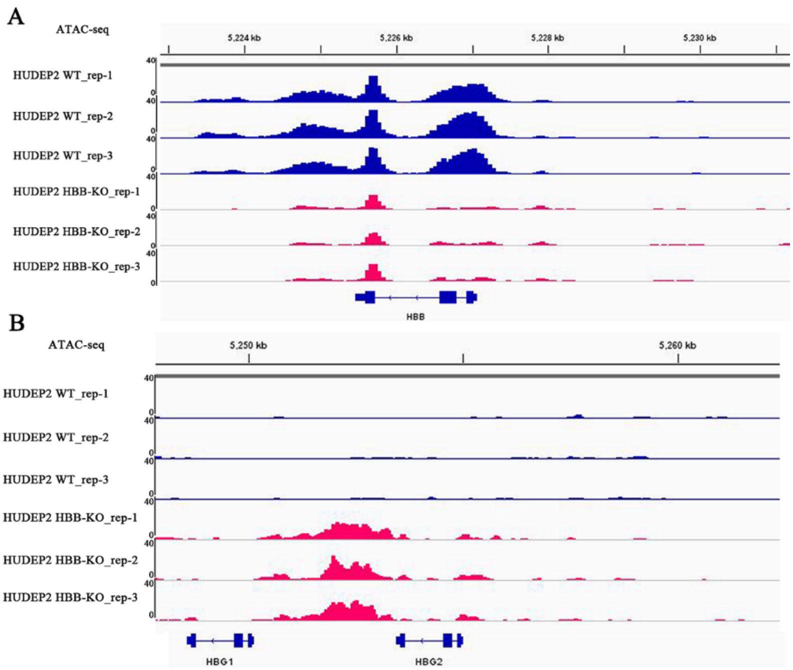


Figure S5. The analysis of HBB and HBG1/2 ATAC-seq track. **(A)** *HBB* ATAC-seq track in HUDEP2 HBB-KO and HUDEP2 WT cells. **(B)** *HBG1/2* ATAC-seq track in HUDEP2 HBB-KO and HUDEP2 WT cells.

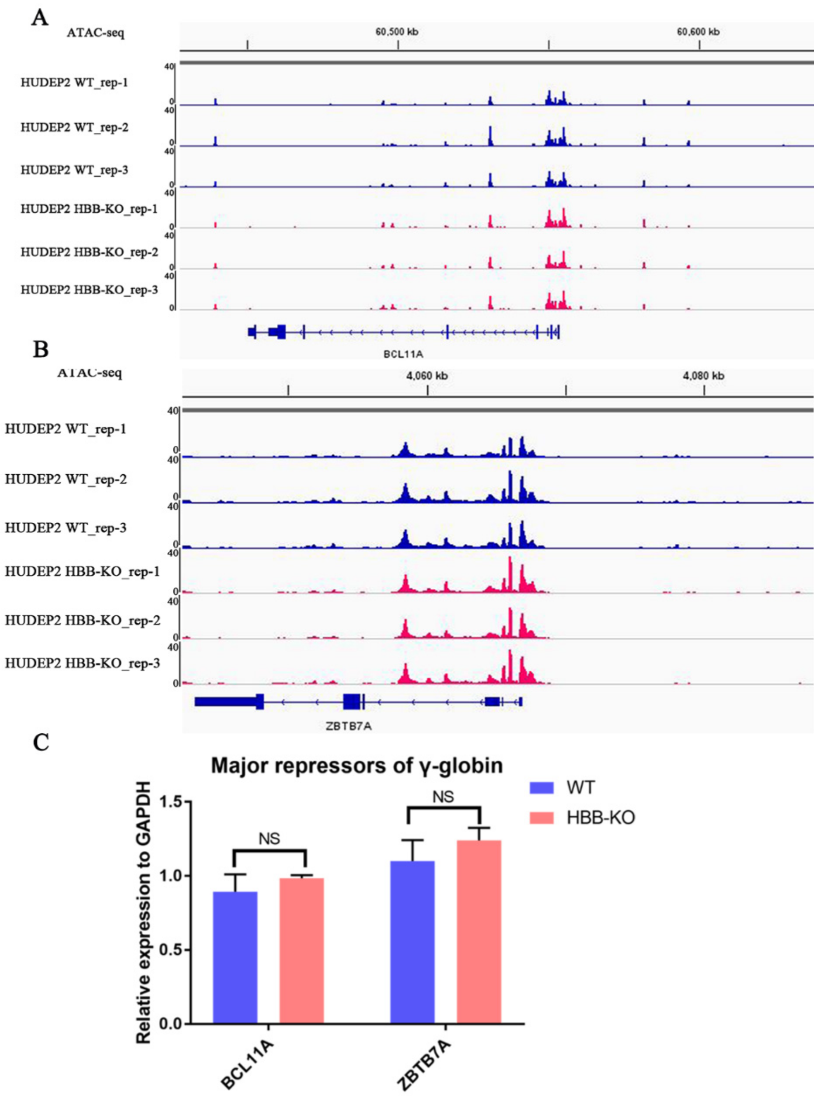


Figure S6. The analysis of BCL11A and ZBTB7A ATAC-seq track. **(A)** BCL11A ATAC-seq track in HUDEP2 HBB-KO and HUDEP2 WT cells. **(B)** ZBTB7A ATAC-seq track in HUDEP2 HBB-KO and HUDEP2 WT cells.**(C)** RT-qPCR assays to determine the BCL11A and ZBTB7A expression in HUDEP2 HBB-KO and HUDEP2 WT cells. The data are presented as mean ± SD of three biological replicates.

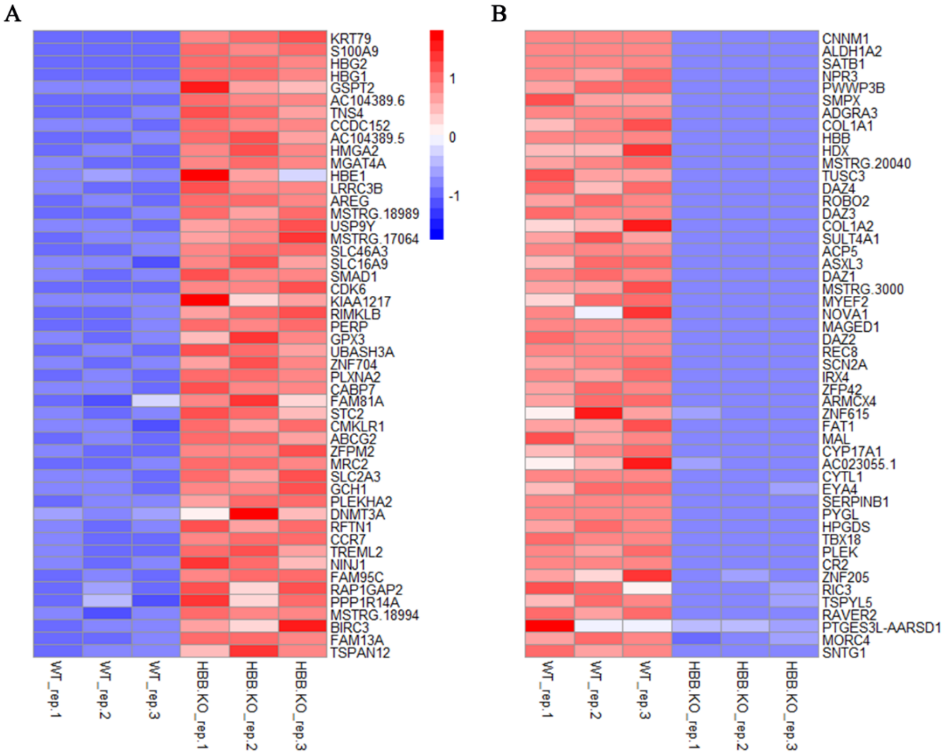


Figure S7. Heat map analysis of DEGs. **(A)** Heat map of up-regulated genes (HBB-KO vs WT). **(B)** Heat map of down-regulated genes (HBB-KO vs WT).

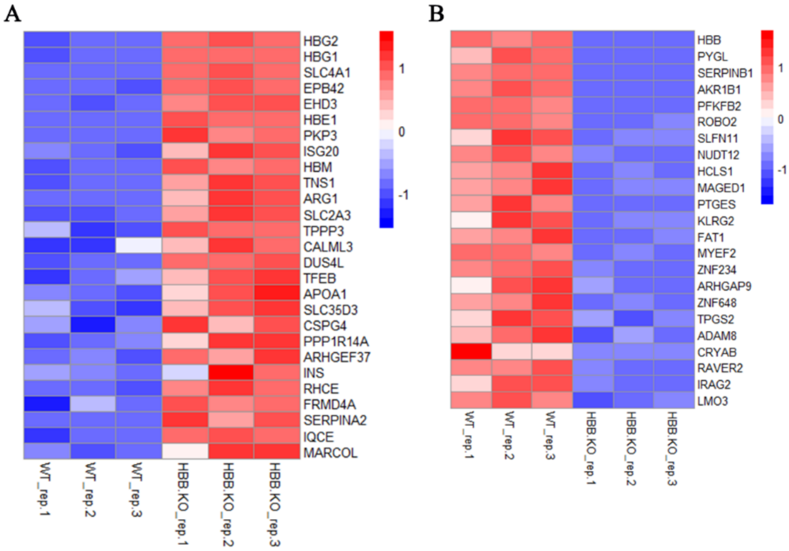


Figure S8. Heat map analysis of DEPs. **(A)** Heat map of up-regulated proteins (HBB-KO vs WT). **(B)** Heat map of down-regulated proteins (HBB-KO vs WT).

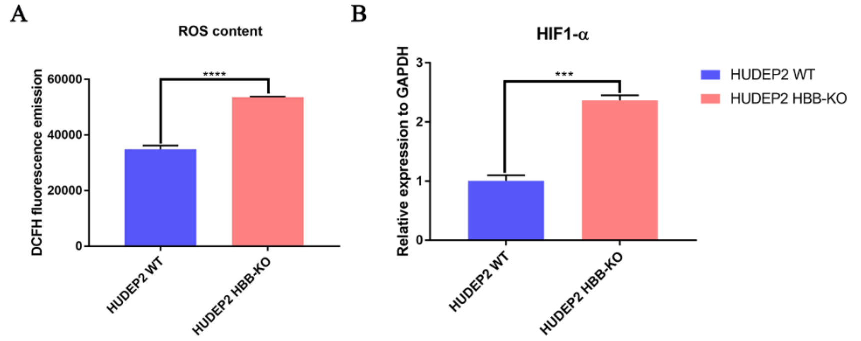


Figure S9. ROS content and HIF1 expression in HUDEP2 HBB-KO and HUDEP2 WT cells. **(A)** ROS content analysis in HUDEP2 HBB-KO and HUDEP2 WT cells. **(B)** HIF1 expression in HUDEP2 HBB-KO and HUDEP2 WT cells.

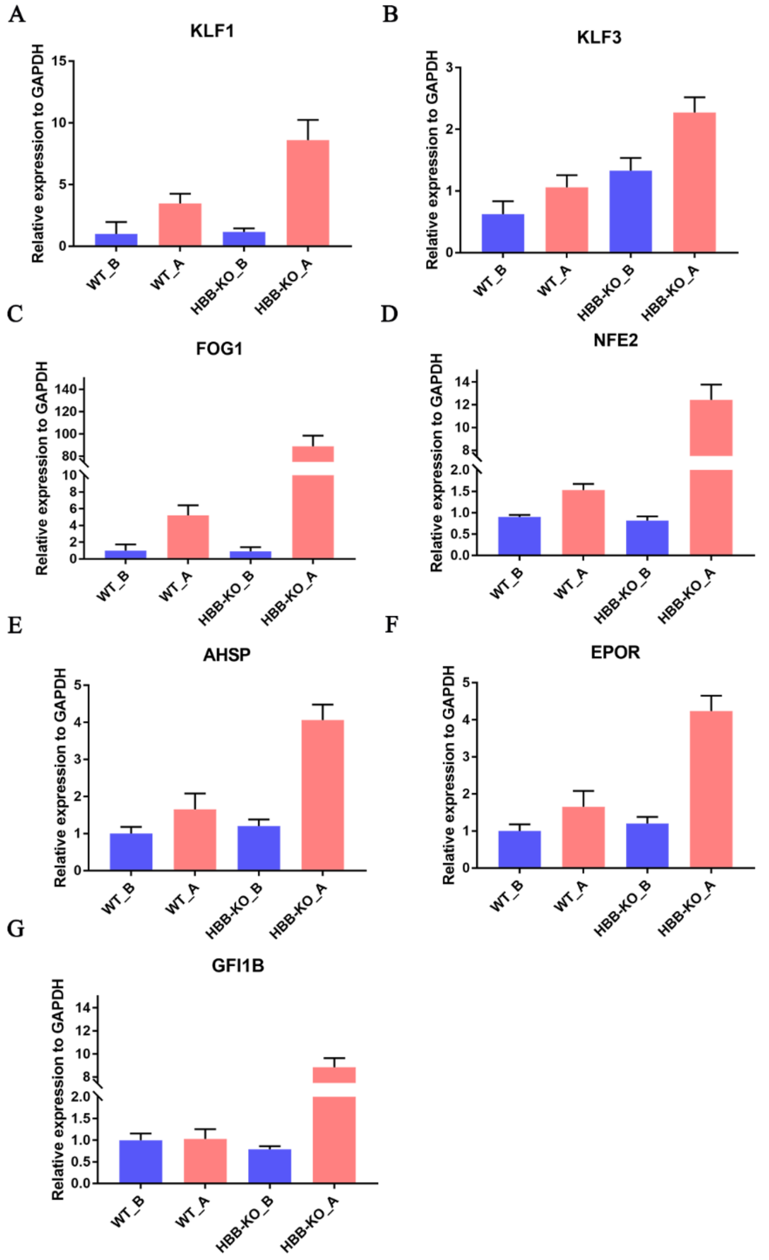


Figure S10. erythroid markers genes expression in HUDEP2 HBB-KO and HUDEP2 WT cells. **(A-G)** RT-qPCR assays to determine KLF1, KLF3, FOG1, NFE2, AHSP, EPOR, GFI1B expression.

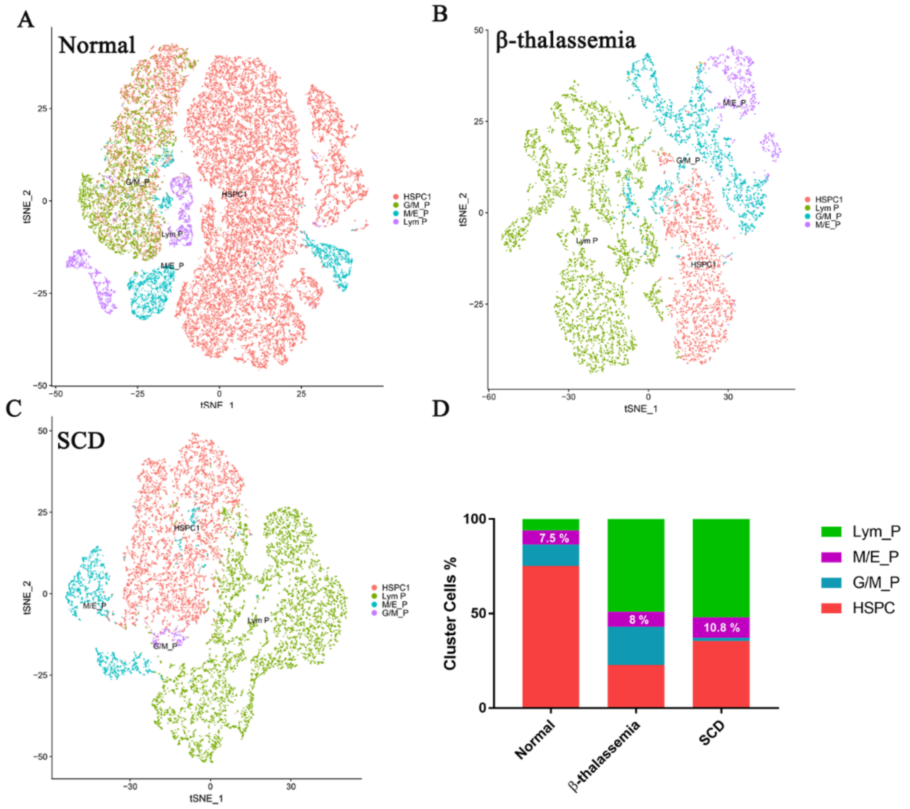


Figure S11. scRNA-seq analysis of β-thalassemia and sickle anemia patients. **(A)** tSNE analysis of CD34+ HSCs from normal people. **(B)** tSNE analysis of CD34+ HSCs from β-thalassemia patients. **(C)** tSNE analysis of CD34+ HSCs from sickle anemia patients. **(D)** statistics analysis of tSNE.

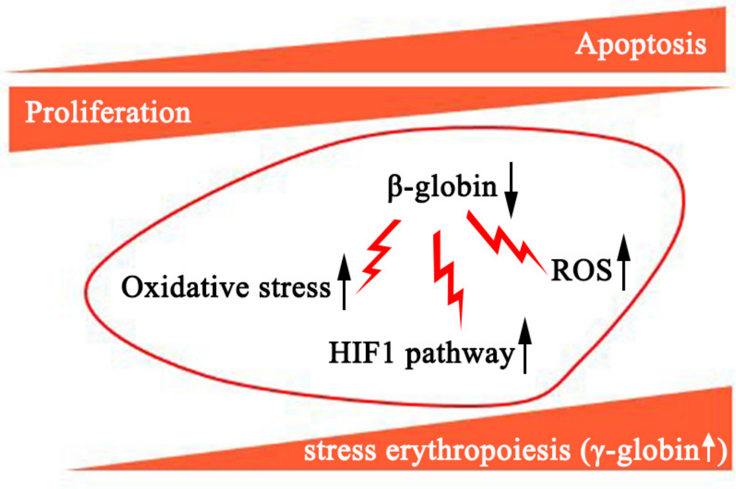


Figure S12. work model.

Table S1. The potential off-targets of gRNA(5'-GAGGTTCTTTGAGTCCTTTG -3') in the construction of HUDEP2 HBB-KO cells.

| The potential off-targets of gRNA(5'-GAGGTTCTTTGAGTCCTTTG -3') in the construction of HUDEP2 HBB-KO cells | | | | | | | | |
|---|---|---|---|---|---|---|---|---|
| off-target | Chromosome | Start | End | Target Sequence (5'-3') | PAM | Position | Gene Name | Gene Id |
| off-target site 1 | chr2 | 1.91E+08 | 1.91E+08 | GAACTCTTTTGAGTCCTTTG | GGG | Intronic | MYO1B | ENSG00000128641 |
| off-target site 2 | chr18 | 45638937 | 45638959 | GATATTTTTGGAGTCCTTTG | AGG | Intronic | SLC14A2 | ENSG00000132874 |
| off-target site 3 | chr15 | 42338488 | 42338510 | GAGGTAAGATGAGTCCTTTG | AGG | Exonic | GANC | ENSG00000214013 |
| off-target site 4 | chr4 | 2496144 | 2496166 | TTGGTTCTTTGAGTTCTTTG | TGG | Intronic | RNF4 | ENSG00000063978 |

| | | | | | | | | |
|---|---|---|---|---|---|---|---|---|
| off-target site 5 | chr2 | 56193010 | 56193032 | TAGGTCCTTTGAGTGCTTTG | GGG | Exonic | CCDC85A | ENSG00000055813 |
| off-target site 6 | chr1 | 1.72E+08 | 1.72E+08 | TACGTTCTTTTATTCCTTTG | AGG | Intronic | DNM3 | ENSG00000197959 |

Table S2. The up-enriched peaks annotation of ATAC-seq.

| The up-enriched peaks annotation of ATAC-seq | | | | | | | | | | | | | | |
|---|---|---|---|---|---|---|---|---|---|---|---|---|---|---|
| seqnames | SYMBOL | start | end | width | strand | Conc | Conc_HBB-KO | Conc_WT | Fold ??HBB-KO vs WT) | p.value | FDR | log10FDR | log2FC | sig |
| chr18 | YES1 | 814221 | 814621 | 401 | * | 5.58 | 6.08 | 4.8 | 1.07 | 1.59E-07 | 8.33E-06 | 5.079355 | 1.07 | up |
| chr12 | WNK1 | 873983 | 874383 | 401 | * | 5.39 | 5.9 | 4.6 | 1.05 | 5.1E-07 | 2.31E-05 | 4.636388 | 1.05 | up |
| chr20 | LOC100289473 | 1713189 | 1713589 | 401 | * | 5.26 | 5.79 | 4.4 | 1.13 | 3.66E-07 | 1.74E-05 | 4.759451 | 1.13 | up |
| chr18 | METTL4 | 2325432 | 2325832 | 401 | * | 5.35 | 5.91 | 4.42 | 1.21 | 1.02E-07 | 5.61E-06 | 5.251037 | 1.21 | up |
| chr19 | ZNF77 | 2944832 | 2945232 | 401 | * | 7.69 | 8.19 | 6.93 | 1.2 | 5.04E-26 | 3.68E-23 | 22.43415 | 1.2 | up |
| chr6 | HTATSF1P2 | 3026746 | 3027146 | 401 | * | 5.96 | 6.46 | 5.2 | 1.08 | 1.1E-08 | 7.7E-07 | 6.113509 | 1.08 | up |
| chr17 | ALOX15 | 4573623 | 4574023 | 401 | * | 5.34 | 5.91 | 4.36 | 1.33 | 8.31E-09 | 5.96E-07 | 6.224754 | 1.33 | up |
| chr19 | TICAM1 | 4841007 | 4841407 | 401 | * | 5.51 | 6.06 | 4.62 | 1.2 | 3.41E-08 | 2.11E-06 | 5.675718 | 1.2 | up |
| chr11 | HBD | 5251889 | 5252289 | 401 | * | 5.19 | 6.17 | 0.09 | 5.31 | 8.14E-13 | 1.29E-10 | 9.88941 | 5.31 | up |
| chr20 | SHLD1 | 5719887 | 5720287 | 401 | * | 6.87 | 7.32 | 6.2 | 1.01 | 1.68E-12 | 2.50E-10 | 9.60206 | 1.01 | up |
| chr18 | MIR3976HG | 5738573 | 5738973 | 401 | * | 4.54 | 5.2 | 3.26 | 1.66 | 1.97E-07 | 0.00001 | 5 | 1.66 | up |
| chr17 | SHBG | 7514002 | 7514402 | 401 | * | 6.11 | 6.63 | 5.3 | 1.17 | 1.88E-10 | 1.92E-08 | 7.716699 | 1.17 | up |
| chr18 | RAB12 | 8602039 | 8602439 | 401 | * | 5.85 | 6.38 | 5.02 | 1.19 | 7.91E-10 | 7.16E-08 | 7.145087 | 1.19 | up |
| chr18 | MTCL1 | 8835602 | 8836002 | 401 | * | 6.59 | 7.05 | 5.9 | 1.03 | 2.06E-11 | 2.55E-09 | 8.59346 | 1.03 | up |
| chr18 | NDUFV2 | 9009021 | 9009421 | 401 | * | 7.37 | 7.85 | 6.64 | 1.13 | 2.70E-18 | 9.12E-16 | 15.04001 | 1.13 | up |
| chr18 | PPP4R1 | 9608216 | 9608616 | 401 | * | 4.99 | 5.54 | 4.08 | 1.14 | 1.94E-06 | 7.36E-05 | 4.133122 | 1.14 | up |
| chr18 | TXNDC2 | 9877135 | 9877535 | 401 | * | 8.01 | 8.48 | 7.31 | 1.11 | 4.02E-27 | 3.18E-24 | 23.49757 | 1.11 | up |
| chr18 | TXNDC2 | 9885565 | 9885965 | 401 | * | 5.32 | 5.87 | 4.42 | 1.19 | 1.5E-07 | 7.89E-06 | 5.102923 | 1.19 | up |
| chr20 | JAG1 | 10664989 | 10665389 | 401 | * | 4.24 | 4.87 | 3.11 | 1.23 | 2.88E-05 | 0.000725 | 3.139662 | 1.23 | up |
| chr18 | PIEZO2 | 10973873 | 10974273 | 401 | * | 6.69 | 7.22 | 5.85 | 1.27 | 1.77E-16 | 4.74E-14 | 13.32422 | 1.27 | up |
| chr16 | CLEC16A | 11133318 | 11133718 | 401 | * | 4.5 | 5.29 | 2.62 | 2.52 | 3.19E-10 | 3.11E-08 | 7.50724 | 2.52 | up |
| chr19 | ZNF833P | 11814003 | 11814403 | 401 | * | 5.12 | 5.65 | 4.28 | 1.03 | 7.76E-06 | 0.000241 | 3.617983 | 1.03 | up |

| | | | | | | | | | | | | | | |
|---|---|---|---|---|---|---|---|---|---|---|---|---|---|---|
| chr18 | CHMP1B | 11851142 | 11851542 | 401 | * | 7.19 | 7.64 | 6.52 | 1.03 | 7.25E-15 | 1.55E-12 | 11.80967 | 1.03 | up |
| chr11 | MICAL2 | 12224527 | 12224927 | 401 | * | 5.26 | 6.01 | 3.62 | 2.31 | 1.59E-14 | 3.21E-12 | 11.49349 | 2.31 | up |
| chr18 | TUBB6 | 12302443 | 12302843 | 401 | * | 7.71 | 8.16 | 7.05 | 1.05 | 8.40E-21 | 3.91E-18 | 17.40782 | 1.05 | up |
| chr2 | MIR4262 | 12325838 | 12326238 | 401 | * | 5.38 | 5.88 | 4.6 | 1.04 | 7.24E-07 | 3.15E-05 | 4.501689 | 1.04 | up |
| chr18 | AFG3L2 | 12359574 | 12359974 | 401 | * | 7.42 | 7.89 | 6.72 | 1.1 | 1.22E-18 | 4.30E-16 | 15.36653 | 1.1 | up |
| chrY | GYG2P1 | 12537398 | 12537798 | 401 | * | 4.87 | 5.76 | 2.16 | 3.41 | 4.67E-15 | 1.01E-12 | 11.99568 | 3.41 | up |
| chr12 | MIR613 | 12899708 | 12900108 | 401 | * | 2.98 | 3.73 | 1.31 | 1.15 | 0.000601 | 0.00866 | 2.062482 | 1.15 | up |
| chr3 | IQSEC1 | 13022282 | 13022682 | 401 | * | 5.36 | 5.92 | 4.42 | 1.27 | 1.68E-08 | 1.13E-06 | 5.946922 | 1.27 | up |
| chr18 | CEP192 | 13055093 | 13055493 | 401 | * | 5.98 | 6.77 | 4.1 | 2.6 | 4.27E-24 | 2.75E-21 | 20.56067 | 2.6 | up |
| chr18 | ANKRD20A5P | 14178808 | 14179208 | 401 | * | 5.94 | 6.59 | 4.71 | 1.8 | 3.81E-16 | 9.71E-14 | 13.01278 | 1.8 | up |
| chr3 | PLCL2 | 16849201 | 16849601 | 401 | * | 4.14 | 4.82 | 2.85 | 1.55 | 4.56E-06 | 0.000154 | 3.812479 | 1.55 | up |
| chr12 | PLEKHA5 | 19382019 | 19382419 | 401 | * | 4.13 | 4.73 | 3.1 | 1.08 | 9.38E-05 | 0.00194 | 2.712198 | 1.08 | up |
| chrY | TTTY14 | 21307389 | 21307789 | 401 | * | 3.26 | 3.99 | 1.74 | 1.22 | 0.000317 | 0.00515 | 2.288193 | 1.22 | up |
| chr20 | FOXA2 | 22757422 | 22757822 | 401 | * | 5.16 | 5.89 | 3.65 | 2.16 | 8.93E-13 | 1.40E-10 | 9.853872 | 2.16 | up |
| chr2 | EFR3B | 25251760 | 25252160 | 401 | * | 5.39 | 6.18 | 3.48 | 2.6 | 6.37E-17 | 1.81E-14 | 13.74232 | 2.6 | up |
| chr2 | EFR3B | 25277167 | 25277567 | 401 | * | 4.18 | 4.83 | 2.96 | 1.39 | 9.69E-06 | 0.000291 | 3.536107 | 1.39 | up |
| chr1 | MAP3K6 | 27686602 | 27687002 | 401 | * | 5.51 | 6 | 4.76 | 1.03 | 4.02E-07 | 1.88E-05 | 4.725842 | 1.03 | up |
| chr13 | PAN3-AS1 | 28718817 | 28719217 | 401 | * | 7.25 | 7.73 | 6.51 | 1.15 | 2.48E-19 | 9.42E-17 | 16.02595 | 1.15 | up |
| chr18 | CCDC178 | 30926858 | 30927258 | 401 | * | 4.08 | 4.87 | 2.17 | 2.47 | 5.97E-08 | 3.48E-06 | 5.458421 | 2.47 | up |
| chr2 | GALNT14 | 31235566 | 31235966 | 401 | * | 4.9 | 5.6 | 3.47 | 2.01 | 2.71E-10 | 2.68E-08 | 7.571865 | 2.01 | up |
| chr20 | BPIFB4 | 31670676 | 31671076 | 401 | * | 6.04 | 6.55 | 5.25 | 1.15 | 2.64E-10 | 2.62E-08 | 7.581699 | 1.15 | up |
| chr11 | WT1 | 32333512 | 32333912 | 401 | * | 4.53 | 5.1 | 3.57 | 1.1 | 1.89E-05 | 0.000511 | 3.291579 | 1.1 | up |
| chr3 | TRIM71 | 32902989 | 32903389 | 401 | * | 4.69 | 5.35 | 3.43 | 1.64 | 8.03E-08 | 4.54E-06 | 5.342944 | 1.64 | up |
| chr8 | NRG1 | 32927693 | 32928093 | 401 | * | 3.98 | 4.64 | 2.73 | 1.4 | 2.38E-05 | 0.00062 | 3.207608 | 1.4 | up |
| chr13 | STARD13 | 34157025 | 34157425 | 401 | * | 5.17 | 5.73 | 4.24 | 1.24 | 1.25E-07 | 6.74E-06 | 5.17134 | 1.24 | up |
| chr3 | DCLK3 | 36679366 | 36679766 | 401 | * | 4.71 | 5.33 | 3.58 | 1.43 | 4.49E-07 | 2.08E-05 | 4.681937 | 1.43 | up |
| chr17 | ZNF385C | 40224788 | 40225188 | 401 | * | 6.18 | 6.7 | 5.36 | 1.19 | 2.48E-11 | 3E-09 | 8.522879 | 1.19 | up |
| chr21 | ETS2 | 40355095 | 40355495 | 401 | * | 3.59 | 4.29 | 2.14 | 1.38 | 9.73E-05 | 0.002 | 2.69897 | 1.38 | up |

| | | | | | | | | | | | | | | |
|---|---|---|---|---|---|---|---|---|---|---|---|---|---|---|
| chr15 | KNSTRN | 40664992 | 40665392 | 401 | * | 4.93 | 5.79 | 2.47 | 3.18 | 3.10E-15 | 6.88E-13 | 12.16241 | 3.18 | up |
| chr2 | SLC8A1 | 41194260 | 41194660 | 401 | * | 5.36 | 5.99 | 4.23 | 1.6 | 1.79E-10 | 1.85E-08 | 7.732828 | 1.6 | up |
| chrX | CASK | 41442301 | 41442701 | 401 | * | 6.83 | 7.35 | 6 | 1.24 | 8.27E-16 | 2.00E-13 | 12.69897 | 1.24 | up |
| chr10 | LOC441666 | 42647092 | 42647492 | 401 | * | 5.91 | 6.61 | 4.51 | 2.04 | 2.23E-18 | 7.60E-16 | 15.11919 | 2.04 | up |
| chr5 | CCDC152 | 42756598 | 42756998 | 401 | * | 4.06 | 4.81 | 2.36 | 2.14 | 6.31E-07 | 2.78E-05 | 4.555955 | 2.14 | up |
| chr1 | SLC2A1 | 43454421 | 43454821 | 401 | * | 6.46 | 6.99 | 5.6 | 1.28 | 3.74E-14 | 7.15E-12 | 11.14569 | 1.28 | up |
| chr13 | ENOX1 | 44244242 | 44244642 | 401 | * | 5.26 | 5.92 | 4 | 1.77 | 4.73E-11 | 5.38E-09 | 8.269218 | 1.77 | up |
| chr18 | SKOR2 | 44887210 | 44887610 | 401 | * | 5.45 | 6.22 | 3.72 | 2.41 | 1.26E-16 | 3.44E-14 | 13.46344 | 2.41 | up |
| chr4 | GNPDA2 | 45294549 | 45294949 | 401 | * | 3.96 | 4.61 | 2.78 | 1.25 | 5.24E-05 | 0.0012 | 2.920819 | 1.25 | up |
| chr21 | TRAPPC10 | 45422335 | 45422735 | 401 | * | 4.42 | 4.98 | 3.49 | 1.01 | 7.28E-05 | 0.00157 | 2.8041 | 1.01 | up |
| chr1 | MAST2 | 46394082 | 46394482 | 401 | * | 4.3 | 4.87 | 3.32 | 1.06 | 6.74E-05 | 0.00147 | 2.832683 | 1.06 | up |
| chr17 | TOB1 | 48995807 | 48996207 | 401 | * | 5.65 | 6.24 | 4.62 | 1.47 | 6.39E-11 | 7.12E-09 | 8.14752 | 1.47 | up |
| chr22 | NA | 50146790 | 50147190 | 401 | * | 4.69 | 5.27 | 3.68 | 1.22 | 2.81E-06 | 0.000102 | 3.9914 | 1.22 | up |
| chrX | SNORA11D | 51743248 | 51743648 | 401 | * | 3.39 | 4.18 | 1.53 | 2 | 0.000029 | 0.000729 | 3.137272 | 2 | up |
| chr12 | KRT75 | 52834405 | 52834805 | 401 | * | 4.76 | 5.36 | 3.72 | 1.32 | 8.78E-07 | 3.72E-05 | 4.429457 | 1.32 | up |
| chr3 | ESRG | 54691190 | 54691590 | 401 | * | 3.34 | 4.14 | 1.35 | 2.14 | 2.58E-05 | 0.000663 | 3.178486 | 2.14 | up |
| chr12 | HSD17B6 | 57208861 | 57209261 | 401 | * | 4.58 | 5.28 | 3.19 | 1.9 | 3.42E-08 | 2.12E-06 | 5.673664 | 1.9 | up |
| chrX | FAAH2 | 57286673 | 57287073 | 401 | * | 3.17 | 4.03 | 0.7 | 2.35 | 3.29E-05 | 0.000807 | 3.093126 | 2.35 | up |
| chr18 | MC4R | 58195235 | 58195635 | 401 | * | 4.52 | 5.1 | 3.55 | 1.08 | 4.77E-05 | 0.00111 | 2.954677 | 1.08 | up |
| chr15 | ICE2 | 60761101 | 60761501 | 401 | * | 4.1 | 4.74 | 2.96 | 1.2 | 5.11E-05 | 0.00117 | 2.931814 | 1.2 | up |
| chr15 | C2CD4B | 62453335 | 62453735 | 401 | * | 5.08 | 5.62 | 4.2 | 1.07 | 7.76E-06 | 0.000241 | 3.617983 | 1.07 | up |
| chr11 | MIR548AR | 65279751 | 65280151 | 401 | * | 4.33 | 5.04 | 2.89 | 1.83 | 4.23E-07 | 1.97E-05 | 4.705534 | 1.83 | up |
| chr12 | MSRB3 | 65822923 | 65823323 | 401 | * | 4.03 | 4.77 | 2.45 | 1.98 | 9.02E-07 | 3.81E-05 | 4.419075 | 1.98 | up |
| chr12 | MSRB3 | 65824916 | 65825316 | 401 | * | 4.62 | 5.21 | 3.61 | 1.22 | 3.97E-06 | 0.000137 | 3.863279 | 1.22 | up |
| chr16 | CDH5 | 66327114 | 66327514 | 401 | * | 4.16 | 4.75 | 3.15 | 1.02 | 0.000146 | 0.00277 | 2.55752 | 1.02 | up |
| chr18 | CCDC102B | 66682925 | 66683325 | 401 | * | 3.76 | 4.48 | 2.26 | 1.66 | 1.73E-05 | 0.000476 | 3.322393 | 1.66 | up |
| chr12 | CAND1 | 67759724 | 67760124 | 401 | * | 4.53 | 5.11 | 3.56 | 1.08 | 3.32E-05 | 0.000814 | 3.089376 | 1.08 | up |
| chr15 | ITGA11 | 68795343 | 68795743 | 401 | * | 5.15 | 5.77 | 4.05 | 1.52 | 4.15E-09 | 3.21E-07 | 6.493495 | 1.52 | up |

| | | | | | | | | | | | | | | |
|---|---|---|---|---|---|---|---|---|---|---|---|---|---|---|
| chr17 | KCNJ2 | 69093276 | 69093676 | 401 | * | 3.69 | 4.5 | 1.62 | 2.5 | 1.11E-06 | 4.58E-05 | 4.339135 | 2.5 | up |
| chr17 | KCNJ2 | 69103585 | 69103985 | 401 | * | 4.13 | 4.92 | 2.26 | 2.44 | 3.89E-08 | 2.38E-06 | 5.623423 | 2.44 | up |
| chr9 | SMC5 | 72952901 | 72953301 | 401 | * | 4.25 | 4.84 | 3.23 | 1.07 | 6.94E-05 | 0.00151 | 2.821023 | 1.07 | up |
| chr4 | LINC02499 | 74365050 | 74365450 | 401 | * | 3.9 | 4.53 | 2.78 | 1.12 | 0.000116 | 0.00231 | 2.636388 | 1.12 | up |
| chr4 | LINC02499 | 74428167 | 74428567 | 401 | * | 5.13 | 5.64 | 4.34 | 1.01 | 5.1E-06 | 0.00017 | 3.769551 | 1.01 | up |
| chr3 | FAM86DP | 75445237 | 75445637 | 401 | * | 4.89 | 5.44 | 4.01 | 1.1 | 4.72E-06 | 0.000159 | 3.798603 | 1.1 | up |
| chr18 | SALL3 | 76476969 | 76477369 | 401 | * | 4.35 | 4.99 | 3.14 | 1.46 | 2.61E-06 | 9.55E-05 | 4.019997 | 1.46 | up |
| chr14 | MIR1260A | 77698610 | 77699010 | 401 | * | 4.92 | 5.48 | 3.99 | 1.15 | 3.56E-06 | 0.000124 | 3.906578 | 1.15 | up |
| chr12 | NAV3 | 77888603 | 77889003 | 401 | * | 6.14 | 6.63 | 5.4 | 1.07 | 1.08E-09 | 9.44E-08 | 7.025028 | 1.07 | up |
| chr14 | VIPAS39 | 77914029 | 77914429 | 401 | * | 6 | 6.49 | 5.24 | 1.07 | 5.67E-09 | 4.22E-07 | 6.374688 | 1.07 | up |
| chr11 | TENM4 | 79793963 | 79794363 | 401 | * | 5.51 | 6.02 | 4.72 | 1.07 | 2.38E-07 | 1.19E-05 | 4.924453 | 1.07 | up |
| chr17 | RPL23AP87 | 81582809 | 81583209 | 401 | * | 5.45 | 6.05 | 4.41 | 1.45 | 9.56E-10 | 8.49E-08 | 7.071092 | 1.45 | up |
| chr14 | LINC00911 | 85531563 | 85531963 | 401 | * | 4.61 | 5.16 | 3.72 | 1.06 | 2.25E-05 | 0.00059 | 3.229148 | 1.06 | up |
| chr13 | SLITRK6 | 86882748 | 86883148 | 401 | * | 3.55 | 4.24 | 2.15 | 1.3 | 0.000139 | 0.00267 | 2.573489 | 1.3 | up |
| chr13 | SLITRK6 | 87124077 | 87124477 | 401 | * | 3.75 | 4.47 | 2.26 | 1.6 | 2.77E-05 | 0.000701 | 3.154282 | 1.6 | up |
| chr4 | PKD2 | 89009712 | 89010112 | 401 | * | 5.23 | 5.9 | 3.96 | 1.82 | 2.95E-11 | 3.51E-09 | 8.454693 | 1.82 | up |
| chr4 | ABCG2 | 89057007 | 89057407 | 401 | * | 4.72 | 5.58 | 2.22 | 3.15 | 2.50E-13 | 4.21E-11 | 10.37572 | 3.15 | up |
| chr1 | MIR760 | 94237439 | 94237839 | 401 | * | 4.46 | 5.09 | 3.3 | 1.42 | 1.92E-06 | 0.000073 | 4.136677 | 1.42 | up |
| chr1 | ABCD3 | 94892861 | 94893261 | 401 | * | 4.93 | 5.47 | 4.05 | 1.08 | 7.48E-06 | 0.000234 | 3.630784 | 1.08 | up |
| chr15 | LINC00924 | 96330405 | 96330805 | 401 | * | 4.1 | 4.91 | 2.11 | 2.61 | 3.08E-08 | 1.94E-06 | 5.712198 | 2.61 | up |
| chr15 | LINC00923 | 97875466 | 97875866 | 401 | * | 6.45 | 7.04 | 5.43 | 1.52 | 1.15E-17 | 3.62E-15 | 14.44129 | 1.52 | up |
| chr7 | CASTOR3 | 99839756 | 99840156 | 401 | * | 3.98 | 4.63 | 2.77 | 1.3 | 0.000036 | 0.000872 | 3.059484 | 1.3 | up |
| chr8 | MIR599 | 1E+08 | 1E+08 | 401 | * | 4.82 | 5.56 | 3.2 | 2.23 | 1.00E-10 | 1.09E-08 | 7.962574 | 2.23 | up |
| chr8 | MIR599 | 1E+08 | 1E+08 | 401 | * | 7.37 | 7.93 | 6.45 | 1.43 | 1.15E-26 | 8.87E-24 | 23.05208 | 1.43 | up |
| chr14 | MIR1247 | 1.02E+08 | 1.02E+08 | 401 | * | 5.52 | 6.19 | 4.23 | 1.89 | 1.45E-13 | 2.52E-11 | 10.5986 | 1.89 | up |
| chr7 | LRRC17 | 1.02E+08 | 1.02E+08 | 401 | * | 6.36 | 6.94 | 5.38 | 1.47 | 1.54E-16 | 4.17E-14 | 13.37986 | 1.47 | up |
| chr12 | PMCH | 1.03E+08 | 1.03E+08 | 401 | * | 3.97 | 4.63 | 2.71 | 1.39 | 2.39E-05 | 0.000623 | 3.205512 | 1.39 | up |
| chr14 | COA8 | 1.04E+08 | 1.04E+08 | 401 | * | 4.39 | 4.95 | 3.46 | 1.03 | 5.59E-05 | 0.00126 | 2.899629 | 1.03 | up |

| | | | | | | | | | | | | | | |
|---|---|---|---|---|---|---|---|---|---|---|---|---|---|---|
| chr8 | LRP12 | 1.06E+08 | 1.06E+08 | 401 | * | 3.95 | 4.65 | 2.54 | 1.64 | 7.97E-06 | 0.000246 | 3.609065 | 1.64 | up |
| chr6 | PREP | 1.06E+08 | 1.06E+08 | 401 | * | 4.41 | 5.03 | 3.29 | 1.36 | 4.48E-06 | 0.000152 | 3.818156 | 1.36 | up |
| chr9 | SLC44A1 | 1.08E+08 | 1.08E+08 | 401 | * | 5.36 | 5.88 | 4.53 | 1.08 | 6.45E-07 | 2.84E-05 | 4.546682 | 1.08 | up |
| chr12 | SSH1 | 1.09E+08 | 1.09E+08 | 401 | * | 3.75 | 4.42 | 2.45 | 1.3 | 7.81E-05 | 0.00167 | 2.777284 | 1.3 | up |
| chrX | PAK3 | 1.1E+08 | 1.1E+08 | 401 | * | 5.44 | 6.03 | 4.43 | 1.42 | 1.05E-09 | 9.26E-08 | 7.033389 | 1.42 | up |
| chr10 | PDCD4-AS1 | 1.13E+08 | 1.13E+08 | 401 | * | 2.83 | 3.73 | 0 | 1.94 | 0.000181 | 0.0033 | 2.481486 | 1.94 | up |
| chr11 | NNMT | 1.14E+08 | 1.14E+08 | 401 | * | 4.94 | 5.47 | 4.09 | 1.07 | 6.92E-06 | 0.00022 | 3.657577 | 1.07 | up |
| chr9 | INIP | 1.15E+08 | 1.15E+08 | 401 | * | 4.33 | 5.05 | 2.85 | 1.95 | 1.12E-07 | 6.11E-06 | 5.213959 | 1.95 | up |
| chr12 | MED13L | 1.17E+08 | 1.17E+08 | 401 | * | 4.51 | 5.1 | 3.51 | 1.19 | 9.88E-06 | 0.000296 | 3.528708 | 1.19 | up |
| chr11 | C2CD2L | 1.19E+08 | 1.19E+08 | 401 | * | 4.05 | 4.76 | 2.62 | 1.73 | 2.94E-06 | 0.000106 | 3.974694 | 1.73 | up |
| chr5 | PRR16 | 1.2E+08 | 1.2E+08 | 401 | * | 3.4 | 4.12 | 1.9 | 1.35 | 0.000159 | 0.00297 | 2.527244 | 1.35 | up |
| chr11 | SLC37A2 | 1.25E+08 | 1.25E+08 | 401 | * | 5.49 | 6.31 | 3.39 | 2.82 | 5.98E-20 | 2.44E-17 | 16.61261 | 2.82 | up |
| chr12 | LINC02372 | 1.27E+08 | 1.27E+08 | 401 | * | 4.38 | 5 | 3.27 | 1.31 | 6.64E-06 | 0.000212 | 3.673664 | 1.31 | up |
| chr6 | LAMA2 | 1.29E+08 | 1.29E+08 | 401 | * | 4.59 | 5.18 | 3.59 | 1.15 | 1.23E-05 | 0.000355 | 3.449772 | 1.15 | up |
| chr9 | LRRC8A | 1.32E+08 | 1.32E+08 | 401 | * | 5.27 | 5.78 | 4.49 | 1.03 | 2.05E-06 | 7.71E-05 | 4.112946 | 1.03 | up |
| chr5 | TRPC7 | 1.36E+08 | 1.36E+08 | 401 | * | 6.55 | 7.36 | 4.55 | 2.76 | 4.82E-38 | 8.00E-35 | 34.09691 | 2.76 | up |
| chr6 | MIR4465 | 1.42E+08 | 1.42E+08 | 401 | * | 5.34 | 5.93 | 4.32 | 1.4 | 3.41E-09 | 2.68E-07 | 6.571865 | 1.4 | up |
| chr8 | PLEC | 1.45E+08 | 1.45E+08 | 401 | * | 7.31 | 7.81 | 6.53 | 1.21 | 4.73E-21 | 2.29E-18 | 17.64016 | 1.21 | up |
| chr8 | GRINA | 1.45E+08 | 1.45E+08 | 401 | * | 6.14 | 6.97 | 3.95 | 2.97 | 2.45E-31 | 2.54E-28 | 27.59517 | 2.97 | up |
| chr2 | RBM43 | 1.52E+08 | 1.52E+08 | 401 | * | 5.83 | 6.31 | 5.12 | 1.01 | 8.14E-08 | 4.59E-06 | 5.338187 | 1.01 | up |
| chr5 | LINC02159 | 1.6E+08 | 1.6E+08 | 401 | * | 6.59 | 7.08 | 5.85 | 1.12 | 9.38E-13 | 1.46E-10 | 9.835647 | 1.12 | up |
| chr6 | PRKN | 1.63E+08 | 1.63E+08 | 401 | * | 5.23 | 5.88 | 4.05 | 1.66 | 4.60E-10 | 4.32E-08 | 7.364516 | 1.66 | up |
| chr3 | TNIK | 1.71E+08 | 1.71E+08 | 401 | * | 3.32 | 4.04 | 1.81 | 1.28 | 0.000257 | 0.00436 | 2.360514 | 1.28 | up |
| chr5 | CPEB4 | 1.73E+08 | 1.73E+08 | 401 | * | 5.77 | 6.39 | 4.64 | 1.63 | 4.36E-13 | 7.16E-11 | 10.14509 | 1.63 | up |
| chr5 | CPEB4 | 1.73E+08 | 1.73E+08 | 401 | * | 4.94 | 5.49 | 4.06 | 1.13 | 2.53E-06 | 9.28E-05 | 4.032452 | 1.13 | up |
| chr5 | SIMC1 | 1.76E+08 | 1.76E+08 | 401 | * | 4.59 | 5.2 | 3.49 | 1.38 | 1.66E-06 | 6.45E-05 | 4.19044 | 1.38 | up |
| chr2 | MIR561 | 1.89E+08 | 1.89E+08 | 401 | * | 4.15 | 4.76 | 3.07 | 1.13 | 5.59E-05 | 0.00126 | 2.899629 | 1.13 | up |
| chr1 | KCTD3 | 2.16E+08 | 2.16E+08 | 401 | * | 4.62 | 5.32 | 3.21 | 1.9 | 1.54E-08 | 1.04E-06 | 5.982967 | 1.9 | up |

| | | | | | | | | | | | | | | |
|---|---|---|---|---|---|---|---|---|---|---|---|---|---|---|
| chr2 | SNORA75 | 2.32E+08 | 2.32E+08 | 401 | * | 5.88 | 6.44 | 4.96 | 1.33 | 4.22E-11 | 4.87E-09 | 8.312471 | 1.33 | up |

Table S3. The down-enriched peaks anotation of ATAC-seq.

| The down-enriched peaks anotation of ATAC-seq | | | | | | | | | | | | | | |
|---|---|---|---|---|---|---|---|---|---|---|---|---|---|---|
| se-qnames | SYMBOL | start | end | width | strand | Conc | Conc_KO | Conc_WT | Fold | p.value | FDR | log10FDR | log2FC | sig |
| chr1 | CATSPERE | 2.45E+08 | 2.45E+08 | 401 | * | 4.07 | 2.88 | 4.72 | -1.31 | 2.33E-05 | 0.000609 | 3.215383 | -1.31 | down |
| chr1 | RGS7 | 2.41E+08 | 2.41E+08 | 401 | * | 4.82 | 2.45 | 5.67 | -3.06 | 1.59E-14 | 3.21E-12 | 11.49349 | -3.06 | down |
| chr1 | RGS7 | 2.41E+08 | 2.41E+08 | 401 | * | 2.88 | 0.91 | 3.69 | -1.34 | 0.000421 | 0.00647 | 2.189096 | -1.34 | down |
| chr1 | RGS7 | 2.41E+08 | 2.41E+08 | 401 | * | 3.69 | 1.18 | 4.56 | -2.98 | 2.39E-07 | 1.19E-05 | 4.924453 | -2.98 | down |
| chr1 | RGS7 | 2.41E+08 | 2.41E+08 | 401 | * | 4.93 | 1.81 | 5.85 | -3.78 | 4.11E-16 | 1.05E-13 | 12.97881 | -3.78 | down |
| chr1 | RGS7 | 2.41E+08 | 2.41E+08 | 401 | * | 4.11 | 1.51 | 4.98 | -3.16 | 1.46E-09 | 1.23E-07 | 6.910095 | -3.16 | down |
| chr1 | RGS7 | 2.41E+08 | 2.41E+08 | 401 | * | 4.9 | 2.54 | 5.75 | -3.06 | 4.15E-15 | 9.03E-13 | 12.04431 | -3.06 | down |
| chr1 | RGS7 | 2.41E+08 | 2.41E+08 | 401 | * | 5.69 | 2.63 | 6.6 | -3.84 | 1.50E-26 | 1.13E-23 | 22.94692 | -3.84 | down |
| chr1 | RYR2 | 2.37E+08 | 2.37E+08 | 401 | * | 6.77 | 6.02 | 7.27 | -1.15 | 1.37E-14 | 2.82E-12 | 11.54975 | -1.15 | down |
| chr1 | NID1 | 2.36E+08 | 2.36E+08 | 401 | * | 6.18 | 5.44 | 6.67 | -1.09 | 2.28E-10 | 2.29E-08 | 7.640165 | -1.09 | down |
| chr2 | SPP2 | 2.35E+08 | 2.35E+08 | 401 | * | 4.61 | 3.48 | 5.23 | -1.43 | 7.34E-07 | 3.19E-05 | 4.496209 | -1.43 | down |
| chr2 | SLC16A14 | 2.31E+08 | 2.31E+08 | 401 | * | 4.19 | 3.02 | 4.82 | -1.35 | 1.25E-05 | 0.00036 | 3.443697 | -1.35 | down |
| chr1 | RNF187 | 2.29E+08 | 2.29E+08 | 401 | * | 4.18 | 3.13 | 4.79 | -1.16 | 3.68E-05 | 0.000889 | 3.051098 | -1.16 | down |
| chr2 | RHBDD1 | 2.28E+08 | 2.28E+08 | 401 | * | 4.23 | 0 | 5.19 | -4.41 | 3.60E-09 | 2.81E-07 | 6.551294 | -4.41 | down |
| chr2 | AP1S3 | 2.25E+08 | 2.25E+08 | 401 | * | 5.35 | 4.44 | 5.9 | -1.23 | 3.21E-08 | 2.02E-06 | 5.694649 | -1.23 | down |
| chr1 | RAB3GAP2 | 2.2E+08 | 2.2E+08 | 401 | * | 6.5 | 5.77 | 6.99 | -1.11 | 3.39E-12 | 4.76E-10 | 9.322393 | -1.11 | down |
| chr2 | VWC2L | 2.15E+08 | 2.15E+08 | 401 | * | 6.99 | 6.14 | 7.53 | -1.32 | 1.77E-20 | 7.81E-18 | 17.10735 | -1.32 | down |
| chr1 | LINC00538 | 2.14E+08 | 2.14E+08 | 401 | * | 4.14 | 2.63 | 4.87 | -1.95 | 3.8E-07 | 1.79E-05 | 4.747147 | -1.95 | down |
| chr2 | MIR548F2 | 2.12E+08 | 2.12E+08 | 401 | * | 5.3 | 4.04 | 5.96 | -1.81 | 1.36E-11 | 1.73E-09 | 8.761954 | -1.81 | down |
| chr2 | MIR548F2 | 2.12E+08 | 2.12E+08 | 401 | * | 4.78 | 3.69 | 5.4 | -1.42 | 2.2E-07 | 1.11E-05 | 4.954677 | -1.42 | down |
| chr2 | MYL1 | 2.11E+08 | 2.11E+08 | 401 | * | 4.62 | 3.47 | 5.25 | -1.48 | 4.74E-07 | 2.17E-05 | 4.66354 | -1.48 | down |
| chr1 | MIR4260 | 2.1E+08 | 2.1E+08 | 401 | * | 6.1 | 2.39 | 7.04 | -4.51 | 8.38E-33 | 1.03E-29 | 28.98716 | -4.51 | down |
| chr1 | IL20 | 2.07E+08 | 2.07E+08 | 401 | * | 6.45 | 3.94 | 7.32 | -3.33 | 6.94E-42 | 1.40E-38 | 37.85387 | -3.33 | down |

| | | | | | | | | | | | | | | |
|---|---|---|---|---|---|---|---|---|---|---|---|---|---|---|
| chr1 | NFASC | 2.05E+08 | 2.05E+08 | 401 | * | 4.08 | 2.45 | 4.83 | -2.09 | 2.79E-07 | 1.36E-05 | 4.866461 | -2.09 | down |
| chr2 | FAM126B | 2.02E+08 | 2.02E+08 | 401 | * | 4.5 | 3.49 | 5.09 | -1.17 | 0.000014 | 0.000399 | 3.399027 | -1.17 | down |
| chr1 | NR5A2 | 2E+08 | 2E+08 | 401 | * | 4.34 | 3.16 | 4.98 | -1.4 | 4.7E-06 | 0.000159 | 3.798603 | -1.4 | down |
| chr1 | NR5A2 | 2E+08 | 2E+08 | 401 | * | 4.36 | 3.41 | 4.93 | -1.05 | 6.91E-05 | 0.0015 | 2.823909 | -1.05 | down |
| chr3 | LRCH3 | 1.98E+08 | 1.98E+08 | 401 | * | 4.62 | 3.69 | 5.17 | -1.1 | 1.31E-05 | 0.000375 | 3.425969 | -1.1 | down |
| chr3 | UBXN7 | 1.96E+08 | 1.96E+08 | 401 | * | 6.76 | 6.01 | 7.25 | -1.14 | 4.15E-14 | 7.90E-12 | 11.10237 | -1.14 | down |
| chr2 | SLC39A10 | 1.96E+08 | 1.96E+08 | 401 | * | 4.56 | 3.67 | 5.11 | -1.03 | 3.19E-05 | 0.000787 | 3.104025 | -1.03 | down |
| chr2 | PCGEM1 | 1.95E+08 | 1.95E+08 | 401 | * | 4.33 | 3.31 | 4.92 | -1.11 | 5.04E-05 | 0.00116 | 2.935542 | -1.11 | down |
| chr1 | B3GALT2 | 1.94E+08 | 1.94E+08 | 401 | * | 5.89 | 4.53 | 6.58 | -1.99 | 1.05E-17 | 3.34E-15 | 14.47625 | -1.99 | down |
| chr2 | PCGEM1 | 1.94E+08 | 1.94E+08 | 401 | * | 6.31 | 2.97 | 7.24 | -4.16 | 6.36E-39 | 1.08E-35 | 34.96658 | -4.16 | down |
| chr2 | PCGEM1 | 1.94E+08 | 1.94E+08 | 401 | * | 2.94 | 0.72 | 3.77 | -1.74 | 0.000185 | 0.00336 | 2.473661 | -1.74 | down |
| chr2 | PCGEM1 | 1.94E+08 | 1.94E+08 | 401 | * | 3.39 | 0 | 4.32 | -3.47 | 2.23E-06 | 8.29E-05 | 4.081445 | -3.47 | down |
| chr2 | TMEFF2 | 1.93E+08 | 1.93E+08 | 401 | * | 6.02 | 3.36 | 6.9 | -3.45 | 5.55E-32 | 6.14E-29 | 28.21183 | -3.45 | down |
| chr1 | UCHL5 | 1.93E+08 | 1.93E+08 | 401 | * | 5.06 | 4.05 | 5.65 | -1.34 | 7.12E-08 | 4.09E-06 | 5.388277 | -1.34 | down |
| chr3 | MB21D2 | 1.93E+08 | 1.93E+08 | 401 | * | 5.36 | 4.34 | 5.96 | -1.43 | 1.31E-09 | 1.12E-07 | 6.950782 | -1.43 | down |
| chr2 | NABP1 | 1.93E+08 | 1.93E+08 | 401 | * | 4.58 | 2.94 | 5.33 | -2.22 | 1.11E-09 | 9.68E-08 | 7.014125 | -2.22 | down |
| chr3 | OSTN | 1.91E+08 | 1.91E+08 | 401 | * | 4.08 | 2.42 | 4.83 | -2.17 | 2.47E-07 | 1.23E-05 | 4.910095 | -2.17 | down |
| chr3 | SNAR-I | 1.91E+08 | 1.91E+08 | 401 | * | 4.67 | 3.66 | 5.25 | -1.23 | 3.26E-06 | 0.000116 | 3.935542 | -1.23 | down |
| chr3 | GMNC | 1.91E+08 | 1.91E+08 | 401 | * | 4.58 | 3.42 | 5.22 | -1.5 | 4.44E-07 | 2.06E-05 | 4.686133 | -1.5 | down |
| chr3 | GMNC | 1.91E+08 | 1.91E+08 | 401 | * | 5.45 | 4.37 | 6.06 | -1.54 | 3.10E-10 | 3.03E-08 | 7.518557 | -1.54 | down |
| chr3 | GMNC | 1.91E+08 | 1.91E+08 | 401 | * | 4.34 | 2.89 | 5.05 | -1.88 | 1.53E-07 | 8.04E-06 | 5.094744 | -1.88 | down |
| chr1 | BRINP3 | 1.9E+08 | 1.9E+08 | 401 | * | 3.24 | 0.37 | 4.14 | -2.97 | 6.75E-06 | 0.000215 | 3.667562 | -2.97 | down |
| chr1 | BRINP3 | 1.9E+08 | 1.9E+08 | 401 | * | 3.46 | 1.16 | 4.3 | -2.63 | 2.8E-06 | 0.000102 | 3.9914 | -2.63 | down |
| chr3 | CLDN1 | 1.9E+08 | 1.9E+08 | 401 | * | 4.85 | 4 | 5.38 | -1.05 | 7.91E-06 | 0.000245 | 3.610834 | -1.05 | down |
| chr1 | BRINP3 | 1.9E+08 | 1.9E+08 | 401 | * | 3.69 | 1.21 | 4.55 | -2.98 | 1.85E-07 | 9.49E-06 | 5.022734 | -2.98 | down |
| chr1 | BRINP3 | 1.89E+08 | 1.89E+08 | 401 | * | 3.17 | 0.46 | 4.05 | -2.78 | 1.37E-05 | 0.00039 | 3.408935 | -2.78 | down |
| chr1 | BRINP3 | 1.89E+08 | 1.89E+08 | 401 | * | 4.21 | 2.78 | 4.91 | -1.83 | 5.23E-07 | 2.36E-05 | 4.627088 | -1.83 | down |
| chr1 | PLA2G4A | 1.88E+08 | 1.88E+08 | 401 | * | 4.84 | 2.68 | 5.67 | -2.85 | 6.18E-14 | 1.15E-11 | 10.9393 | -2.85 | down |

| | | | | | | | | | | | | | | |
|---|---|---|---|---|---|---|---|---|---|---|---|---|---|---|
| chr1 | PLA2G4A | 1.88E+08 | 1.88E+08 | 401 | * | 7.11 | 3.96 | 8.02 | -4.01 | 1.45E-67 | 1.37E-63 | 62.86328 | -4.01 | down |
| chr1 | PLA2G4A | 1.88E+08 | 1.88E+08 | 401 | * | 3.65 | 0.37 | 4.58 | -3.58 | 1.65E-07 | 8.57E-06 | 5.067019 | -3.58 | down |
| chr4 | LOC339975 | 1.88E+08 | 1.88E+08 | 401 | * | 3.47 | 1.13 | 4.32 | -2.63 | 2.98E-06 | 0.000107 | 3.970616 | -2.63 | down |
| chr4 | LOC339975 | 1.88E+08 | 1.88E+08 | 401 | * | 5.36 | 1.18 | 6.32 | -4.78 | 6.03E-19 | 2.21E-16 | 15.65561 | -4.78 | down |
| chr4 | FAT1 | 1.88E+08 | 1.88E+08 | 401 | * | 4.31 | 0.32 | 5.27 | -4.29 | 2.40E-10 | 2.4E-08 | 7.619789 | -4.29 | down |
| chr4 | FAT1 | 1.88E+08 | 1.88E+08 | 401 | * | 6.36 | 3.86 | 7.23 | -3.3 | 1.37E-39 | 2.40E-36 | 35.61979 | -3.3 | down |
| chr4 | FAT1 | 1.88E+08 | 1.88E+08 | 401 | * | 3.44 | 0.37 | 4.35 | -3.31 | 1.09E-06 | 4.51E-05 | 4.345823 | -3.31 | down |
| chr3 | LPP | 1.88E+08 | 1.88E+08 | 401 | * | 5.34 | 3.95 | 6.04 | -1.98 | 4.14E-13 | 6.83E-11 | 10.16558 | -1.98 | down |
| chr4 | F11-AS1 | 1.87E+08 | 1.87E+08 | 401 | * | 5.05 | 1.41 | 5.99 | -4.3 | 6.36E-17 | 1.81E-14 | 13.74232 | -4.3 | down |
| chr3 | RTP4 | 1.87E+08 | 1.87E+08 | 401 | * | 6.42 | 5.32 | 7.03 | -1.64 | 3.44E-19 | 1.29E-16 | 15.88941 | -1.64 | down |
| chr4 | SORBS2 | 1.87E+08 | 1.87E+08 | 401 | * | 4.68 | 3.74 | 5.24 | -1.13 | 8.26E-06 | 0.000254 | 3.595166 | -1.13 | down |
| chr4 | SORBS2 | 1.87E+08 | 1.87E+08 | 401 | * | 7.43 | 6.38 | 8.03 | -1.61 | 9.92E-35 | 1.34E-31 | 30.8729 | -1.61 | down |
| chr4 | SORBS2 | 1.87E+08 | 1.87E+08 | 401 | * | 4.72 | 3.7 | 5.32 | -1.29 | 1.01E-06 | 4.21E-05 | 4.375718 | -1.29 | down |
| chr4 | SORBS2 | 1.87E+08 | 1.87E+08 | 401 | * | 3.94 | 2.89 | 4.54 | -1.02 | 0.000197 | 0.00353 | 2.452225 | -1.02 | down |
| chr4 | SORBS2 | 1.87E+08 | 1.87E+08 | 401 | * | 4.65 | 3.58 | 5.26 | -1.33 | 1.25E-06 | 5.05E-05 | 4.296709 | -1.33 | down |
| chr3 | ST6GAL1 | 1.87E+08 | 1.87E+08 | 401 | * | 5.09 | 3.8 | 5.76 | -1.83 | 3.29E-10 | 3.19E-08 | 7.496209 | -1.83 | down |
| chr4 | SORBS2 | 1.87E+08 | 1.87E+08 | 401 | * | 3.78 | 2.65 | 4.4 | -1.08 | 0.000197 | 0.00354 | 2.450997 | -1.08 | down |
| chr4 | SORBS2 | 1.87E+08 | 1.87E+08 | 401 | * | 5.81 | 4.37 | 6.51 | -2.07 | 2.30E-17 | 6.91E-15 | 14.16052 | -2.07 | down |
| chr4 | SORBS2 | 1.87E+08 | 1.87E+08 | 401 | * | 5.19 | 3.57 | 5.93 | -2.3 | 1.04E-13 | 1.86E-11 | 10.73049 | -2.3 | down |
| chr4 | SORBS2 | 1.87E+08 | 1.87E+08 | 401 | * | 4.33 | 2.48 | 5.12 | -2.48 | 2.32E-09 | 1.88E-07 | 6.725842 | -2.48 | down |
| chr4 | SORBS2 | 1.87E+08 | 1.87E+08 | 401 | * | 4.27 | 2.2 | 5.09 | -2.7 | 2.21E-09 | 1.81E-07 | 6.742321 | -2.7 | down |
| chr4 | SORBS2 | 1.87E+08 | 1.87E+08 | 401 | * | 3.02 | 0.97 | 3.83 | -1.8 | 0.000113 | 0.00227 | 2.643974 | -1.8 | down |
| chr2 | ZNF804A | 1.86E+08 | 1.86E+08 | 401 | * | 6.56 | 4.31 | 7.4 | -3.05 | 2.24E-43 | 5.12E-40 | 39.29073 | -3.05 | down |
| chr2 | ZNF804A | 1.86E+08 | 1.86E+08 | 401 | * | 3.08 | 0.94 | 3.9 | -1.94 | 7.79E-05 | 0.00166 | 2.779892 | -1.94 | down |
| chr2 | ZNF804A | 1.86E+08 | 1.86E+08 | 401 | * | 4.1 | 1.97 | 4.92 | -2.73 | 7.39E-09 | 5.35E-07 | 6.271646 | -2.73 | down |
| chr4 | LINC02363 | 1.85E+08 | 1.85E+08 | 401 | * | 4.22 | 3.19 | 4.82 | -1.13 | 4.16E-05 | 0.000984 | 3.007005 | -1.13 | down |
| chr2 | ZNF804A | 1.85E+08 | 1.85E+08 | 401 | * | 7.16 | 5.6 | 7.9 | -2.28 | 9.33E-48 | 3.26E-44 | 43.48678 | -2.28 | down |
| chr2 | ZNF804A | 1.85E+08 | 1.85E+08 | 401 | * | 3.78 | 1.57 | 4.61 | -2.73 | 2.25E-07 | 1.13E-05 | 4.946922 | -2.73 | down |

| | | | | | | | | | | | | | | |
|---|---|---|---|---|---|---|---|---|---|---|---|---|---|---|
| chr2 | ZNF804A | 1.85E+08 | 1.85E+08 | 401 | * | 4 | 2.46 | 4.73 | -1.93 | 1.14E-06 | 4.69E-05 | 4.328827 | -1.93 | down |
| chr2 | ZNF804A | 1.85E+08 | 1.85E+08 | 401 | * | 4.95 | 2.83 | 5.78 | -2.83 | 9.94E-15 | 2.10E-12 | 11.67778 | -2.83 | down |
| chr4 | CDKN2AIP | 1.84E+08 | 1.84E+08 | 401 | * | 5.28 | 3.19 | 6.1 | -2.82 | 4.74E-18 | 1.57E-15 | 14.8041 | -2.82 | down |
| chr3 | DVL3 | 1.84E+08 | 1.84E+08 | 401 | * | 6.2 | 5.47 | 6.68 | -1.08 | 3.86E-10 | 3.67E-08 | 7.435334 | -1.08 | down |
| chr3 | KLHL6 | 1.83E+08 | 1.83E+08 | 401 | * | 4.33 | 3.29 | 4.93 | -1.19 | 2.18E-05 | 0.000575 | 3.240332 | -1.19 | down |
| chr3 | LINC00888 | 1.83E+08 | 1.83E+08 | 401 | * | 5.8 | 4.98 | 6.32 | -1.17 | 1.55E-09 | 1.3E-07 | 6.886057 | -1.17 | down |
| chr1 | RGS8 | 1.83E+08 | 1.83E+08 | 401 | * | 4.78 | 3.93 | 5.31 | -1.02 | 2.41E-05 | 0.000626 | 3.203426 | -1.02 | down |
| chr2 | ITGA4 | 1.82E+08 | 1.82E+08 | 401 | * | 3.92 | 2.84 | 4.54 | -1.04 | 0.000188 | 0.00341 | 2.467246 | -1.04 | down |
| chr2 | UBE2E3 | 1.82E+08 | 1.82E+08 | 401 | * | 5.29 | 4.45 | 5.81 | -1.12 | 2.68E-07 | 1.32E-05 | 4.879426 | -1.12 | down |
| chr5 | OR4F16 | 1.81E+08 | 1.81E+08 | 401 | * | 6.78 | 5.94 | 7.31 | -1.28 | 1.48E-17 | 4.63E-15 | 14.33442 | -1.28 | down |
| chr5 | OR4F16 | 1.81E+08 | 1.81E+08 | 401 | * | 4.75 | 3.89 | 5.28 | -1.02 | 1.96E-05 | 0.000528 | 3.277366 | -1.02 | down |
| chr5 | RUFY1 | 1.79E+08 | 1.79E+08 | 401 | * | 5.56 | 4.57 | 6.15 | -1.38 | 1.15E-09 | 9.94E-08 | 7.002614 | -1.38 | down |
| chr2 | HNRNPA3 | 1.78E+08 | 1.78E+08 | 401 | * | 5.13 | 4.31 | 5.65 | -1.07 | 1.58E-06 | 6.19E-05 | 4.208309 | -1.07 | down |
| chr3 | TBL1XR1 | 1.76E+08 | 1.76E+08 | 401 | * | 4.8 | 3.89 | 5.36 | -1.12 | 4.34E-06 | 0.000148 | 3.829738 | -1.12 | down |
| chr5 | SIMC1 | 1.76E+08 | 1.76E+08 | 401 | * | 4.39 | 3.26 | 5.01 | -1.31 | 7.18E-06 | 0.000226 | 3.645892 | -1.31 | down |
| chr5 | SIMC1 | 1.76E+08 | 1.76E+08 | 401 | * | 3.78 | 2.56 | 4.43 | -1.21 | 0.000102 | 0.00209 | 2.679854 | -1.21 | down |
| chr3 | MIR4789 | 1.75E+08 | 1.75E+08 | 401 | * | 5.98 | 3.82 | 6.81 | -2.95 | 9.39E-29 | 7.99E-26 | 25.09745 | -2.95 | down |
| chr3 | MIR4789 | 1.75E+08 | 1.75E+08 | 401 | * | 4.24 | 1.64 | 5.11 | -3.17 | 2.25E-10 | 2.27E-08 | 7.643974 | -3.17 | down |
| chr3 | NAALADL2 | 1.74E+08 | 1.74E+08 | 401 | * | 5.2 | 4.25 | 5.77 | -1.27 | 7.1E-08 | 4.08E-06 | 5.38934 | -1.27 | down |
| chr3 | NAALADL2 | 1.74E+08 | 1.74E+08 | 401 | * | 4.03 | 2.16 | 4.82 | -2.39 | 9.62E-08 | 5.33E-06 | 5.273273 | -2.39 | down |
| chr2 | MAP3K20 | 1.74E+08 | 1.74E+08 | 401 | * | 3.91 | 2.6 | 4.58 | -1.36 | 4.46E-05 | 0.00104 | 2.982967 | -1.36 | down |
| chr3 | NLGN1 | 1.74E+08 | 1.74E+08 | 401 | * | 3.53 | 1.23 | 4.38 | -2.74 | 1.34E-06 | 5.35E-05 | 4.271646 | -2.74 | down |
| chr3 | NLGN1 | 1.74E+08 | 1.74E+08 | 401 | * | 4.54 | 0.68 | 5.49 | -4.29 | 5.44E-12 | 7.28E-10 | 9.137869 | -4.29 | down |
| chr3 | NLGN1 | 1.74E+08 | 1.74E+08 | 401 | * | 5.86 | 3.02 | 6.76 | -3.63 | 8.60E-30 | 8.04E-27 | 26.09474 | -3.63 | down |
| chr5 | NSG2 | 1.74E+08 | 1.74E+08 | 401 | * | 4.34 | 3.39 | 4.91 | -1.07 | 4.69E-05 | 0.00109 | 2.962574 | -1.07 | down |
| chr3 | NLGN1 | 1.74E+08 | 1.74E+08 | 401 | * | 3.79 | 2.18 | 4.53 | -1.86 | 8.18E-06 | 0.000251 | 3.600326 | -1.86 | down |
| chr5 | NSG2 | 1.73E+08 | 1.73E+08 | 401 | * | 4.71 | 3.84 | 5.25 | -1.02 | 3.02E-05 | 0.000753 | 3.123205 | -1.02 | down |
| chr5 | STC2 | 1.73E+08 | 1.73E+08 | 401 | * | 6.43 | 5.66 | 6.93 | -1.15 | 2.55E-12 | 3.69E-10 | 9.432974 | -1.15 | down |

| | | | | | | | | | | | | | | |
|---|---|---|---|---|---|---|---|---|---|---|---|---|---|---|
| chr2 | NA | 1.72E+08 | 1.72E+08 | 401 | * | 6.57 | 5.37 | 7.22 | -1.8 | 1.41E-23 | 8.69E-21 | 20.06098 | -1.8 | down |
| chr3 | CLDN11 | 1.7E+08 | 1.7E+08 | 401 | * | 4.82 | 3.97 | 5.35 | -1.01 | 2.11E-05 | 0.000559 | 3.252588 | -1.01 | down |
| chr6 | WDR27 | 1.7E+08 | 1.7E+08 | 401 | * | 4.68 | 3.69 | 5.26 | -1.18 | 6.11E-06 | 0.000198 | 3.703335 | -1.18 | down |
| chr6 | THBS2 | 1.7E+08 | 1.7E+08 | 401 | * | 7.51 | 6.88 | 7.96 | -1.02 | 6.64E-18 | 2.16E-15 | 14.66555 | -1.02 | down |
| chr2 | STK39 | 1.69E+08 | 1.69E+08 | 401 | * | 4.15 | 3.05 | 4.76 | -1.14 | 6.35E-05 | 0.0014 | 2.853872 | -1.14 | down |
| chr6 | LINC01558 | 1.68E+08 | 1.68E+08 | 401 | * | 7.75 | 6.85 | 8.3 | -1.4 | 1.46E-32 | 1.76E-29 | 28.75449 | -1.4 | down |
| chr6 | TCP10 | 1.68E+08 | 1.68E+08 | 401 | * | 3.65 | 2.36 | 4.33 | -1.22 | 0.000134 | 0.00259 | 2.5867 | -1.22 | down |
| chr1 | MPC2 | 1.68E+08 | 1.68E+08 | 401 | * | 5.4 | 4.57 | 5.92 | -1.12 | 1.2E-07 | 6.45E-06 | 5.19044 | -1.12 | down |
| chr6 | TCP10 | 1.68E+08 | 1.68E+08 | 401 | * | 5.48 | 3.92 | 6.21 | -2.24 | 6.40E-16 | 1.58E-13 | 12.80134 | -2.24 | down |
| chr6 | TCP10 | 1.68E+08 | 1.68E+08 | 401 | * | 3.4 | 0.41 | 4.3 | -3.24 | 1.64E-06 | 6.41E-05 | 4.193142 | -3.24 | down |
| chr1 | MPZL1 | 1.68E+08 | 1.68E+08 | 401 | * | 5.13 | 3.97 | 5.76 | -1.61 | 1.78E-09 | 1.48E-07 | 6.829738 | -1.61 | down |
| chr6 | LINC00473 | 1.66E+08 | 1.66E+08 | 401 | * | 4.79 | 3.9 | 5.33 | -1.09 | 0.000011 | 0.000324 | 3.489455 | -1.09 | down |
| chr2 | CSRNP3 | 1.66E+08 | 1.66E+08 | 401 | * | 3.75 | 2.55 | 4.39 | -1.16 | 0.00014 | 0.00268 | 2.571865 | -1.16 | down |
| chr2 | GRB14 | 1.65E+08 | 1.65E+08 | 401 | * | 4.21 | 1.55 | 5.09 | -3.27 | 2.10E-10 | 2.12E-08 | 7.673664 | -3.27 | down |
| chr2 | GRB14 | 1.65E+08 | 1.65E+08 | 401 | * | 5.21 | 4.2 | 5.8 | -1.35 | 4.25E-08 | 2.56E-06 | 5.59176 | -1.35 | down |
| chr4 | ANP32C | 1.65E+08 | 1.65E+08 | 401 | * | 4.71 | 3.83 | 5.26 | -1.04 | 0.000021 | 0.000558 | 3.253366 | -1.04 | down |
| chr4 | FSTL5 | 1.62E+08 | 1.62E+08 | 401 | * | 6.02 | 4.6 | 6.72 | -2.06 | 1.84E-20 | 8.08E-18 | 17.09259 | -2.06 | down |
| chr5 | GABRG2 | 1.62E+08 | 1.62E+08 | 401 | * | 7.19 | 6.07 | 7.81 | -1.7 | 1.53E-29 | 1.41E-26 | 25.85078 | -1.7 | down |
| chr5 | GABRG2 | 1.62E+08 | 1.62E+08 | 401 | * | 4.31 | 3.38 | 4.87 | -1.01 | 8.59E-05 | 0.0018 | 2.744727 | -1.01 | down |
| chr4 | RAPGEF2 | 1.61E+08 | 1.61E+08 | 401 | * | 2.84 | 0.03 | 3.73 | -2.16 | 0.000114 | 0.00228 | 2.642065 | -2.16 | down |
| chr4 | RAPGEF2 | 1.61E+08 | 1.61E+08 | 401 | * | 3.16 | 1.41 | 3.93 | -1.5 | 0.000182 | 0.00332 | 2.478862 | -1.5 | down |
| chr2 | MIR4785 | 1.61E+08 | 1.61E+08 | 401 | * | 5.2 | 2.3 | 6.09 | -3.64 | 2.20E-19 | 8.39E-17 | 16.07624 | -3.64 | down |
| chr3 | SPTSSB | 1.61E+08 | 1.61E+08 | 401 | * | 4.75 | 3.39 | 5.44 | -1.88 | 4.23E-09 | 3.26E-07 | 6.486782 | -1.88 | down |
| chr3 | IQCJ-SCHIP1 | 1.59E+08 | 1.59E+08 | 401 | * | 4.14 | 2.93 | 4.79 | -1.32 | 2.26E-05 | 0.000593 | 3.226945 | -1.32 | down |
| chr3 | MFSD1 | 1.59E+08 | 1.59E+08 | 401 | * | 4 | 2.97 | 4.59 | -1.03 | 0.000157 | 0.00295 | 2.530178 | -1.03 | down |
| chr6 | SNX9 | 1.58E+08 | 1.58E+08 | 401 | * | 6.49 | 5.66 | 7.01 | -1.24 | 2.80E-14 | 5.47E-12 | 11.26201 | -1.24 | down |
| chr6 | SNX9 | 1.58E+08 | 1.58E+08 | 401 | * | 5.38 | 3.96 | 6.08 | -2.03 | 7.95E-14 | 1.45E-11 | 10.83863 | -2.03 | down |
| chr3 | MLF1 | 1.58E+08 | 1.58E+08 | 401 | * | 4.37 | 1.95 | 5.23 | -3.05 | 4.70E-11 | 5.36E-09 | 8.270835 | -3.05 | down |

| | | | | | | | | | | | | | | |
|---|---|---|---|---|---|---|---|---|---|---|---|---|---|---|
| chr3 | MLF1 | 1.58E+08 | 1.58E+08 | 401 | * | 3.16 | 1.37 | 3.93 | -1.57 | 0.000145 | 0.00276 | 2.559091 | -1.57 | down |
| chr3 | LINC00881 | 1.57E+08 | 1.57E+08 | 401 | * | 6.3 | 5.62 | 6.76 | -1.01 | 1.32E-09 | 1.12E-07 | 6.950782 | -1.01 | down |
| chr7 | LMBR1 | 1.57E+08 | 1.57E+08 | 401 | * | 5.69 | 4.93 | 6.18 | -1.05 | 6.2E-08 | 3.61E-06 | 5.442493 | -1.05 | down |
| chr1 | SMG5 | 1.56E+08 | 1.56E+08 | 401 | * | 5.54 | 4.34 | 6.19 | -1.75 | 8.95E-13 | 1.40E-10 | 9.853872 | -1.75 | down |
| chr2 | KCNJ3 | 1.56E+08 | 1.56E+08 | 401 | * | 3.58 | 0.97 | 4.46 | -3.04 | 4.6E-07 | 2.12E-05 | 4.673664 | -3.04 | down |
| chr4 | SFRP2 | 1.55E+08 | 1.55E+08 | 401 | * | 3.91 | 2.11 | 4.68 | -2.29 | 4.22E-07 | 1.96E-05 | 4.707744 | -2.29 | down |
| chr4 | TLR2 | 1.55E+08 | 1.55E+08 | 401 | * | 4.97 | 4.07 | 5.52 | -1.15 | 1.78E-06 | 6.84E-05 | 4.164944 | -1.15 | down |
| chr4 | TMEM131L | 1.54E+08 | 1.54E+08 | 401 | * | 5.55 | 4.63 | 6.1 | -1.28 | 4.58E-09 | 3.51E-07 | 6.454693 | -1.28 | down |
| chr3 | ARHGEF26 | 1.54E+08 | 1.54E+08 | 401 | * | 3.93 | 1.81 | 4.75 | -2.64 | 6.91E-08 | 3.99E-06 | 5.399027 | -2.64 | down |
| chr6 | RGS17 | 1.53E+08 | 1.53E+08 | 401 | * | 5.09 | 3.96 | 5.72 | -1.54 | 5.01E-09 | 3.77E-07 | 6.423659 | -1.54 | down |
| chr6 | RGS17 | 1.53E+08 | 1.53E+08 | 401 | * | 4.62 | 3.58 | 5.22 | -1.26 | 3.02E-06 | 0.000108 | 3.966576 | -1.26 | down |
| chr4 | FBXW7 | 1.53E+08 | 1.53E+08 | 401 | * | 4.82 | 3.95 | 5.36 | -1.06 | 8.92E-06 | 0.000271 | 3.567031 | -1.06 | down |
| chr7 | GALNT11 | 1.52E+08 | 1.52E+08 | 401 | * | 5.67 | 4.86 | 6.18 | -1.12 | 1.84E-08 | 1.22E-06 | 5.91364 | -1.12 | down |
| chr6 | AKAP12 | 1.51E+08 | 1.51E+08 | 401 | * | 4.93 | 4.07 | 5.46 | -1.05 | 9.31E-06 | 0.000281 | 3.551294 | -1.05 | down |
| chr1 | TNFAIP8L2 | 1.51E+08 | 1.51E+08 | 401 | * | 4.83 | 2.39 | 5.69 | -3.15 | 9.29E-15 | 1.97E-12 | 11.70553 | -3.15 | down |
| chr6 | PPP1R14C | 1.5E+08 | 1.5E+08 | 401 | * | 5.09 | 3.91 | 5.73 | -1.64 | 2.49E-09 | 2.01E-07 | 6.696804 | -1.64 | down |
| chr7 | ATP6V0E2-AS1 | 1.5E+08 | 1.5E+08 | 401 | * | 6.07 | 5.34 | 6.55 | -1.05 | 3.23E-09 | 2.56E-07 | 6.59176 | -1.05 | down |
| chr7 | ATP6V0E2-AS1 | 1.5E+08 | 1.5E+08 | 401 | * | 3.92 | 2.67 | 4.57 | -1.31 | 4.15E-05 | 0.000983 | 3.007446 | -1.31 | down |
| chr3 | PFN2 | 1.5E+08 | 1.5E+08 | 401 | * | 6.51 | 5.43 | 7.12 | -1.62 | 7.75E-20 | 3.14E-17 | 16.50307 | -1.62 | down |
| chr5 | CAMK2A | 1.5E+08 | 1.5E+08 | 401 | * | 4.26 | 3.23 | 4.85 | -1.13 | 3.79E-05 | 0.00091 | 3.040959 | -1.13 | down |
| chr7 | KRBA1 | 1.49E+08 | 1.49E+08 | 401 | * | 4.52 | 2.05 | 5.39 | -3.11 | 6.44E-12 | 8.54E-10 | 9.068542 | -3.11 | down |
| chrX | IDS | 1.49E+08 | 1.49E+08 | 401 | * | 3.28 | 0.37 | 4.18 | -3.02 | 5.39E-06 | 0.000178 | 3.74958 | -3.02 | down |
| chr6 | SASH1 | 1.48E+08 | 1.48E+08 | 401 | * | 4.49 | 3.61 | 5.03 | -1.01 | 0.000055 | 0.00125 | 2.90309 | -1.01 | down |
| chr1 | NBPF14 | 1.48E+08 | 1.48E+08 | 401 | * | 3.69 | 0 | 4.64 | -3.86 | 2.23E-07 | 1.12E-05 | 4.950782 | -3.86 | down |
| chr1 | NBPF9 | 1.48E+08 | 1.48E+08 | 401 | * | 4.04 | 0 | 5.01 | -4.43 | 1.31E-07 | 7.02E-06 | 5.153663 | -4.43 | down |
| chr1 | NBPF14 | 1.48E+08 | 1.48E+08 | 401 | * | 2.76 | 0 | 3.68 | -2.05 | 0.000171 | 0.00315 | 2.501689 | -2.05 | down |
| chr4 | TTC29 | 1.48E+08 | 1.48E+08 | 401 | * | 4.8 | 3.72 | 5.42 | -1.43 | 2.45E-07 | 1.22E-05 | 4.91364 | -1.43 | down |
| chr6 | ADGB | 1.47E+08 | 1.47E+08 | 401 | * | 4.72 | 3.49 | 5.38 | -1.62 | 5.8E-08 | 3.39E-06 | 5.4698 | -1.62 | down |

| | | | | | | | | | | | | | | |
|---|---|---|---|---|---|---|---|---|---|---|---|---|---|---|
| chr6 | ADGB | 1.47E+08 | 1.47E+08 | 401 | * | 6.02 | 4.79 | 6.67 | -1.8 | 5.53E-17 | 1.59E-14 | 13.7986 | -1.8 | down |
| chr7 | MIR548AR | 1.47E+08 | 1.47E+08 | 401 | * | 5.45 | 1.7 | 6.39 | -4.44 | 2.24E-21 | 1.16E-18 | 17.93554 | -4.44 | down |
| chr7 | MIR548AR | 1.47E+08 | 1.47E+08 | 401 | * | 3.32 | 0.68 | 4.2 | -2.85 | 4.44E-06 | 0.000151 | 3.821023 | -2.85 | down |
| chr6 | EPM2A | 1.46E+08 | 1.46E+08 | 401 | * | 6 | 5.28 | 6.48 | -1.04 | 6.23E-09 | 4.6E-07 | 6.337242 | -1.04 | down |
| chr1 | HJV | 1.45E+08 | 1.45E+08 | 401 | * | 6.2 | 5.42 | 6.71 | -1.15 | 2.26E-11 | 2.76E-09 | 8.559091 | -1.15 | down |
| chr1 | NOTCH2NLA | 1.45E+08 | 1.45E+08 | 401 | * | 4.65 | 3.74 | 5.21 | -1.1 | 1.08E-05 | 0.000318 | 3.497573 | -1.1 | down |
| chr2 | GTDC1 | 1.45E+08 | 1.45E+08 | 401 | * | 6.16 | 5.44 | 6.65 | -1.06 | 6.38E-10 | 5.86E-08 | 7.232102 | -1.06 | down |
| chr2 | ARHGAP15 | 1.44E+08 | 1.44E+08 | 401 | * | 5.41 | 4.58 | 5.93 | -1.11 | 1.61E-07 | 8.39E-06 | 5.076238 | -1.11 | down |
| chr1 | NA | 1.44E+08 | 1.44E+08 | 401 | * | 3.39 | 1.88 | 4.11 | -1.41 | 0.00014 | 0.00268 | 2.571865 | -1.41 | down |
| chr5 | NR3C1 | 1.43E+08 | 1.43E+08 | 401 | * | 4.12 | 2.89 | 4.77 | -1.38 | 1.49E-05 | 0.000419 | 3.377786 | -1.38 | down |
| chr5 | NR3C1 | 1.43E+08 | 1.43E+08 | 401 | * | 6.43 | 5.45 | 7.01 | -1.46 | 2.28E-16 | 6.04E-14 | 13.21896 | -1.46 | down |
| chr8 | LINC01300 | 1.42E+08 | 1.42E+08 | 401 | * | 4.37 | 3.09 | 5.04 | -1.63 | 8.33E-07 | 3.56E-05 | 4.44855 | -1.63 | down |
| chr3 | ATR | 1.42E+08 | 1.42E+08 | 401 | * | 5.76 | 4.79 | 6.34 | -1.39 | 5.03E-11 | 5.68E-09 | 8.245652 | -1.39 | down |
| chr3 | TFDP2 | 1.42E+08 | 1.42E+08 | 401 | * | 5.82 | 5.06 | 6.31 | -1.05 | 1.62E-07 | 8.43E-06 | 5.074172 | -1.05 | down |
| chr6 | MIR4465 | 1.41E+08 | 1.41E+08 | 401 | * | 4.37 | 3.14 | 5.02 | -1.54 | 1.35E-06 | 5.39E-05 | 4.268411 | -1.54 | down |
| chr6 | MIR4465 | 1.41E+08 | 1.41E+08 | 401 | * | 5.55 | 4.59 | 6.12 | -1.35 | 1.93E-09 | 1.59E-07 | 6.798603 | -1.35 | down |
| chr5 | DELE1 | 1.41E+08 | 1.41E+08 | 401 | * | 4.15 | 2.89 | 4.81 | -1.49 | 9.5E-06 | 0.000286 | 3.543634 | -1.49 | down |
| chr6 | MIR4465 | 1.41E+08 | 1.41E+08 | 401 | * | 5.25 | 4.26 | 5.84 | -1.35 | 1.47E-08 | 9.98E-07 | 6.000869 | -1.35 | down |
| chr6 | MIR4465 | 1.41E+08 | 1.41E+08 | 401 | * | 6.16 | 2.77 | 7.09 | -4.19 | 1.14E-34 | 1.51E-31 | 30.82102 | -4.19 | down |
| chr8 | TRAPPC9 | 1.41E+08 | 1.41E+08 | 401 | * | 6.5 | 5.8 | 6.97 | -1.05 | 2.62E-11 | 3.16E-09 | 8.500313 | -1.05 | down |
| chr6 | MIR4465 | 1.41E+08 | 1.41E+08 | 401 | * | 4.44 | 1.83 | 5.32 | -3.26 | 6.86E-12 | 9.05E-10 | 9.043351 | -3.26 | down |
| chr6 | MIR3668 | 1.41E+08 | 1.41E+08 | 401 | * | 4.42 | 2.97 | 5.13 | -1.94 | 5.1E-08 | 3.04E-06 | 5.517126 | -1.94 | down |
| chr6 | MIR3668 | 1.4E+08 | 1.4E+08 | 401 | * | 5.75 | 4.29 | 6.46 | -2.12 | 1.47E-17 | 4.63E-15 | 14.33442 | -2.12 | down |
| chr8 | COL22A1 | 1.4E+08 | 1.4E+08 | 401 | * | 4.82 | 3.98 | 5.35 | -1.02 | 0.000019 | 0.000513 | 3.289883 | -1.02 | down |
| chr6 | HECA | 1.4E+08 | 1.4E+08 | 401 | * | 4.5 | 3.54 | 5.07 | -1.12 | 1.79E-05 | 0.000489 | 3.310691 | -1.12 | down |
| chr8 | FAM135B | 1.39E+08 | 1.39E+08 | 401 | * | 5.53 | 4.75 | 6.03 | -1.07 | 2.05E-07 | 1.04E-05 | 4.982967 | -1.07 | down |
| chr5 | NRG2 | 1.39E+08 | 1.39E+08 | 401 | * | 4.68 | 3.27 | 5.38 | -1.9 | 7.38E-09 | 5.35E-07 | 6.271646 | -1.9 | down |
| chr5 | PSD2 | 1.39E+08 | 1.39E+08 | 401 | * | 5.25 | 4.21 | 5.85 | -1.43 | 4.34E-09 | 3.34E-07 | 6.476254 | -1.43 | down |

| | | | | | | | | | | | | | | |
|---|---|---|---|---|---|---|---|---|---|---|---|---|---|---|
| chr5 | LRRTM2 | 1.38E+08 | 1.38E+08 | 401 | * | 5.69 | 4.87 | 6.2 | -1.12 | 1.96E-08 | 1.28E-06 | 5.89279 | -1.12 | down |
| chr7 | TRIM24 | 1.38E+08 | 1.38E+08 | 401 | * | 5.71 | 4.52 | 6.35 | -1.72 | 7.05E-14 | 1.30E-11 | 10.88606 | -1.72 | down |
| chrX | ZIC3 | 1.37E+08 | 1.37E+08 | 401 | * | 4.24 | 2.76 | 4.96 | -1.92 | 2.1E-07 | 1.06E-05 | 4.974694 | -1.92 | down |
| chr7 | PTN | 1.37E+08 | 1.37E+08 | 401 | * | 5.88 | 5.15 | 6.36 | -1.03 | 2.53E-08 | 1.62E-06 | 5.790485 | -1.03 | down |
| chr9 | VAV2 | 1.37E+08 | 1.37E+08 | 401 | * | 6.15 | 5.43 | 6.63 | -1.04 | 2.75E-09 | 2.21E-07 | 6.655608 | -1.04 | down |
| chr4 | PABPC4L | 1.36E+08 | 1.36E+08 | 401 | * | 2.99 | 0 | 3.93 | -2.71 | 5.12E-05 | 0.00117 | 2.931814 | -2.71 | down |
| chr4 | PABPC4L | 1.36E+08 | 1.36E+08 | 401 | * | 3.6 | 0.97 | 4.48 | -3.06 | 3.45E-07 | 1.64E-05 | 4.785156 | -3.06 | down |
| chr7 | LUZP6 | 1.36E+08 | 1.36E+08 | 401 | * | 5.99 | 4.84 | 6.62 | -1.68 | 1.04E-15 | 2.48E-13 | 12.60555 | -1.68 | down |
| chr7 | LUZP6 | 1.36E+08 | 1.36E+08 | 401 | * | 5.45 | 4.51 | 6.02 | -1.06 | 4.13E-05 | 0.000979 | 3.009217 | -1.06 | down |
| chr4 | PABPC4L | 1.36E+08 | 1.36E+08 | 401 | * | 3.9 | 0 | 4.87 | -4.29 | 2.86E-07 | 1.39E-05 | 4.856985 | -4.29 | down |
| chr4 | PABPC4L | 1.36E+08 | 1.36E+08 | 401 | * | 4.4 | 1.21 | 5.32 | -3.78 | 9.50E-12 | 1.24E-09 | 8.906578 | -3.78 | down |
| chr4 | PABPC4L | 1.36E+08 | 1.36E+08 | 401 | * | 3.38 | 0 | 4.34 | -3.56 | 6.41E-06 | 0.000206 | 3.686133 | -3.56 | down |
| chr4 | PABPC4L | 1.36E+08 | 1.36E+08 | 401 | * | 3.5 | 0 | 4.48 | -4.3 | 1.22E-06 | 4.96E-05 | 4.304518 | -4.3 | down |
| chr4 | PABPC4L | 1.36E+08 | 1.36E+08 | 401 | * | 3.91 | 0.41 | 4.84 | -3.85 | 2.03E-08 | 1.32E-06 | 5.879426 | -3.85 | down |
| chr4 | PABPC4L | 1.35E+08 | 1.35E+08 | 401 | * | 6.96 | 2.1 | 7.94 | -5.67 | 3.06E-45 | 7.82E-42 | 41.10679 | -5.67 | down |
| chr5 | VTRNA2-1 | 1.35E+08 | 1.35E+08 | 401 | * | 5.02 | 4.21 | 5.53 | -1.02 | 6.94E-06 | 0.00022 | 3.657577 | -1.02 | down |
| chr5 | SLC25A48-AS1 | 1.35E+08 | 1.35E+08 | 401 | * | 5.57 | 3.03 | 6.44 | -3.31 | 6.05E-24 | 3.86E-21 | 20.41341 | -3.31 | down |
| chr8 | ST3GAL1 | 1.35E+08 | 1.35E+08 | 401 | * | 4.21 | 3.23 | 4.79 | -1.05 | 8.14E-05 | 0.00172 | 2.764472 | -1.05 | down |
| chr4 | PABPC4L | 1.35E+08 | 1.35E+08 | 401 | * | 4.48 | 0 | 5.47 | -5.37 | 7.20E-09 | 5.26E-07 | 6.279014 | -5.37 | down |
| chr7 | CALD1 | 1.34E+08 | 1.34E+08 | 401 | * | 7.27 | 5.35 | 8.07 | -2.7 | 4.97E-62 | 3.66E-58 | 57.43652 | -2.7 | down |
| chr7 | CALD1 | 1.34E+08 | 1.34E+08 | 401 | * | 7.37 | 5.95 | 8.07 | -2.1 | 7.62E-49 | 3.16E-45 | 44.50031 | -2.1 | down |
| chr6 | LINC01312 | 1.34E+08 | 1.34E+08 | 401 | * | 5.73 | 4.95 | 6.23 | -1.09 | 2.42E-08 | 1.55E-06 | 5.809668 | -1.09 | down |
| chr4 | PCDH10 | 1.34E+08 | 1.34E+08 | 401 | * | 4.11 | 1.97 | 4.93 | -2.74 | 5.66E-09 | 4.22E-07 | 6.374688 | -2.74 | down |
| chr4 | PCDH10 | 1.34E+08 | 1.34E+08 | 401 | * | 4.31 | 0.91 | 5.24 | -3.88 | 4.73E-11 | 5.38E-09 | 8.269218 | -3.88 | down |
| chr4 | PCDH10 | 1.33E+08 | 1.33E+08 | 401 | * | 3.08 | 0.72 | 3.94 | -2.32 | 3.53E-05 | 0.000857 | 3.067019 | -2.32 | down |
| chr4 | PCDH10 | 1.33E+08 | 1.33E+08 | 401 | * | 5.1 | 3.58 | 5.83 | -2.14 | 2.02E-12 | 2.97E-10 | 9.527244 | -2.14 | down |
| chr4 | PCDH10 | 1.33E+08 | 1.33E+08 | 401 | * | 4.44 | 3.24 | 5.09 | -1.51 | 9.43E-07 | 3.95E-05 | 4.403403 | -1.51 | down |
| chr11 | OPCML | 1.33E+08 | 1.33E+08 | 401 | * | 7.92 | 7.18 | 8.41 | -1.19 | 5.39E-28 | 4.48E-25 | 24.34872 | -1.19 | down |

| | | | | | | | | | | | | | | |
|---|---|---|---|---|---|---|---|---|---|---|---|---|---|---|
| chr11 | OPCML | 1.33E+08 | 1.33E+08 | 401 | * | 3.24 | 1.43 | 4.02 | -1.77 | 0.000072 | 0.00156 | 2.806875 | -1.77 | down |
| chr9 | LINC00963 | 1.32E+08 | 1.32E+08 | 401 | * | 6.29 | 5.33 | 6.86 | -1.43 | 1.74E-15 | 4.00E-13 | 12.39794 | -1.43 | down |
| chr10 | GLRX3 | 1.32E+08 | 1.32E+08 | 401 | * | 3.66 | 2.37 | 4.33 | -1.19 | 0.000169 | 0.00313 | 2.504456 | -1.19 | down |
| chr3 | ACPP | 1.32E+08 | 1.32E+08 | 401 | * | 5.28 | 4.25 | 5.87 | -1.41 | 4.66E-09 | 3.56E-07 | 6.44855 | -1.41 | down |
| chr8 | ADCY8 | 1.32E+08 | 1.32E+08 | 401 | * | 3.96 | 2.89 | 4.56 | -1.06 | 0.000143 | 0.00273 | 2.563837 | -1.06 | down |
| chr10 | MGMT | 1.31E+08 | 1.31E+08 | 401 | * | 3.55 | 1.59 | 4.36 | -2.3 | 0.0000005 | 0.000167 | 3.777284 | -2.3 | down |
| chr9 | ODF2 | 1.31E+08 | 1.31E+08 | 401 | * | 5.15 | 3.67 | 5.86 | -2.1 | 1.44E-12 | 2.17E-10 | 9.66354 | -2.1 | down |
| chr2 | PTPN18 | 1.31E+08 | 1.31E+08 | 401 | * | 4.96 | 3.29 | 5.71 | -2.33 | 4.16E-12 | 5.71E-10 | 9.243364 | -2.33 | down |
| chr9 | MIR219B | 1.31E+08 | 1.31E+08 | 401 | * | 5.88 | 4.96 | 6.44 | -1.33 | 1.93E-11 | 2.41E-09 | 8.617983 | -1.33 | down |
| chr4 | C4orf33 | 1.3E+08 | 1.3E+08 | 401 | * | 3.55 | 1.21 | 4.4 | -2.78 | 9.81E-07 | 4.08E-05 | 4.38934 | -2.78 | down |
| chr12 | TMEM132D | 1.3E+08 | 1.3E+08 | 401 | * | 3.71 | 2.47 | 4.36 | -1.14 | 0.000187 | 0.00339 | 2.4698 | -1.14 | down |
| chr3 | COL6A5 | 1.3E+08 | 1.3E+08 | 401 | * | 5.12 | 2.95 | 5.95 | -2.89 | 9.30E-17 | 2.58E-14 | 13.58838 | -2.89 | down |
| chr8 | MIR1208 | 1.3E+08 | 1.3E+08 | 401 | * | 5.24 | 3.99 | 5.91 | -1.79 | 1.11E-10 | 1.19E-08 | 7.924453 | -1.79 | down |
| chr8 | MIR1208 | 1.3E+08 | 1.3E+08 | 401 | * | 4.35 | 3.22 | 4.98 | -1.33 | 6.18E-06 | 0.0002 | 3.69897 | -1.33 | down |
| chr3 | TMCC1 | 1.29E+08 | 1.29E+08 | 401 | * | 7.09 | 6.28 | 7.6 | -1.25 | 7.69E-19 | 2.78E-16 | 15.55596 | -1.25 | down |
| chr5 | CHSY3 | 1.29E+08 | 1.29E+08 | 401 | * | 3.52 | 2.3 | 4.18 | -1.04 | 0.000374 | 0.00589 | 2.229885 | -1.04 | down |
| chr7 | MIR183 | 1.29E+08 | 1.29E+08 | 401 | * | 5.22 | 3.72 | 5.94 | -2.1 | 5.14E-12 | 6.94E-10 | 9.158641 | -2.1 | down |
| chr3 | H1-8 | 1.29E+08 | 1.29E+08 | 401 | * | 6.48 | 3.27 | 7.39 | -4.03 | 2.13E-44 | 5.24E-41 | 40.28067 | -4.03 | down |
| chr11 | ARHGAP32 | 1.29E+08 | 1.29E+08 | 401 | * | 4.12 | 2.66 | 4.84 | -1.75 | 2.95E-06 | 0.000106 | 3.974694 | -1.75 | down |
| chr11 | KIRREL3 | 1.27E+08 | 1.27E+08 | 401 | * | 4.86 | 2.55 | 5.71 | -3.02 | 1.13E-14 | 2.35E-12 | 11.62893 | -3.02 | down |
| chr12 | LINC00943 | 1.27E+08 | 1.27E+08 | 401 | * | 5.02 | 3.62 | 5.72 | -1.97 | 4.34E-11 | 5.01E-09 | 8.300162 | -1.97 | down |
| chr10 | LHPP | 1.26E+08 | 1.26E+08 | 401 | * | 6.43 | 5.64 | 6.93 | -1.16 | 4.11E-12 | 5.66E-10 | 9.247184 | -1.16 | down |
| chr7 | MIR592 | 1.26E+08 | 1.26E+08 | 401 | * | 4.86 | 2.9 | 5.66 | -2.64 | 3.02E-13 | 5.03E-11 | 10.29843 | -2.64 | down |
| chrX | PRR32 | 1.26E+08 | 1.26E+08 | 401 | * | 3.65 | 1.83 | 4.43 | -2.15 | 3.86E-06 | 0.000133 | 3.876148 | -2.15 | down |
| chr3 | OSBPL11 | 1.25E+08 | 1.25E+08 | 401 | * | 4.25 | 3.06 | 4.89 | -1.4 | 5.84E-06 | 0.000191 | 3.718967 | -1.4 | down |
| chr12 | NCOR2 | 1.25E+08 | 1.25E+08 | 401 | * | 7.57 | 6.79 | 8.07 | -1.21 | 1.59E-24 | 1.06E-21 | 20.97469 | -1.21 | down |
| chr8 | FER1L6-AS1 | 1.25E+08 | 1.25E+08 | 401 | * | 3.73 | 2.48 | 4.38 | -1.22 | 0.000115 | 0.00229 | 2.640165 | -1.22 | down |
| chr12 | RFLNA | 1.25E+08 | 1.25E+08 | 401 | * | 4.93 | 3.65 | 5.6 | -1.74 | 3.86E-09 | 2.99E-07 | 6.524329 | -1.74 | down |

| | | | | | | | | | | | | | | |
|---|---|---|---|---|---|---|---|---|---|---|---|---|---|---|
| chr11 | ROBO3 | 1.25E+08 | 1.25E+08 | 401 | * | 4.34 | 3.27 | 4.94 | -1.2 | 1.77E-05 | 0.000485 | 3.314258 | -1.2 | down |
| chr12 | ZNF664 | 1.25E+08 | 1.25E+08 | 401 | * | 4.64 | 3.5 | 5.27 | -1.46 | 4.78E-07 | 2.19E-05 | 4.659556 | -1.46 | down |
| chr8 | WDYHV1 | 1.24E+08 | 1.24E+08 | 401 | * | 4.18 | 3.11 | 4.79 | -1.16 | 4.05E-05 | 0.000964 | 3.015923 | -1.16 | down |
| chr9 | GGTA1P | 1.24E+08 | 1.24E+08 | 401 | * | 3.84 | 2.76 | 4.45 | -1.02 | 0.00023 | 0.00401 | 2.396856 | -1.02 | down |
| chr3 | MIR5002 | 1.24E+08 | 1.24E+08 | 401 | * | 4.45 | 3.49 | 5.03 | -1.11 | 2.68E-05 | 0.000683 | 3.165579 | -1.11 | down |
| chr2 | CNTNAP5 | 1.24E+08 | 1.24E+08 | 401 | * | 4.58 | 3.2 | 5.27 | -1.87 | 2.15E-08 | 1.4E-06 | 5.853872 | -1.87 | down |
| chr2 | CNTNAP5 | 1.24E+08 | 1.24E+08 | 401 | * | 4 | 2.72 | 4.66 | -1.47 | 1.26E-05 | 0.000363 | 3.440093 | -1.47 | down |
| chr2 | CNTNAP5 | 1.24E+08 | 1.24E+08 | 401 | * | 6.3 | 4.41 | 7.09 | -2.65 | 1.78E-32 | 2.11E-29 | 28.67572 | -2.65 | down |
| chr12 | PITPNM2-AS1 | 1.24E+08 | 1.24E+08 | 401 | * | 4.63 | 3.17 | 5.35 | -1.98 | 4.91E-09 | 3.71E-07 | 6.430626 | -1.98 | down |
| chr4 | IL21 | 1.24E+08 | 1.24E+08 | 401 | * | 5.34 | 4.42 | 5.89 | -1.23 | 7.59E-08 | 4.33E-06 | 5.363512 | -1.23 | down |
| chr2 | TSN | 1.23E+08 | 1.23E+08 | 401 | * | 4.03 | 2.92 | 4.65 | -1.18 | 5.72E-05 | 0.00128 | 2.89279 | -1.18 | down |
| chr12 | KNTC1 | 1.23E+08 | 1.23E+08 | 401 | * | 6.27 | 5.53 | 6.75 | -1.09 | 8.81E-11 | 9.68E-09 | 8.014125 | -1.09 | down |
| chrX | THOC2 | 1.23E+08 | 1.23E+08 | 401 | * | 4.24 | 2.58 | 4.99 | -2.22 | 5.17E-08 | 3.07E-06 | 5.512862 | -2.22 | down |
| chr3 | HSPBAP1 | 1.23E+08 | 1.23E+08 | 401 | * | 6.04 | 4.5 | 6.77 | -2.24 | 1.36E-22 | 7.66E-20 | 19.11577 | -2.24 | down |
| chr9 | BRINP1 | 1.22E+08 | 1.22E+08 | 401 | * | 4.3 | 2.77 | 5.02 | -2 | 1.05E-07 | 5.77E-06 | 5.238824 | -2 | down |
| chrX | GRIA3 | 1.22E+08 | 1.22E+08 | 401 | * | 3.8 | 1.8 | 4.61 | -2.45 | 4.8E-07 | 0.000022 | 4.657577 | -2.45 | down |
| chr2 | CLASP1 | 1.22E+08 | 1.22E+08 | 401 | * | 5.07 | 4.09 | 5.66 | -1.32 | 7.36E-08 | 4.22E-06 | 5.374688 | -1.32 | down |
| chr12 | KDM2B | 1.22E+08 | 1.22E+08 | 401 | * | 5.84 | 4.55 | 6.51 | -1.89 | 8.16E-17 | 2.29E-14 | 13.64016 | -1.89 | down |
| chrX | GRIA3 | 1.22E+08 | 1.22E+08 | 401 | * | 3.95 | 0 | 4.91 | -4.12 | 4.07E-08 | 2.48E-06 | 5.605548 | -4.12 | down |
| chr12 | P2RX4 | 1.22E+08 | 1.22E+08 | 401 | * | 5.84 | 5.14 | 6.32 | -1.01 | 4.43E-08 | 2.66E-06 | 5.575118 | -1.01 | down |
| chr12 | SPPL3 | 1.21E+08 | 1.21E+08 | 401 | * | 5.4 | 3.14 | 6.25 | -3.02 | 1.46E-20 | 6.57E-18 | 17.18243 | -3.02 | down |
| chr11 | ARHGEF12 | 1.2E+08 | 1.2E+08 | 401 | * | 5.49 | 4.74 | 5.98 | -1.02 | 4.59E-07 | 2.12E-05 | 4.673664 | -1.02 | down |
| chr12 | CIT | 1.2E+08 | 1.2E+08 | 401 | * | 5.35 | 4.56 | 5.86 | -1.06 | 6.15E-07 | 2.72E-05 | 4.565431 | -1.06 | down |
| chr12 | SRRM4 | 1.19E+08 | 1.19E+08 | 401 | * | 5.14 | 4.3 | 5.66 | -1.09 | 1.68E-06 | 6.54E-05 | 4.184422 | -1.09 | down |
| chr4 | NDST3 | 1.19E+08 | 1.19E+08 | 401 | * | 3.89 | 2.7 | 4.53 | -1.22 | 7.99E-05 | 0.0017 | 2.769551 | -1.22 | down |
| chr11 | TMPRSS4 | 1.18E+08 | 1.18E+08 | 401 | * | 4.62 | 2.88 | 5.39 | -2.36 | 2.02E-10 | 2.06E-08 | 7.686133 | -2.36 | down |
| chr10 | ATRNL1 | 1.17E+08 | 1.17E+08 | 401 | * | 5.09 | 3.49 | 5.83 | -2.23 | 7.13E-13 | 1.13E-10 | 9.946922 | -2.23 | down |
| chr11 | SIK3 | 1.17E+08 | 1.17E+08 | 401 | * | 5.22 | 4.36 | 5.76 | -1.14 | 3.07E-07 | 1.48E-05 | 4.829738 | -1.14 | down |

| | | | | | | | | | | | | | | |
|---|---|---|---|---|---|---|---|---|---|---|---|---|---|---|
| chr2 | DPP10 | 1.17E+08 | 1.17E+08 | 401 | * | 4.92 | 3.18 | 5.69 | -2.4 | 1.71E-12 | 2.54E-10 | 9.595166 | -2.4 | down |
| chr2 | DPP10 | 1.17E+08 | 1.17E+08 | 401 | * | 5.62 | 4.07 | 6.35 | -2.21 | 1.78E-17 | 5.47E-15 | 14.26201 | -2.21 | down |
| chr2 | DPP10 | 1.17E+08 | 1.17E+08 | 401 | * | 3.66 | 2.17 | 4.37 | -1.59 | 2.84E-05 | 0.000717 | 3.144481 | -1.59 | down |
| chr10 | FAM160B1 | 1.17E+08 | 1.17E+08 | 401 | * | 5.65 | 3.83 | 6.44 | -2.54 | 9.01E-21 | 4.15E-18 | 17.38195 | -2.54 | down |
| chr8 | TRPS1 | 1.17E+08 | 1.17E+08 | 401 | * | 4.82 | 3.56 | 5.48 | -1.71 | 1.71E-08 | 1.14E-06 | 5.943095 | -1.71 | down |
| chr4 | NDST4 | 1.16E+08 | 1.16E+08 | 401 | * | 5.56 | 4.82 | 6.04 | -1.01 | 3.4E-07 | 1.62E-05 | 4.790485 | -1.01 | down |
| chr11 | LINC00900 | 1.16E+08 | 1.16E+08 | 401 | * | 6.21 | 5.47 | 6.7 | -1.08 | 2.10E-10 | 2.12E-08 | 7.673664 | -1.08 | down |
| chr8 | CSMD3 | 1.15E+08 | 1.15E+08 | 401 | * | 4.24 | 2.92 | 4.92 | -1.66 | 1.32E-06 | 5.32E-05 | 4.274088 | -1.66 | down |
| chr7 | MDFIC | 1.15E+08 | 1.15E+08 | 401 | * | 5.47 | 2.79 | 6.35 | -3.46 | 1.74E-22 | 9.55E-20 | 19.02 | -3.46 | down |
| chr12 | TBX5 | 1.15E+08 | 1.15E+08 | 401 | * | 4.16 | 2.36 | 4.94 | -2.35 | 3.33E-08 | 2.07E-06 | 5.68403 | -2.35 | down |
| chr8 | CSMD3 | 1.15E+08 | 1.15E+08 | 401 | * | 3.91 | 1.34 | 4.78 | -3.08 | 1.7E-08 | 1.13E-06 | 5.946922 | -3.08 | down |
| chr8 | CSMD3 | 1.14E+08 | 1.14E+08 | 401 | * | 4.08 | 2.08 | 4.88 | -2.58 | 1.94E-08 | 1.27E-06 | 5.896196 | -2.58 | down |
| chrX | MIR448 | 1.14E+08 | 1.14E+08 | 401 | * | 5.66 | 4.8 | 6.2 | -1.21 | 3.10E-09 | 2.47E-07 | 6.607303 | -1.21 | down |
| chr3 | NAA50 | 1.13E+08 | 1.13E+08 | 401 | * | 4.13 | 1.18 | 5.03 | -3.49 | 6.69E-10 | 6.09E-08 | 7.215383 | -3.49 | down |
| chr8 | CSMD3 | 1.13E+08 | 1.13E+08 | 401 | * | 4.7 | 3.51 | 5.35 | -1.57 | 1.39E-07 | 7.39E-06 | 5.131356 | -1.57 | down |
| chr8 | CSMD3 | 1.13E+08 | 1.13E+08 | 401 | * | 4.1 | 2.17 | 4.89 | -2.49 | 2.74E-08 | 1.74E-06 | 5.759451 | -2.49 | down |
| chr12 | RPH3A | 1.13E+08 | 1.13E+08 | 401 | * | 6.1 | 5.41 | 6.57 | -1.01 | 4.50E-09 | 3.45E-07 | 6.462181 | -1.01 | down |
| chr13 | SPACA7 | 1.13E+08 | 1.13E+08 | 401 | * | 5.06 | 4.06 | 5.65 | -1.33 | 1.18E-07 | 6.37E-06 | 5.195861 | -1.33 | down |
| chr10 | ADRA2A | 1.13E+08 | 1.13E+08 | 401 | * | 5.1 | 3.72 | 5.79 | -1.92 | 1.75E-10 | 1.82E-08 | 7.739929 | -1.92 | down |
| chr4 | FAM241A | 1.13E+08 | 1.13E+08 | 401 | * | 7.37 | 6.28 | 7.99 | -1.68 | 6.64E-36 | 9.79E-33 | 32.00922 | -1.68 | down |
| chr8 | CSMD3 | 1.13E+08 | 1.13E+08 | 401 | * | 2.99 | 0.37 | 3.87 | -2.38 | 4.75E-05 | 0.0011 | 2.958607 | -2.38 | down |
| chr4 | FAM241A | 1.13E+08 | 1.13E+08 | 401 | * | 3.28 | 1.49 | 4.05 | -1.63 | 9.55E-05 | 0.00197 | 2.705534 | -1.63 | down |
| chr3 | BOC | 1.13E+08 | 1.13E+08 | 401 | * | 4.94 | 3.94 | 5.53 | -1.3 | 3.94E-07 | 1.85E-05 | 4.732828 | -1.3 | down |
| chrX | AMOT | 1.13E+08 | 1.13E+08 | 401 | * | 5.85 | 4.97 | 6.39 | -1.26 | 1.87E-10 | 1.92E-08 | 7.716699 | -1.26 | down |
| chr3 | NEPRO | 1.13E+08 | 1.13E+08 | 401 | * | 5.47 | 4.72 | 5.97 | -1.03 | 5E-07 | 2.28E-05 | 4.642065 | -1.03 | down |
| chr5 | DCP2 | 1.12E+08 | 1.12E+08 | 401 | * | 3.62 | 2.36 | 4.28 | -1.12 | 0.00024 | 0.00413 | 2.38405 | -1.12 | down |
| chr12 | ALDH2 | 1.12E+08 | 1.12E+08 | 401 | * | 5.29 | 4.48 | 5.8 | -1.07 | 8.01E-07 | 3.44E-05 | 4.463442 | -1.07 | down |
| chr11 | SDHD | 1.12E+08 | 1.12E+08 | 401 | * | 5.34 | 4.23 | 5.96 | -1.57 | 3.61E-10 | 3.47E-08 | 7.459671 | -1.57 | down |

| | | | | | | | | | | | | | | |
|---|---|---|---|---|---|---|---|---|---|---|---|---|---|---|
| chr8 | KCNV1 | 1.11E+08 | 1.11E+08 | 401 | * | 4.09 | 1.57 | 4.96 | -3.13 | 1.54E-09 | 1.29E-07 | 6.88941 | -3.13 | down |
| chr8 | KCNV1 | 1.11E+08 | 1.11E+08 | 401 | * | 4.23 | 1.18 | 5.14 | -3.61 | 1.10E-10 | 1.19E-08 | 7.924453 | -3.61 | down |
| chr8 | KCNV1 | 1.11E+08 | 1.11E+08 | 401 | * | 2.89 | 0 | 3.83 | -2.43 | 8.78E-05 | 0.00184 | 2.735182 | -2.43 | down |
| chr2 | LIMS3-LOC440895 | 1.11E+08 | 1.11E+08 | 401 | * | 6.02 | 5.19 | 6.55 | -1.2 | 1.11E-10 | 1.19E-08 | 7.924453 | -1.2 | down |
| chr8 | KCNV1 | 1.11E+08 | 1.11E+08 | 401 | * | 3.66 | 0.32 | 4.59 | -3.59 | 1.58E-07 | 8.25E-06 | 5.083546 | -3.59 | down |
| chr8 | KCNV1 | 1.11E+08 | 1.11E+08 | 401 | * | 4.25 | 0 | 5.24 | -5.15 | 2.43E-08 | 1.56E-06 | 5.806875 | -5.15 | down |
| chr6 | AMD1 | 1.11E+08 | 1.11E+08 | 401 | * | 3.96 | 2.6 | 4.64 | -1.52 | 1.36E-05 | 0.000388 | 3.411168 | -1.52 | down |
| chr8 | KCNV1 | 1.11E+08 | 1.11E+08 | 401 | * | 3.75 | 1.57 | 4.58 | -2.69 | 3.11E-07 | 0.000015 | 4.823909 | -2.69 | down |
| chr5 | NREP | 1.11E+08 | 1.11E+08 | 401 | * | 5 | 3.97 | 5.6 | -1.38 | 6.92E-08 | 3.99E-06 | 5.399027 | -1.38 | down |
| chr8 | KCNV1 | 1.11E+08 | 1.11E+08 | 401 | * | 3.67 | 0.64 | 4.58 | -3.38 | 1.28E-07 | 6.85E-06 | 5.164309 | -3.38 | down |
| chr8 | KCNV1 | 1.11E+08 | 1.11E+08 | 401 | * | 3.01 | 0.93 | 3.83 | -1.74 | 0.00014 | 0.00268 | 2.571865 | -1.74 | down |
| chr8 | KCNV1 | 1.11E+08 | 1.11E+08 | 401 | * | 3.86 | 0.41 | 4.79 | -3.81 | 2.6E-08 | 1.66E-06 | 5.779892 | -3.81 | down |
| chr8 | KCNV1 | 1.11E+08 | 1.11E+08 | 401 | * | 6.75 | 2.19 | 7.72 | -5.35 | 3.14E-42 | 6.72E-39 | 38.17263 | -5.35 | down |
| chr8 | KCNV1 | 1.11E+08 | 1.11E+08 | 401 | * | 3.71 | 1.18 | 4.58 | -3 | 2.26E-07 | 1.13E-05 | 4.946922 | -3 | down |
| chr8 | KCNV1 | 1.11E+08 | 1.11E+08 | 401 | * | 3.73 | 1 | 4.62 | -3.23 | 7.68E-08 | 4.38E-06 | 5.358526 | -3.23 | down |
| chr8 | KCNV1 | 1.11E+08 | 1.11E+08 | 401 | * | 5.2 | 0.41 | 6.17 | -5.17 | 1.01E-14 | 2.12E-12 | 11.67366 | -5.17 | down |
| chr8 | SYBU | 1.11E+08 | 1.11E+08 | 401 | * | 3.78 | 2.37 | 4.48 | -1.5 | 2.94E-05 | 0.000739 | 3.131356 | -1.5 | down |
| chr13 | IRS2 | 1.11E+08 | 1.11E+08 | 401 | * | 5.6 | 4.8 | 6.12 | -1.11 | 4.23E-08 | 2.56E-06 | 5.59176 | -1.11 | down |
| chr13 | IRS2 | 1.1E+08 | 1.1E+08 | 401 | * | 5.22 | 4.33 | 5.77 | -1.18 | 1.72E-07 | 8.91E-06 | 5.050122 | -1.18 | down |
| chr8 | TRHR | 1.1E+08 | 1.1E+08 | 401 | * | 4.66 | 2.91 | 5.43 | -2.4 | 6.37E-11 | 7.12E-09 | 8.14752 | -2.4 | down |
| chr8 | TRHR | 1.1E+08 | 1.1E+08 | 401 | * | 4.28 | 2.96 | 4.95 | -1.66 | 9.04E-07 | 3.81E-05 | 4.419075 | -1.66 | down |
| chr12 | FOXN4 | 1.1E+08 | 1.1E+08 | 401 | * | 5.29 | 3.55 | 6.05 | -2.42 | 1.91E-15 | 4.38E-13 | 12.35853 | -2.42 | down |
| chr1 | KIAA1324 | 1.1E+08 | 1.1E+08 | 401 | * | 5.8 | 4.68 | 6.42 | -1.64 | 7.85E-14 | 1.44E-11 | 10.84164 | -1.64 | down |
| chr8 | EMC2 | 1.1E+08 | 1.1E+08 | 401 | * | 4.33 | 3.32 | 4.91 | -1.12 | 3.21E-05 | 0.000791 | 3.101824 | -1.12 | down |
| chr1 | AKNAD1 | 1.09E+08 | 1.09E+08 | 401 | * | 4.63 | 3.49 | 5.26 | -1.44 | 4.96E-07 | 2.26E-05 | 4.645892 | -1.44 | down |
| chr1 | AKNAD1 | 1.09E+08 | 1.09E+08 | 401 | * | 4.71 | 3.57 | 5.34 | -1.45 | 3.02E-07 | 1.46E-05 | 4.835647 | -1.45 | down |
| chr2 | LIMS1 | 1.09E+08 | 1.09E+08 | 401 | * | 6.07 | 5.28 | 6.57 | -1.14 | 4.15E-10 | 3.92E-08 | 7.406714 | -1.14 | down |
| chr8 | RSPO2 | 1.09E+08 | 1.09E+08 | 401 | * | 8.18 | 7.29 | 8.72 | -1.4 | 2.97E-45 | 7.82E-42 | 41.10679 | -1.4 | down |

| | | | | | | | | | | | | | | |
|---|---|---|---|---|---|---|---|---|---|---|---|---|---|---|
| chr8 | RSPO2 | 1.09E+08 | 1.09E+08 | 401 | * | 7.4 | 6.59 | 7.91 | -1.26 | 1.05E-23 | 6.57E-21 | 20.18243 | -1.26 | down |
| chr2 | SLC5A7 | 1.09E+08 | 1.09E+08 | 401 | * | 6.46 | 5.5 | 7.03 | -1.45 | 5.36E-17 | 1.55E-14 | 13.80967 | -1.45 | down |
| chr11 | DDX10 | 1.09E+08 | 1.09E+08 | 401 | * | 3.76 | 2.66 | 4.38 | -1.02 | 0.000288 | 0.00478 | 2.320572 | -1.02 | down |
| chr5 | FER | 1.08E+08 | 1.08E+08 | 401 | * | 5.31 | 4.39 | 5.87 | -1.26 | 3.27E-08 | 2.04E-06 | 5.69037 | -1.26 | down |
| chr13 | LINC00443 | 1.08E+08 | 1.08E+08 | 401 | * | 3.24 | 0 | 4.22 | -3.92 | 4.42E-06 | 0.00015 | 3.823909 | -3.92 | down |
| chrX | COL4A5 | 1.08E+08 | 1.08E+08 | 401 | * | 5.77 | 5.02 | 6.26 | -1.03 | 8.56E-08 | 4.8E-06 | 5.318759 | -1.03 | down |
| chrX | PRPS1 | 1.07E+08 | 1.07E+08 | 401 | * | 5.29 | 4.06 | 5.94 | -1.74 | 4.57E-11 | 5.25E-09 | 8.279841 | -1.74 | down |
| chr7 | NAMPT | 1.06E+08 | 1.06E+08 | 401 | * | 6.24 | 5.56 | 6.7 | -1.01 | 2.01E-09 | 1.66E-07 | 6.779892 | -1.01 | down |
| chr7 | NAMPT | 1.06E+08 | 1.06E+08 | 401 | * | 5.45 | 4.51 | 6.02 | -1.28 | 7.46E-09 | 5.39E-07 | 6.268411 | -1.28 | down |
| chr14 | GPR132 | 1.06E+08 | 1.06E+08 | 401 | * | 6.4 | 5.58 | 6.92 | -1.22 | 1.83E-13 | 3.14E-11 | 10.50307 | -1.22 | down |
| chr14 | GPR132 | 1.06E+08 | 1.06E+08 | 401 | * | 6.65 | 5.92 | 7.14 | -1.1 | 1.13E-12 | 1.74E-10 | 9.759451 | -1.1 | down |
| chr14 | GPR132 | 1.06E+08 | 1.06E+08 | 401 | * | 4.69 | 3.72 | 5.27 | -1.19 | 4.99E-06 | 0.000167 | 3.777284 | -1.19 | down |
| chr14 | AHNAK2 | 1.05E+08 | 1.05E+08 | 401 | * | 7.35 | 6.6 | 7.84 | -1.18 | 1.91E-20 | 8.27E-18 | 17.08249 | -1.18 | down |
| chr1 | LOC100129138 | 1.05E+08 | 1.05E+08 | 401 | * | 4.36 | 1.98 | 5.22 | -3.04 | 7.24E-11 | 8.02E-09 | 8.095826 | -3.04 | down |
| chr5 | RAB9BP1 | 1.05E+08 | 1.05E+08 | 401 | * | 2.85 | 0.78 | 3.67 | -1.36 | 0.000466 | 0.00703 | 2.153045 | -1.36 | down |
| chr1 | LOC100129138 | 1.05E+08 | 1.05E+08 | 401 | * | 4.22 | 2.58 | 4.97 | -2.17 | 8.06E-08 | 4.55E-06 | 5.341989 | -2.17 | down |
| chr6 | HACE1 | 1.05E+08 | 1.05E+08 | 401 | * | 4.07 | 2.61 | 4.79 | -1.79 | 1.93E-06 | 7.33E-05 | 4.134896 | -1.79 | down |
| chr14 | C14orf180 | 1.05E+08 | 1.05E+08 | 401 | * | 4.44 | 3.51 | 5 | -1.05 | 0.000045 | 0.00105 | 2.978811 | -1.05 | down |
| chr13 | MIR548AS | 1.05E+08 | 1.05E+08 | 401 | * | 5.54 | 4.26 | 6.2 | -1.85 | 4.07E-13 | 6.72E-11 | 10.17263 | -1.85 | down |
| chr11 | CASP12 | 1.04E+08 | 1.04E+08 | 401 | * | 6.43 | 5.73 | 6.91 | -1.06 | 4.16E-11 | 4.81E-09 | 8.317855 | -1.06 | down |
| chrX | IL1RAPL2 | 1.04E+08 | 1.04E+08 | 401 | * | 4.93 | 4.07 | 5.47 | -1.08 | 4.24E-06 | 0.000145 | 3.838632 | -1.08 | down |
| chr3 | MIR548A3 | 1.04E+08 | 1.04E+08 | 401 | * | 5.39 | 1.55 | 6.34 | -4.51 | 1.89E-20 | 8.23E-18 | 17.0846 | -4.51 | down |
| chr3 | MIR548A3 | 1.04E+08 | 1.04E+08 | 401 | * | 4.02 | 1.92 | 4.84 | -2.64 | 2.77E-08 | 1.76E-06 | 5.754487 | -2.64 | down |
| chr3 | MIR548A3 | 1.04E+08 | 1.04E+08 | 401 | * | 3.31 | 0.68 | 4.19 | -2.83 | 4.97E-06 | 0.000166 | 3.779892 | -2.83 | down |
| chr12 | STAB2 | 1.04E+08 | 1.04E+08 | 401 | * | 6.87 | 6.18 | 7.34 | -1.07 | 7.37E-14 | 1.36E-11 | 10.86646 | -1.07 | down |
| chrX | IL1RAPL2 | 1.04E+08 | 1.04E+08 | 401 | * | 5.32 | 4.37 | 5.89 | -1.3 | 1.49E-08 | 1.01E-06 | 5.995679 | -1.3 | down |
| chr7 | RELN | 1.04E+08 | 1.04E+08 | 401 | * | 3.89 | 2.75 | 4.52 | -1.09 | 0.000157 | 0.00295 | 2.530178 | -1.09 | down |
| chr9 | PLPPR1 | 1.04E+08 | 1.04E+08 | 401 | * | 4.28 | 2.47 | 5.05 | -2.38 | 1.91E-08 | 1.26E-06 | 5.899629 | -2.38 | down |

| | | | | | | | | | | | | | | |
|---|---|---|---|---|---|---|---|---|---|---|---|---|---|---|
| chr9 | PLPPR1 | 1.04E+08 | 1.04E+08 | 401 | * | 4.27 | 2.87 | 4.96 | -1.79 | 7.45E-07 | 3.22E-05 | 4.492144 | -1.79 | down |
| chr10 | POLL | 1.03E+08 | 1.03E+08 | 401 | * | 5.46 | 4.46 | 6.05 | -1.41 | 5.96E-10 | 5.5E-08 | 7.259637 | -1.41 | down |
| chr9 | TEX10 | 1.03E+08 | 1.03E+08 | 401 | * | 4.71 | 1.93 | 5.6 | -3.44 | 4.84E-14 | 9.16E-12 | 11.0381 | -3.44 | down |
| chr6 | GRIK2 | 1.03E+08 | 1.03E+08 | 401 | * | 3.06 | 0.91 | 3.89 | -1.88 | 9.91E-05 | 0.00203 | 2.692504 | -1.88 | down |
| chr10 | KAZALD1 | 1.03E+08 | 1.03E+08 | 401 | * | 6.14 | 5.07 | 6.74 | -1.6 | 5.24E-16 | 1.31E-13 | 12.88273 | -1.6 | down |
| chr6 | GRIK2 | 1.02E+08 | 1.02E+08 | 401 | * | 4.53 | 2.48 | 5.34 | -2.72 | 3.95E-11 | 4.61E-09 | 8.336299 | -2.72 | down |
| chr2 | MAP4K4 | 1.02E+08 | 1.02E+08 | 401 | * | 5.09 | 4.1 | 5.67 | -1.3 | 1.12E-07 | 6.1E-06 | 5.21467 | -1.3 | down |
| chr2 | RFX8 | 1.02E+08 | 1.02E+08 | 401 | * | 4.66 | 3.01 | 5.41 | -2.25 | 2.69E-10 | 2.67E-08 | 7.573489 | -2.25 | down |
| chr8 | YWHAZ | 1.02E+08 | 1.02E+08 | 401 | * | 5.92 | 5.04 | 6.46 | -1.28 | 5.40E-11 | 6.08E-09 | 8.216096 | -1.28 | down |
| chr7 | SH2B2 | 1.02E+08 | 1.02E+08 | 401 | * | 5.81 | 3.33 | 6.67 | -3.26 | 3.46E-27 | 2.77E-24 | 23.55752 | -3.26 | down |
| chrX | NXF2 | 1.02E+08 | 1.02E+08 | 401 | * | 3.88 | 1.85 | 4.69 | -2.56 | 1.64E-07 | 8.52E-06 | 5.06956 | -2.56 | down |
| chr9 | GABBR2 | 1.01E+08 | 1.01E+08 | 401 | * | 7.35 | 6.65 | 7.82 | -1.11 | 2.86E-19 | 1.08E-16 | 15.96658 | -1.11 | down |
| chrX | TCP11X2 | 1.01E+08 | 1.01E+08 | 401 | * | 4.07 | 2.92 | 4.7 | -1.28 | 2.69E-05 | 0.000684 | 3.164944 | -1.28 | down |
| chrX | BEX5 | 1.01E+08 | 1.01E+08 | 401 | * | 3.87 | 0.71 | 4.78 | -3.59 | 1.69E-08 | 1.13E-06 | 5.946922 | -3.59 | down |
| chr5 | SLCO4C1 | 1.01E+08 | 1.01E+08 | 401 | * | 5.46 | 4.54 | 6.02 | -1.26 | 7.67E-09 | 5.54E-07 | 6.25649 | -1.26 | down |
| chr8 | FBXO43 | 1.01E+08 | 1.01E+08 | 401 | * | 4.75 | 3.63 | 5.38 | -1.47 | 1.52E-07 | 8.01E-06 | 5.096367 | -1.47 | down |
| chr7 | ACHE | 1E+08 | 1E+08 | 401 | * | 5.27 | 4.28 | 5.86 | -1.34 | 1.25E-08 | 8.67E-07 | 6.061981 | -1.34 | down |
| chr11 | ARHGAP42 | 1E+08 | 1E+08 | 401 | * | 4.18 | 2.89 | 4.85 | -1.55 | 3.22E-06 | 0.000115 | 3.939302 | -1.55 | down |
| chr4 | ADH1B | 1E+08 | 1E+08 | 401 | * | 5.17 | 4.33 | 5.69 | -1.09 | 1.15E-06 | 0.000047 | 4.327902 | -1.09 | down |
| chr11 | CNTN5 | 1E+08 | 1E+08 | 401 | * | 3.88 | 1.51 | 4.74 | -2.89 | 3.65E-08 | 2.25E-06 | 5.647817 | -2.89 | down |
| chr11 | CNTN5 | 99766815 | 99767215 | 401 | * | 5.64 | 4.06 | 6.38 | -2.27 | 5.73E-18 | 1.89E-15 | 14.72354 | -2.27 | down |
| chr10 | PI4K2A | 99345372 | 99345772 | 401 | * | 4.21 | 2.53 | 4.97 | -2.2 | 5.83E-08 | 3.41E-06 | 5.467246 | -2.2 | down |
| chr10 | ANKRD2 | 99337262 | 99337662 | 401 | * | 3.11 | 0.41 | 4 | -2.69 | 1.84E-05 | 0.000501 | 3.300162 | -2.69 | down |
| chr10 | ANKRD2 | 99329556 | 99329956 | 401 | * | 5.51 | 2.3 | 6.42 | -3.97 | 1.54E-23 | 9.30E-21 | 20.03152 | -3.97 | down |
| chr9 | CDC14B | 99301971 | 99302371 | 401 | * | 5.63 | 4.75 | 6.18 | -1.24 | 2.44E-09 | 1.97E-07 | 6.705534 | -1.24 | down |
| chr8 | SNORA72 | 99027752 | 99028152 | 401 | * | 5.23 | 3.92 | 5.9 | -1.84 | 2.47E-11 | 3E-09 | 8.522879 | -1.84 | down |
| chr8 | MATN2 | 98972272 | 98972672 | 401 | * | 5.18 | 4.16 | 5.78 | -1.36 | 3.26E-08 | 2.04E-06 | 5.69037 | -1.36 | down |
| chr14 | C14orf177 | 98926158 | 98926558 | 401 | * | 4.59 | 3.69 | 5.14 | -1.03 | 0.000029 | 0.00073 | 3.136677 | -1.03 | down |

| | | | | | | | | | | | | | | |
|---|---|---|---|---|---|---|---|---|---|---|---|---|---|---|
| chr15 | ARRDC4 | 98703259 | 98703659 | 401 | * | 4.49 | 3.58 | 5.04 | -1.01 | 5.98E-05 | 0.00133 | 2.876148 | -1.01 | down |
| chr7 | TRRAP | 98617017 | 98617417 | 401 | * | 5.75 | 4.92 | 6.27 | -1.16 | 4.48E-09 | 3.45E-07 | 6.462181 | -1.16 | down |
| chr3 | DCBLD2 | 98556193 | 98556593 | 401 | * | 4.82 | 2.45 | 5.67 | -3.06 | 2.85E-14 | 5.55E-12 | 11.25571 | -3.06 | down |
| chr7 | TMEM130 | 98467934 | 98468334 | 401 | * | 3.68 | 2.4 | 4.35 | -1.27 | 9.61E-05 | 0.00198 | 2.703335 | -1.27 | down |
| chr4 | STPG2-AS1 | 98447100 | 98447500 | 401 | * | 4.58 | 3.19 | 5.27 | -1.87 | 2.04E-08 | 1.33E-06 | 5.876148 | -1.87 | down |
| chr2 | TMEM131 | 98378914 | 98379314 | 401 | * | 5.45 | 4.63 | 5.97 | -1.13 | 8.2E-08 | 4.63E-06 | 5.334419 | -1.13 | down |
| chr15 | LINC00923 | 98351666 | 98352066 | 401 | * | 5.96 | 4.95 | 6.55 | -1.46 | 3.14E-13 | 5.21E-11 | 10.28316 | -1.46 | down |
| chr13 | MBNL2 | 97889740 | 97890140 | 401 | * | 5.27 | 4.49 | 5.78 | -1.04 | 1.14E-06 | 4.68E-05 | 4.329754 | -1.04 | down |
| chr9 | AOPEP | 97714414 | 97714814 | 401 | * | 6.22 | 5.29 | 6.78 | -1.37 | 5.66E-14 | 1.06E-11 | 10.97469 | -1.37 | down |
| chr8 | PTDSS1 | 97277747 | 97278147 | 401 | * | 5.06 | 3.9 | 5.7 | -1.58 | 6.16E-09 | 4.57E-07 | 6.340084 | -1.58 | down |
| chr10 | SORBS1 | 97222278 | 97222678 | 401 | * | 3.9 | 2.28 | 4.65 | -2 | 1.47E-06 | 5.84E-05 | 4.233587 | -2 | down |
| chr9 | MFSD14B | 97221386 | 97221786 | 401 | * | 6.02 | 5.09 | 6.58 | -1.36 | 2.78E-12 | 3.97E-10 | 9.401209 | -1.36 | down |
| chr8 | LOC100500773 | 96911758 | 96912158 | 401 | * | 4.24 | 2.85 | 4.93 | -1.75 | 8.86E-07 | 3.75E-05 | 4.425969 | -1.75 | down |
| chr8 | LOC100500773 | 96829900 | 96830300 | 401 | * | 6.77 | 5.99 | 7.27 | -1.18 | 2.23E-15 | 5.09E-13 | 12.29328 | -1.18 | down |
| chr2 | ADRA2B | 96764246 | 96764646 | 401 | * | 5.47 | 4.54 | 6.04 | -1.29 | 1.07E-08 | 7.55E-07 | 6.122053 | -1.29 | down |
| chr8 | LOC100500773 | 96761056 | 96761456 | 401 | * | 3.38 | 1.53 | 4.16 | -1.9 | 3.39E-05 | 0.000827 | 3.082494 | -1.9 | down |
| chr10 | CYP2C9 | 96744114 | 96744514 | 401 | * | 3.89 | 2.54 | 4.58 | -1.51 | 1.58E-05 | 0.000441 | 3.355561 | -1.51 | down |
| chr15 | NR2F2 | 96736436 | 96736836 | 401 | * | 5.28 | 4.35 | 5.84 | -1.24 | 4.95E-08 | 2.96E-06 | 5.528708 | -1.24 | down |
| chr15 | NR2F2 | 96514719 | 96515119 | 401 | * | 3.87 | 1.7 | 4.7 | -2.7 | 1.03E-07 | 5.67E-06 | 5.246417 | -2.7 | down |
| chr2 | FAHD2CP | 96498129 | 96498529 | 401 | * | 7.11 | 5.39 | 7.87 | -2.46 | 2.01E-50 | 1.03E-46 | 45.98716 | -2.46 | down |
| chr15 | LINC00924 | 96345573 | 96345973 | 401 | * | 4.19 | 1.86 | 5.04 | -2.97 | 8.33E-10 | 7.51E-08 | 7.12436 | -2.97 | down |
| chr15 | LINC00924 | 96340940 | 96341340 | 401 | * | 3.58 | 1.53 | 4.39 | -2.42 | 2.43E-06 | 8.96E-05 | 4.047692 | -2.42 | down |
| chr15 | LINC00924 | 96327149 | 96327549 | 401 | * | 4 | 1.92 | 4.82 | -2.62 | 4.22E-08 | 2.56E-06 | 5.59176 | -2.62 | down |
| chr15 | LINC00924 | 96322651 | 96323051 | 401 | * | 3.17 | 1.53 | 3.92 | -1.34 | 0.000253 | 0.0043 | 2.366532 | -1.34 | down |
| chr15 | LINC00924 | 96273698 | 96274098 | 401 | * | 7.66 | 3.34 | 8.63 | -5.21 | 1.02E-82 | 2.25E-78 | 77.64782 | -5.21 | down |
| chr15 | LINC00924 | 96234008 | 96234408 | 401 | * | 5.09 | 0.32 | 6.06 | -5.07 | 3.70E-14 | 7.11E-12 | 11.14813 | -5.07 | down |
| chr6 | MANEA | 96151282 | 96151682 | 401 | * | 5.85 | 5.01 | 6.38 | -1.21 | 8.78E-10 | 7.9E-08 | 7.102373 | -1.21 | down |
| chr4 | BMPR1B | 96103095 | 96103495 | 401 | * | 6.09 | 5.1 | 6.67 | -1.45 | 5.13E-14 | 9.65E-12 | 11.01547 | -1.45 | down |

| | | | | | | | | | | | | | | |
|---|---|---|---|---|---|---|---|---|---|---|---|---|---|---|
| chr6 | MANEA | 96015481 | 96015881 | 401 | * | 8.25 | 7.27 | 8.82 | -1.53 | 1.58E-54 | 9.53E-51 | 50.02091 | -1.53 | down |
| chr6 | MANEA | 96010171 | 96010571 | 401 | * | 6.5 | 5.69 | 7.01 | -1.2 | 1.50E-13 | 2.61E-11 | 10.58336 | -1.2 | down |
| chr15 | LINC00924 | 95929330 | 95929730 | 401 | * | 5.59 | 4.61 | 6.17 | -1.37 | 1.01E-09 | 8.93E-08 | 7.049149 | -1.37 | down |
| chr5 | PCSK1 | 95878461 | 95878861 | 401 | * | 4.62 | 3.69 | 5.19 | -1.1 | 1.19E-05 | 0.000346 | 3.460924 | -1.1 | down |
| chr15 | LINC01197 | 95735062 | 95735462 | 401 | * | 6.06 | 2.92 | 6.98 | -3.96 | 2.61E-33 | 3.40E-30 | 29.46852 | -3.96 | down |
| chr15 | LINC01197 | 95728751 | 95729151 | 401 | * | 4.47 | 1.18 | 5.4 | -3.86 | 2.93E-12 | 4.18E-10 | 9.378824 | -3.86 | down |
| chr15 | LINC01197 | 95710045 | 95710445 | 401 | * | 7.25 | 3.84 | 8.18 | -4.29 | 1.96E-73 | 2.60E-69 | 68.58503 | -4.29 | down |
| chr15 | LINC01197 | 95698223 | 95698623 | 401 | * | 4.04 | 0 | 5.03 | -4.93 | 7.39E-08 | 4.23E-06 | 5.37366 | -4.93 | down |
| chr15 | LINC01197 | 95684700 | 95685100 | 401 | * | 5.6 | 2.35 | 6.52 | -3.98 | 1.33E-24 | 8.94E-22 | 21.04866 | -3.98 | down |
| chr15 | LINC01197 | 95645740 | 95646140 | 401 | * | 3.02 | 0.68 | 3.87 | -2.13 | 6.26E-05 | 0.00139 | 2.856985 | -2.13 | down |
| chr15 | LOC440311 | 95559831 | 95560231 | 401 | * | 4.83 | 1.98 | 5.72 | -3.55 | 3.05E-15 | 6.79E-13 | 12.16813 | -3.55 | down |
| chr1 | TLCD4 | 95557218 | 95557618 | 401 | * | 6.45 | 5.76 | 6.92 | -1.04 | 5.59E-11 | 6.29E-09 | 8.201349 | -1.04 | down |
| chr15 | LOC440311 | 95284925 | 95285325 | 401 | * | 3.43 | 0.37 | 4.34 | -3.26 | 1.64E-06 | 6.38E-05 | 4.195179 | -3.26 | down |
| chr10 | CEP55 | 95260524 | 95260924 | 401 | * | 5.71 | 4.56 | 6.35 | -1.44 | 1.09E-06 | 4.51E-05 | 4.345823 | -1.44 | down |
| chr2 | FAM95A | 95025747 | 95026147 | 401 | * | 3.7 | 2.52 | 4.33 | -1.04 | 0.000293 | 0.00485 | 2.314258 | -1.04 | down |
| chr8 | MIR378D2 | 94921028 | 94921428 | 401 | * | 7.04 | 6.08 | 7.61 | -1.47 | 1.21E-22 | 6.90E-20 | 19.16115 | -1.47 | down |
| chr15 | MCTP2 | 94845062 | 94845462 | 401 | * | 4.04 | 2.41 | 4.79 | -2.1 | 4.62E-07 | 2.13E-05 | 4.67162 | -2.1 | down |
| chrX | BRDTP1 | 94674009 | 94674409 | 401 | * | 4.75 | 0.37 | 5.72 | -4.73 | 1.80E-12 | 2.67E-10 | 9.573489 | -4.73 | down |
| chr6 | TSG1 | 94636952 | 94637352 | 401 | * | 4.16 | 1.18 | 5.06 | -3.53 | 3.32E-10 | 3.22E-08 | 7.492144 | -3.53 | down |
| chr10 | IDE | 94274813 | 94275213 | 401 | * | 4.33 | 3.23 | 4.94 | -1.23 | 2.03E-05 | 0.000543 | 3.2652 | -1.23 | down |
| chr8 | FLJ46284 | 93551880 | 93552280 | 401 | * | 5.61 | 4.67 | 6.18 | -1.34 | 3.02E-10 | 2.96E-08 | 7.528708 | -1.34 | down |
| chr6 | EPHA7 | 93472126 | 93472526 | 401 | * | 3.32 | 0.37 | 4.22 | -3.12 | 3.15E-06 | 0.000113 | 3.946922 | -3.12 | down |
| chr10 | PPP1R3C | 93458780 | 93459180 | 401 | * | 3.78 | 2.45 | 4.46 | -1.39 | 4.43E-05 | 0.00104 | 2.982967 | -1.39 | down |
| chr6 | EPHA7 | 93419478 | 93419878 | 401 | * | 6.24 | 4.31 | 7.04 | -2.69 | 8.80E-31 | 8.59E-28 | 27.06601 | -2.69 | down |
| chr13 | GPC5-AS1 | 93108070 | 93108470 | 401 | * | 4.14 | 2.95 | 4.78 | -1.34 | 1.39E-05 | 0.000396 | 3.402305 | -1.34 | down |
| chr6 | MIR4643 | 93105093 | 93105493 | 401 | * | 4.97 | 3.37 | 5.71 | -2.23 | 5.16E-12 | 6.95E-10 | 9.158015 | -2.23 | down |
| chr6 | MIR4643 | 93021506 | 93021906 | 401 | * | 4.96 | 2.88 | 5.77 | -2.77 | 1.18E-14 | 2.46E-12 | 11.60906 | -2.77 | down |
| chr6 | MIR4643 | 93017096 | 93017496 | 401 | * | 4.96 | 3.52 | 5.67 | -1.98 | 2.86E-10 | 2.82E-08 | 7.549751 | -1.98 | down |

| | | | | | | | | | | | | | | |
|---|---|---|---|---|---|---|---|---|---|---|---|---|---|---|
| chr6 | MIR4643 | 93005312 | 93005712 | 401 | * | 3.42 | 1.82 | 4.16 | -1.48 | 0.000109 | 0.0022 | 2.657577 | -1.48 | down |
| chr8 | RUNX1T1 | 92945208 | 92945608 | 401 | * | 6.54 | 5.85 | 7 | -1.04 | 2.14E-11 | 2.63E-09 | 8.580044 | -1.04 | down |
| chr15 | SLCO3A1 | 92655591 | 92655991 | 401 | * | 4.28 | 1.98 | 5.12 | -2.94 | 3.51E-10 | 3.38E-08 | 7.471083 | -2.94 | down |
| chr7 | CDK6 | 92442784 | 92443184 | 401 | * | 6.7 | 5.8 | 7.25 | -1.37 | 4.20E-18 | 1.40E-15 | 14.85387 | -1.37 | down |
| chr10 | HTR7 | 92402329 | 92402729 | 401 | * | 3.56 | 1.82 | 4.33 | -1.93 | 1.49E-05 | 0.000419 | 3.377786 | -1.93 | down |
| chr10 | HTR7 | 92381603 | 92382003 | 401 | * | 3.34 | 1.8 | 4.07 | -1.3 | 0.000223 | 0.00391 | 2.407823 | -1.3 | down |
| chr9 | UNQ6494 | 92354918 | 92355318 | 401 | * | 6.09 | 5.18 | 6.64 | -1.33 | 1.03E-12 | 1.60E-10 | 9.79588 | -1.33 | down |
| chr6 | MIR4643 | 92201584 | 92201984 | 401 | * | 4.27 | 2.13 | 5.09 | -2.8 | 1.22E-09 | 1.05E-07 | 6.978811 | -2.8 | down |
| chr6 | MIR4643 | 91976815 | 91977215 | 401 | * | 4.28 | 2.39 | 5.07 | -2.49 | 5.88E-09 | 4.36E-07 | 6.360514 | -2.49 | down |
| chr10 | PANK1 | 91380894 | 91381294 | 401 | * | 4.85 | 4.01 | 5.38 | -1.02 | 1.23E-05 | 0.000357 | 3.447332 | -1.02 | down |
| chr13 | MIR622 | 91017516 | 91017916 | 401 | * | 5.39 | 4.49 | 5.94 | -1.23 | 2.51E-08 | 1.61E-06 | 5.793174 | -1.23 | down |
| chr14 | LINC00642 | 90953621 | 90954021 | 401 | * | 6.43 | 5.74 | 6.89 | -1.04 | 5.98E-11 | 6.70E-09 | 8.173925 | -1.04 | down |
| chr9 | SPATA31C2 | 90815421 | 90815821 | 401 | * | 4.39 | 3.37 | 4.98 | -1.16 | 2.01E-05 | 0.00054 | 3.267606 | -1.16 | down |
| chr8 | RIPK2 | 90791622 | 90792022 | 401 | * | 4.88 | 3.99 | 5.43 | -1.1 | 4.75E-06 | 0.00016 | 3.79588 | -1.1 | down |
| chr8 | RIPK2 | 90777173 | 90777573 | 401 | * | 4 | 2.75 | 4.67 | -1.38 | 1.95E-05 | 0.000526 | 3.279014 | -1.38 | down |
| chr4 | SNCA | 90648782 | 90649182 | 401 | * | 4.15 | 2.16 | 4.96 | -2.56 | 1.77E-08 | 1.17E-06 | 5.931814 | -2.56 | down |
| chr10 | ANKRD22 | 90602813 | 90603213 | 401 | * | 4.95 | 3.42 | 5.67 | -2.17 | 3.10E-11 | 3.68E-09 | 8.434152 | -2.17 | down |
| chr5 | ARRDC3-AS1 | 90558430 | 90558830 | 401 | * | 5.16 | 4.22 | 5.72 | -1.26 | 9.27E-08 | 5.17E-06 | 5.286509 | -1.26 | down |
| chr7 | CDK14 | 90485785 | 90486185 | 401 | * | 6.05 | 5.25 | 6.55 | -1.14 | 2.71E-10 | 2.68E-08 | 7.571865 | -1.14 | down |
| chr7 | CDK14 | 90119294 | 90119694 | 401 | * | 3.41 | 1.51 | 4.2 | -2.05 | 1.76E-05 | 0.000482 | 3.316953 | -2.05 | down |
| chr13 | LINC00353 | 89920393 | 89920793 | 401 | * | 4.77 | 2.52 | 5.61 | -2.92 | 2.38E-13 | 4.04E-11 | 10.39362 | -2.92 | down |
| chr9 | GAS1 | 89519289 | 89519689 | 401 | * | 3.87 | 2.77 | 4.49 | -1.06 | 0.00017 | 0.00314 | 2.50307 | -1.06 | down |
| chr9 | GAS1 | 89461268 | 89461668 | 401 | * | 4.91 | 3.98 | 5.47 | -1.2 | 1.15E-06 | 4.71E-05 | 4.326979 | -1.2 | down |
| chr16 | ANKRD11 | 89357340 | 89357740 | 401 | * | 5.6 | 4.7 | 6.15 | -1.25 | 2.73E-09 | 2.19E-07 | 6.659556 | -1.25 | down |
| chr10 | MIR4678 | 89251820 | 89252220 | 401 | * | 7.93 | 6.73 | 8.58 | -1.83 | 1.29E-58 | 8.57E-55 | 54.06702 | -1.83 | down |
| chr6 | CNR1 | 89190185 | 89190585 | 401 | * | 5.48 | 4.69 | 5.98 | -1.04 | 7.15E-07 | 3.12E-05 | 4.505845 | -1.04 | down |
| chr10 | SHLD2 | 88897387 | 88897787 | 401 | * | 3.9 | 2.21 | 4.65 | -2.11 | 1.14E-06 | 4.68E-05 | 4.329754 | -2.11 | down |
| chr10 | GLUD1 | 88830597 | 88830997 | 401 | * | 5.75 | 3.68 | 6.57 | -2.81 | 9.37E-24 | 5.92E-21 | 20.22768 | -2.81 | down |

| | | | | | | | | | | | | | | |
|---|---|---|---|---|---|---|---|---|---|---|---|---|---|---|
| chr10 | FAM25A | 88800743 | 88801143 | 401 | * | 6.68 | 5.66 | 7.27 | -1.54 | 8.41E-21 | 3.91E-18 | 17.40782 | -1.54 | down |
| chr10 | ADIRF | 88728402 | 88728802 | 401 | * | 4.52 | 3.51 | 5.11 | -1.21 | 6.94E-06 | 0.00022 | 3.657577 | -1.21 | down |
| chr2 | FOXI3 | 88683284 | 88683684 | 401 | * | 4.88 | 3.16 | 5.64 | -2.35 | 4.84E-12 | 6.56E-10 | 9.183096 | -2.35 | down |
| chr10 | BMPR1A | 88582974 | 88583374 | 401 | * | 4.37 | 3.18 | 5.01 | -1.44 | 3.74E-06 | 0.00013 | 3.886057 | -1.44 | down |
| chr14 | LINC01146 | 88551389 | 88551789 | 401 | * | 5.09 | 2.72 | 5.94 | -3.11 | 4.95E-17 | 1.45E-14 | 13.83863 | -3.11 | down |
| chr6 | RARS2 | 88246649 | 88247049 | 401 | * | 6.27 | 5.55 | 6.75 | -1.05 | 4.95E-10 | 4.63E-08 | 7.334419 | -1.05 | down |
| chr2 | RGPD1 | 88239727 | 88240127 | 401 | * | 4.67 | 3.36 | 5.35 | -1.8 | 1.9E-08 | 1.25E-06 | 5.90309 | -1.8 | down |
| chr8 | CNBD1 | 87936017 | 87936417 | 401 | * | 4.1 | 0.72 | 5.03 | -3.84 | 1.30E-09 | 1.12E-07 | 6.950782 | -3.84 | down |
| chr10 | MIR346 | 87921891 | 87922291 | 401 | * | 3.47 | 2.18 | 4.14 | -1.02 | 0.000505 | 0.0075 | 2.124939 | -1.02 | down |
| chr7 | SRI | 87857943 | 87858343 | 401 | * | 4.5 | 3.55 | 5.06 | -1.1 | 2.13E-05 | 0.000564 | 3.248721 | -1.1 | down |
| chr13 | MIR4500 | 87671189 | 87671589 | 401 | * | 5.54 | 3.8 | 6.31 | -2.44 | 1.15E-18 | 4.05E-16 | 15.39254 | -2.44 | down |
| chr12 | MGAT4C | 87580154 | 87580554 | 401 | * | 3.66 | 0.94 | 4.55 | -3.15 | 1.72E-07 | 8.91E-06 | 5.050122 | -3.15 | down |
| chr8 | WWP1 | 87447326 | 87447726 | 401 | * | 4.04 | 1.93 | 4.86 | -2.66 | 3.29E-08 | 2.05E-06 | 5.688246 | -2.66 | down |
| chr9 | NTRK2 | 87445633 | 87446033 | 401 | * | 4.92 | 3.86 | 5.53 | -1.39 | 1.18E-07 | 6.37E-06 | 5.195861 | -1.39 | down |
| chrX | CPXCR1 | 87409441 | 87409841 | 401 | * | 5.37 | 2.09 | 6.29 | -4.02 | 3.88E-21 | 1.91E-18 | 17.71897 | -4.02 | down |
| chr16 | FBXO31 | 87407526 | 87407926 | 401 | * | 5.37 | 4.35 | 5.97 | -1.44 | 1.14E-09 | 9.86E-08 | 7.006123 | -1.44 | down |
| chrX | CPXCR1 | 87397440 | 87397840 | 401 | * | 4.5 | 0.32 | 5.46 | -4.47 | 3.56E-11 | 4.18E-09 | 8.378824 | -4.47 | down |
| chr16 | C16orf95 | 87305729 | 87306129 | 401 | * | 4.82 | 3.66 | 5.46 | -1.55 | 4.05E-08 | 2.47E-06 | 5.607303 | -1.55 | down |
| chr16 | C16orf95 | 87304903 | 87305303 | 401 | * | 4.58 | 3.68 | 5.13 | -1.03 | 3.07E-05 | 0.000762 | 3.118045 | -1.03 | down |
| chr10 | GRID1-AS1 | 87211813 | 87212213 | 401 | * | 4.94 | 4.11 | 5.46 | -1.03 | 9.05E-06 | 0.000274 | 3.562249 | -1.03 | down |
| chr15 | AGBL1-AS1 | 87167099 | 87167499 | 401 | * | 6.66 | 5.95 | 7.14 | -1.08 | 1.18E-12 | 1.79E-10 | 9.747147 | -1.08 | down |
| chr16 | C16orf95 | 87145431 | 87145831 | 401 | * | 5.15 | 3.23 | 5.95 | -2.63 | 7.78E-16 | 1.90E-13 | 12.72125 | -2.63 | down |
| chr7 | ABCB4 | 87123102 | 87123502 | 401 | * | 6.25 | 5.53 | 6.72 | -1.06 | 2.03E-10 | 2.07E-08 | 7.68403 | -1.06 | down |
| chr10 | GRID1-AS1 | 87054931 | 87055331 | 401 | * | 3.99 | 2.62 | 4.68 | -1.58 | 7.79E-06 | 0.000242 | 3.616185 | -1.58 | down |
| chr4 | MAPK10 | 86996494 | 86996894 | 401 | * | 4.05 | 2.71 | 4.73 | -1.55 | 1.02E-05 | 0.000303 | 3.518557 | -1.55 | down |
| chr16 | FOXL1 | 86901490 | 86901890 | 401 | * | 5.24 | 4.19 | 5.84 | -1.45 | 4.04E-09 | 3.12E-07 | 6.505845 | -1.45 | down |
| chr14 | LINC01148 | 86799317 | 86799717 | 401 | * | 5.23 | 3.57 | 5.98 | -2.31 | 2.73E-14 | 5.34E-12 | 11.27246 | -2.31 | down |
| chr14 | LINC01148 | 86787919 | 86788319 | 401 | * | 4.19 | 2.28 | 4.98 | -2.48 | 1.91E-08 | 1.26E-06 | 5.899629 | -2.48 | down |

| | | | | | | | | | | | | | | |
|---|---|---|---|---|---|---|---|---|---|---|---|---|---|---|
| chr14 | LINC01148 | 86775859 | 86776259 | 401 | * | 5.2 | 3.13 | 6.02 | -2.79 | 6.16E-17 | 1.76E-14 | 13.75449 | -2.79 | down |
| chr10 | CCSER2 | 86535403 | 86535803 | 401 | * | 7.27 | 6.52 | 7.77 | -1.18 | 2.98E-20 | 1.25E-17 | 16.90309 | -1.18 | down |
| chr9 | IDNK | 86230845 | 86231245 | 401 | * | 7.9 | 6.8 | 8.51 | -1.7 | 1.43E-50 | 7.90E-47 | 46.10237 | -1.7 | down |
| chrX | DACH2 | 86148134 | 86148534 | 401 | * | 4.36 | 0.32 | 5.32 | -4.34 | 1.72E-10 | 1.8E-08 | 7.744727 | -4.34 | down |
| chr16 | MIR1910 | 85778966 | 85779366 | 401 | * | 4.7 | 2.14 | 5.58 | -3.22 | 9.46E-14 | 1.71E-11 | 10.767 | -3.22 | down |
| chr13 | LINC00351 | 85675225 | 85675625 | 401 | * | 3.19 | 0 | 4.12 | -3.14 | 8.69E-06 | 0.000265 | 3.576754 | -3.14 | down |
| chr15 | PDE8A | 85543719 | 85544119 | 401 | * | 3.47 | 2.19 | 4.14 | -1.08 | 0.000351 | 0.00561 | 2.251037 | -1.08 | down |
| chr7 | SEMA3D | 85489519 | 85489919 | 401 | * | 4.5 | 1.35 | 5.42 | -3.74 | 1.42E-12 | 2.14E-10 | 9.669586 | -3.74 | down |
| chr8 | RALYL | 85323734 | 85324134 | 401 | * | 7.56 | 6.86 | 8.04 | -1.12 | 2.99E-22 | 1.63E-19 | 18.78781 | -1.12 | down |
| chr7 | SEMA3D | 85186698 | 85187098 | 401 | * | 4.2 | 1.95 | 5.04 | -2.86 | 1.28E-09 | 1.1E-07 | 6.958607 | -2.86 | down |
| chr13 | LINC00333 | 85093811 | 85094211 | 401 | * | 4.4 | 2.69 | 5.17 | -2.29 | 4.74E-09 | 3.61E-07 | 6.442493 | -2.29 | down |
| chr13 | LINC00333 | 85082734 | 85083134 | 401 | * | 3.57 | 2.16 | 4.27 | -1.31 | 0.000133 | 0.00258 | 2.58838 | -1.31 | down |
| chr13 | LINC00333 | 85080649 | 85081049 | 401 | * | 4.57 | 1.7 | 5.47 | -3.52 | 4.66E-13 | 7.57E-11 | 10.1209 | -3.52 | down |
| chr12 | SLC6A15 | 84912664 | 84913064 | 401 | * | 4.63 | 3.62 | 5.21 | -1.23 | 3.92E-06 | 0.000135 | 3.869666 | -1.23 | down |
| chr9 | SPATA31D1 | 84782486 | 84782886 | 401 | * | 5.92 | 5.06 | 6.46 | -1.24 | 1.67E-10 | 1.75E-08 | 7.756962 | -1.24 | down |
| chr6 | MRAP2 | 84774671 | 84775071 | 401 | * | 6.51 | 5.62 | 7.05 | -1.33 | 7.46E-16 | 1.83E-13 | 12.73755 | -1.33 | down |
| chr6 | MRAP2 | 84764516 | 84764916 | 401 | * | 7.2 | 5.97 | 7.85 | -1.85 | 3.30E-36 | 5.10E-33 | 32.29243 | -1.85 | down |
| chr6 | MRAP2 | 84763430 | 84763830 | 401 | * | 6.49 | 4.33 | 7.31 | -2.94 | 3.12E-40 | 5.76E-37 | 36.23958 | -2.94 | down |
| chr6 | MRAP2 | 84731798 | 84732198 | 401 | * | 3.51 | 2.29 | 4.16 | -1.03 | 0.000403 | 0.00626 | 2.203426 | -1.03 | down |
| chr6 | MRAP2 | 84667524 | 84667924 | 401 | * | 6.35 | 5.3 | 6.94 | -1.56 | 2.12E-17 | 6.40E-15 | 14.19382 | -1.56 | down |
| chrX | ZNF711 | 84501709 | 84502109 | 401 | * | 5.18 | 0 | 6.17 | -5.39 | 4.58E-13 | 7.48E-11 | 10.1261 | -5.39 | down |
| chr9 | TLE1 | 84375962 | 84376362 | 401 | * | 6.57 | 5.78 | 7.07 | -1.18 | 4.32E-14 | 8.20E-12 | 11.08619 | -1.18 | down |
| chr12 | SLC6A15 | 84304255 | 84304655 | 401 | * | 4.43 | 0.37 | 5.39 | -4.41 | 6.41E-11 | 7.14E-09 | 8.146302 | -4.41 | down |
| chr12 | TMTC2 | 84136784 | 84137184 | 401 | * | 3.82 | 0 | 4.79 | -4.21 | 4.37E-07 | 2.03E-05 | 4.692504 | -4.21 | down |
| chr12 | TMTC2 | 83823605 | 83824005 | 401 | * | 4.12 | 1.95 | 4.95 | -2.76 | 4.89E-09 | 3.7E-07 | 6.431798 | -2.76 | down |
| chr7 | SEMA3A | 83821844 | 83822244 | 401 | * | 6.06 | 5.24 | 6.57 | -1.17 | 9.04E-11 | 9.90E-09 | 8.004365 | -1.17 | down |
| chr13 | SLITRK1 | 83780271 | 83780671 | 401 | * | 4.09 | 0.72 | 5.01 | -3.83 | 1.32E-09 | 1.13E-07 | 6.946922 | -3.83 | down |
| chr7 | SEMA3A | 83552652 | 83553052 | 401 | * | 6.06 | 4.88 | 6.69 | -1.73 | 3.08E-17 | 9.13E-15 | 14.03953 | -1.73 | down |

| | | | | | | | | | | | | | | |
|---|---|---|---|---|---|---|---|---|---|---|---|---|---|---|
| chr15 | FSD2 | 83463033 | 83463433 | 401 | * | 5.83 | 4.25 | 6.57 | -2.28 | 5.03E-20 | 2.07E-17 | 16.68403 | -2.28 | down |
| chr12 | TMTC2 | 83335740 | 83336140 | 401 | * | 4.39 | 1.55 | 5.28 | -3.46 | 1.18E-11 | 1.51E-09 | 8.821023 | -3.46 | down |
| chr13 | SLITRK1 | 83190046 | 83190446 | 401 | * | 7.14 | 6.26 | 7.68 | -1.36 | 8.96E-23 | 5.17E-20 | 19.28651 | -1.36 | down |
| chr12 | TMTC2 | 83173219 | 83173619 | 401 | * | 5.7 | 2.81 | 6.6 | -3.7 | 1.44E-26 | 1.10E-23 | 22.95861 | -3.7 | down |
| chr8 | SNX16 | 83167339 | 83167739 | 401 | * | 4.67 | 2.26 | 5.53 | -3.08 | 2.96E-13 | 4.93E-11 | 10.30715 | -3.08 | down |
| chr12 | TMTC2 | 83150851 | 83151251 | 401 | * | 6 | 3.29 | 6.89 | -3.52 | 2.80E-32 | 3.26E-29 | 28.48678 | -3.52 | down |
| chr8 | SNX16 | 83149596 | 83149996 | 401 | * | 5 | 1.39 | 5.94 | -4.25 | 2.56E-16 | 6.72E-14 | 13.17263 | -4.25 | down |
| chr8 | SNX16 | 83073424 | 83073824 | 401 | * | 5.09 | 2.93 | 5.92 | -2.91 | 3.41E-16 | 8.74E-14 | 13.05849 | -2.91 | down |
| chr13 | SLITRK1 | 83057143 | 83057543 | 401 | * | 3.79 | 1.84 | 4.59 | -2.4 | 9.06E-07 | 3.82E-05 | 4.417937 | -2.4 | down |
| chr13 | SLITRK1 | 83053409 | 83053809 | 401 | * | 2.97 | 0.94 | 3.78 | -1.65 | 0.000175 | 0.00321 | 2.493495 | -1.65 | down |
| chr12 | TMTC2 | 83052887 | 83053287 | 401 | * | 3.87 | 1.43 | 4.74 | -3.03 | 2.94E-08 | 1.86E-06 | 5.730487 | -3.03 | down |
| chr12 | TMTC2 | 83016174 | 83016574 | 401 | * | 5.2 | 3.84 | 5.88 | -1.94 | 9.27E-12 | 1.21E-09 | 8.917215 | -1.94 | down |
| chr13 | SLITRK1 | 82962473 | 82962873 | 401 | * | 4.08 | 3.04 | 4.67 | -1.06 | 0.000124 | 0.00243 | 2.614394 | -1.06 | down |
| chr8 | SNX16 | 82961027 | 82961427 | 401 | * | 7.57 | 6.34 | 8.22 | -1.86 | 1.50E-47 | 4.75E-44 | 43.32331 | -1.86 | down |
| chr15 | NA | 82921185 | 82921585 | 401 | * | 4.16 | 2.12 | 4.97 | -2.66 | 1.49E-08 | 1.01E-06 | 5.995679 | -2.66 | down |
| chr8 | SNX16 | 82913393 | 82913793 | 401 | * | 3.49 | 1.71 | 4.26 | -1.9 | 0.000026 | 0.000667 | 3.175874 | -1.9 | down |
| chr13 | SLITRK1 | 82898603 | 82899003 | 401 | * | 5.13 | 2.92 | 5.97 | -2.96 | 5.38E-17 | 1.55E-14 | 13.80967 | -2.96 | down |
| chr5 | VCAN | 82854951 | 82855351 | 401 | * | 4.66 | 3.68 | 5.24 | -1.19 | 6.51E-06 | 0.000209 | 3.679854 | -1.19 | down |
| chr13 | SLITRK1 | 82719977 | 82720377 | 401 | * | 4.5 | 0.37 | 5.46 | -4.47 | 3.11E-11 | 3.68E-09 | 8.434152 | -4.47 | down |
| chr13 | SPRY2 | 82637234 | 82637634 | 401 | * | 4.4 | 2.42 | 5.2 | -2.64 | 6.75E-10 | 6.13E-08 | 7.21254 | -2.64 | down |
| chr2 | DHFRP3 | 82636325 | 82636725 | 401 | * | 4.82 | 3.07 | 5.59 | -2.41 | 8.05E-12 | 1.06E-09 | 8.974694 | -2.41 | down |
| chr13 | SPRY2 | 82565201 | 82565601 | 401 | * | 6.88 | 5.24 | 7.63 | -2.37 | 1.61E-41 | 3.15E-38 | 37.50169 | -2.37 | down |
| chr8 | IMPA1 | 82557678 | 82558078 | 401 | * | 3.9 | 1.68 | 4.73 | -2.76 | 5.45E-08 | 3.21E-06 | 5.493495 | -2.76 | down |
| chr13 | SPRY2 | 82477449 | 82477849 | 401 | * | 4.56 | 2.17 | 5.41 | -3.06 | 2.08E-12 | 3.05E-10 | 9.5157 | -3.06 | down |
| chr16 | CMIP | 81551446 | 81551846 | 401 | * | 5.66 | 4.8 | 6.19 | -1.2 | 4.80E-09 | 3.65E-07 | 6.437707 | -1.2 | down |
| chr3 | GBE1 | 81329958 | 81330358 | 401 | * | 3.57 | 1.57 | 4.38 | -2.38 | 3.29E-06 | 0.000116 | 3.935542 | -2.38 | down |
| chr13 | SPRY2 | 81285843 | 81286243 | 401 | * | 4.11 | 0.41 | 5.06 | -4.08 | 1.89E-09 | 1.56E-07 | 6.806875 | -4.08 | down |
| chr16 | CDYL2 | 80931963 | 80932363 | 401 | * | 3.59 | 2.32 | 4.26 | -1.13 | 0.000267 | 0.00449 | 2.347754 | -1.13 | down |

| | | | | | | | | | | | | | | |
|---|---|---|---|---|---|---|---|---|---|---|---|---|---|---|
| chr14 | DIO2 | 80930742 | 80931142 | 401 | * | 5.75 | 4.9 | 6.28 | -1.21 | 2.02E-09 | 1.66E-07 | 6.779892 | -1.21 | down |
| chr13 | SPRY2 | 80924599 | 80924999 | 401 | * | 5.95 | 4.28 | 6.71 | -2.35 | 5.75E-22 | 3.08E-19 | 18.51145 | -2.35 | down |
| chr13 | SPRY2 | 80897588 | 80897988 | 401 | * | 3.89 | 2.8 | 4.5 | -1.07 | 0.000167 | 0.0031 | 2.508638 | -1.07 | down |
| chr10 | ZMIZ1-AS1 | 80772041 | 80772441 | 401 | * | 4.44 | 3.08 | 5.12 | -1.77 | 1.51E-07 | 7.93E-06 | 5.100727 | -1.77 | down |
| chr17 | FN3K | 80689221 | 80689621 | 401 | * | 4.65 | 3.17 | 5.37 | -1.99 | 7.04E-09 | 5.15E-07 | 6.288193 | -1.99 | down |
| chr7 | SEMA3C | 80471183 | 80471583 | 401 | * | 5.09 | 4.09 | 5.68 | -1.35 | 6E-08 | 3.5E-06 | 5.455932 | -1.35 | down |
| chr9 | GNAQ | 80377322 | 80377722 | 401 | * | 5.99 | 4.8 | 6.63 | -1.76 | 2.36E-15 | 5.37E-13 | 12.27003 | -1.76 | down |
| chr4 | NAA11 | 80202984 | 80203384 | 401 | * | 6.25 | 5.29 | 6.82 | -1.42 | 5.43E-15 | 1.17E-12 | 11.93181 | -1.42 | down |
| chr18 | PARD6G | 80164804 | 80165204 | 401 | * | 4.79 | 2.77 | 5.6 | -2.7 | 5.89E-13 | 9.49E-11 | 10.02273 | -2.7 | down |
| chr17 | CCDC57 | 80152236 | 80152636 | 401 | * | 6.23 | 5.53 | 6.7 | -1.03 | 9.74E-10 | 8.64E-08 | 7.063486 | -1.03 | down |
| chr2 | CTNNA2 | 80079333 | 80079733 | 401 | * | 3.91 | 2.69 | 4.57 | -1.29 | 4.24E-05 | 0.001 | 3 | -1.29 | down |
| chr12 | SYT1 | 78916865 | 78917265 | 401 | * | 4.59 | 2.99 | 5.33 | -2.14 | 5.45E-09 | 4.08E-07 | 6.38934 | -2.14 | down |
| chr1 | MIGA1 | 78297876 | 78298276 | 401 | * | 6.41 | 5.72 | 6.88 | -1.04 | 9.17E-11 | 1E-08 | 8 | -1.04 | down |
| chrX | LPAR4 | 78068886 | 78069286 | 401 | * | 5.78 | 4.93 | 6.31 | -1.19 | 1.79E-09 | 1.48E-07 | 6.829738 | -1.19 | down |
| chr15 | LINGO1 | 78034519 | 78034919 | 401 | * | 3.97 | 2.72 | 4.63 | -1.41 | 1.93E-05 | 0.00052 | 3.283997 | -1.41 | down |
| chrX | LPAR4 | 78024352 | 78024752 | 401 | * | 5.75 | 4.86 | 6.29 | -1.24 | 1.13E-09 | 9.77E-08 | 7.010105 | -1.24 | down |
| chr15 | LINGO1-AS1 | 77944408 | 77944808 | 401 | * | 4.52 | 2.46 | 5.34 | -2.72 | 3.29E-11 | 3.88E-09 | 8.411168 | -2.72 | down |
| chr3 | ROBO2 | 77576951 | 77577351 | 401 | * | 2.43 | 0 | 3.35 | -1.22 | 0.000812 | 0.011 | 1.958607 | -1.22 | down |
| chr17 | RBFOX3 | 77516832 | 77517232 | 401 | * | 4.01 | 2.25 | 4.78 | -2.2 | 3.99E-07 | 1.87E-05 | 4.728158 | -2.2 | down |
| chr11 | RSF1 | 77503055 | 77503455 | 401 | * | 5.84 | 5.06 | 6.34 | -1.09 | 1.09E-08 | 7.65E-07 | 6.116339 | -1.09 | down |
| chr6 | IMPG1 | 77463362 | 77463762 | 401 | * | 3.76 | 2.15 | 4.5 | -1.81 | 1.11E-05 | 0.000328 | 3.484126 | -1.81 | down |
| chr7 | PHTF2 | 77438189 | 77438589 | 401 | * | 3.8 | 2.64 | 4.44 | -1.11 | 0.000171 | 0.00315 | 2.501689 | -1.11 | down |
| chr6 | IMPG1 | 77383023 | 77383423 | 401 | * | 5.8 | 4.45 | 6.49 | -1.98 | 4.02E-17 | 1.18E-14 | 13.92812 | -1.98 | down |
| chr3 | ROBO2 | 77348250 | 77348650 | 401 | * | 5 | 1.16 | 5.95 | -4.4 | 8.05E-16 | 1.96E-13 | 12.70774 | -4.4 | down |
| chr3 | ROBO2 | 77298488 | 77298888 | 401 | * | 3.23 | 0.97 | 4.07 | -2.39 | 1.62E-05 | 0.00045 | 3.346787 | -2.39 | down |
| chr3 | ROBO2 | 77296539 | 77296939 | 401 | * | 4.09 | 2.28 | 4.87 | -2.35 | 7.46E-08 | 4.26E-06 | 5.37059 | -2.35 | down |
| chr6 | IMPG1 | 77293113 | 77293513 | 401 | * | 5.32 | 4 | 6 | -1.89 | 2.97E-12 | 4.22E-10 | 9.374688 | -1.89 | down |
| chr3 | ROBO2 | 77281717 | 77282117 | 401 | * | 3.35 | 0 | 4.28 | -3.38 | 3.4E-06 | 0.00012 | 3.920819 | -3.38 | down |

| | | | | | | | | | | | | | | |
|---|---|---|---|---|---|---|---|---|---|---|---|---|---|---|
| chr3 | ROBO2 | 77255205 | 77255605 | 401 | * | 4.23 | 1.98 | 5.06 | -2.88 | 9.41E-10 | 8.38E-08 | 7.076756 | -2.88 | down |
| chr3 | ROBO2 | 77236096 | 77236496 | 401 | * | 5.67 | 1.03 | 6.64 | -5.25 | 2.29E-20 | 9.82E-18 | 17.00789 | -5.25 | down |
| chr3 | ROBO2 | 77221050 | 77221450 | 401 | * | 2.67 | 0 | 3.63 | -2.6 | 6.94E-05 | 0.00151 | 2.821023 | -2.6 | down |
| chr3 | ROBO2 | 77179741 | 77180141 | 401 | * | 6.84 | 2.11 | 7.81 | -5.54 | 3.68E-43 | 8.15E-40 | 39.08884 | -5.54 | down |
| chr3 | ROBO2 | 77171032 | 77171432 | 401 | * | 3.68 | 0 | 4.68 | -5.47 | 5.61E-09 | 4.19E-07 | 6.377786 | -5.47 | down |
| chr3 | ROBO2 | 77159159 | 77159559 | 401 | * | 3.6 | 0 | 4.58 | -4.44 | 7.41E-07 | 3.21E-05 | 4.493495 | -4.44 | down |
| chr3 | ROBO2 | 77146036 | 77146436 | 401 | * | 3.37 | 0.68 | 4.25 | -2.94 | 2.83E-06 | 0.000103 | 3.987163 | -2.94 | down |
| chr3 | ROBO2 | 77142624 | 77143024 | 401 | * | 3.3 | 0.68 | 4.18 | -2.8 | 6.4E-06 | 0.000206 | 3.686133 | -2.8 | down |
| chr3 | ROBO2 | 77101979 | 77102379 | 401 | * | 2.91 | 0 | 3.81 | -2.4 | 0.000068 | 0.00148 | 2.829738 | -2.4 | down |
| chr3 | ROBO2 | 77097147 | 77097547 | 401 | * | 3.18 | 0.94 | 4.02 | -2.2 | 0.000038 | 0.000912 | 3.040005 | -2.2 | down |
| chr3 | ROBO2 | 77081341 | 77081741 | 401 | * | 4.34 | 0.97 | 5.27 | -3.9 | 2.93E-11 | 3.50E-09 | 8.455932 | -3.9 | down |
| chr3 | ROBO2 | 77080257 | 77080657 | 401 | * | 4.08 | 0 | 5.06 | -4.49 | 9.4E-08 | 5.23E-06 | 5.281498 | -4.49 | down |
| chr3 | ROBO2 | 77068333 | 77068733 | 401 | * | 4.17 | 0.72 | 5.1 | -3.92 | 4.79E-10 | 4.49E-08 | 7.347754 | -3.92 | down |
| chr3 | ROBO2 | 77039783 | 77040183 | 401 | * | 5.6 | 1.86 | 6.55 | -4.48 | 1.25E-23 | 7.77E-21 | 20.10958 | -4.48 | down |
| chr3 | ROBO2 | 77038425 | 77038825 | 401 | * | 5.8 | 2.53 | 6.72 | -4.03 | 2.42E-28 | 2.03E-25 | 24.6925 | -4.03 | down |
| chr4 | ART3 | 76989280 | 76989680 | 401 | * | 3.87 | 2.78 | 4.48 | -1.06 | 0.000178 | 0.00326 | 2.486782 | -1.06 | down |
| chr6 | IMPG1 | 76770188 | 76770588 | 401 | * | 5.54 | 4.16 | 6.23 | -1.99 | 6.09E-15 | 1.30E-12 | 11.88606 | -1.99 | down |
| chr17 | DNAH17-AS1 | 76515207 | 76515607 | 401 | * | 4.83 | 3.87 | 5.4 | -1.19 | 1.94E-06 | 7.36E-05 | 4.133122 | -1.19 | down |
| chr2 | GCFC2 | 76120408 | 76120808 | 401 | * | 3.92 | 2.8 | 4.55 | -1.15 | 0.000102 | 0.00208 | 2.681937 | -1.15 | down |
| chr3 | MIR1324 | 75672380 | 75672780 | 401 | * | 6.5 | 5.04 | 7.21 | -2.14 | 3.57E-29 | 3.20E-26 | 25.49485 | -2.14 | down |
| chr3 | MIR1324 | 75658620 | 75659020 | 401 | * | 3.79 | 2.29 | 4.5 | -1.72 | 0.00001 | 0.000299 | 3.524329 | -1.72 | down |
| chr9 | ALDH1A1 | 75648000 | 75648400 | 401 | * | 4.13 | 3.07 | 4.73 | -1.11 | 6.67E-05 | 0.00146 | 2.835647 | -1.11 | down |
| chr3 | MIR1324 | 75641007 | 75641407 | 401 | * | 5.03 | 3.59 | 5.74 | -2.04 | 1.54E-11 | 1.95E-09 | 8.709965 | -2.04 | down |
| chr4 | BTC | 75630381 | 75630781 | 401 | * | 7.26 | 6.35 | 7.81 | -1.4 | 1.54E-25 | 1.10E-22 | 21.95861 | -1.4 | down |
| chr9 | ALDH1A1 | 75607497 | 75607897 | 401 | * | 5.26 | 4.46 | 5.78 | -1.05 | 1.21E-06 | 0.000049 | 4.309804 | -1.05 | down |
| chr15 | GOLGA6C | 75559373 | 75559773 | 401 | * | 5.17 | 3.54 | 5.92 | -2.28 | 5.28E-14 | 9.90E-12 | 11.00436 | -2.28 | down |
| chr12 | KCNC2 | 75480645 | 75481045 | 401 | * | 4.17 | 2.93 | 4.82 | -1.4 | 1.05E-05 | 0.000311 | 3.50724 | -1.4 | down |
| chr14 | YLPM1 | 75249442 | 75249842 | 401 | * | 3.83 | 2.28 | 4.57 | -1.82 | 6.71E-06 | 0.000214 | 3.669586 | -1.82 | down |

| | | | | | | | | | | | | | | |
|---|---|---|---|---|---|---|---|---|---|---|---|---|---|---|
| chr16 | LDHD | 75116583 | 75116983 | 401 | * | 6.78 | 5.72 | 7.38 | -1.6 | 5.01E-23 | 2.97E-20 | 19.52724 | -1.6 | down |
| chr17 | SNHG20 | 75077412 | 75077812 | 401 | * | 5.75 | 4.28 | 6.46 | -2.11 | 3.22E-17 | 9.50E-15 | 14.02228 | -2.11 | down |
| chr11 | SPCS2 | 74673151 | 74673551 | 401 | * | 4.42 | 2.53 | 5.21 | -2.5 | 2.12E-09 | 1.74E-07 | 6.759451 | -2.5 | down |
| chr14 | LIN52 | 74602081 | 74602481 | 401 | * | 4.9 | 3.85 | 5.5 | -1.36 | 2.71E-07 | 1.33E-05 | 4.876148 | -1.36 | down |
| chr4 | RASSF6 | 74433632 | 74434032 | 401 | * | 3.9 | 2.76 | 4.53 | -1.13 | 0.000106 | 0.00215 | 2.667562 | -1.13 | down |
| chr16 | LINC01568 | 73396636 | 73397036 | 401 | * | 6.45 | 5.4 | 7.06 | -1.59 | 1.41E-18 | 4.89E-16 | 15.31069 | -1.59 | down |
| chr10 | CDH23 | 73358616 | 73359016 | 401 | * | 6.28 | 5.51 | 6.78 | -1.14 | 3.26E-11 | 3.85E-09 | 8.414539 | -1.14 | down |
| chr11 | FAM168A | 73309134 | 73309534 | 401 | * | 5.44 | 4.3 | 6.07 | -1.62 | 2.30E-11 | 2.80E-09 | 8.552842 | -1.62 | down |
| chr2 | SFXN5 | 73285148 | 73285548 | 401 | * | 5.4 | 4.58 | 5.92 | -1.11 | 1.9E-07 | 9.73E-06 | 5.011887 | -1.11 | down |
| chr3 | EBLN2 | 73255543 | 73255943 | 401 | * | 5.04 | 3.96 | 5.64 | -1.43 | 5.09E-08 | 3.03E-06 | 5.518557 | -1.43 | down |
| chr15 | NEO1 | 73225373 | 73225773 | 401 | * | 5.37 | 4.48 | 5.91 | -1.19 | 6.09E-08 | 3.55E-06 | 5.449772 | -1.19 | down |
| chr11 | RELT | 73205728 | 73206128 | 401 | * | 4.43 | 3 | 5.13 | -1.87 | 8.27E-08 | 4.66E-06 | 5.331614 | -1.87 | down |
| chr9 | KLF9 | 73172209 | 73172609 | 401 | * | 4.03 | 2.96 | 4.64 | -1.11 | 8.94E-05 | 0.00187 | 2.728158 | -1.11 | down |
| chr3 | EBLN2 | 73094005 | 73094405 | 401 | * | 3.94 | 2.82 | 4.56 | -1.11 | 0.000119 | 0.00236 | 2.627088 | -1.11 | down |
| chr15 | ADPGK | 73046170 | 73046570 | 401 | * | 3.85 | 2.68 | 4.49 | -1.14 | 0.000117 | 0.00232 | 2.634512 | -1.14 | down |
| chr11 | FCHSD2 | 72821991 | 72822391 | 401 | * | 5.37 | 2.05 | 6.29 | -4.02 | 2.03E-21 | 1.06E-18 | 17.97469 | -4.02 | down |
| chr2 | EXOC6B | 72757244 | 72757644 | 401 | * | 4.87 | 3.77 | 5.49 | -1.43 | 1.92E-07 | 9.77E-06 | 5.010105 | -1.43 | down |
| chr11 | ATG16L2 | 72613969 | 72614369 | 401 | * | 5.87 | 5.06 | 6.38 | -1.16 | 1.37E-09 | 1.16E-07 | 6.935542 | -1.16 | down |
| chr12 | TRHDE | 72578224 | 72578624 | 401 | * | 6.12 | 5.13 | 6.71 | -1.46 | 1.69E-14 | 3.39E-12 | 11.4698 | -1.46 | down |
| chr4 | GC | 72513224 | 72513624 | 401 | * | 3.85 | 2.68 | 4.49 | -1.16 | 0.000108 | 0.00218 | 2.661544 | -1.16 | down |
| chr15 | NR2E3 | 72102288 | 72102688 | 401 | * | 3.64 | 2.45 | 4.28 | -1.03 | 0.00033 | 0.00532 | 2.274088 | -1.03 | down |
| chr15 | NR2E3 | 72096788 | 72097188 | 401 | * | 4.41 | 3.33 | 5.03 | -1.29 | 5.22E-06 | 0.000173 | 3.761954 | -1.29 | down |
| chr5 | TNPO1 | 72015118 | 72015518 | 401 | * | 4.91 | 4.01 | 5.46 | -1.14 | 2.51E-06 | 0.000092 | 4.036212 | -1.14 | down |
| chr4 | DCK | 71905487 | 71905887 | 401 | * | 5.47 | 4.65 | 5.99 | -1.11 | 1.64E-07 | 8.55E-06 | 5.068034 | -1.11 | down |
| chr17 | LINC00469 | 71841395 | 71841795 | 401 | * | 5.46 | 3.17 | 6.3 | -3.03 | 2.99E-21 | 1.50E-18 | 17.82391 | -3.03 | down |
| chr2 | PAIP2B | 71466606 | 71467006 | 401 | * | 4.86 | 4 | 5.39 | -1.07 | 6.48E-06 | 0.000208 | 3.681937 | -1.07 | down |
| chr17 | SLC39A11 | 71071812 | 71072212 | 401 | * | 4.96 | 2.49 | 5.83 | -3.21 | 1.37E-15 | 3.21E-13 | 12.49349 | -3.21 | down |
| chr2 | CLEC4F | 71043957 | 71044357 | 401 | * | 4.22 | 3.19 | 4.82 | -1.13 | 4.48E-05 | 0.00105 | 2.978811 | -1.13 | down |

| | | | | | | | | | | | | | | |
|---|---|---|---|---|---|---|---|---|---|---|---|---|---|---|
| chr17 | SLC39A11 | 70876010 | 70876410 | 401 | * | 6.69 | 2.61 | 7.64 | -4.88 | 9.72E-46 | 2.69E-42 | 41.57025 | -4.88 | down |
| chr17 | LINC00511 | 70825391 | 70825791 | 401 | * | 3.6 | 0 | 4.56 | -3.92 | 1.72E-06 | 6.67E-05 | 4.175874 | -3.92 | down |
| chr15 | TLE3 | 70633482 | 70633882 | 401 | * | 3.9 | 2.76 | 4.53 | -1.13 | 0.000109 | 0.0022 | 2.657577 | -1.13 | down |
| chr15 | TLE3 | 70463029 | 70463429 | 401 | * | 7.72 | 6.66 | 8.33 | -1.64 | 1.01E-43 | 2.40E-40 | 39.61979 | -1.64 | down |
| chr15 | TLE3 | 70428998 | 70429398 | 401 | * | 5.52 | 4.49 | 6.12 | -1.45 | 1.79E-10 | 1.85E-08 | 7.732828 | -1.45 | down |
| chr11 | SHANK2-AS1 | 70419941 | 70420341 | 401 | * | 6.43 | 5.21 | 7.08 | -1.73 | 9.01E-11 | 9.89E-09 | 8.004804 | -1.73 | down |
| chr15 | LINC00593 | 70171538 | 70171938 | 401 | * | 4.93 | 4.06 | 5.47 | -1.1 | 2.93E-06 | 0.000106 | 3.974694 | -1.1 | down |
| chr17 | SOX9 | 70169235 | 70169635 | 401 | * | 5.06 | 2.7 | 5.91 | -3.08 | 8.34E-17 | 2.33E-14 | 13.63264 | -3.08 | down |
| chr17 | SOX9 | 70059014 | 70059414 | 401 | * | 4.96 | 2.16 | 5.85 | -3.49 | 1.53E-16 | 4.16E-14 | 13.38091 | -3.49 | down |
| chr17 | SOX9 | 69857404 | 69857804 | 401 | * | 3.84 | 2.09 | 4.6 | -2.17 | 1.23E-06 | 4.99E-05 | 4.301899 | -2.17 | down |
| chr3 | MITF | 69784990 | 69785390 | 401 | * | 4.66 | 3.62 | 5.26 | -1.29 | 2.07E-06 | 7.78E-05 | 4.10902 | -1.29 | down |
| chr4 | TMPRSS11E | 69309458 | 69309858 | 401 | * | 6.44 | 5.71 | 6.92 | -1.1 | 3.82E-11 | 4.47E-09 | 8.349692 | -1.1 | down |
| chr2 | GKN2 | 69178280 | 69178680 | 401 | * | 5.3 | 4.25 | 5.91 | -1.45 | 3.04E-09 | 2.42E-07 | 6.616185 | -1.45 | down |
| chr11 | MYEOV | 69089034 | 69089434 | 401 | * | 6.16 | 5.35 | 6.68 | -1.2 | 1.97E-11 | 2.45E-09 | 8.610834 | -1.2 | down |
| chr9 | FRG1HP | 68861374 | 68861774 | 401 | * | 5.64 | 4.07 | 6.38 | -2.27 | 4.14E-18 | 1.39E-15 | 14.85699 | -2.27 | down |
| chr2 | APLF | 68774654 | 68775054 | 401 | * | 5.57 | 4.59 | 6.15 | -1.39 | 2.45E-10 | 2.44E-08 | 7.61261 | -1.39 | down |
| chr15 | ITGA11 | 68691684 | 68692084 | 401 | * | 5.15 | 3.83 | 5.83 | -1.9 | 4.59E-11 | 5.26E-09 | 8.279014 | -1.9 | down |
| chr15 | ITGA11 | 68675999 | 68676399 | 401 | * | 5.34 | 4.57 | 5.84 | -1.02 | 1.19E-06 | 4.86E-05 | 4.313364 | -1.02 | down |
| chr2 | PLEK | 68629059 | 68629459 | 401 | * | 4.15 | 3.02 | 4.77 | -1.27 | 2.05E-05 | 0.000547 | 3.262013 | -1.27 | down |
| chr16 | SMPD3 | 68470548 | 68470948 | 401 | * | 4.7 | 3.83 | 5.24 | -1.04 | 1.82E-05 | 0.000497 | 3.303644 | -1.04 | down |
| chr15 | PIAS1 | 68257041 | 68257441 | 401 | * | 5.15 | 2.5 | 6.03 | -3.41 | 1.88E-18 | 6.49E-16 | 15.18776 | -3.41 | down |
| chr18 | RTTN | 67790781 | 67791181 | 401 | * | 6.23 | 1.8 | 7.2 | -5.13 | 1.47E-31 | 1.55E-28 | 27.80967 | -5.13 | down |
| chr18 | RTTN | 67783556 | 67783956 | 401 | * | 5.4 | 1.7 | 6.34 | -4.39 | 4.93E-21 | 2.37E-18 | 17.62525 | -4.39 | down |
| chr11 | UNC93B1 | 67699533 | 67699933 | 401 | * | 4.63 | 3.55 | 5.24 | -1.35 | 1.23E-06 | 4.98E-05 | 4.302771 | -1.35 | down |
| chr1 | SLC35D1 | 67513835 | 67514235 | 401 | * | 5.96 | 4.97 | 6.54 | -1.43 | 5.56E-13 | 8.98E-11 | 10.04672 | -1.43 | down |
| chr18 | CD226 | 67388755 | 67389155 | 401 | * | 4.61 | 3 | 5.35 | -2.17 | 1.85E-09 | 1.53E-07 | 6.815309 | -2.17 | down |
| chr18 | CD226 | 67385442 | 67385842 | 401 | * | 5.36 | 4.25 | 5.98 | -1.56 | 2.05E-10 | 2.08E-08 | 7.681937 | -1.56 | down |
| chr18 | CD226 | 67360127 | 67360527 | 401 | * | 7.12 | 6.12 | 7.7 | -1.54 | 2.17E-27 | 1.76E-24 | 23.75449 | -1.54 | down |

| | | | | | | | | | | | | | | |
|---|---|---|---|---|---|---|---|---|---|---|---|---|---|---|
| chr18 | CD226 | 67351100 | 67351500 | 401 | * | 3.99 | 1.7 | 4.84 | -2.87 | 1.57E-08 | 1.06E-06 | 5.974694 | -2.87 | down |
| chr18 | DOK6 | 67335530 | 67335930 | 401 | * | 4.81 | 3.94 | 5.35 | -1.06 | 0.000012 | 0.000349 | 3.457175 | -1.06 | down |
| chr18 | DOK6 | 67265634 | 67266034 | 401 | * | 3.57 | 2.11 | 4.28 | -1.5 | 5.93E-05 | 0.00132 | 2.879426 | -1.5 | down |
| chr15 | SMAD6 | 66938057 | 66938457 | 401 | * | 5.51 | 4.55 | 6.07 | -1.33 | 1.51E-09 | 1.27E-07 | 6.896196 | -1.33 | down |
| chr8 | DNAJC5B | 66870586 | 66870986 | 401 | * | 3.9 | 2.44 | 4.61 | -1.68 | 8.32E-06 | 0.000255 | 3.59346 | -1.68 | down |
| chr11 | PC | 66727393 | 66727793 | 401 | * | 5.07 | 4.07 | 5.66 | -1.3 | 2.6E-07 | 1.29E-05 | 4.88941 | -1.3 | down |
| chr11 | PC | 66720203 | 66720603 | 401 | * | 5.4 | 2.92 | 6.26 | -3.25 | 3.87E-21 | 1.91E-18 | 17.71897 | -3.25 | down |
| chr6 | SLC25A51P1 | 66522842 | 66523242 | 401 | * | 3.3 | 0.68 | 4.17 | -2.82 | 5.16E-06 | 0.000171 | 3.767004 | -2.82 | down |
| chr7 | TMEM248 | 66413496 | 66413896 | 401 | * | 6.8 | 6.05 | 7.29 | -1.15 | 2.50E-15 | 5.61E-13 | 12.25104 | -1.15 | down |
| chr5 | MAST4 | 65976509 | 65976909 | 401 | * | 4.56 | 3.41 | 5.2 | -1.45 | 7.48E-07 | 3.23E-05 | 4.490797 | -1.45 | down |
| chr15 | HACD3 | 65832710 | 65833110 | 401 | * | 4.6 | 3.73 | 5.14 | -1.01 | 3.38E-05 | 0.000826 | 3.08302 | -1.01 | down |
| chr6 | EYS | 65646525 | 65646925 | 401 | * | 4.53 | 3.55 | 5.11 | -1.16 | 1.14E-05 | 0.000335 | 3.474955 | -1.16 | down |
| chr13 | OR7E156P | 65623094 | 65623494 | 401 | * | 4.38 | 3.06 | 5.06 | -1.67 | 4.41E-07 | 2.04E-05 | 4.69037 | -1.67 | down |
| chr6 | EYS | 65609014 | 65609414 | 401 | * | 5.2 | 3.35 | 5.99 | -2.53 | 2.41E-15 | 5.45E-13 | 12.2636 | -2.53 | down |
| chr6 | EYS | 65595892 | 65596292 | 401 | * | 4.96 | 2.92 | 5.78 | -2.76 | 2.17E-14 | 4.29E-12 | 11.36754 | -2.76 | down |
| chr13 | OR7E156P | 65595854 | 65596254 | 401 | * | 4.65 | 1.94 | 5.54 | -3.36 | 2.40E-13 | 4.07E-11 | 10.39041 | -3.36 | down |
| chr14 | CHURC1-FNTB | 65496606 | 65497006 | 401 | * | 3.85 | 2.52 | 4.53 | -1.39 | 4.11E-05 | 0.000975 | 3.010995 | -1.39 | down |
| chr13 | OR7E156P | 65468572 | 65468972 | 401 | * | 5.87 | 4.49 | 6.56 | -2.02 | 2.71E-18 | 9.12E-16 | 15.04001 | -2.02 | down |
| chr6 | EYS | 65461169 | 65461569 | 401 | * | 3.81 | 0 | 4.77 | -4.19 | 4.83E-07 | 2.21E-05 | 4.655608 | -4.19 | down |
| chr1 | JAK1 | 65422634 | 65423034 | 401 | * | 6.81 | 5.65 | 7.45 | -1.75 | 4.83E-27 | 3.77E-24 | 23.42366 | -1.75 | down |
| chr10 | REEP3 | 65394914 | 65395314 | 401 | * | 5.16 | 4.22 | 5.72 | -1.26 | 9.37E-08 | 5.22E-06 | 5.282329 | -1.26 | down |
| chr10 | REEP3 | 65341424 | 65341824 | 401 | * | 3.4 | 2.07 | 4.08 | -1.08 | 0.000403 | 0.00626 | 2.203426 | -1.08 | down |
| chr1 | RAVER2 | 65264410 | 65264810 | 401 | * | 5.58 | 4.77 | 6.1 | -1.12 | 4.23E-08 | 2.56E-06 | 5.59176 | -1.12 | down |
| chr3 | MIR548A2 | 65013057 | 65013457 | 401 | * | 4.8 | 3.8 | 5.38 | -1.26 | 8.8E-07 | 3.73E-05 | 4.428291 | -1.26 | down |
| chr2 | SERTAD2 | 64915125 | 64915525 | 401 | * | 5.5 | 4.69 | 6.01 | -1.09 | 2.22E-07 | 1.11E-05 | 4.954677 | -1.09 | down |
| chr1 | UBE2U | 64757234 | 64757634 | 401 | * | 4.08 | 3.02 | 4.69 | -1.08 | 0.000102 | 0.00209 | 2.679854 | -1.08 | down |
| chr1 | UBE2U | 64745811 | 64746211 | 401 | * | 4.8 | 3.42 | 5.49 | -1.89 | 3.67E-09 | 2.87E-07 | 6.542118 | -1.89 | down |
| chr3 | ADAMTS9 | 64686005 | 64686405 | 401 | * | 4.84 | 3.5 | 5.53 | -1.85 | 1.60E-09 | 1.34E-07 | 6.872895 | -1.85 | down |

| | | | | | | | | | | | | | | |
|---|---|---|---|---|---|---|---|---|---|---|---|---|---|---|
| chr15 | CSNK1G1 | 64634010 | 64634410 | 401 | * | 6.56 | 5.88 | 7.02 | -1.02 | 2.80E-11 | 3.36E-09 | 8.473661 | -1.02 | down |
| chr4 | TECRL | 64461690 | 64462090 | 401 | * | 4.12 | 0 | 5.07 | -4.3 | 8.54E-09 | 6.11E-07 | 6.213959 | -4.3 | down |
| chr4 | TECRL | 64437592 | 64437992 | 401 | * | 4.78 | 0.72 | 5.73 | -4.54 | 2.45E-13 | 4.14E-11 | 10.383 | -4.54 | down |
| chr13 | OR7E156P | 64274354 | 64274754 | 401 | * | 6.26 | 4.4 | 7.05 | -2.61 | 1.60E-30 | 1.52E-27 | 26.81816 | -2.61 | down |
| chr4 | TECRL | 64271643 | 64272043 | 401 | * | 3.75 | 0.72 | 4.66 | -3.46 | 5.69E-08 | 3.34E-06 | 5.476254 | -3.46 | down |
| chr14 | SGPP1 | 64217728 | 64218128 | 401 | * | 5.25 | 4.42 | 5.77 | -1.08 | 1.4E-06 | 5.57E-05 | 4.254145 | -1.08 | down |
| chr11 | DNAJC4 | 63999356 | 63999756 | 401 | * | 5.08 | 4.21 | 5.62 | -1.12 | 1.56E-06 | 6.13E-05 | 4.21254 | -1.12 | down |
| chr11 | TRPT1 | 63986216 | 63986616 | 401 | * | 7.09 | 6.44 | 7.54 | -1.02 | 1.63E-14 | 3.27E-12 | 11.48545 | -1.02 | down |
| chr11 | MACROD1 | 63908496 | 63908896 | 401 | * | 5.99 | 5.29 | 6.45 | -1.01 | 1.43E-08 | 9.81E-07 | 6.008331 | -1.01 | down |
| chr16 | CDH11 | 63760361 | 63760761 | 401 | * | 2.88 | 0 | 3.79 | -2.37 | 7.33E-05 | 0.00158 | 2.801343 | -2.37 | down |
| chr18 | CDH7 | 63457958 | 63458358 | 401 | * | 4.49 | 3.53 | 5.06 | -1.1 | 2.48E-05 | 0.000642 | 3.192465 | -1.1 | down |
| chr16 | CDH8 | 63312431 | 63312831 | 401 | * | 3.03 | 1.37 | 3.79 | -1.21 | 0.000445 | 0.00675 | 2.170696 | -1.21 | down |
| chr15 | TPM1 | 63226693 | 63227093 | 401 | * | 3.99 | 2.95 | 4.59 | -1.03 | 0.000159 | 0.00297 | 2.527244 | -1.03 | down |
| chr2 | EHBP1 | 63048546 | 63048946 | 401 | * | 5.3 | 4.22 | 5.91 | -1.46 | 7.30E-09 | 5.3E-07 | 6.275724 | -1.46 | down |
| chr12 | LINC01465 | 62983657 | 62984057 | 401 | * | 3.99 | 2.96 | 4.59 | -1.02 | 0.000176 | 0.00323 | 2.490797 | -1.02 | down |
| chr20 | C20orf204 | 62651190 | 62651590 | 401 | * | 5.15 | 4.35 | 5.66 | -1.03 | 4.1E-06 | 0.000141 | 3.850781 | -1.03 | down |
| chr8 | ASPH | 62623992 | 62624392 | 401 | * | 6.13 | 5.2 | 6.69 | -1.38 | 2.63E-13 | 4.42E-11 | 10.35458 | -1.38 | down |
| chr20 | DNAJC5 | 62530679 | 62531079 | 401 | * | 6.26 | 5.38 | 6.81 | -1.31 | 1.60E-13 | 2.76E-11 | 10.55909 | -1.31 | down |
| chr10 | ANK3 | 62421968 | 62422368 | 401 | * | 4.22 | 3.24 | 4.8 | -1.04 | 0.000102 | 0.00208 | 2.681937 | -1.04 | down |
| chr10 | ANK3 | 62388392 | 62388792 | 401 | * | 5.81 | 4.42 | 6.51 | -2.03 | 7.83E-18 | 2.52E-15 | 14.5986 | -2.03 | down |
| chr11 | MYRF | 61499088 | 61499488 | 401 | * | 3.81 | 2.69 | 4.44 | -1.04 | 0.000243 | 0.00417 | 2.379864 | -1.04 | down |
| chr2 | USP34 | 61460140 | 61460540 | 401 | * | 4.26 | 3.17 | 4.87 | -1.23 | 1.76E-05 | 0.000482 | 3.316953 | -1.23 | down |
| chr16 | CDH8 | 61349024 | 61349424 | 401 | * | 5.03 | 2.73 | 5.87 | -3.05 | 2.95E-16 | 7.63E-14 | 13.11748 | -3.05 | down |
| chr16 | CDH8 | 61347124 | 61347524 | 401 | * | 4.59 | 2.09 | 5.46 | -3.18 | 1.18E-12 | 1.79E-10 | 9.747147 | -3.18 | down |
| chr16 | CDH8 | 61340816 | 61341216 | 401 | * | 3.13 | 0.32 | 4.02 | -2.62 | 2.59E-05 | 0.000664 | 3.177832 | -2.62 | down |
| chr16 | CDH8 | 61332728 | 61333128 | 401 | * | 2.84 | 0.96 | 3.63 | -1.16 | 0.000699 | 0.00975 | 2.010995 | -1.16 | down |
| chr16 | CDH8 | 61323442 | 61323842 | 401 | * | 4.07 | 3 | 4.68 | -1.1 | 9.38E-05 | 0.00194 | 2.712198 | -1.1 | down |
| chr2 | REL | 61144836 | 61145236 | 401 | * | 6.35 | 5.6 | 6.84 | -1.11 | 2.64E-11 | 3.18E-09 | 8.497573 | -1.11 | down |

| | | | | | | | | | | | | | | |
|---|---|---|---|---|---|---|---|---|---|---|---|---|---|---|
| chr16 | CDH8 | 61068745 | 61069145 | 401 | * | 4.49 | 3.01 | 5.21 | -1.99 | 1.86E-08 | 1.23E-06 | 5.910095 | -1.99 | down |
| chr8 | CA8 | 60950966 | 60951366 | 401 | * | 6.62 | 5.88 | 7.1 | -1.12 | 4.99E-13 | 8.08E-11 | 10.09259 | -1.12 | down |
| chr20 | CDH4 | 60209064 | 60209464 | 401 | * | 5.63 | 4.83 | 6.13 | -1.1 | 3.7E-08 | 2.27E-06 | 5.643974 | -1.1 | down |
| chr20 | CDH4 | 59835768 | 59836168 | 401 | * | 3.84 | 2.64 | 4.49 | -1.24 | 6.96E-05 | 0.00151 | 2.821023 | -1.24 | down |
| chr13 | DIAPH3 | 59743185 | 59743585 | 401 | * | 4.48 | 3.48 | 5.06 | -1.19 | 9.7E-06 | 0.000291 | 3.536107 | -1.19 | down |
| chr13 | DIAPH3 | 59356682 | 59357082 | 401 | * | 4.24 | 3.14 | 4.86 | -1.25 | 1.81E-05 | 0.000493 | 3.307153 | -1.25 | down |
| chr18 | CDH20 | 58859089 | 58859489 | 401 | * | 4.43 | 3.4 | 5.03 | -1.24 | 8.76E-06 | 0.000267 | 3.573489 | -1.24 | down |
| chr18 | CDH20 | 58829951 | 58830351 | 401 | * | 5.01 | 4.2 | 5.53 | -1.01 | 0.000008 | 0.000247 | 3.607303 | -1.01 | down |
| chr12 | ATP23 | 58651774 | 58652174 | 401 | * | 4.14 | 2.89 | 4.8 | -1.46 | 7.97E-06 | 0.000246 | 3.609065 | -1.46 | down |
| chr15 | AQP9 | 58399796 | 58400196 | 401 | * | 5.13 | 4.06 | 5.74 | -1.45 | 1.06E-08 | 7.43E-07 | 6.129011 | -1.45 | down |
| chr3 | PXK | 58351930 | 58352330 | 401 | * | 5.82 | 4.94 | 6.37 | -1.26 | 1.69E-10 | 1.77E-08 | 7.752027 | -1.26 | down |
| chr20 | LOC100506384 | 58194116 | 58194516 | 401 | * | 4.38 | 3.37 | 4.97 | -1.14 | 0.000026 | 0.000668 | 3.175224 | -1.14 | down |
| chr15 | POLR2M | 58111261 | 58111661 | 401 | * | 5.66 | 4.4 | 6.32 | -1.83 | 2.71E-14 | 5.31E-12 | 11.27491 | -1.83 | down |
| chr19 | ZIK1 | 58098178 | 58098578 | 401 | * | 3.77 | 2.63 | 4.4 | -1.04 | 0.000268 | 0.0045 | 2.346787 | -1.04 | down |
| chr3 | DENND6A | 57671305 | 57671705 | 401 | * | 4.32 | 3.01 | 5 | -1.66 | 6.64E-07 | 2.91E-05 | 4.536107 | -1.66 | down |
| chr4 | SPINK2 | 57647169 | 57647569 | 401 | * | 3.51 | 2.16 | 4.2 | -1.21 | 0.000187 | 0.00339 | 2.4698 | -1.21 | down |
| chr4 | HOPX | 57591066 | 57591466 | 401 | * | 3.97 | 2.38 | 4.71 | -1.98 | 1.18E-06 | 4.81E-05 | 4.317855 | -1.98 | down |
| chr12 | NEMP1 | 57476199 | 57476599 | 401 | * | 4.69 | 3.81 | 5.23 | -1.06 | 1.55E-05 | 0.000433 | 3.363512 | -1.06 | down |
| chr17 | SMG8 | 57285206 | 57285606 | 401 | * | 5.13 | 4.27 | 5.66 | -1.1 | 1.71E-06 | 6.62E-05 | 4.179142 | -1.1 | down |
| chr12 | MIP | 56853528 | 56853928 | 401 | * | 6.86 | 6.19 | 7.31 | -1.03 | 4.96E-13 | 8.04E-11 | 10.09474 | -1.03 | down |
| chr19 | ZNF787 | 56595033 | 56595433 | 401 | * | 4.99 | 4.02 | 5.56 | -1.25 | 3.54E-07 | 1.68E-05 | 4.774691 | -1.25 | down |
| chr19 | NLRP5 | 56567294 | 56567694 | 401 | * | 6.12 | 4.87 | 6.78 | -1.85 | 8.88E-19 | 3.19E-16 | 15.49621 | -1.85 | down |
| chr19 | NLRP8 | 56477910 | 56478310 | 401 | * | 6.71 | 6 | 7.18 | -1.08 | 9.10E-13 | 1.42E-10 | 9.847712 | -1.08 | down |
| chr16 | AMFR | 56438020 | 56438420 | 401 | * | 7.07 | 6.39 | 7.53 | -1.06 | 1.12E-15 | 2.63E-13 | 12.58004 | -1.06 | down |
| chr20 | NA | 56429446 | 56429846 | 401 | * | 6.71 | 5.85 | 7.24 | -1.31 | 1.69E-17 | 5.23E-15 | 14.2815 | -1.31 | down |
| chr8 | XKR4 | 55844131 | 55844531 | 401 | * | 6.22 | 4.72 | 6.94 | -2.19 | 4.89E-25 | 3.34E-22 | 21.47625 | -2.19 | down |
| chr8 | RP1 | 55723826 | 55724226 | 401 | * | 6.38 | 5.66 | 6.85 | -1.07 | 1.05E-10 | 1.14E-08 | 7.943095 | -1.07 | down |
| chrX | RRAGB | 55702749 | 55703149 | 401 | * | 4.67 | 3.63 | 5.27 | -1.3 | 1.48E-06 | 5.87E-05 | 4.231362 | -1.3 | down |

| | | | | | | | | | | | | | | |
|---|---|---|---|---|---|---|---|---|---|---|---|---|---|---|
| chr10 | PCDH15 | 55631159 | 55631559 | 401 | * | 3.81 | 1.68 | 4.63 | -2.62 | 2.54E-07 | 1.26E-05 | 4.899629 | -2.62 | down |
| chr15 | RSL24D1 | 55457411 | 55457811 | 401 | * | 4.84 | 3.84 | 5.42 | -1.28 | 6.35E-07 | 0.000028 | 4.552842 | -1.28 | down |
| chr19 | NLRP7 | 55440665 | 55441065 | 401 | * | 6.1 | 5.12 | 6.68 | -1.45 | 3.50E-14 | 6.77E-12 | 11.16941 | -1.45 | down |
| chr10 | MBL2 | 55401228 | 55401628 | 401 | * | 4.2 | 0.97 | 5.12 | -3.76 | 1.93E-10 | 1.97E-08 | 7.705534 | -3.76 | down |
| chr10 | MBL2 | 55283027 | 55283427 | 401 | * | 3.9 | 1.21 | 4.79 | -3.24 | 1.34E-08 | 9.2E-07 | 6.036212 | -3.24 | down |
| chr10 | MBL2 | 55163618 | 55164018 | 401 | * | 3.61 | 1.15 | 4.47 | -2.85 | 7.5E-07 | 3.25E-05 | 4.488117 | -2.85 | down |
| chr4 | LNX1 | 54539542 | 54539942 | 401 | * | 5.57 | 3.94 | 6.32 | -2.3 | 2.53E-17 | 7.56E-15 | 14.12148 | -2.3 | down |
| chr5 | ESM1 | 54224027 | 54224427 | 401 | * | 6.61 | 5.9 | 7.09 | -1.08 | 3.97E-12 | 5.50E-10 | 9.259637 | -1.08 | down |
| chr13 | LINC00558 | 54029215 | 54029615 | 401 | * | 3.93 | 2.24 | 4.69 | -2.05 | 1.52E-06 | 6.01E-05 | 4.221126 | -2.05 | down |
| chr19 | ZNF813 | 53980188 | 53980588 | 401 | * | 5.03 | 3.96 | 5.63 | -1.43 | 3.92E-08 | 2.4E-06 | 5.619789 | -1.43 | down |
| chr20 | DOK5 | 53800781 | 53801181 | 401 | * | 4.53 | 3.62 | 5.09 | -1.06 | 2.42E-05 | 0.000628 | 3.20204 | -1.06 | down |
| chr18 | LINC01539 | 53796020 | 53796420 | 401 | * | 5.51 | 3.61 | 6.3 | -2.64 | 1.43E-19 | 5.58E-17 | 16.25337 | -2.64 | down |
| chr18 | LINC01539 | 53745594 | 53745994 | 401 | * | 3.64 | 0.41 | 4.56 | -3.56 | 1.9E-07 | 9.72E-06 | 5.012334 | -3.56 | down |
| chr20 | DOK5 | 53739289 | 53739689 | 401 | * | 4.59 | 3.23 | 5.28 | -1.82 | 2.41E-08 | 1.55E-06 | 5.809668 | -1.82 | down |
| chr18 | TCF4 | 53191843 | 53192243 | 401 | * | 4.59 | 0.97 | 5.53 | -4.15 | 1.16E-12 | 1.77E-10 | 9.752027 | -4.15 | down |
| chr14 | ERO1A | 53128628 | 53129028 | 401 | * | 6.89 | 6.17 | 7.38 | -1.12 | 2.50E-15 | 5.61E-13 | 12.25104 | -1.12 | down |
| chr14 | ERO1A | 53114056 | 53114456 | 401 | * | 7.09 | 6.37 | 7.56 | -1.12 | 5.16E-17 | 1.50E-14 | 13.82391 | -1.12 | down |
| chr4 | SPATA18 | 53039795 | 53040195 | 401 | * | 5.21 | 4.21 | 5.8 | -1.36 | 1.92E-08 | 1.26E-06 | 5.899629 | -1.36 | down |
| chr7 | POM121L12 | 52936752 | 52937152 | 401 | * | 6.01 | 4.81 | 6.66 | -1.78 | 9.53E-17 | 2.64E-14 | 13.5784 | -1.78 | down |
| chr18 | DYNAP | 52180997 | 52181397 | 401 | * | 3.48 | 1.86 | 4.22 | -1.71 | 3.71E-05 | 0.000895 | 3.048177 | -1.71 | down |
| chr19 | SIGLEC12 | 52008053 | 52008453 | 401 | * | 4.71 | 3.35 | 5.4 | -1.86 | 1.24E-08 | 8.59E-07 | 6.066007 | -1.86 | down |
| chr13 | INTS6 | 51978941 | 51979341 | 401 | * | 5.7 | 4.83 | 6.24 | -1.2 | 4.52E-09 | 3.46E-07 | 6.460924 | -1.2 | down |
| chrX | MAGED4 | 51893276 | 51893676 | 401 | * | 4.77 | 1.32 | 5.7 | -4.01 | 1.54E-14 | 3.16E-12 | 11.50031 | -4.01 | down |
| chr19 | LIM2 | 51887792 | 51888192 | 401 | * | 6.27 | 4.59 | 7.03 | -2.39 | 1.31E-27 | 1.07E-24 | 23.97062 | -2.39 | down |
| chrX | SNORA11D | 51803130 | 51803530 | 401 | * | 3.53 | 0 | 4.49 | -3.82 | 2.47E-06 | 9.11E-05 | 4.040482 | -3.82 | down |
| chr12 | METTL7A | 51318153 | 51318553 | 401 | * | 4.12 | 3.06 | 4.72 | -1.11 | 6.87E-05 | 0.0015 | 2.823909 | -1.11 | down |
| chr1 | FAF1 | 51307992 | 51308392 | 401 | * | 5.81 | 4.85 | 6.39 | -1.4 | 1.33E-11 | 1.69E-09 | 8.772113 | -1.4 | down |
| chr14 | NIN | 51219521 | 51219921 | 401 | * | 3.8 | 2.19 | 4.54 | -1.89 | 5.38E-06 | 0.000178 | 3.74958 | -1.89 | down |

| | | | | | | | | | | | | | | |
|---|---|---|---|---|---|---|---|---|---|---|---|---|---|---|
| chr15 | SPPL2A | 51077563 | 51077963 | 401 | * | 4.75 | 3.59 | 5.38 | -1.53 | 9.04E-08 | 5.05E-06 | 5.296709 | -1.53 | down |
| chr14 | MAP4K5 | 50944289 | 50944689 | 401 | * | 6.6 | 4.79 | 7.38 | -2.55 | 4.46E-37 | 7.22E-34 | 33.14146 | -2.55 | down |
| chr19 | MYH14 | 50728368 | 50728768 | 401 | * | 7.02 | 5.52 | 7.74 | -2.2 | 3.32E-40 | 5.96E-37 | 36.22475 | -2.2 | down |
| chr8 | SNTG1 | 50649252 | 50649652 | 401 | * | 3.69 | 1.88 | 4.47 | -2.2 | 3.19E-06 | 0.000113 | 3.946922 | -2.2 | down |
| chr8 | SNTG1 | 50643600 | 50644000 | 401 | * | 6.26 | 4.74 | 6.99 | -2.22 | 4.11E-26 | 3.03E-23 | 22.51856 | -2.22 | down |
| chr8 | SNTG1 | 50621106 | 50621506 | 401 | * | 5.89 | 4.99 | 6.43 | -1.29 | 4.67E-11 | 5.33E-09 | 8.273273 | -1.29 | down |
| chr8 | SNTG1 | 50617603 | 50618003 | 401 | * | 4.14 | 2.82 | 4.81 | -1.57 | 3.65E-06 | 0.000127 | 3.896196 | -1.57 | down |
| chr2 | NRXN1 | 50597142 | 50597542 | 401 | * | 4.43 | 2.92 | 5.15 | -2.03 | 3.11E-08 | 1.96E-06 | 5.707744 | -2.03 | down |
| chr10 | C10orf71 | 50527420 | 50527820 | 401 | * | 3.24 | 1.68 | 3.97 | -1.27 | 0.000277 | 0.00464 | 2.333482 | -1.27 | down |
| chr19 | SCAF1 | 50148099 | 50148499 | 401 | * | 6.71 | 5.96 | 7.21 | -1.15 | 1.57E-14 | 3.19E-12 | 11.49621 | -1.15 | down |
| chr19 | CCDC155 | 49889992 | 49890392 | 401 | * | 8.07 | 7.35 | 8.55 | -1.16 | 2.59E-31 | 2.65E-28 | 27.57675 | -1.16 | down |
| chr8 | SNAI2 | 49874823 | 49875223 | 401 | * | 3.87 | 2.78 | 4.49 | -1.05 | 0.00018 | 0.00329 | 2.482804 | -1.05 | down |
| chr3 | APEH | 49686678 | 49687078 | 401 | * | 6.13 | 4.86 | 6.8 | -1.74 | 2.79E-09 | 2.23E-07 | 6.651695 | -1.74 | down |
| chr17 | NME2 | 49260394 | 49260794 | 401 | * | 5.68 | 4.82 | 6.21 | -1.21 | 3.20E-09 | 2.54E-07 | 6.595166 | -1.21 | down |
| chr19 | MAMSTR | 49210097 | 49210497 | 401 | * | 6.52 | 5.79 | 7.01 | -1.1 | 3.01E-12 | 4.27E-10 | 9.369572 | -1.1 | down |
| chr19 | SEC1P | 49142840 | 49143240 | 401 | * | 7.48 | 6.75 | 7.97 | -1.16 | 2.51E-21 | 1.28E-18 | 17.89279 | -1.16 | down |
| chr18 | MEX3C | 48715650 | 48716050 | 401 | * | 6.01 | 5.25 | 6.51 | -1.11 | 1.33E-09 | 1.13E-07 | 6.946922 | -1.11 | down |
| chr4 | ZAR1 | 48491162 | 48491562 | 401 | * | 5 | 3.01 | 5.81 | -2.68 | 1.09E-14 | 2.29E-12 | 11.64016 | -2.68 | down |
| chrX | WDR13 | 48487434 | 48487834 | 401 | * | 6.31 | 5.3 | 6.9 | -1.51 | 2.82E-16 | 7.34E-14 | 13.1343 | -1.51 | down |
| chr15 | SEMA6D | 48177694 | 48178094 | 401 | * | 5.14 | 2.89 | 5.98 | -2.98 | 2.78E-17 | 8.27E-15 | 14.08249 | -2.98 | down |
| chr14 | MDGA2 | 47778643 | 47779043 | 401 | * | 4.67 | 1.41 | 5.59 | -3.9 | 1.01E-13 | 1.81E-11 | 10.74232 | -3.9 | down |
| chr8 | LINC00293 | 47737226 | 47737626 | 401 | * | 6.82 | 6.06 | 7.31 | -1.16 | 1.47E-15 | 3.43E-13 | 12.46471 | -1.16 | down |
| chr14 | MDGA2 | 47675972 | 47676372 | 401 | * | 3.61 | 2.33 | 4.28 | -1.22 | 0.00016 | 0.003 | 2.522879 | -1.22 | down |
| chrX | ZNF81 | 47637510 | 47637910 | 401 | * | 6.64 | 4.95 | 7.4 | -2.42 | 3.70E-36 | 5.57E-33 | 32.25414 | -2.42 | down |
| chr2 | EPCAM | 47571248 | 47571648 | 401 | * | 5.26 | 4.28 | 5.84 | -1.34 | 1.61E-08 | 1.08E-06 | 5.966576 | -1.34 | down |
| chr19 | STRN4 | 47262401 | 47262801 | 401 | * | 5.6 | 4.6 | 6.19 | -1.43 | 1.48E-10 | 1.55E-08 | 7.809668 | -1.43 | down |
| chrX | ZNF157 | 47179263 | 47179663 | 401 | * | 5.29 | 4.34 | 5.86 | -1.25 | 9.03E-08 | 5.05E-06 | 5.296709 | -1.25 | down |
| chr14 | RPL10L | 47040611 | 47041011 | 401 | * | 5.1 | 0.94 | 6.05 | -4.68 | 4.64E-16 | 1.17E-13 | 12.93181 | -4.68 | down |

| | | | | | | | | | | | | | | |
|---|---|---|---|---|---|---|---|---|---|---|---|---|---|---|
| chr19 | RNU6-66P | 46821371 | 46821771 | 401 | * | 5.4 | 4.54 | 5.94 | -1.16 | 7.92E-08 | 4.49E-06 | 5.347754 | -1.16 | down |
| chr14 | LINC00871 | 46571746 | 46572146 | 401 | * | 3.23 | 0.41 | 4.13 | -2.96 | 6.44E-06 | 0.000207 | 3.68403 | -2.96 | down |
| chr19 | PGLYRP1 | 46529869 | 46530269 | 401 | * | 5.54 | 4.72 | 6.06 | -1.14 | 5.79E-08 | 3.39E-06 | 5.4698 | -1.14 | down |
| chr14 | LINC00871 | 46480836 | 46481236 | 401 | * | 5.94 | 5.01 | 6.5 | -1.36 | 3.67E-12 | 5.12E-10 | 9.29073 | -1.36 | down |
| chr1 | MAST2 | 46277990 | 46278390 | 401 | * | 4.95 | 3.89 | 5.55 | -1.39 | 9.16E-08 | 5.12E-06 | 5.29073 | -1.39 | down |
| chr14 | MIS18BP1 | 46048334 | 46048734 | 401 | * | 4.23 | 3.14 | 4.84 | -1.24 | 1.85E-05 | 0.000503 | 3.298432 | -1.24 | down |
| chr1 | LINC01144 | 45769489 | 45769889 | 401 | * | 5.48 | 4.7 | 5.98 | -1.06 | 3.18E-07 | 1.53E-05 | 4.815309 | -1.06 | down |
| chr2 | LINC01121 | 45567887 | 45568287 | 401 | * | 3.42 | 1.37 | 4.24 | -2.22 | 0.000017 | 0.000468 | 3.329754 | -2.22 | down |
| chr19 | TOMM40 | 45398164 | 45398564 | 401 | * | 5.24 | 4.35 | 5.79 | -1.17 | 1.78E-07 | 9.15E-06 | 5.038579 | -1.17 | down |
| chr13 | LINC00330 | 45361549 | 45361949 | 401 | * | 4.68 | 3.77 | 5.23 | -1.09 | 1.04E-05 | 0.000309 | 3.510042 | -1.09 | down |
| chr11 | SYT13 | 45355251 | 45355651 | 401 | * | 4.15 | 2.17 | 4.95 | -2.55 | 1.24E-08 | 8.61E-07 | 6.064997 | -2.55 | down |
| chr11 | LINC02685 | 45048978 | 45049378 | 401 | * | 5.55 | 4.49 | 6.16 | -1.52 | 4.00E-11 | 4.66E-09 | 8.331614 | -1.52 | down |
| chr2 | CAMKMT | 44642135 | 44642535 | 401 | * | 5.6 | 4.8 | 6.12 | -1.11 | 4.19E-08 | 2.55E-06 | 5.59346 | -1.11 | down |
| chr19 | ZNF283 | 44356542 | 44356942 | 401 | * | 3.58 | 1.83 | 4.35 | -1.99 | 1.05E-05 | 0.000313 | 3.504456 | -1.99 | down |
| chr7 | CAMK2B | 44331718 | 44332118 | 401 | * | 3.42 | 1.97 | 4.13 | -1.31 | 0.000168 | 0.0031 | 2.508638 | -1.31 | down |
| chr19 | SMG9 | 44259685 | 44260085 | 401 | * | 4.38 | 3.36 | 4.97 | -1.15 | 2.17E-05 | 0.000573 | 3.241845 | -1.15 | down |
| chr20 | SPINT3 | 44133448 | 44133848 | 401 | * | 3.77 | 1.85 | 4.57 | -2.4 | 6.28E-07 | 2.77E-05 | 4.55752 | -2.4 | down |
| chr17 | KANSL1 | 44108709 | 44109109 | 401 | * | 5.3 | 4.29 | 5.88 | -1.36 | 7.72E-09 | 5.56E-07 | 6.254925 | -1.36 | down |
| chr11 | ALKBH3-AS1 | 43952352 | 43952752 | 401 | * | 6.37 | 5.5 | 6.91 | -1.31 | 1.65E-14 | 3.31E-12 | 11.48017 | -1.31 | down |
| chr13 | ENOX1 | 43879330 | 43879730 | 401 | * | 6.08 | 5.23 | 6.62 | -1.24 | 2.18E-11 | 2.66E-09 | 8.575118 | -1.24 | down |
| chr22 | MPPED1 | 43862529 | 43862929 | 401 | * | 3.38 | 1.94 | 4.08 | -1.25 | 0.000216 | 0.0038 | 2.420216 | -1.25 | down |
| chr12 | ADAMTS20 | 43847823 | 43848223 | 401 | * | 6.89 | 6.16 | 7.38 | -1.13 | 3.41E-15 | 7.51E-13 | 12.12436 | -1.13 | down |
| chr13 | TNFSF11 | 43058276 | 43058676 | 401 | * | 4.99 | 4.11 | 5.53 | -1.1 | 4.51E-06 | 0.000153 | 3.815309 | -1.1 | down |
| chr13 | TNFSF11 | 43036228 | 43036628 | 401 | * | 4.33 | 2.95 | 5.03 | -1.78 | 3.16E-07 | 1.52E-05 | 4.818156 | -1.78 | down |
| chr13 | TNFSF11 | 43023493 | 43023893 | 401 | * | 6.38 | 5.15 | 7.04 | -1.83 | 4.48E-22 | 2.42E-19 | 18.61618 | -1.83 | down |
| chr3 | CCDC13 | 42811428 | 42811828 | 401 | * | 4.41 | 3.44 | 4.98 | -1.04 | 5.88E-05 | 0.00131 | 2.882729 | -1.04 | down |
| chr17 | FZD2 | 42653674 | 42654074 | 401 | * | 3.22 | 1.21 | 4.03 | -1.99 | 4.93E-05 | 0.00114 | 2.943095 | -1.99 | down |
| chr19 | CD79A | 42374371 | 42374771 | 401 | * | 5.14 | 4.34 | 5.65 | -1.01 | 6.07E-06 | 0.000197 | 3.705534 | -1.01 | down |

| | | | | | | | | | | | | | | |
|---|---|---|---|---|---|---|---|---|---|---|---|---|---|---|
| chr2 | PKDCC | 42331730 | 42332130 | 401 | * | 5.92 | 5.2 | 6.4 | -1.03 | 3.44E-08 | 2.13E-06 | 5.67162 | -1.03 | down |
| chr17 | ASB16 | 42248582 | 42248982 | 401 | * | 5.55 | 3.27 | 6.39 | -3.03 | 1.45E-22 | 8.07E-20 | 19.09313 | -3.03 | down |
| chr15 | MGA | 41930432 | 41930832 | 401 | * | 3.39 | 2.06 | 4.07 | -1.02 | 0.00052 | 0.00766 | 2.115771 | -1.02 | down |
| chrX | CASK | 41737088 | 41737488 | 401 | * | 4.97 | 3.91 | 5.58 | -1.42 | 4.83E-08 | 2.89E-06 | 5.539102 | -1.42 | down |
| chr6 | PGC | 41723499 | 41723899 | 401 | * | 5.65 | 4.8 | 6.18 | -1.16 | 1.7E-08 | 1.14E-06 | 5.943095 | -1.16 | down |
| chr20 | PTPRT | 41525186 | 41525586 | 401 | * | 4.73 | 3.82 | 5.29 | -1.12 | 6.77E-06 | 0.000216 | 3.665546 | -1.12 | down |
| chr13 | MIR621 | 41426143 | 41426543 | 401 | * | 3.52 | 2.27 | 4.17 | -1.06 | 0.000334 | 0.00537 | 2.270026 | -1.06 | down |
| chr13 | MIR621 | 41398752 | 41399152 | 401 | * | 3.73 | 2.43 | 4.41 | -1.33 | 7.78E-05 | 0.00166 | 2.779892 | -1.33 | down |
| chr1 | CITED4 | 41353754 | 41354154 | 401 | * | 5.01 | 3.85 | 5.65 | -1.61 | 4.79E-09 | 3.64E-07 | 6.438899 | -1.61 | down |
| chr21 | PCP4 | 41209233 | 41209633 | 401 | * | 4.91 | 3.98 | 5.46 | -1.16 | 1.88E-06 | 7.16E-05 | 4.145087 | -1.16 | down |
| chr4 | APBB2 | 40856978 | 40857378 | 401 | * | 4.29 | 3.1 | 4.93 | -1.43 | 5.44E-06 | 0.000179 | 3.747147 | -1.43 | down |
| chr3 | ZNF621 | 40681593 | 40681993 | 401 | * | 4.36 | 3.38 | 4.93 | -1.07 | 0.000046 | 0.00107 | 2.970616 | -1.07 | down |
| chrX | MED14 | 40539187 | 40539587 | 401 | * | 4.55 | 3.56 | 5.13 | -1.19 | 7.41E-06 | 0.000232 | 3.634512 | -1.19 | down |
| chr22 | TNRC6B | 40463241 | 40463641 | 401 | * | 5.36 | 4.47 | 5.91 | -1.2 | 6.66E-08 | 3.85E-06 | 5.414539 | -1.2 | down |
| chr17 | CNP | 40108165 | 40108565 | 401 | * | 5.85 | 5.02 | 6.37 | -1.18 | 1.05E-09 | 9.24E-08 | 7.034328 | -1.18 | down |
| chr14 | FBXO33 | 40004776 | 40005176 | 401 | * | 4.37 | 2.37 | 5.18 | -2.64 | 5.56E-10 | 5.15E-08 | 7.288193 | -2.64 | down |
| chr19 | RPS16 | 39926409 | 39926809 | 401 | * | 5.67 | 4.62 | 6.27 | -1.5 | 2.07E-11 | 2.55E-09 | 8.59346 | -1.5 | down |
| chr15 | THBS1 | 39831001 | 39831401 | 401 | * | 3.47 | 2.08 | 4.16 | -1.28 | 0.000161 | 0.00301 | 2.521434 | -1.28 | down |
| chr3 | XIRP1 | 39267642 | 39268042 | 401 | * | 5.94 | 5.16 | 6.45 | -1.13 | 1.12E-09 | 9.77E-08 | 7.010105 | -1.13 | down |
| chr3 | XIRP1 | 39260917 | 39261317 | 401 | * | 6.3 | 5.46 | 6.82 | -1.24 | 1.12E-12 | 1.72E-10 | 9.764472 | -1.24 | down |
| chr3 | ACVR2B | 38459186 | 38459586 | 401 | * | 5.16 | 3.88 | 5.83 | -1.8 | 2.39E-10 | 2.39E-08 | 7.621602 | -1.8 | down |
| chr7 | STARD3NL | 38232904 | 38233304 | 401 | * | 4.99 | 4.06 | 5.55 | -1.2 | 6.22E-07 | 2.75E-05 | 4.560667 | -1.2 | down |
| chr8 | DDHD2 | 38090473 | 38090873 | 401 | * | 6.33 | 5.26 | 6.94 | -1.6 | 9.97E-18 | 3.20E-15 | 14.49485 | -1.6 | down |
| chr22 | TRIOBP | 38080860 | 38081260 | 401 | * | 5.88 | 5.06 | 6.4 | -1.15 | 2.63E-09 | 2.12E-07 | 6.673664 | -1.15 | down |
| chr11 | RAG2 | 37721398 | 37721798 | 401 | * | 3.06 | 1.18 | 3.85 | -1.59 | 0.000162 | 0.00303 | 2.518557 | -1.59 | down |
| chr4 | RELL1 | 37659638 | 37660038 | 401 | * | 4.73 | 3.43 | 5.4 | -1.76 | 1.47E-08 | 9.98E-07 | 6.000869 | -1.76 | down |
| chr7 | ELMO1 | 37353586 | 37353986 | 401 | * | 6.07 | 2.04 | 7.02 | -4.75 | 5.86E-31 | 5.80E-28 | 27.23657 | -4.75 | down |
| chr19 | ZNF850 | 37251688 | 37252088 | 401 | * | 3.55 | 2.21 | 4.23 | -1.26 | 0.00015 | 0.00284 | 2.546682 | -1.26 | down |

| | | | | | | | | | | | | | | |
|---|---|---|---|---|---|---|---|---|---|---|---|---|---|---|
| chr7 | ELMO1 | 37129013 | 37129413 | 401 | * | 6 | 5.3 | 6.47 | -1.01 | 1.18E-08 | 8.25E-07 | 6.083546 | -1.01 | down |
| chr14 | MBIP | 36754404 | 36754804 | 401 | * | 5.02 | 4.22 | 5.53 | -1.02 | 5.8E-06 | 0.000189 | 3.723538 | -1.02 | down |
| chr6 | MIR3925 | 36577321 | 36577721 | 401 | * | 7.32 | 6.54 | 7.81 | -1.2 | 2.35E-21 | 1.21E-18 | 17.91721 | -1.2 | down |
| chr20 | VSTM2L | 36573014 | 36573414 | 401 | * | 6.21 | 5.17 | 6.8 | -1.53 | 1.24E-14 | 2.55E-12 | 11.59346 | -1.53 | down |
| chr15 | MIR4510 | 36449250 | 36449650 | 401 | * | 5.9 | 4.21 | 6.66 | -2.4 | 1.69E-22 | 9.35E-20 | 19.02919 | -2.4 | down |
| chr19 | LRFN3 | 36418397 | 36418797 | 401 | * | 5.2 | 4.03 | 5.84 | -1.66 | 6.16E-10 | 5.68E-08 | 7.245652 | -1.66 | down |
| chr15 | MIR4510 | 36359314 | 36359714 | 401 | * | 4.03 | 3.02 | 4.62 | -1.01 | 0.000164 | 0.00305 | 2.5157 | -1.01 | down |
| chr7 | KIAA0895 | 36312545 | 36312945 | 401 | * | 3.86 | 2.68 | 4.5 | -1.17 | 0.000105 | 0.00213 | 2.67162 | -1.17 | down |
| chr18 | MIR924HG | 36200360 | 36200760 | 401 | * | 6.18 | 5.43 | 6.67 | -1.1 | 1.78E-10 | 1.85E-08 | 7.732828 | -1.1 | down |
| chr4 | ARAP2 | 36111894 | 36112294 | 401 | * | 3.94 | 1.95 | 4.75 | -2.51 | 1.28E-07 | 6.85E-06 | 5.164309 | -2.51 | down |
| chr22 | RASD2 | 35957318 | 35957718 | 401 | * | 4.96 | 4.02 | 5.53 | -1.21 | 8.06E-07 | 3.46E-05 | 4.460924 | -1.21 | down |
| chr4 | ARAP2 | 35908632 | 35909032 | 401 | * | 3.53 | 1.88 | 4.29 | -1.74 | 3.91E-05 | 0.000936 | 3.028724 | -1.74 | down |
| chr4 | ARAP2 | 35818508 | 35818908 | 401 | * | 5.95 | 3.69 | 6.79 | -3.03 | 3.84E-29 | 3.39E-26 | 25.4698 | -3.03 | down |
| chr4 | ARAP2 | 35760833 | 35761233 | 401 | * | 8.51 | 6.16 | 9.36 | -3.19 | ######## | ######## | 155.1726 | -3.19 | down |
| chr22 | HMGXB4 | 35627440 | 35627840 | 401 | * | 4.85 | 3.93 | 5.4 | -1.14 | 3.12E-06 | 0.000112 | 3.950782 | -1.14 | down |
| chr11 | PAMR1 | 35526400 | 35526800 | 401 | * | 4.13 | 3.13 | 4.72 | -1.03 | 0.000115 | 0.00229 | 2.640165 | -1.03 | down |
| chr1 | DLGAP3 | 35355869 | 35356269 | 401 | * | 4.25 | 3.25 | 4.83 | -1.07 | 6.85E-05 | 0.00149 | 2.826814 | -1.07 | down |
| chr2 | CRIM1-DT | 35298499 | 35298899 | 401 | * | 3.6 | 1.34 | 4.45 | -2.67 | 9.06E-07 | 3.82E-05 | 4.417937 | -2.67 | down |
| chr18 | MIR4318 | 35267273 | 35267673 | 401 | * | 5.71 | 3.13 | 6.58 | -3.36 | 4.08E-26 | 3.03E-23 | 22.51856 | -3.36 | down |
| chr10 | PARD3 | 35149583 | 35149983 | 401 | * | 4.38 | 2.69 | 5.14 | -2.24 | 1.17E-08 | 8.2E-07 | 6.086186 | -2.24 | down |
| chr11 | CD44 | 35082521 | 35082921 | 401 | * | 6.6 | 5.88 | 7.08 | -1.09 | 1.95E-12 | 2.88E-10 | 9.540608 | -1.09 | down |
| chr8 | UNC5D | 34910357 | 34910757 | 401 | * | 4.15 | 2.37 | 4.92 | -2.31 | 4.78E-08 | 2.86E-06 | 5.543634 | -2.31 | down |
| chr8 | UNC5D | 34905400 | 34905800 | 401 | * | 3.83 | 2.28 | 4.56 | -1.82 | 6.18E-06 | 0.0002 | 3.69897 | -1.82 | down |
| chr20 | PHF20 | 34474208 | 34474608 | 401 | * | 4.58 | 2.36 | 5.42 | -2.89 | 5.02E-12 | 6.79E-10 | 9.16813 | -2.89 | down |
| chr20 | SPAG4 | 34216051 | 34216451 | 401 | * | 6.34 | 5.55 | 6.84 | -1.16 | 4.26E-12 | 5.84E-10 | 9.233587 | -1.16 | down |
| chr18 | FHOD3 | 34061788 | 34062188 | 401 | * | 4.01 | 1.57 | 4.87 | -3.03 | 6.41E-09 | 4.72E-07 | 6.326058 | -3.03 | down |
| chr3 | PDCD6IP | 33911860 | 33912260 | 401 | * | 4.86 | 3.94 | 5.42 | -1.12 | 7.05E-06 | 0.000223 | 3.651695 | -1.12 | down |
| chr6 | LINC01016 | 33909783 | 33910183 | 401 | * | 5.93 | 4.97 | 6.51 | -1.39 | 5.21E-12 | 7.01E-10 | 9.154282 | -1.39 | down |

| | | | | | | | | | | | | | | |
|---|---|---|---|---|---|---|---|---|---|---|---|---|---|---|
| chr20 | UQCC1 | 33909487 | 33909887 | 401 | * | 4.7 | 3.66 | 5.3 | -1.31 | 1.02E-06 | 4.22E-05 | 4.374688 | -1.31 | down |
| chr18 | FHOD3 | 33838764 | 33839164 | 401 | * | 3 | 1.16 | 3.78 | -1.39 | 0.000306 | 0.005 | 2.30103 | -1.39 | down |
| chr18 | MOCOS | 33756188 | 33756588 | 401 | * | 7.56 | 6.41 | 8.19 | -1.76 | 3.24E-42 | 6.73E-39 | 38.17198 | -1.76 | down |
| chr18 | MOCOS | 33738835 | 33739235 | 401 | * | 5.23 | 4.26 | 5.81 | -1.32 | 3.07E-08 | 1.94E-06 | 5.712198 | -1.32 | down |
| chr18 | ELP2 | 33735409 | 33735809 | 401 | * | 4.57 | 3.14 | 5.28 | -1.95 | 1.03E-08 | 7.29E-07 | 6.137272 | -1.95 | down |
| chr18 | ELP2 | 33711563 | 33711963 | 401 | * | 6.76 | 5.57 | 7.41 | -1.8 | 1.18E-24 | 8.01E-22 | 21.09637 | -1.8 | down |
| chr2 | RASGRP3 | 33607149 | 33607549 | 401 | * | 4.36 | 3.16 | 5.01 | -1.44 | 2.89E-06 | 0.000104 | 3.982967 | -1.44 | down |
| chr18 | C18orf21 | 33578881 | 33579281 | 401 | * | 4.9 | 3.17 | 5.66 | -2.37 | 3.99E-12 | 5.52E-10 | 9.258061 | -2.37 | down |
| chr18 | C18orf21 | 33577972 | 33578372 | 401 | * | 5.63 | 3.95 | 6.38 | -2.39 | 5.95E-19 | 2.20E-16 | 15.65758 | -2.39 | down |
| chr18 | C18orf21 | 33560650 | 33561050 | 401 | * | 4.55 | 3.42 | 5.18 | -1.42 | 1.21E-06 | 4.92E-05 | 4.308035 | -1.42 | down |
| chr18 | MIR187 | 33469721 | 33470121 | 401 | * | 8.48 | 7.87 | 8.91 | -1.01 | 3.18E-32 | 3.64E-29 | 28.4389 | -1.01 | down |
| chr18 | MIR187 | 33440173 | 33440573 | 401 | * | 5.72 | 4.76 | 6.29 | -1.35 | 1.82E-10 | 1.88E-08 | 7.725842 | -1.35 | down |
| chr18 | GALNT1 | 33232041 | 33232441 | 401 | * | 5.17 | 4.26 | 5.72 | -1.15 | 9.21E-07 | 3.87E-05 | 4.412289 | -1.15 | down |
| chr5 | NPR3 | 32759930 | 32760330 | 401 | * | 3.51 | 2.29 | 4.17 | -1.03 | 0.000392 | 0.00612 | 2.213249 | -1.03 | down |
| chr20 | EIF2S2 | 32743243 | 32743643 | 401 | * | 5.3 | 4.33 | 5.87 | -1.28 | 5.65E-08 | 3.32E-06 | 5.478862 | -1.28 | down |
| chr5 | NPR3 | 32714015 | 32714415 | 401 | * | 6.39 | 4.28 | 7.21 | -2.88 | 1.76E-35 | 2.49E-32 | 31.6038 | -2.88 | down |
| chr5 | NPR3 | 32712038 | 32712438 | 401 | * | 6.3 | 4.02 | 7.14 | -3.07 | 1.45E-36 | 2.30E-33 | 32.63827 | -3.07 | down |
| chr5 | NPR3 | 32709779 | 32710179 | 401 | * | 6.21 | 5.5 | 6.69 | -1.04 | 6.51E-10 | 5.96E-08 | 7.224754 | -1.04 | down |
| chr1 | KPNA6 | 32612114 | 32612514 | 401 | * | 6.29 | 4.33 | 7.09 | -2.72 | 4.09E-32 | 4.60E-29 | 28.33724 | -2.72 | down |
| chr3 | CMTM7 | 32422132 | 32422532 | 401 | * | 4.32 | 3.36 | 4.9 | -1.01 | 0.000105 | 0.00213 | 2.67162 | -1.01 | down |
| chr12 | BICD1 | 32390556 | 32390956 | 401 | * | 4.49 | 3.56 | 5.05 | -1.02 | 7.46E-05 | 0.0016 | 2.79588 | -1.02 | down |
| chr14 | NUBPL | 32076428 | 32076828 | 401 | * | 6.5 | 5.79 | 6.97 | -1.06 | 1.76E-11 | 2.22E-09 | 8.653647 | -1.06 | down |
| chr1 | PUM1 | 31505967 | 31506367 | 401 | * | 5.97 | 5.09 | 6.52 | -1.28 | 1.85E-11 | 2.33E-09 | 8.632644 | -1.28 | down |
| chr16 | ITGAX | 31386608 | 31387008 | 401 | * | 4.58 | 3.64 | 5.14 | -1.07 | 2.37E-05 | 0.000619 | 3.208309 | -1.07 | down |
| chr14 | COCH | 31336683 | 31337083 | 401 | * | 4.31 | 3.27 | 4.91 | -1.17 | 2.13E-05 | 0.000563 | 3.249492 | -1.17 | down |
| chr2 | CAPN13 | 31137209 | 31137609 | 401 | * | 4.64 | 3.73 | 5.19 | -1.06 | 2.14E-05 | 0.000567 | 3.246417 | -1.06 | down |
| chr12 | IPO8 | 30862464 | 30862864 | 401 | * | 4.76 | 3.61 | 5.39 | -1.5 | 1.15E-07 | 6.22E-06 | 5.20621 | -1.5 | down |
| chr10 | MTPAP | 30529501 | 30529901 | 401 | * | 3.64 | 1.97 | 4.4 | -1.94 | 8.47E-06 | 0.000259 | 3.5867 | -1.94 | down |

| | | | | | | | | | | | | | | |
|---|---|---|---|---|---|---|---|---|---|---|---|---|---|---|
| chr16 | ITGAL | 30472539 | 30472939 | 401 | * | 3.11 | 1.43 | 3.87 | -1.35 | 0.000294 | 0.00486 | 2.313364 | -1.35 | down |
| chr2 | YPEL5 | 30274644 | 30275044 | 401 | * | 5.23 | 4.4 | 5.76 | -1.1 | 5.47E-07 | 2.46E-05 | 4.609065 | -1.1 | down |
| chr16 | C16orf92 | 30033292 | 30033692 | 401 | * | 5.29 | 4.52 | 5.79 | -1.02 | 1.95E-06 | 7.38E-05 | 4.131944 | -1.02 | down |
| chr14 | MIR548AI | 29805627 | 29806027 | 401 | * | 3.92 | 1.39 | 4.79 | -3.09 | 1.29E-08 | 8.9E-07 | 6.05061 | -3.09 | down |
| chr20 | DEFB115 | 29781823 | 29782223 | 401 | * | 3.71 | 0.32 | 4.64 | -3.65 | 9.54E-08 | 5.29E-06 | 5.276544 | -3.65 | down |
| chr16 | C16orf54 | 29745892 | 29746292 | 401 | * | 6.75 | 5.27 | 7.47 | -2.17 | 9.14E-35 | 1.26E-31 | 30.89963 | -2.17 | down |
| chr9 | LINGO2 | 29740504 | 29740904 | 401 | * | 5.14 | 4.31 | 5.67 | -1.05 | 2.44E-06 | 9.01E-05 | 4.045275 | -1.05 | down |
| chrX | MAGEB2 | 29662672 | 29663072 | 401 | * | 4.13 | 2.44 | 4.89 | -2.18 | 1.34E-07 | 7.16E-06 | 5.145087 | -2.18 | down |
| chr4 | MIR4275 | 29467045 | 29467445 | 401 | * | 3.83 | 0 | 4.81 | -4.71 | 2.25E-07 | 1.13E-05 | 4.946922 | -4.71 | down |
| chr22 | ZNRF3 | 29313178 | 29313578 | 401 | * | 7.29 | 6.64 | 7.73 | -1.01 | 4.20E-16 | 1.06E-13 | 12.97469 | -1.01 | down |
| chr4 | MIR4275 | 29248260 | 29248660 | 401 | * | 3.25 | 0 | 4.17 | -3.24 | 6.11E-06 | 0.000198 | 3.703335 | -3.24 | down |
| chr9 | LINGO2 | 29160731 | 29161131 | 401 | * | 3.62 | 2.41 | 4.26 | -1.08 | 0.000287 | 0.00477 | 2.321482 | -1.08 | down |
| chr9 | LINGO2 | 29144505 | 29144905 | 401 | * | 6.94 | 6.12 | 7.46 | -1.25 | 7.19E-18 | 2.33E-15 | 14.63264 | -1.25 | down |
| chr22 | TTC28 | 28927571 | 28927971 | 401 | * | 5.73 | 4.97 | 6.23 | -1.07 | 5.43E-08 | 3.21E-06 | 5.493495 | -1.07 | down |
| chr9 | MIR873 | 28883210 | 28883610 | 401 | * | 3.53 | 2.16 | 4.22 | -1.21 | 0.000196 | 0.00352 | 2.453457 | -1.21 | down |
| chr15 | GOLGA8G | 28832088 | 28832488 | 401 | * | 6.31 | 2.97 | 7.24 | -3.82 | 3.81E-10 | 3.64E-08 | 7.438899 | -3.82 | down |
| chr9 | MIR876 | 28818987 | 28819387 | 401 | * | 3.63 | 2.47 | 4.27 | -1.01 | 0.000386 | 0.00605 | 2.218245 | -1.01 | down |
| chr13 | PAN3 | 28693543 | 28693943 | 401 | * | 3.85 | 2.16 | 4.61 | -2.05 | 1.8E-06 | 6.91E-05 | 4.160522 | -2.05 | down |
| chr2 | LOC100505716 | 28516935 | 28517335 | 401 | * | 4.23 | 3.24 | 4.81 | -1.07 | 6.64E-05 | 0.00146 | 2.835647 | -1.07 | down |
| chr22 | TTC28-AS1 | 28442199 | 28442599 | 401 | * | 5.85 | 4.35 | 6.57 | -2.16 | 1.01E-19 | 4.00E-17 | 16.39794 | -2.16 | down |
| chr2 | LOC100505716 | 28406712 | 28407112 | 401 | * | 5.38 | 4.5 | 5.92 | -1.19 | 4.5E-08 | 2.7E-06 | 5.568636 | -1.19 | down |
| chr22 | TTC28-AS1 | 28369841 | 28370241 | 401 | * | 5.01 | 4.17 | 5.54 | -1.05 | 5.61E-06 | 0.000184 | 3.735182 | -1.05 | down |
| chr9 | LINGO2 | 28270127 | 28270527 | 401 | * | 4.89 | 3.9 | 5.47 | -1.28 | 3.97E-07 | 1.86E-05 | 4.730487 | -1.28 | down |
| chr17 | EFCAB5 | 28227653 | 28228053 | 401 | * | 5.73 | 4.36 | 6.42 | -1.97 | 2.91E-16 | 7.54E-14 | 13.12263 | -1.97 | down |
| chr16 | GSG1L | 27993581 | 27993981 | 401 | * | 6.68 | 5.87 | 7.2 | -1.23 | 8.62E-15 | 1.83E-12 | 11.73755 | -1.23 | down |
| chr13 | RASL11A | 27919772 | 27920172 | 401 | * | 4.09 | 3.04 | 4.69 | -1.11 | 7.49E-05 | 0.00161 | 2.793174 | -1.11 | down |
| chr6 | LINC01012 | 27679544 | 27679944 | 401 | * | 4.82 | 2.97 | 5.6 | -2.52 | 2.98E-12 | 4.23E-10 | 9.37366 | -2.52 | down |
| chr4 | STIM2 | 27604656 | 27605056 | 401 | * | 4.17 | 1.83 | 5.02 | -2.95 | 1.08E-09 | 9.44E-08 | 7.025028 | -2.95 | down |

| | | | | | | | | | | | | | | |
|---|---|---|---|---|---|---|---|---|---|---|---|---|---|---|
| chr6 | LINC01012 | 27602410 | 27602810 | 401 | * | 4.87 | 3.14 | 5.64 | -2.4 | 9.81E-12 | 1.27E-09 | 8.896196 | -2.4 | down |
| chrX | PPP4R3C | 27598264 | 27598664 | 401 | * | 3.72 | 0 | 4.69 | -4.08 | 8.06E-07 | 3.46E-05 | 4.460924 | -4.08 | down |
| chrX | PPP4R3C | 27566039 | 27566439 | 401 | * | 5.15 | 0.08 | 6.13 | -5.35 | 6.30E-13 | 1.01E-10 | 9.995679 | -5.35 | down |
| chr6 | LINC01012 | 27561585 | 27561985 | 401 | * | 5.42 | 4 | 6.12 | -2.05 | 1.82E-14 | 3.62E-12 | 11.44129 | -2.05 | down |
| chr4 | STIM2 | 27474631 | 27475031 | 401 | * | 4.42 | 2.96 | 5.13 | -1.93 | 6.23E-08 | 3.62E-06 | 5.441291 | -1.93 | down |
| chr4 | STIM2 | 27442138 | 27442538 | 401 | * | 3.83 | 2.7 | 4.45 | -1.05 | 0.000225 | 0.00394 | 2.404504 | -1.05 | down |
| chr6 | ZNF391 | 27375072 | 27375472 | 401 | * | 5.15 | 3.07 | 5.97 | -2.83 | 3.26E-16 | 8.40E-14 | 13.07572 | -2.83 | down |
| chr22 | MIAT | 27314379 | 27314779 | 401 | * | 6.27 | 5.56 | 6.74 | -1.04 | 1.64E-09 | 1.36E-07 | 6.866461 | -1.04 | down |
| chr6 | VN1R10P | 27303603 | 27304003 | 401 | * | 4.42 | 3.33 | 5.03 | -1.3 | 5.67E-06 | 0.000186 | 3.730487 | -1.3 | down |
| chr6 | VN1R10P | 27290554 | 27290954 | 401 | * | 6.38 | 5.31 | 6.98 | -1.6 | 2.19E-18 | 7.49E-16 | 15.12552 | -1.6 | down |
| chr6 | PRSS16 | 27238323 | 27238723 | 401 | * | 5.57 | 4.72 | 6.1 | -1.18 | 1.72E-08 | 1.15E-06 | 5.939302 | -1.18 | down |
| chr6 | PRSS16 | 27230328 | 27230728 | 401 | * | 6.72 | 6.04 | 7.18 | -1.04 | 2.72E-12 | 3.92E-10 | 9.406714 | -1.04 | down |
| chr7 | HOXA1 | 27095954 | 27096354 | 401 | * | 5.12 | 3.47 | 5.88 | -2.32 | 7.68E-14 | 1.41E-11 | 10.85078 | -2.32 | down |
| chr1 | ARID1A | 27027010 | 27027410 | 401 | * | 5.03 | 4.01 | 5.62 | -1.36 | 7.51E-08 | 4.29E-06 | 5.367543 | -1.36 | down |
| chr6 | ZNF322 | 26756472 | 26756872 | 401 | * | 4.26 | 2.29 | 5.07 | -2.6 | 2.20E-09 | 1.8E-07 | 6.744727 | -2.6 | down |
| chr6 | ZNF322 | 26744989 | 26745389 | 401 | * | 6.54 | 5.59 | 7.11 | -1.43 | 1.65E-17 | 5.10E-15 | 14.29243 | -1.43 | down |
| chr6 | ZNF322 | 26743170 | 26743570 | 401 | * | 5.32 | 4.37 | 5.89 | -1.27 | 3.36E-08 | 2.08E-06 | 5.681937 | -1.27 | down |
| chr4 | TBC1D19 | 26692018 | 26692418 | 401 | * | 3.63 | 2.39 | 4.29 | -1.15 | 0.000196 | 0.00352 | 2.453457 | -1.15 | down |
| chr22 | SEZ6L | 26610246 | 26610646 | 401 | * | 5.41 | 4.55 | 5.94 | -1.17 | 5.51E-08 | 3.24E-06 | 5.489455 | -1.17 | down |
| chr14 | NOVA1 | 26598292 | 26598692 | 401 | * | 3.91 | 0.68 | 4.83 | -3.65 | 8.77E-09 | 6.26E-07 | 6.203426 | -3.65 | down |
| chr4 | TBC1D19 | 26583859 | 26584259 | 401 | * | 6.85 | 5.9 | 7.42 | -1.44 | 5.33E-21 | 2.55E-18 | 17.59346 | -1.44 | down |
| chr6 | ABT1 | 26575632 | 26576032 | 401 | * | 6.44 | 5.7 | 6.93 | -1.1 | 1.07E-11 | 1.38E-09 | 8.860121 | -1.1 | down |
| chr11 | ANO3 | 26323814 | 26324214 | 401 | * | 5.66 | 4.85 | 6.17 | -1.14 | 1.31E-08 | 9.03E-07 | 6.044312 | -1.14 | down |
| chr14 | STXBP6 | 26199576 | 26199976 | 401 | * | 3.44 | 1.51 | 4.24 | -2.1 | 1.55E-05 | 0.000434 | 3.36251 | -2.1 | down |
| chr12 | RASSF8 | 26126314 | 26126714 | 401 | * | 5.24 | 4.27 | 5.81 | -1.31 | 5.16E-08 | 3.07E-06 | 5.512862 | -1.31 | down |
| chr20 | FAM182B | 25868053 | 25868453 | 401 | * | 4.81 | 3.82 | 5.39 | -1.23 | 1.82E-06 | 6.98E-05 | 4.156145 | -1.23 | down |
| chr4 | SLC34A2 | 25723000 | 25723400 | 401 | * | 6.29 | 5.59 | 6.76 | -1.04 | 3.93E-10 | 3.73E-08 | 7.428291 | -1.04 | down |
| chr18 | CDH2 | 25505325 | 25505725 | 401 | * | 5.42 | 4.43 | 6.01 | -1.39 | 1.25E-09 | 1.08E-07 | 6.966576 | -1.39 | down |

| | | | | | | | | | | | | | | |
|---|---|---|---|---|---|---|---|---|---|---|---|---|---|---|
| chr4 | ANAPC4 | 25441532 | 25441932 | 401 | * | 4.46 | 3.41 | 5.06 | -1.22 | 9.1E-06 | 0.000276 | 3.559091 | -1.22 | down |
| chr4 | ANAPC4 | 25439939 | 25440339 | 401 | * | 5.06 | 4.2 | 5.6 | -1.12 | 1.36E-06 | 5.45E-05 | 4.263603 | -1.12 | down |
| chr18 | CDH2 | 25282276 | 25282676 | 401 | * | 4.82 | 3.91 | 5.37 | -1.13 | 4.95E-06 | 0.000166 | 3.779892 | -1.13 | down |
| chr4 | PI4K2B | 25248751 | 25249151 | 401 | * | 7.1 | 6.31 | 7.61 | -1.22 | 1.10E-19 | 4.36E-17 | 16.36051 | -1.22 | down |
| chr4 | PI4K2B | 25246956 | 25247356 | 401 | * | 5.41 | 4.4 | 6.01 | -1.43 | 1.33E-09 | 1.13E-07 | 6.946922 | -1.43 | down |
| chr18 | CDH2 | 25233715 | 25234115 | 401 | * | 5.59 | 4.78 | 6.1 | -1.1 | 9.79E-08 | 5.42E-06 | 5.266001 | -1.1 | down |
| chr22 | GUCD1 | 24961466 | 24961866 | 401 | * | 4.26 | 3.24 | 4.86 | -1.14 | 3.26E-05 | 0.000802 | 3.095826 | -1.14 | down |
| chr1 | SRRM1 | 24929469 | 24929869 | 401 | * | 5.36 | 4.55 | 5.87 | -1.06 | 5.41E-07 | 2.43E-05 | 4.614394 | -1.06 | down |
| chr4 | CCDC149 | 24888489 | 24888889 | 401 | * | 6 | 4.4 | 6.74 | -2.3 | 7.44E-23 | 4.33E-20 | 19.36351 | -2.3 | down |
| chr4 | SOD3 | 24823881 | 24824281 | 401 | * | 3.32 | 1.18 | 4.15 | -2.34 | 1.19E-05 | 0.000347 | 3.459671 | -2.34 | down |
| chr4 | SOD3 | 24736120 | 24736520 | 401 | * | 4.73 | 3.34 | 5.43 | -1.92 | 3.69E-09 | 2.87E-07 | 6.542118 | -1.92 | down |
| chr4 | DHX15 | 24570688 | 24571088 | 401 | * | 4.66 | 3.82 | 5.2 | -1.01 | 2.85E-05 | 0.000719 | 3.143271 | -1.01 | down |
| chr4 | DHX15 | 24537028 | 24537428 | 401 | * | 5.67 | 4.69 | 6.25 | -1.4 | 6.53E-11 | 7.26E-09 | 8.139063 | -1.4 | down |
| chr4 | MIR573 | 24514774 | 24515174 | 401 | * | 4.45 | 3.53 | 5.01 | -1.01 | 0.000062 | 0.00137 | 2.863279 | -1.01 | down |
| chr11 | LUZP2 | 24453237 | 24453637 | 401 | * | 4.27 | 0.37 | 5.22 | -4.24 | 3.91E-10 | 3.71E-08 | 7.430626 | -4.24 | down |
| chr11 | LUZP2 | 24198454 | 24198854 | 401 | * | 7.28 | 5.3 | 8.08 | -2.78 | 8.81E-63 | 7.31E-59 | 58.13608 | -2.78 | down |
| chr14 | DHRS2 | 24171746 | 24172146 | 401 | * | 8.06 | 4.84 | 8.98 | -4.12 | ######## | ######## | 121.2097 | -4.12 | down |
| chr9 | ELAVL2 | 23831351 | 23831751 | 401 | * | 4.9 | 3.99 | 5.45 | -1.14 | 2.16E-06 | 8.07E-05 | 4.093126 | -1.14 | down |
| chr9 | ELAVL2 | 23826220 | 23826620 | 401 | * | 4.13 | 2.14 | 4.93 | -2.53 | 2.28E-08 | 1.47E-06 | 5.832683 | -2.53 | down |
| chr21 | LINC00308 | 23809574 | 23809974 | 401 | * | 6.05 | 5.03 | 6.64 | -1.5 | 1.32E-13 | 2.33E-11 | 10.63264 | -1.5 | down |
| chr11 | CCDC179 | 23550723 | 23551123 | 401 | * | 3.85 | 0.37 | 4.78 | -3.81 | 2.43E-08 | 1.56E-06 | 5.806875 | -3.81 | down |
| chr2 | KLHL29 | 23375432 | 23375832 | 401 | * | 4.05 | 2.7 | 4.73 | -1.59 | 5.12E-06 | 0.000171 | 3.767004 | -1.59 | down |
| chr4 | PPARGC1A | 23373315 | 23373715 | 401 | * | 3.81 | 1.53 | 4.66 | -2.79 | 1.09E-07 | 5.97E-06 | 5.224026 | -2.79 | down |
| chr4 | PPARGC1A | 23333850 | 23334250 | 401 | * | 4.14 | 1.55 | 5.01 | -3.18 | 1.07E-09 | 9.38E-08 | 7.027797 | -3.18 | down |
| chr6 | HDGFL1 | 23309881 | 23310281 | 401 | * | 4.84 | 3.31 | 5.57 | -2.12 | 9.49E-11 | 1.03E-08 | 7.987163 | -2.12 | down |
| chr11 | CCDC179 | 23205388 | 23205788 | 401 | * | 3.09 | 0.03 | 4.01 | -2.92 | 1.78E-05 | 0.000487 | 3.312471 | -2.92 | down |
| chr11 | CCDC179 | 22931149 | 22931549 | 401 | * | 5.83 | 2.45 | 6.76 | -4.15 | 6.59E-29 | 5.76E-26 | 25.23958 | -4.15 | down |
| chr10 | PIP4K2A | 22927798 | 22928198 | 401 | * | 3.55 | 1.37 | 4.38 | -2.57 | 1.84E-06 | 7.02E-05 | 4.153663 | -2.57 | down |

| | | | | | | | | | | | | | | |
|---|---|---|---|---|---|---|---|---|---|---|---|---|---|---|
| chr5 | CDH12 | 22907199 | 22907599 | 401 | * | 4.61 | 3.72 | 5.16 | -1.05 | 3.36E-05 | 0.000821 | 3.085657 | -1.05 | down |
| chr5 | CDH12 | 22853174 | 22853574 | 401 | * | 4.62 | 2.76 | 5.41 | -2.51 | 1.26E-10 | 1.35E-08 | 7.869666 | -2.51 | down |
| chr5 | CDH12 | 22757966 | 22758366 | 401 | * | 3.66 | 0.02 | 4.6 | -3.81 | 3.28E-07 | 1.57E-05 | 4.8041 | -3.81 | down |
| chr5 | CDH12 | 22754193 | 22754593 | 401 | * | 5.35 | 1.39 | 6.31 | -4.61 | 1.44E-19 | 5.61E-17 | 16.25104 | -4.61 | down |
| chr5 | CDH12 | 22591441 | 22591841 | 401 | * | 4.09 | 0.97 | 5.01 | -3.64 | 8.81E-10 | 7.91E-08 | 7.101824 | -3.64 | down |
| chr12 | ST8SIA1 | 22554684 | 22555084 | 401 | * | 5.46 | 4.58 | 6 | -1.2 | 3.24E-08 | 2.04E-06 | 5.69037 | -1.2 | down |
| chr4 | ADGRA3 | 22515291 | 22515691 | 401 | * | 4.48 | 2.61 | 5.26 | -2.49 | 3.58E-10 | 3.45E-08 | 7.462181 | -2.49 | down |
| chr9 | DMRTA1 | 22416642 | 22417042 | 401 | * | 3.39 | 1.83 | 4.13 | -1.44 | 0.000121 | 0.00239 | 2.621602 | -1.44 | down |
| chr10 | DNAJC1 | 22345249 | 22345649 | 401 | * | 5.39 | 3.24 | 6.21 | -2.89 | 8.96E-20 | 3.58E-17 | 16.44612 | -2.89 | down |
| chr16 | EEF2K | 22239053 | 22239453 | 401 | * | 4.02 | 2.86 | 4.65 | -1.27 | 3.61E-05 | 0.000875 | 3.057992 | -1.27 | down |
| chr9 | CDKN2B-AS1 | 22148094 | 22148494 | 401 | * | 5.1 | 4.29 | 5.62 | -1.02 | 6.16E-06 | 0.0002 | 3.69897 | -1.02 | down |
| chr21 | LINC00320 | 22092314 | 22092714 | 401 | * | 3.97 | 1 | 4.87 | -3.49 | 6.39E-09 | 4.71E-07 | 6.326979 | -3.49 | down |
| chr5 | PMCHL1 | 21806944 | 21807344 | 401 | * | 4.22 | 2.7 | 4.94 | -1.98 | 1.73E-07 | 8.97E-06 | 5.047208 | -1.98 | down |
| chrX | SMPX | 21786352 | 21786752 | 401 | * | 5.15 | 3.24 | 5.94 | -2.62 | 1.12E-15 | 2.63E-13 | 12.58004 | -2.62 | down |
| chrX | SMPX | 21740874 | 21741274 | 401 | * | 2.91 | 0.94 | 3.71 | -1.36 | 0.000407 | 0.0063 | 2.200659 | -1.36 | down |
| chr21 | LINC00320 | 21200089 | 21200489 | 401 | * | 4.63 | 2.68 | 5.42 | -2.6 | 2.84E-11 | 3.39E-09 | 8.4698 | -2.6 | down |
| chr10 | NEBL | 21093056 | 21093456 | 401 | * | 4.3 | 3.28 | 4.89 | -1.13 | 3.32E-05 | 0.000813 | 3.089909 | -1.13 | down |
| chr21 | LINC00320 | 20998156 | 20998556 | 401 | * | 5.24 | 3.8 | 5.95 | -2.06 | 1.18E-12 | 1.79E-10 | 9.747147 | -2.06 | down |
| chr22 | ZNF74 | 20729309 | 20729709 | 401 | * | 4.32 | 3.28 | 4.92 | -1.17 | 2.65E-05 | 0.000676 | 3.170053 | -1.17 | down |
| chr2 | RHOB | 20601305 | 20601705 | 401 | * | 6.17 | 5.26 | 6.72 | -1.34 | 2.65E-13 | 4.44E-11 | 10.35262 | -1.34 | down |
| chr4 | MIR218-1 | 20512070 | 20512470 | 401 | * | 5.04 | 4.06 | 5.62 | -1.3 | 1.23E-07 | 6.62E-06 | 5.179142 | -1.3 | down |
| chr3 | SGO1 | 20459358 | 20459758 | 401 | * | 4.4 | 3.46 | 4.96 | -1.03 | 6.64E-05 | 0.00146 | 2.835647 | -1.03 | down |
| chr4 | SLIT2 | 20251675 | 20252075 | 401 | * | 6.16 | 5.36 | 6.67 | -1.16 | 1.85E-10 | 1.9E-08 | 7.721246 | -1.16 | down |
| chr6 | MBOAT1 | 20069145 | 20069545 | 401 | * | 4.12 | 2.46 | 4.88 | -2.16 | 1.49E-07 | 7.88E-06 | 5.103474 | -2.16 | down |
| chr22 | TANGO2 | 20005619 | 20006019 | 401 | * | 6.09 | 5.25 | 6.61 | -1.21 | 4.55E-11 | 5.24E-09 | 8.280669 | -1.21 | down |
| chr6 | ID4 | 19995887 | 19996287 | 401 | * | 7.1 | 6.27 | 7.63 | -1.29 | 6.71E-21 | 3.18E-18 | 17.49757 | -1.29 | down |
| chr14 | BMS1P18 | 19879228 | 19879628 | 401 | * | 4.9 | 3.75 | 5.54 | -1.57 | 2.09E-08 | 1.36E-06 | 5.866461 | -1.57 | down |
| chr6 | ID4 | 19860342 | 19860742 | 401 | * | 4.8 | 3.22 | 5.54 | -2.16 | 2.18E-10 | 2.2E-08 | 7.657577 | -2.16 | down |

| | | | | | | | | | | | | | | |
|---|---|---|---|---|---|---|---|---|---|---|---|---|---|---|
| chrX | SH3KBP1 | 19846897 | 19847297 | 401 | * | 4.93 | 4.02 | 5.49 | -1.16 | 1.32E-06 | 0.000053 | 4.275724 | -1.16 | down |
| chrX | SH3KBP1 | 19840838 | 19841238 | 401 | * | 6.3 | 5.5 | 6.82 | -1.19 | 2.74E-12 | 3.94E-10 | 9.404504 | -1.19 | down |
| chrX | SH3KBP1 | 19829717 | 19830117 | 401 | * | 3.69 | 2.45 | 4.34 | -1.16 | 0.000158 | 0.00296 | 2.528708 | -1.16 | down |
| chr19 | ZNF14 | 19821582 | 19821982 | 401 | * | 4.3 | 3.32 | 4.88 | -1.07 | 5.39E-05 | 0.00122 | 2.91364 | -1.07 | down |
| chrY | FAM224B | 19758695 | 19759095 | 401 | * | 4.55 | 3.42 | 5.18 | -1.43 | 8.56E-07 | 3.64E-05 | 4.438899 | -1.43 | down |
| chr19 | PBX4 | 19732949 | 19733349 | 401 | * | 6.38 | 5 | 7.07 | -2.03 | 2.36E-25 | 1.65E-22 | 21.78252 | -2.03 | down |
| chr9 | ACER2 | 19403264 | 19403664 | 401 | * | 4.65 | 3.71 | 5.21 | -1.07 | 2.75E-05 | 0.000696 | 3.157391 | -1.07 | down |
| chr17 | RNF112 | 19337859 | 19338259 | 401 | * | 6.01 | 5.21 | 6.53 | -1.15 | 3.59E-10 | 3.45E-08 | 7.462181 | -1.15 | down |
| chr3 | SATB1 | 18425892 | 18426292 | 401 | * | 3.94 | 1.81 | 4.76 | -2.62 | 1.59E-07 | 8.32E-06 | 5.079877 | -2.62 | down |
| chr4 | LCORL | 18166941 | 18167341 | 401 | * | 2.94 | 0.41 | 3.81 | -2.18 | 8.13E-05 | 0.00172 | 2.764472 | -2.18 | down |
| chr22 | ATP6V1E1 | 18110482 | 18110882 | 401 | * | 4.58 | 3.7 | 5.12 | -1.03 | 3.06E-05 | 0.00076 | 3.119186 | -1.03 | down |
| chr12 | MIR3974 | 17920696 | 17921096 | 401 | * | 2.9 | 0.64 | 3.74 | -1.65 | 0.000235 | 0.00406 | 2.391474 | -1.65 | down |
| chr4 | MED28 | 17670429 | 17670829 | 401 | * | 6.24 | 5.47 | 6.74 | -1.13 | 4.02E-11 | 4.68E-09 | 8.329754 | -1.13 | down |
| chr4 | LAP3 | 17549393 | 17549793 | 401 | * | 6.69 | 5.87 | 7.21 | -1.25 | 5.52E-16 | 1.37E-13 | 12.86328 | -1.25 | down |
| chr12 | SKP1P2 | 17296830 | 17297230 | 401 | * | 4.72 | 2.81 | 5.51 | -2.55 | 1.11E-11 | 1.42E-09 | 8.847712 | -2.55 | down |
| chr4 | QDPR | 17272171 | 17272571 | 401 | * | 6.35 | 5.42 | 6.91 | -1.37 | 4.04E-14 | 7.70E-12 | 11.11351 | -1.37 | down |
| chr4 | QDPR | 17260441 | 17260841 | 401 | * | 6.44 | 5.71 | 6.92 | -1.07 | 5.30E-11 | 5.98E-09 | 8.223299 | -1.07 | down |
| chr12 | SKP1P2 | 16955216 | 16955616 | 401 | * | 3.45 | 0 | 4.38 | -3.55 | 1.53E-06 | 6.03E-05 | 4.219683 | -3.55 | down |
| chr12 | LMO3 | 16939848 | 16940248 | 401 | * | 4.91 | 3.75 | 5.54 | -1.58 | 2.05E-08 | 1.34E-06 | 5.872895 | -1.58 | down |
| chr1 | MIR3675 | 16872606 | 16873006 | 401 | * | 4.34 | 3.17 | 4.97 | -1.38 | 6.06E-06 | 0.000197 | 3.705534 | -1.38 | down |
| chr12 | LMO3 | 16872066 | 16872466 | 401 | * | 4.2 | 1.34 | 5.09 | -3.41 | 2.33E-10 | 2.34E-08 | 7.630784 | -3.41 | down |
| chr12 | LMO3 | 16833309 | 16833709 | 401 | * | 6.8 | 4.58 | 7.63 | -3.01 | 7.57E-50 | 3.35E-46 | 45.47496 | -3.01 | down |
| chr12 | LMO3 | 16832269 | 16832669 | 401 | * | 7.33 | 5.19 | 8.15 | -2.95 | 5.88E-69 | 6.51E-65 | 64.18642 | -2.95 | down |
| chr12 | LMO3 | 16796324 | 16796724 | 401 | * | 4.49 | 2.77 | 5.25 | -2.3 | 3.96E-09 | 3.06E-07 | 6.514279 | -2.3 | down |
| chr12 | LMO3 | 16793456 | 16793856 | 401 | * | 4.3 | 2.3 | 5.11 | -2.64 | 1.02E-09 | 9.02E-08 | 7.044793 | -2.64 | down |
| chr12 | LMO3 | 16757160 | 16757560 | 401 | * | 6.14 | 4.05 | 6.96 | -2.86 | 2.84E-31 | 2.85E-28 | 27.54516 | -2.86 | down |
| chr12 | MGST1 | 16611173 | 16611573 | 401 | * | 3.93 | 2.06 | 4.72 | -2.36 | 2.63E-07 | 1.29E-05 | 4.88941 | -2.36 | down |
| chr12 | MGST1 | 16606391 | 16606791 | 401 | * | 5.07 | 3.44 | 5.82 | -2.3 | 5.99E-13 | 9.63E-11 | 10.01637 | -2.3 | down |

| | | | | | | | | | | | | | | |
|---|---|---|---|---|---|---|---|---|---|---|---|---|---|---|
| chrX | S100G | 16604866 | 16605266 | 401 | * | 3.33 | 1.83 | 4.04 | -1.3 | 0.000196 | 0.00353 | 2.452225 | -1.3 | down |
| chr12 | MGST1 | 16600545 | 16600945 | 401 | * | 5.26 | 3.14 | 6.08 | -2.85 | 1.59E-17 | 4.96E-15 | 14.30452 | -2.85 | down |
| chr12 | MGST1 | 16586227 | 16586627 | 401 | * | 4.53 | 2.71 | 5.31 | -2.46 | 3.37E-10 | 3.26E-08 | 7.486782 | -2.46 | down |
| chr5 | ZNF622 | 16418518 | 16418918 | 401 | * | 4.43 | 2.47 | 5.23 | -2.61 | 2.94E-10 | 2.88E-08 | 7.540608 | -2.61 | down |
| chr3 | GALNT15 | 16200240 | 16200640 | 401 | * | 4.29 | 2.48 | 5.06 | -2.41 | 6.54E-09 | 4.8E-07 | 6.318759 | -2.41 | down |
| chr2 | MYCN | 16149040 | 16149440 | 401 | * | 3.54 | 1.95 | 4.27 | -1.63 | 4.49E-05 | 0.00105 | 2.978811 | -1.63 | down |
| chr2 | MYCN | 16142537 | 16142937 | 401 | * | 4.91 | 3.93 | 5.49 | -1.26 | 5.09E-07 | 2.31E-05 | 4.636388 | -1.26 | down |
| chr4 | TAPT1 | 16139574 | 16139974 | 401 | * | 5.53 | 4.77 | 6.03 | -1.04 | 2.86E-07 | 1.39E-05 | 4.856985 | -1.04 | down |
| chr4 | BST1 | 15702977 | 15703377 | 401 | * | 4.67 | 3.18 | 5.39 | -2.04 | 2.68E-09 | 2.16E-07 | 6.665546 | -2.04 | down |
| chr3 | HACL1 | 15619742 | 15620142 | 401 | * | 4.63 | 3.44 | 5.27 | -1.53 | 3.71E-07 | 1.76E-05 | 4.754487 | -1.53 | down |
| chr21 | LIPI | 15557962 | 15558362 | 401 | * | 5.28 | 4.15 | 5.91 | -1.57 | 4.43E-10 | 4.18E-08 | 7.378824 | -1.57 | down |
| chr4 | CC2D2A | 15550484 | 15550884 | 401 | * | 3.98 | 2.48 | 4.7 | -1.81 | 3.33E-06 | 0.000118 | 3.928118 | -1.81 | down |
| chr8 | TUSC3 | 15540482 | 15540882 | 401 | * | 4.85 | 3.47 | 5.54 | -1.91 | 8.54E-10 | 7.69E-08 | 7.114074 | -1.91 | down |
| chr8 | TUSC3 | 15533723 | 15534123 | 401 | * | 4.01 | 2.28 | 4.77 | -2.2 | 3.95E-07 | 1.85E-05 | 4.732828 | -2.2 | down |
| chr20 | MACROD2 | 15528395 | 15528795 | 401 | * | 3.1 | 0.41 | 3.98 | -2.65 | 0.000021 | 0.000558 | 3.253366 | -2.65 | down |
| chrY | DDX3Y | 15112249 | 15112649 | 401 | * | 5.86 | 0.94 | 6.83 | -5.45 | 8.17E-22 | 4.34E-19 | 18.36251 | -5.45 | down |
| chrY | DDX3Y | 15059468 | 15059868 | 401 | * | 3.94 | 0 | 4.91 | -4.34 | 2.13E-07 | 1.08E-05 | 4.966576 | -4.34 | down |
| chr9 | FREM1 | 14927015 | 14927415 | 401 | * | 5.84 | 4.75 | 6.45 | -1.61 | 1.41E-13 | 2.46E-11 | 10.60906 | -1.61 | down |
| chr3 | FGD5 | 14926046 | 14926446 | 401 | * | 5.33 | 4 | 6.01 | -1.91 | 2.86E-12 | 4.09E-10 | 9.388277 | -1.91 | down |
| chr4 | CPEB2-DT | 14924277 | 14924677 | 401 | * | 4.38 | 2.64 | 5.15 | -2.35 | 3.59E-09 | 2.81E-07 | 6.551294 | -2.35 | down |
| chr9 | FREM1 | 14906640 | 14907040 | 401 | * | 3.92 | 2.21 | 4.68 | -2.16 | 9.71E-07 | 4.04E-05 | 4.393619 | -2.16 | down |
| chr4 | CPEB2-DT | 14841822 | 14842222 | 401 | * | 7 | 6.19 | 7.51 | -1.24 | 6.93E-19 | 2.52E-16 | 15.5986 | -1.24 | down |
| chr5 | TRIO | 14281840 | 14282240 | 401 | * | 5.45 | 4.18 | 6.12 | -1.83 | 1.81E-12 | 2.68E-10 | 9.571865 | -1.83 | down |
| chr21 | ANKRD30BP2 | 14273815 | 14274215 | 401 | * | 7.07 | 5.29 | 7.85 | -2.53 | 2.83E-50 | 1.34E-46 | 45.8729 | -2.53 | down |
| chr21 | ANKRD30BP2 | 14271038 | 14271438 | 401 | * | 8.1 | 7.16 | 8.66 | -1.48 | 4.38E-47 | 1.32E-43 | 42.87943 | -1.48 | down |
| chr5 | TRIO | 14268269 | 14268669 | 401 | * | 4.59 | 3.48 | 5.21 | -1.4 | 1.15E-06 | 4.71E-05 | 4.326979 | -1.4 | down |
| chr5 | TRIO | 14264587 | 14264987 | 401 | * | 4.79 | 3.56 | 5.44 | -1.62 | 3.33E-08 | 2.07E-06 | 5.68403 | -1.62 | down |
| chr3 | XPC | 14204948 | 14205348 | 401 | * | 5.53 | 4.74 | 6.04 | -1.05 | 7.12E-07 | 3.11E-05 | 4.50724 | -1.05 | down |

| | | | | | | | | | | | | | | |
|---|---|---|---|---|---|---|---|---|---|---|---|---|---|---|
| chr5 | TRIO | 14145144 | 14145544 | 401 | * | 4.63 | 3.55 | 5.24 | -1.33 | 1.71E-06 | 6.63E-05 | 4.178486 | -1.33 | down |
| chr5 | TRIO | 14142862 | 14143262 | 401 | * | 5.31 | 4.51 | 5.83 | -1.08 | 5.47E-07 | 2.46E-05 | 4.609065 | -1.08 | down |
| chr21 | ANKRD30BP2 | 14120151 | 14120551 | 401 | * | 4.02 | 2.77 | 4.68 | -1.41 | 1.67E-05 | 0.000462 | 3.335358 | -1.41 | down |
| chr21 | ANKRD30BP2 | 14027144 | 14027544 | 401 | * | 6.49 | 4.79 | 7.24 | -2.42 | 6.89E-33 | 8.63E-30 | 29.06399 | -2.42 | down |
| chr5 | DNAH5 | 14011554 | 14011954 | 401 | * | 6.69 | 5.96 | 7.17 | -1.11 | 1.54E-12 | 2.30E-10 | 9.638272 | -1.11 | down |
| chr7 | ETV1 | 13989490 | 13989890 | 401 | * | 4.8 | 3.25 | 5.53 | -2.15 | 1.28E-10 | 1.36E-08 | 7.866461 | -2.15 | down |
| chr21 | ANKRD30BP2 | 13979976 | 13980376 | 401 | * | 3.87 | 0 | 4.85 | -4.75 | 1.85E-07 | 9.49E-06 | 5.022734 | -4.75 | down |
| chr21 | ANKRD30BP2 | 13973833 | 13974233 | 401 | * | 3.21 | 1.15 | 4.03 | -1.98 | 5.19E-05 | 0.00119 | 2.924453 | -1.98 | down |
| chr8 | SGCZ | 13952179 | 13952579 | 401 | * | 3.41 | 2.06 | 4.09 | -1.09 | 0.000397 | 0.00619 | 2.208309 | -1.09 | down |
| chr8 | C8orf48 | 13903023 | 13903423 | 401 | * | 6.39 | 5.47 | 6.94 | -1.36 | 2.43E-15 | 5.48E-13 | 12.26122 | -1.36 | down |
| chr8 | C8orf48 | 13882365 | 13882765 | 401 | * | 7.83 | 6.75 | 8.45 | -1.67 | 4.95E-48 | 1.83E-44 | 43.73755 | -1.67 | down |
| chr2 | LOC100506474 | 13696621 | 13697021 | 401 | * | 4.43 | 3.37 | 5.04 | -1.25 | 7.71E-06 | 0.00024 | 3.619789 | -1.25 | down |
| chr3 | HDAC11 | 13537855 | 13538255 | 401 | * | 5.75 | 4.06 | 6.5 | -2.39 | 3.04E-20 | 1.27E-17 | 16.8962 | -2.39 | down |
| chr2 | LOC100506474 | 13523178 | 13523578 | 401 | * | 4.59 | 3.71 | 5.14 | -1.04 | 3.04E-05 | 0.000758 | 3.120331 | -1.04 | down |
| chr4 | LINC01097 | 13522579 | 13522979 | 401 | * | 3.53 | 2.24 | 4.2 | -1.04 | 0.000409 | 0.00632 | 2.199283 | -1.04 | down |
| chr1 | PRAMEF20 | 13513815 | 13514215 | 401 | * | 4.33 | 3.12 | 4.98 | -1.49 | 3.08E-06 | 0.00011 | 3.958607 | -1.49 | down |
| chr6 | GFOD1 | 13454848 | 13455248 | 401 | * | 5.26 | 4.13 | 5.88 | -1.58 | 5.05E-10 | 4.72E-08 | 7.326058 | -1.58 | down |
| chr6 | GFOD1 | 13407797 | 13408197 | 401 | * | 6.98 | 6.11 | 7.52 | -1.34 | 1.58E-20 | 7.04E-18 | 17.15243 | -1.34 | down |
| chr6 | TBC1D7 | 13355452 | 13355852 | 401 | * | 6.69 | 5.89 | 7.2 | -1.21 | 1.66E-15 | 3.83E-13 | 12.4168 | -1.21 | down |
| chr19 | NFIX | 13167454 | 13167854 | 401 | * | 5.93 | 5.23 | 6.4 | -1.01 | 2.32E-08 | 1.5E-06 | 5.823909 | -1.01 | down |
| chrX | PRPS2 | 12771441 | 12771841 | 401 | * | 5.3 | 4.13 | 5.94 | -1.65 | 9.70E-11 | 1.06E-08 | 7.974694 | -1.65 | down |
| chr3 | RAF1 | 12750546 | 12750946 | 401 | * | 4.69 | 3.6 | 5.3 | -1.38 | 7.52E-07 | 3.25E-05 | 4.488117 | -1.38 | down |
| chr4 | HS3ST1 | 12456984 | 12457384 | 401 | * | 3.65 | 1.52 | 4.48 | -2.52 | 1.51E-06 | 5.98E-05 | 4.223299 | -2.52 | down |
| chr19 | ZNF563 | 12440933 | 12441333 | 401 | * | 6.61 | 4.65 | 7.41 | -2.73 | 6.83E-41 | 1.29E-37 | 36.88941 | -2.73 | down |
| chr16 | SNX29 | 12420664 | 12421064 | 401 | * | 5.94 | 5.23 | 6.42 | -1.03 | 1.48E-08 | 1.01E-06 | 5.995679 | -1.03 | down |
| chr4 | HS3ST1 | 12401185 | 12401585 | 401 | * | 3.8 | 1.18 | 4.68 | -3.13 | 4.39E-08 | 2.64E-06 | 5.578396 | -3.13 | down |
| chr19 | ZNF44 | 12401079 | 12401479 | 401 | * | 7.22 | 5.7 | 7.94 | -2.21 | 1.80E-46 | 5.19E-43 | 42.28483 | -2.21 | down |
| chr4 | HS3ST1 | 12399796 | 12400196 | 401 | * | 4.02 | 0.36 | 4.96 | -3.98 | 5.04E-09 | 3.79E-07 | 6.421361 | -3.98 | down |

| | | | | | | | | | | | | | | |
|---|---|---|---|---|---|---|---|---|---|---|---|---|---|---|
| chr4 | HS3ST1 | 12388946 | 12389346 | 401 | * | 3.73 | 0 | 4.67 | -3.9 | 1.73E-07 | 8.95E-06 | 5.048177 | -3.9 | down |
| chr3 | SYN2 | 12004144 | 12004544 | 401 | * | 4.38 | 3.2 | 5.02 | -1.47 | 2.16E-06 | 8.06E-05 | 4.093665 | -1.47 | down |
| chr19 | ZNF439 | 11964774 | 11965174 | 401 | * | 4.12 | 2.88 | 4.78 | -1.41 | 1.09E-05 | 0.000322 | 3.492144 | -1.41 | down |
| chr10 | ECHDC3 | 11792842 | 11793242 | 401 | * | 3.17 | 1.68 | 3.88 | -1.06 | 0.000625 | 0.00895 | 2.048177 | -1.06 | down |
| chr3 | VGLL4 | 11596376 | 11596776 | 401 | * | 4.08 | 2.68 | 4.77 | -1.67 | 3.63E-06 | 0.000126 | 3.899629 | -1.67 | down |
| chr19 | ELAVL3 | 11578425 | 11578825 | 401 | * | 4.29 | 3.26 | 4.88 | -1.12 | 0.00004 | 0.000954 | 3.020452 | -1.12 | down |
| chr8 | BLK | 11347284 | 11347684 | 401 | * | 5.57 | 4.72 | 6.11 | -1.18 | 1.56E-08 | 1.05E-06 | 5.978811 | -1.18 | down |
| chr1 | MTOR | 11305091 | 11305491 | 401 | * | 4.38 | 2.46 | 5.18 | -2.55 | 6.46E-10 | 5.92E-08 | 7.227678 | -2.55 | down |
| chr6 | MAK | 10855258 | 10855658 | 401 | * | 5.69 | 4.31 | 6.39 | -2.02 | 1.80E-16 | 4.79E-14 | 13.31966 | -2.02 | down |
| chr6 | MAK | 10853909 | 10854309 | 401 | * | 8.39 | 7.56 | 8.91 | -1.32 | 1.50E-47 | 4.75E-44 | 43.32331 | -1.32 | down |
| chr7 | PER4 | 9937935 | 9938335 | 401 | * | 3.7 | 1.41 | 4.54 | -2.79 | 3.06E-07 | 1.48E-05 | 4.829738 | -2.79 | down |
| chr20 | PLCB4 | 9392985 | 9393385 | 401 | * | 4.81 | 3.98 | 5.34 | -1.02 | 1.73E-05 | 0.000476 | 3.322393 | -1.02 | down |
| chr1 | MIR34A | 9239642 | 9240042 | 401 | * | 4.56 | 3.02 | 5.28 | -2.09 | 5.13E-09 | 3.85E-07 | 6.414539 | -2.09 | down |
| chr8 | PPP1R3B | 9087841 | 9088241 | 401 | * | 3.28 | 1.35 | 4.07 | -1.93 | 4.29E-05 | 0.00101 | 2.995679 | -1.93 | down |
| chr17 | CCDC42 | 8638698 | 8639098 | 401 | * | 5.47 | 4.69 | 5.97 | -1.04 | 4.55E-07 | 0.000021 | 4.677781 | -1.04 | down |
| chr4 | TRMT44 | 8495783 | 8496183 | 401 | * | 5.01 | 4.17 | 5.54 | -1.08 | 2.33E-06 | 8.64E-05 | 4.063486 | -1.08 | down |
| chr11 | LMO1 | 8384528 | 8384928 | 401 | * | 3.34 | 1.75 | 4.08 | -1.5 | 0.000116 | 0.00231 | 2.636388 | -1.5 | down |
| chr12 | ZNF705A | 8328759 | 8329159 | 401 | * | 5.77 | 4.77 | 6.35 | -1.41 | 1.07E-10 | 1.15E-08 | 7.939302 | -1.41 | down |
| chr12 | ZNF705A | 8320168 | 8320568 | 401 | * | 5.14 | 3.7 | 5.85 | -2.04 | 4.42E-12 | 6.05E-10 | 9.218245 | -2.04 | down |
| chr4 | SH3TC1 | 8183262 | 8183662 | 401 | * | 4.67 | 3.81 | 5.2 | -1.02 | 2.29E-05 | 0.000599 | 3.222573 | -1.02 | down |
| chr11 | RIC3 | 8168493 | 8168893 | 401 | * | 4.86 | 1.8 | 5.77 | -3.71 | 1.11E-15 | 2.61E-13 | 12.58336 | -3.71 | down |
| chr4 | ABLIM2 | 8079067 | 8079467 | 401 | * | 6.49 | 5.75 | 6.98 | -1.12 | 1.45E-12 | 2.18E-10 | 9.661544 | -1.12 | down |
| chr1 | CAMTA1 | 7586195 | 7586595 | 401 | * | 6.22 | 5.52 | 6.69 | -1.02 | 2.85E-09 | 2.28E-07 | 6.642065 | -1.02 | down |
| chr1 | CAMTA1 | 7572694 | 7573094 | 401 | * | 6.33 | 5.63 | 6.8 | -1.04 | 1.92E-10 | 1.97E-08 | 7.705534 | -1.04 | down |
| chr10 | SFMBT2 | 7412396 | 7412796 | 401 | * | 4.58 | 3.39 | 5.22 | -1.53 | 3.78E-07 | 1.78E-05 | 4.74958 | -1.53 | down |
| chr10 | SFMBT2 | 7408414 | 7408814 | 401 | * | 4.56 | 3.52 | 5.16 | -1.26 | 4.73E-06 | 0.000159 | 3.798603 | -1.26 | down |
| chr10 | SFMBT2 | 7389898 | 7390298 | 401 | * | 3.01 | 1.39 | 3.76 | -1.14 | 0.000564 | 0.00821 | 2.085657 | -1.14 | down |
| chrY | PRKY | 7288514 | 7288914 | 401 | * | 4.51 | 2.77 | 5.28 | -2.35 | 6.58E-10 | 6E-08 | 7.221849 | -2.35 | down |

| | | | | | | | | | | | | | | |
|---|---|---|---|---|---|---|---|---|---|---|---|---|---|---|
| chr6 | SSR1 | 7261163 | 7261563 | 401 | * | 6.98 | 6.28 | 7.45 | -1.08 | 4.78E-15 | 1.03E-12 | 11.98716 | -1.08 | down |
| chr10 | SFMBT2 | 7252009 | 7252409 | 401 | * | 5.36 | 4.36 | 5.94 | -1.38 | 2.70E-09 | 2.17E-07 | 6.66354 | -1.38 | down |
| chr17 | CLDN7 | 7169054 | 7169454 | 401 | * | 5.7 | 4.84 | 6.23 | -1.18 | 1.11E-08 | 7.77E-07 | 6.109579 | -1.18 | down |
| chr8 | DEFB109B | 7070403 | 7070803 | 401 | * | 4.59 | 3.66 | 5.15 | -1.07 | 2.43E-05 | 0.000631 | 3.199971 | -1.07 | down |
| chr11 | ZNF214 | 7020174 | 7020574 | 401 | * | 4.81 | 3.1 | 5.57 | -2.37 | 1.11E-11 | 1.42E-09 | 8.847712 | -2.37 | down |
| chr11 | OR2D2 | 6926154 | 6926554 | 401 | * | 3.65 | 1.57 | 4.47 | -2.54 | 9.17E-07 | 3.86E-05 | 4.413413 | -2.54 | down |
| chr1 | DNAJC11 | 6743673 | 6744073 | 401 | * | 6.52 | 5.77 | 7.01 | -1.12 | 1.55E-12 | 2.32E-10 | 9.634512 | -1.12 | down |
| chr6 | LY86 | 6712510 | 6712910 | 401 | * | 5.22 | 3.47 | 5.98 | -2.44 | 4.14E-15 | 9.03E-13 | 12.04431 | -2.44 | down |
| chr11 | DCHS1 | 6690378 | 6690778 | 401 | * | 3.12 | 1.02 | 3.94 | -2.01 | 6.33E-05 | 0.0014 | 2.853872 | -2.01 | down |
| chr9 | GLDC | 6645664 | 6646064 | 401 | * | 4.15 | 2.89 | 4.81 | -1.46 | 9.06E-06 | 0.000275 | 3.560667 | -1.46 | down |
| chr1 | TNFRSF25 | 6520137 | 6520537 | 401 | * | 5 | 3.77 | 5.65 | -1.66 | 9.18E-09 | 6.52E-07 | 6.185752 | -1.66 | down |
| chr10 | LINC02649 | 6394038 | 6394438 | 401 | * | 3.06 | 1.16 | 3.86 | -1.57 | 0.000186 | 0.00337 | 2.47237 | -1.57 | down |
| chr11 | OR56B4 | 6127938 | 6128338 | 401 | * | 6.14 | 5.45 | 6.6 | -1.01 | 4.69E-09 | 3.57E-07 | 6.447332 | -1.01 | down |
| chr10 | IL2RA | 6074811 | 6075211 | 401 | * | 4.72 | 3.06 | 5.47 | -2.26 | 1.52E-10 | 1.6E-08 | 7.79588 | -2.26 | down |
| chr6 | NRN1 | 5892068 | 5892468 | 401 | * | 2.92 | 1.18 | 3.69 | -1.16 | 0.000621 | 0.00892 | 2.049635 | -1.16 | down |
| chr10 | GDI2 | 5853490 | 5853890 | 401 | * | 6.05 | 5.28 | 6.55 | -1.12 | 5.55E-10 | 5.15E-08 | 7.288193 | -1.12 | down |
| chr10 | GDI2 | 5852450 | 5852850 | 401 | * | 5.33 | 3.63 | 6.09 | -2.39 | 5.25E-16 | 1.31E-13 | 12.88273 | -2.39 | down |
| chr10 | ASB13 | 5692752 | 5693152 | 401 | * | 5.94 | 5.15 | 6.45 | -1.12 | 2.22E-09 | 1.81E-07 | 6.742321 | -1.12 | down |
| chr9 | PDCD1LG2 | 5529604 | 5530004 | 401 | * | 4.62 | 3.65 | 5.19 | -1.15 | 7.66E-06 | 0.000239 | 3.621602 | -1.15 | down |
| chr10 | NET1 | 5462541 | 5462941 | 401 | * | 3.93 | 2.7 | 4.59 | -1.34 | 3.18E-05 | 0.000784 | 3.105684 | -1.34 | down |
| chr6 | FARS2 | 5365424 | 5365824 | 401 | * | 4.99 | 4.18 | 5.51 | -1.02 | 7.07E-06 | 0.000223 | 3.651695 | -1.02 | down |
| chr19 | PTPRS | 5352474 | 5352874 | 401 | * | 4.67 | 3.7 | 5.24 | -1.17 | 5.45E-06 | 0.000179 | 3.747147 | -1.17 | down |
| chr6 | FARS2 | 5351806 | 5352206 | 401 | * | 3.95 | 2.61 | 4.64 | -1.51 | 1.28E-05 | 0.000366 | 3.436519 | -1.51 | down |
| chr11 | OR51V1 | 5226866 | 5227266 | 401 | * | 7.49 | 5.48 | 8.3 | -2.8 | 4.51E-74 | 7.48E-70 | 69.1261 | -2.8 | down |
| chr11 | OR51V1 | 5225500 | 5225900 | 401 | * | 7.44 | 6.73 | 7.92 | -1.12 | 1.56E-20 | 6.99E-18 | 17.15552 | -1.12 | down |
| chr9 | INSL6 | 5180540 | 5180940 | 401 | * | 5.78 | 4.58 | 6.42 | -1.74 | 9.06E-14 | 1.64E-11 | 10.78516 | -1.74 | down |
| chr8 | CSMD1 | 5135021 | 5135421 | 401 | * | 4.54 | 3.39 | 5.18 | -1.46 | 8.59E-07 | 3.65E-05 | 4.437707 | -1.46 | down |
| chr9 | MIR101-2 | 4865883 | 4866283 | 401 | * | 6.83 | 6.01 | 7.35 | -1.26 | 1.81E-17 | 5.50E-15 | 14.25964 | -1.26 | down |

| | | | | | | | | | | | | | | |
|---|---|---|---|---|---|---|---|---|---|---|---|---|---|---|
| chr2 | LINC01249 | 4668339 | 4668739 | 401 | * | 4.01 | 0 | 4.99 | -4.42 | 1.37E-07 | 7.29E-06 | 5.137272 | -4.42 | down |
| chr2 | LINC01249 | 4588633 | 4589033 | 401 | * | 3.37 | 0.46 | 4.27 | -3.17 | 2.5E-06 | 9.18E-05 | 4.037157 | -3.17 | down |
| chr4 | NSG1 | 4339548 | 4339948 | 401 | * | 5.72 | 4.28 | 6.43 | -2.09 | 1.80E-17 | 5.50E-15 | 14.25964 | -2.09 | down |
| chr19 | ZBTB7A | 4077790 | 4078190 | 401 | * | 4.66 | 3.58 | 5.26 | -1.32 | 1.79E-06 | 6.89E-05 | 4.161781 | -1.32 | down |
| chr10 | KLF6 | 3740301 | 3740701 | 401 | * | 6.36 | 5.45 | 6.91 | -1.35 | 1.24E-14 | 2.55E-12 | 11.59346 | -1.35 | down |
| chr20 | C20orf194 | 3352165 | 3352565 | 401 | * | 6.65 | 5.81 | 7.18 | -1.27 | 9.79E-16 | 2.34E-13 | 12.63078 | -1.27 | down |
| chr16 | ZNF213 | 3175509 | 3175909 | 401 | * | 6.16 | 5.4 | 6.65 | -1.1 | 3.23E-10 | 3.14E-08 | 7.50307 | -1.1 | down |
| chr3 | IL5RA | 3143359 | 3143759 | 401 | * | 4.67 | 3.57 | 5.28 | -1.41 | 6.27E-07 | 2.77E-05 | 4.55752 | -1.41 | down |
| chrX | ARSH | 2928638 | 2929038 | 401 | * | 4.31 | 2.53 | 5.09 | -2.35 | 7.23E-09 | 5.27E-07 | 6.278189 | -2.35 | down |
| chr6 | SERPINB1 | 2841737 | 2842137 | 401 | * | 6.88 | 4.88 | 7.69 | -2.79 | 9.95E-49 | 3.88E-45 | 44.41117 | -2.79 | down |
| chr17 | RAP1GAP2 | 2796146 | 2796546 | 401 | * | 5.18 | 4.28 | 5.72 | -1.18 | 2.21E-07 | 1.11E-05 | 4.954677 | -1.18 | down |
| chr9 | KCNV2 | 2744010 | 2744410 | 401 | * | 6.39 | 5.64 | 6.88 | -1.11 | 3.48E-11 | 4.09E-09 | 8.388277 | -1.11 | down |
| chr20 | NOP56 | 2604117 | 2604517 | 401 | * | 3.46 | 2.18 | 4.13 | -1.07 | 0.00036 | 0.00572 | 2.242604 | -1.07 | down |
| chr9 | SMARCA2 | 2027739 | 2028139 | 401 | * | 5.19 | 4.28 | 5.75 | -1.21 | 1.82E-07 | 9.34E-06 | 5.029653 | -1.21 | down |
| chr5 | IRX4 | 1882085 | 1882485 | 401 | * | 6.09 | 3.28 | 6.98 | -3.61 | 3.64E-33 | 4.64E-30 | 29.33348 | -3.61 | down |
| chr5 | IRX4 | 1876358 | 1876758 | 401 | * | 3.81 | 1.73 | 4.63 | -1.72 | 0.000162 | 0.00302 | 2.519993 | -1.72 | down |
| chr12 | MIR3649 | 1770002 | 1770402 | 401 | * | 3.91 | 2.84 | 4.52 | -1.02 | 0.00022 | 0.00386 | 2.413413 | -1.02 | down |
| chr16 | SSTR5 | 1120077 | 1120477 | 401 | * | 3.54 | 2.29 | 4.2 | -1.11 | 0.000264 | 0.00446 | 2.350665 | -1.11 | down |
| chr10 | LARP4B | 875504 | 875904 | 401 | * | 4.36 | 3.25 | 4.98 | -1.32 | 6.68E-06 | 0.000213 | 3.67162 | -1.32 | down |
| chr20 | SCRT2 | 688824 | 689224 | 401 | * | 4.39 | 3.34 | 4.99 | -1.23 | 0.000012 | 0.000349 | 3.457175 | -1.23 | down |
| chr7 | LOC442497 | 480601 | 481001 | 401 | * | 5.96 | 5.08 | 6.5 | -1.28 | 1.97E-11 | 2.45E-09 | 8.610834 | -1.28 | down |
| chr5 | AHRR | 317127 | 317527 | 401 | * | 4.73 | 3.62 | 5.35 | -1.43 | 3.06E-07 | 1.48E-05 | 4.829738 | -1.43 | down |

Table S4. Differentiially expression genes of RNA-seq.

| Differentiially expression genes of RNA seq | | | | | | | | | |
|---|---|---|---|---|---|---|---|---|---|
| Gene | WT-1_FPKM | WT-2_FPKM | WT-3_FPKM | beta0-1_FPKM | beta0-2_FPKM | beta0-3_FPKM | FDR | log2FC(HBB-KO vs WT) | sig. |
| ASS1 | 1.344944 | 1.636988 | 0.827691 | 2.961283 | 2.74165 | 2.752462 | 2.31E-06 | 1.028349 | up |
| ZNF704 | 0.014169 | 0.010164 | 0.01359 | 0.084708 | 0.113387 | 0.092248 | 3.62E-08 | 2.8078 | up |
| CYP27B1 | 3.072066 | 2.529321 | 3.069475 | 0.380955 | 0.376346 | 0.736043 | 2.22E-30 | -2.56641 | down |
| ITGAL | 0.33011 | 0.307927 | 0.282686 | 0.067809 | 0.06867 | 0.062749 | 5.85E-08 | -2.21124 | down |
| SMAD1 | 0.172385 | 0.053275 | 0.131195 | 1.015345 | 0.869793 | 0.93245 | 3.28E-16 | 3.014905 | up |

| | | | | | | | | | |
|---|---|---|---|---|---|---|---|---|---|
| ART4 | 1.26987 | 1.414566 | 1.44561 | 0.54204 | 0.492522 | 0.541175 | 1.50E-11 | -1.25736 | down |
| MTCL1 | 0.270596 | 0.259259 | 0.184199 | 0.855534 | 0.676219 | 1.042547 | 5.88E-12 | 1.658361 | up |
| SPHK2 | 17.74776 | 18.4866 | 17.52777 | 9.451357 | 9.136345 | 8.33047 | 1.22E-43 | -1.0601 | down |
| MSTRG.11246 | 0.376332 | 0.289171 | 0.600782 | 1.342405 | 1.060611 | 0.712789 | 0.005231 | 1.162723 | up |
| ATP6V0E2 | 7.217704 | 7.770843 | 5.779182 | 2.570817 | 2.504974 | 1.801277 | 6.25E-35 | -1.60771 | down |
| LTBP1 | 0.580418 | 0.505027 | 0.442425 | 1.349449 | 1.129696 | 1.599001 | 7.96E-14 | 1.301612 | up |
| ZFP82 | 0.186905 | 0.296905 | 0.333371 | 0.069308 | 0.097663 | 0.039627 | 1.85E-06 | -2.17212 | down |
| PM20D2 | 2.296651 | 2.336532 | 2.475881 | 8.0957 | 8.351847 | 8.718287 | ######## | 1.798052 | up |
| KHDRBS3 | 0.67661 | 0.541417 | 0.569241 | 0.227175 | 0.332907 | 0.213989 | 0.00316 | -1.24508 | down |
| DPYSL2 | 2.558247 | 2.922907 | 2.428299 | 1.172325 | 1.151313 | 1.297527 | 8.03E-19 | -1.15964 | down |
| IGFBP7 | 1.475326 | 1.681893 | 1.308875 | 4.872562 | 4.28363 | 4.879588 | 1.19E-18 | 1.615792 | up |
| IGFBP4 | 2.423979 | 2.244665 | 2.364016 | 8.173383 | 7.540567 | 6.894798 | 1.27E-41 | 1.658056 | up |
| GJA1 | 0.488641 | 0.718631 | 0.503791 | 0.028665 | 0.091855 | 0.019859 | 7.86E-12 | -3.49931 | down |
| MSTRG.15179 | 0.814459 | 0.942888 | 1.052116 | 1.7174 | 1.565242 | 1.477381 | 1.42E-10 | 1.033882 | up |
| MSTRG.20690 | 1.204574 | 1.030512 | 1.10698 | 3.461479 | 3.701138 | 3.850659 | 2.66E-66 | 1.859623 | up |
| RTKN2 | 0.7896 | 0.953074 | 0.858332 | 0.273858 | 0.261051 | 0.360759 | 4.04E-15 | -1.64741 | down |
| SOWAHA | 0.666068 | 0.716777 | 0.649638 | 0.146673 | 0.113638 | 0.033848 | 8.72E-13 | -2.75286 | down |
| RAC3 | 3.186138 | 3.526548 | 2.682578 | 1.275421 | 1.0706 | 0.892639 | 3.92E-08 | -1.48454 | down |
| B3GAT2 | 0.246505 | 0.157897 | 0.176366 | 0.416879 | 1.001596 | 0.677258 | 1.01E-09 | 1.812889 | up |
| AC104389.6 | 15.36496 | 13.78447 | 13.89599 | 539.0593 | 478.1829 | 499.8416 | 0 | 5.11349 | up |
| MRC2 | 0.069069 | 0.056273 | 0.08093 | 0.35916 | 0.369739 | 0.351234 | 9.96E-11 | 2.464721 | up |
| MSTRG.10546 | 1.693466 | 1.480149 | 1.38489 | 0.52199 | 0.186506 | 0.421505 | 1.09E-09 | -2.14692 | down |
| TTYH3 | 0.3379 | 0.442838 | 0.27679 | 0.086144 | 0.076092 | 0.059293 | 7.68E-08 | -2.18648 | down |
| LRRC20 | 2.029046 | 2.507269 | 1.967895 | 0.749258 | 0.663085 | 0.858111 | 5.77E-16 | -1.48571 | down |
| LPCAT1 | 0.397352 | 0.226181 | 0.396319 | 0.021156 | 0.103483 | 0.099998 | 4.28E-07 | -2.23821 | down |
| RHPN1 | 1.583202 | 1.440133 | 1.668221 | 0.548554 | 0.650648 | 0.59999 | 1.26E-13 | -1.39916 | down |
| PDK2 | 3.254636 | 3.047948 | 2.951027 | 0.641876 | 0.783776 | 0.711624 | 2.51E-41 | -2.11518 | down |
| LNPEP | 1.459517 | 1.645763 | 1.591419 | 2.958592 | 3.242574 | 3.522012 | 3.73E-37 | 1.024279 | up |
| ALPL | 1.283506 | 1.485736 | 1.513486 | 0.518141 | 0.419384 | 0.539142 | 2.04E-10 | -1.54616 | down |
| ARHGAP26 | 0.721752 | 0.617194 | 0.485907 | 0.216926 | 0.082168 | 0.077246 | 7.53E-15 | -2.07974 | down |
| CELSR3 | 0.910612 | 1.02689 | 0.916296 | 0.380307 | 0.39421 | 0.343247 | 6.14E-24 | -1.37236 | down |
| SWT1 | 2.872135 | 3.030184 | 2.695405 | 1.22185 | 1.445278 | 1.593695 | 3.69E-14 | -1.0318 | down |
| RSAD2 | 13.77274 | 15.08396 | 14.62015 | 6.377753 | 6.679018 | 8.331452 | 6.76E-41 | -1.08644 | down |
| ATP8B3 | 7.499049 | 3.109489 | 8.777111 | 2.094706 | 1.884907 | 2.695574 | 0.000124 | -1.00677 | down |
| C3orf52 | 0.51317 | 0.511555 | 0.458308 | 1.308027 | 1.31413 | 1.192175 | 3.55E-07 | 1.382066 | up |
| CAPG | 25.98663 | 27.19304 | 24.55906 | 11.51593 | 9.046355 | 9.720917 | 6.36E-48 | -1.3806 | down |
| STC2 | 0.099073 | 0.068908 | 0.103359 | 0.652786 | 0.600986 | 0.467376 | 3.79E-15 | 2.550544 | up |
| PERP | 0.407084 | 0.404131 | 0.467322 | 3.333605 | 3.232721 | 3.290453 | 1.59E-78 | 2.913315 | up |
| ZFP36L1 | 13.53867 | 14.04445 | 13.30857 | 28.70964 | 28.45557 | 27.56479 | 1.01E-74 | 1.034796 | up |
| PFKFB2 | 0.688701 | 0.892758 | 0.862956 | 0.097072 | 0.028066 | 0.069307 | 2.59E-34 | -3.65742 | down |
| SH3KBP1 | 4.722835 | 4.875275 | 4.298257 | 1.399882 | 1.332205 | 1.649484 | 1.46E-54 | -1.68862 | down |
| MSTRG.12025 | 2.531771 | 3.003236 | 3.540895 | 1.574771 | 1.628514 | 1.519968 | 2.67E-08 | -1.01507 | down |
| MSTRG.12026 | 1.048841 | 1.033128 | 1.245575 | 0.422463 | 0.643983 | 0.38859 | 1.25E-07 | -1.18169 | down |
| CRYZ | 27.22183 | 29.75444 | 28.58867 | 12.58662 | 11.06843 | 14.35998 | 3.41E-65 | -1.26032 | down |
| MFGE8 | 5.82588 | 5.935581 | 5.406968 | 1.528368 | 1.649568 | 1.353806 | 1.86E-35 | -1.92363 | down |
| DAZ3 | 2.886291 | 2.597535 | 2.632635 | 0.018198 | 0 | 0 | 1.94E-21 | -8.31647 | down |
| DAZ2 | 2.206155 | 1.921205 | 1.947417 | 0.004464 | 0.013565 | 0 | 5.82E-27 | -7.34941 | down |
| DAZ1 | 2.787787 | 3.064256 | 2.768483 | 0.00374 | 0.005029 | 0.0209 | 3.86E-36 | -7.73751 | down |
| DAZ4 | 3.42263 | 2.663435 | 3.538383 | 0.007249 | 0.01406 | 0 | 1.17E-22 | -8.55262 | down |
| NOVA1 | 0.3773 | 0.172192 | 0.500063 | 0 | 0 | 0 | 1.50E-08 | -7.51036 | down |
| MSTRG.20040 | 1.238424 | 1.460955 | 1.682175 | 0 | 0 | 0 | 3.76E-12 | -8.81448 | down |
| TRIM46 | 0.788633 | 1.004777 | 0.652346 | 0.061829 | 0.151258 | 0.185421 | 1.13E-10 | -2.40306 | down |
| IL2RB | 0.571797 | 0.745577 | 0.549993 | 1.420518 | 1.443743 | 1.270289 | 6.64E-09 | 1.123664 | up |
| IL2RG | 1.140524 | 1.341713 | 1.370059 | 3.305353 | 3.004453 | 3.424564 | 3.00E-10 | 1.299169 | up |
| MRAP2 | 11.96023 | 13.0712 | 12.79461 | 5.106037 | 5.410879 | 5.095891 | 7.59E-49 | -1.29959 | down |

| | | | | | | | | | |
|---|---|---|---|---|---|---|---|---|---|
| C16orf54 | 0.592079 | 0.687413 | 0.663284 | 0.077708 | 0 | 0.090717 | 1.97E-11 | -3.51781 | down |
| ZNF205 | 0.750457 | 0.606443 | 1.10877 | 0 | 0.068265 | 0.023531 | 1.29E-11 | -4.38486 | down |
| FMO5 | 0.23156 | 0.382856 | 0.188116 | 0.627193 | 0.701946 | 0.678012 | 0.002902 | 1.159793 | up |
| CDK15 | 11.26838 | 11.98406 | 11.29289 | 2.046424 | 1.932365 | 1.994422 | ######## | -2.52969 | down |
| MSTRG.17064 | 0.067753 | 0.089918 | 0.080607 | 0.631512 | 0.673941 | 0.889506 | 1.25E-26 | 3.133982 | up |
| MSTRG.17065 | 0.635078 | 1.183124 | 1.007043 | 2.733778 | 3.061059 | 3.304563 | 1.69E-25 | 1.696868 | up |
| PLXNA2 | 0.068196 | 0.087149 | 0.06125 | 0.472745 | 0.490156 | 0.455305 | 4.41E-29 | 2.663306 | up |
| CCDC152 | 0.150979 | 0.159136 | 0.076073 | 2.043966 | 1.864046 | 1.863516 | 1.64E-45 | 4.103615 | up |
| FN1 | 0.1723 | 0.179387 | 0.213317 | 0.036306 | 0.054042 | 0.087236 | 1.49E-05 | -1.67024 | down |
| AL133352.1 | 5.952135 | 5.420728 | 7.744979 | 3.370303 | 2.096753 | 1.750497 | 1.23E-14 | -1.54257 | down |
| MSTRG.1134 | 0.966487 | 0.867158 | 0.783804 | 0.065385 | 0.012581 | 0.072629 | 5.96E-08 | -3.88808 | down |
| KRT79 | 0 | 0 | 0 | 0.581423 | 0.649169 | 0.71737 | 2.85E-09 | 7.792455 | up |
| ITGBL1 | 2.349986 | 2.520886 | 2.246978 | 0.809473 | 1.012754 | 0.989619 | 2.14E-21 | -1.34553 | down |
| TTC28 | 0.526646 | 0.546213 | 0.503964 | 0.134002 | 0.151718 | 0.164548 | 1.19E-21 | -1.82621 | down |
| SLC22A16 | 1.30397 | 1.222169 | 1.526855 | 4.755955 | 4.345822 | 4.874796 | 6.36E-28 | 1.752764 | up |
| SYNJ2BP | 0.089349 | 0.12482 | 0.126982 | 0.28069 | 0.330341 | 0.215689 | 0.000247 | 1.238545 | up |
| ZCWPW1 | 3.0672 | 3.946186 | 3.807491 | 1.53544 | 1.987345 | 1.496845 | 9.51E-12 | -1.1566 | down |
| ZNF431 | 0.406245 | 0.368885 | 0.385413 | 0.899026 | 0.896968 | 0.964412 | 1.32E-23 | 1.238483 | up |
| MAP1A | 1.889061 | 1.866595 | 2.043146 | 0.914502 | 1.002458 | 0.997958 | 2.15E-25 | -1.0172 | down |
| MSTRG.3000 | 1.364736 | 1.488949 | 1.969398 | 0 | 0 | 0 | 1.21E-08 | -7.55227 | down |
| GNAL | 0.115168 | 0.096528 | 0.108953 | 0.343464 | 0.150439 | 0.299628 | 0.001496 | 1.257613 | up |
| DPF1 | 1.850141 | 2.06025 | 1.618949 | 0.649457 | 1.191838 | 0.689 | 2.81E-05 | -1.01012 | down |
| SPTBN4 | 1.117759 | 1.31912 | 1.863429 | 0.565827 | 0.51436 | 0.547437 | 6.33E-14 | -1.13529 | down |
| KLF15 | 5.094367 | 5.077761 | 5.008171 | 2.532537 | 2.461182 | 2.488619 | 1.27E-18 | -1.04361 | down |
| TFAP2A | 0.519462 | 0.461146 | 0.470976 | 0.089288 | 0.202846 | 0.124135 | 8.23E-06 | -1.6962 | down |
| SHOX2 | 1.527038 | 1.840459 | 1.494765 | 0.163006 | 0.313188 | 0.33673 | 1.93E-16 | -2.40774 | down |
| HBD | 227.9758 | 226.659 | 225.1868 | 34.53192 | 35.47565 | 33.14612 | 0 | -2.59342 | down |
| HBB | 14729.88 | 14895.21 | 15188.66 | 34.46737 | 32.65386 | 27.01872 | 0 | -8.88856 | down |
| DENND2B | 0.546335 | 0.495803 | 0.418323 | 0.047593 | 0.141592 | 0.104014 | 6.36E-12 | -2.82523 | down |
| SRGN | 6.302442 | 7.41306 | 7.392427 | 15.33693 | 14.01218 | 14.20689 | 3.50E-21 | 1.021403 | up |
| LRRC61 | 11.77145 | 11.78127 | 10.9433 | 6.239715 | 6.074569 | 5.179664 | 1.12E-22 | -1.00731 | down |
| DPEP2 | 3.129591 | 3.289822 | 2.821137 | 1.511948 | 1.305846 | 1.506663 | 9.30E-11 | -1.21267 | down |
| POU4F1 | 0.770452 | 0.809417 | 0.73778 | 2.003377 | 2.208248 | 2.193756 | 7.39E-23 | 1.439311 | up |
| CPNE7 | 1.50367 | 1.770446 | 1.468856 | 0.347133 | 0.646226 | 0.491213 | 1.39E-08 | -1.53692 | down |
| PYCARD | 8.853884 | 7.635194 | 6.607715 | 2.960185 | 4.192094 | 3.179388 | 1.84E-08 | -1.15818 | down |
| DNMT3A | 0.94459 | 0.465224 | 0.920198 | 2.545971 | 6.198183 | 3.150299 | 4.55E-79 | 2.314925 | up |
| MAST1 | 0.519473 | 0.771162 | 0.585644 | 0.135984 | 0.095157 | 0.098446 | 3.16E-06 | -1.67811 | down |
| NAPG | 2.671268 | 2.92778 | 2.910534 | 6.942169 | 6.976786 | 7.542755 | 1.54E-45 | 1.322814 | up |
| C3orf18 | 0.523363 | 0.446065 | 0.61914 | 0.033149 | 0.072763 | 0.008135 | 8.53E-07 | -3.03672 | down |
| C3orf14 | 2.702375 | 3.033862 | 1.361138 | 5.1049 | 4.306428 | 4.956249 | 1.29E-26 | 1.743392 | up |
| COL15A1 | 6.397782 | 6.524981 | 6.568827 | 3.097845 | 2.861393 | 2.907053 | 1.14E-49 | -1.16136 | down |
| SULT4A1 | 0.495225 | 0.613166 | 0.47009 | 0 | 0 | 0 | 8.96E-10 | -7.97395 | down |
| CABP7 | 0.149749 | 0.142406 | 0.093859 | 0.912551 | 0.806573 | 0.799577 | 1.63E-15 | 2.648603 | up |
| TMEM74B | 0.723676 | 0.844477 | 0.771316 | 0.179787 | 0.326472 | 0.17329 | 0.00022 | -1.62989 | down |
| HBE1 | 0.856252 | 1.424093 | 0.921777 | 18.37813 | 10.87377 | 3.970707 | 8.07E-07 | 3.524615 | up |
| DTX3 | 0.740129 | 0.487278 | 0.63433 | 0.130779 | 0.146981 | 0.032728 | 2.40E-06 | -2.34366 | down |
| S100A9 | 0 | 0 | 0 | 1.773489 | 1.57786 | 1.660809 | 1.94E-08 | 7.463587 | up |
| KIAA0513 | 0.263358 | 0.21904 | 0.256187 | 0.18102 | 0.058684 | 0.060369 | 0.001919 | -1.29528 | down |
| MORC4 | 0.438672 | 0.50303 | 0.462348 | 0 | 0.021467 | 0.052421 | 2.77E-12 | -4.11204 | down |
| DYSF | 3.311751 | 3.284427 | 2.867769 | 1.618305 | 1.627173 | 1.50298 | 9.32E-24 | -1.01801 | down |
| PPP1R13L | 2.618013 | 2.510321 | 2.310081 | 0.961552 | 0.988779 | 0.775771 | 7.26E-18 | -1.43871 | down |
| MCF2 | 9.36087 | 9.455548 | 9.796332 | 3.732751 | 3.446107 | 4.272881 | 4.41E-52 | -1.33204 | down |
| TUSC3 | 0.77425 | 0.609446 | 0.57762 | 0 | 0 | 0 | 8.88E-12 | -8.68139 | down |
| DNAJC15 | 2.016927 | 1.837511 | 1.993826 | 0.630691 | 0.580076 | 0.575424 | 4.77E-43 | -1.73054 | down |
| NEK3 | 2.047272 | 1.739258 | 2.209065 | 5.092154 | 5.481318 | 5.339951 | 4.54E-23 | 1.317705 | up |

| | | | | | | | | | |
|---|---|---|---|---|---|---|---|---|---|
| TGFB3 | 1.827691 | 1.819243 | 1.811846 | 0.676897 | 0.806167 | 0.917525 | 1.00E-11 | -1.21044 | down |
| TBKBP1 | 0.65688 | 0.53843 | 0.449884 | 0.062042 | 0.07847 | 0.036003 | 1.53E-14 | -3.21914 | down |
| FEZ1 | 7.48618 | 6.752108 | 6.103327 | 2.219669 | 2.578942 | 2.157275 | 9.48E-52 | -1.84887 | down |
| CD52 | 30.39817 | 36.24104 | 35.24912 | 17.09035 | 13.19575 | 12.234 | 2.69E-22 | -1.2804 | down |
| EPX | 1.636527 | 1.602063 | 1.195817 | 0.524023 | 0.411826 | 0.338664 | 1.46E-12 | -1.80681 | down |
| MSTRG.12488 | 0.438024 | 0.478665 | 0.586786 | 1.027209 | 1.163113 | 1.396694 | 9.91E-07 | 1.194621 | up |
| RHOB | 20.91703 | 21.56982 | 20.76617 | 8.219181 | 7.711373 | 7.493425 | 6.36E-94 | -1.45647 | down |
| ELAC1 | 1.078495 | 1.231982 | 1.270574 | 3.060861 | 2.794633 | 2.229068 | 6.65E-09 | 1.163984 | up |
| FLT3 | 0.07083 | 0.073153 | 0.1067 | 0.256398 | 0.238881 | 0.33365 | 0.000502 | 1.646322 | up |
| KCNA7 | 0.24446 | 0.317494 | 0.199926 | 0.074649 | 0.027171 | 0.045109 | 9.34E-06 | -2.26839 | down |
| NOL4L | 1.062919 | 0.669283 | 0.827408 | 1.907945 | 1.950451 | 2.028893 | 5.44E-19 | 1.247387 | up |
| SLC35D3 | 1.427737 | 1.248753 | 1.428935 | 0.593164 | 0.295608 | 0.628279 | 2.62E-07 | -1.43851 | down |
| RAB38 | 0.22152 | 0.375294 | 0.362879 | 0.998421 | 0.951654 | 1.001465 | 9.27E-05 | 1.568054 | up |
| C11orf96 | 1.299302 | 1.262454 | 1.11695 | 0.325507 | 0.137155 | 0.024319 | 9.06E-09 | -2.91315 | down |
| SYNJ2 | 1.180499 | 1.239946 | 0.985127 | 0.833694 | 0.732531 | 0.543024 | 8.64E-11 | -1.15877 | down |
| WDR31 | 0.776204 | 1.105218 | 1.499621 | 0.44218 | 0.531276 | 0.578825 | 1.27E-06 | -1.04472 | down |
| SMIM20 | 24.93251 | 26.36411 | 25.88153 | 12.32126 | 11.83631 | 11.65848 | 2.56E-38 | -1.14491 | down |
| NKX2-4 | 7.76667 | 7.457883 | 7.977285 | 3.387592 | 3.327105 | 3.777432 | 1.71E-22 | -1.16845 | down |
| TDRD7 | 2.48846 | 2.453935 | 2.263912 | 0.750156 | 0.599286 | 0.884069 | 4.63E-25 | -1.71378 | down |
| SERPINB1 | 4.931203 | 4.557935 | 5.025734 | 0.114152 | 0.106897 | 0.120752 | 2.79E-72 | -5.40035 | down |
| MAGED1 | 11.21381 | 11.23775 | 10.81782 | 0.054358 | 0.070942 | 0.045005 | 4.55E-97 | -7.41361 | down |
| RNASET2 | 1.521221 | 1.162455 | 1.272758 | 0.669736 | 0.619515 | 0.539266 | 1.76E-09 | -1.01615 | down |
| ASXL3 | 0.085239 | 0.113814 | 0.118865 | 0 | 0 | 0 | 1.80E-09 | -7.8646 | down |
| SMC1B | 6.172498 | 5.581572 | 6.582734 | 2.522074 | 2.600993 | 3.271785 | 1.36E-27 | -1.09718 | down |
| TRIM47 | 1.168047 | 1.398685 | 1.442367 | 0.668442 | 0.586126 | 0.677618 | 3.73E-05 | -1.06896 | down |
| GAS6 | 2.074977 | 2.234964 | 1.577467 | 0.353783 | 0.500496 | 0.71763 | 8.34E-15 | -1.92124 | down |
| RAB12 | 1.321626 | 1.614994 | 1.251819 | 3.724473 | 3.845027 | 3.765962 | 3.13E-18 | 1.405038 | up |
| ME3 | 1.688042 | 1.623351 | 1.724032 | 0.731964 | 0.374307 | 0.597626 | 9.98E-11 | -1.62502 | down |
| GGT1 | 2.35159 | 1.872637 | 2.31788 | 5.441742 | 4.449668 | 5.067474 | 3.76E-12 | 1.100561 | up |
| PTPRS | 0.240653 | 0.239002 | 0.322275 | 0.12691 | 0.030967 | 0.025185 | 3.29E-08 | -2.4439 | down |
| CLGN | 0.410168 | 0.620651 | 0.581705 | 0.177422 | 0.086385 | 0.231078 | 4.96E-05 | -1.67782 | down |
| CDR2L | 0.406658 | 0.30192 | 0.407642 | 0.125521 | 0.038484 | 0.109779 | 1.39E-05 | -1.999 | down |
| IFIT5 | 0.462349 | 0.617711 | 0.479656 | 0.049487 | 0.125862 | 0.110273 | 2.37E-10 | -2.42662 | down |
| IFIT1 | 0.531264 | 0.578101 | 0.584983 | 0.213067 | 0.328054 | 0.260632 | 0.000141 | -1.08595 | down |
| CEBPD | 1.677968 | 1.773356 | 1.493295 | 0.630721 | 0.718675 | 0.304659 | 5.68E-06 | -1.57457 | down |
| ABHD17C | 1.373672 | 1.577182 | 1.241194 | 0.392817 | 0.451584 | 0.57344 | 2.68E-09 | -1.55847 | down |
| GCH1 | 1.060346 | 1.121398 | 0.948891 | 5.00197 | 5.05734 | 6.281703 | 9.62E-59 | 2.333415 | up |
| SIPA1L3 | 1.599317 | 1.768754 | 1.67206 | 0.804785 | 0.866753 | 0.857114 | 2.22E-18 | -1.01902 | down |
| PLA2G4A | 2.360902 | 2.006899 | 1.976087 | 0.792224 | 1.19811 | 0.893815 | 2.80E-09 | -1.15488 | down |
| CMTM7 | 3.934199 | 4.18843 | 3.830781 | 2.184564 | 1.659396 | 1.734016 | 2.97E-12 | -1.23748 | down |
| TSPAN12 | 0.167185 | 0.240957 | 0.194423 | 0.710018 | 1.104122 | 0.835757 | 7.15E-09 | 1.992224 | up |
| VIM | 257.0962 | 257.8437 | 244.4591 | 85.14834 | 83.49125 | 81.39622 | 0 | -1.62822 | down |
| TBC1D8B | 0.331159 | 0.231801 | 0.393752 | 0.03412 | 0 | 0.034412 | 1.82E-10 | -3.78774 | down |
| CLDN3 | 5.337831 | 4.780979 | 3.923502 | 1.419957 | 1.839013 | 1.274325 | 3.78E-15 | -1.64136 | down |
| H2BC11 | 3.902354 | 4.046675 | 4.569597 | 1.650924 | 1.698198 | 2.12825 | 2.14E-07 | -1.26854 | down |
| SPATA32 | 0.645693 | 0.633313 | 0.728754 | 2.699717 | 2.450895 | 3.042805 | 9.09E-11 | 1.580492 | up |
| NAGPA | 6.70903 | 7.41108 | 6.232385 | 3.601857 | 3.020881 | 3.249457 | 3.89E-19 | -1.06961 | down |
| EGR1 | 56.62703 | 56.78919 | 55.55113 | 13.13804 | 13.41296 | 13.55503 | 0 | -2.0988 | down |
| RAPGEF5 | 0.690118 | 0.816966 | 0.723109 | 1.639668 | 1.722965 | 1.889245 | 8.27E-20 | 1.188244 | up |
| NKPD1 | 5.231554 | 3.317936 | 2.991547 | 1.066591 | 1.267312 | 0.904144 | 0.000228 | -2.28243 | down |
| MSTRG.12871 | 0.875063 | 0.589591 | 0.803097 | 0.408575 | 0.295686 | 0.377562 | 0.001075 | -1.00981 | down |
| WWTR1 | 1.018238 | 1.314893 | 1.125708 | 0.211813 | 0.126181 | 0.134516 | 6.33E-32 | -3.09862 | down |
| TNFRSF14 | 4.741622 | 4.350852 | 4.740638 | 1.847547 | 2.472274 | 1.919354 | 4.03E-12 | -1.18995 | down |
| MSTRG.18994 | 0.974966 | 0.385138 | 1.019737 | 3.489994 | 3.124014 | 3.25675 | 0.000807 | 2.078953 | up |
| CR2 | 0.945304 | 0.908919 | 0.955225 | 0.038566 | 0.033591 | 0.044179 | 4.18E-27 | -4.52509 | down |
| TNFRSF1A | 5.520028 | 4.228762 | 4.6214 | 10.0752 | 9.500308 | 10.2751 | 2.97E-20 | 1.021646 | up |

| | | | | | | | | | |
|---|---|---|---|---|---|---|---|---|---|
| FAM174B | 1.242658 | 0.903252 | 1.286428 | 0.311301 | 0.348407 | 0.121205 | 1.30E-15 | -3.08623 | down |
| CAPS2 | 0.081283 | 0.046147 | 0.088048 | 0.252717 | 0.273327 | 0.34363 | 0.000332 | 1.791026 | up |
| PTPN6 | 16.76002 | 17.55135 | 16.70982 | 7.854298 | 7.726559 | 7.710281 | 1.44E-52 | -1.14184 | down |
| EPB41L4B | 1.295734 | 1.478287 | 1.366168 | 0.538305 | 0.486332 | 0.59083 | 6.78E-13 | -1.37011 | down |
| CD68 | 3.422856 | 3.338193 | 3.829027 | 7.248167 | 7.53493 | 7.550276 | 1.26E-17 | 1.046394 | up |
| USP9Y | 0.032316 | 0.023923 | 0.010766 | 0.19952 | 0.209495 | 0.252459 | 1.40E-13 | 3.154035 | up |
| ZFP42 | 1.852845 | 2.146178 | 2.131307 | 0.015238 | 0.023585 | 0.030813 | 8.08E-26 | -6.23951 | down |
| RHOBTB1 | 0.469685 | 0.442824 | 0.748019 | 0.07782 | 0.067415 | 0.127466 | 6.34E-12 | -2.57507 | down |
| KCNE3 | 0.824509 | 0.813444 | 0.624371 | 2.188038 | 2.597023 | 2.885413 | 3.65E-21 | 1.699343 | up |
| RFTN1 | 0.782602 | 0.568256 | 0.741288 | 2.7116 | 2.242151 | 2.61358 | 6.49E-30 | 2.306317 | up |
| KCTD17 | 3.043219 | 2.109819 | 2.226777 | 0.9964 | 1.148192 | 1.227453 | 1.64E-06 | -1.08528 | down |
| SLC25A35 | 2.714972 | 3.048866 | 2.790264 | 1.229534 | 1.75467 | 1.267484 | 2.92E-08 | -1.00205 | down |
| ENDOD1 | 2.241044 | 2.716004 | 2.997229 | 5.966248 | 6.18813 | 6.443805 | 5.05E-36 | 1.20174 | up |
| TSPAN33 | 0.323688 | 0.394582 | 0.561933 | 0.219903 | 0.182851 | 0.128011 | 0.002894 | -1.26443 | down |
| FOSB | 8.442645 | 9.060479 | 9.229722 | 19.70296 | 20.69525 | 18.31727 | 1.60E-52 | 1.038086 | up |
| HEPH | 1.060664 | 1.086197 | 0.945531 | 0.150671 | 0.231973 | 0.374447 | 9.00E-18 | -2.00061 | down |
| RIC3 | 1.182058 | 1.113455 | 0.545538 | 0.012072 | 0.010207 | 0.103473 | 5.08E-17 | -4.31862 | down |
| SNN | 0.807985 | 0.923403 | 0.710121 | 0.218592 | 0.335625 | 0.216908 | 3.06E-08 | -1.65344 | down |
| ANXA1 | 20.71931 | 21.03177 | 22.39525 | 5.092677 | 3.638014 | 4.653278 | ######## | -2.27244 | down |
| MAK | 3.301791 | 3.696534 | 3.63161 | 1.231591 | 1.908693 | 1.946276 | 2.04E-22 | -1.2468 | down |
| MAL | 1.098747 | 0.800257 | 0.896318 | 0 | 0.053411 | 0 | 1.24E-06 | -5.71181 | down |
| COL1A2 | 0.204646 | 0.239005 | 0.46855 | 0 | 0 | 0 | 4.02E-10 | -8.17741 | down |
| PRSS57 | 2.765443 | 3.344665 | 2.274367 | 8.282433 | 7.439365 | 5.962049 | 1.27E-12 | 1.333691 | up |
| CYTL1 | 29.77223 | 30.62925 | 32.27719 | 0.863723 | 0.463357 | 0.565203 | ######## | -5.60758 | down |
| VAPA | 11.98467 | 10.77336 | 9.790953 | 26.24197 | 27.21447 | 29.4205 | ######## | 1.297853 | up |
| ARG2 | 4.588711 | 4.877802 | 4.478928 | 1.817036 | 2.38408 | 1.870512 | 3.19E-15 | -1.22018 | down |
| RDM1 | 1.061522 | 1.603662 | 0.980543 | 0.502325 | 0.618288 | 0.460718 | 0.006603 | -1.04694 | down |
| TSPYL5 | 0.19912 | 0.301629 | 0.273232 | 0 | 0 | 0.039153 | 0.004048 | -4.28343 | down |
| MSTRG.8802 | 0.44608 | 0.606919 | 0.613631 | 1.649694 | 1.257812 | 1.645374 | 4.67E-08 | 1.425357 | up |
| SFMBT2 | 0.750575 | 0.66284 | 0.656833 | 0.109991 | 0.046509 | 0.113487 | 1.24E-28 | -2.88768 | down |
| KRT19 | 11.91099 | 10.7343 | 11.61349 | 2.965053 | 2.62827 | 2.215412 | 9.09E-58 | -2.15184 | down |
| ZBTB16 | 0.24212 | 0.234346 | 0.24937 | 0.847112 | 0.710673 | 0.688638 | 6.54E-14 | 1.431613 | up |
| ANKRD13B | 3.041817 | 2.617616 | 2.866355 | 1.483882 | 1.327624 | 1.155293 | 3.56E-12 | -1.0275 | down |
| MCTP2 | 0.965114 | 0.942092 | 1.039727 | 3.085196 | 2.95999 | 3.655987 | 4.51E-53 | 1.562198 | up |
| PLPP2 | 0.99187 | 1.090845 | 1.077722 | 0.313319 | 0.391028 | 0.226603 | 3.38E-05 | -1.71455 | down |
| CKB | 38.00518 | 40.54414 | 38.22243 | 13.705 | 13.48523 | 12.92822 | ######## | -1.57562 | down |
| SLFN12 | 0.518615 | 0.54507 | 0.532385 | 0.221477 | 0.221597 | 0.149104 | 0.000794 | -1.34743 | down |
| MST1 | 0.613618 | 0.759842 | 0.805435 | 0.258573 | 0.296076 | 0.253475 | 7.01E-05 | -1.35367 | down |
| CDH24 | 4.202298 | 4.076929 | 3.982225 | 1.935037 | 1.564305 | 1.316893 | 2.25E-24 | -1.35853 | down |
| AKR1B1 | 30.05014 | 31.41299 | 32.22424 | 1.902258 | 1.974606 | 2.364429 | ######## | -3.91315 | down |
| ARHGAP5 | 2.462902 | 2.559717 | 3.27721 | 0.296944 | 0.297811 | 0.269806 | 1.15E-98 | -2.90667 | down |
| ZNF615 | 0.379648 | 1.021771 | 0.588852 | 0.026187 | 0 | 0 | 1.77E-12 | -5.82556 | down |
| ARHGAP25 | 0.583149 | 0.587564 | 0.737333 | 0.100766 | 0.039068 | 0.069907 | 3.27E-11 | -3.02527 | down |
| HPGDS | 1.566913 | 1.93282 | 1.635516 | 0.072761 | 0.047999 | 0 | 2.09E-17 | -5.3198 | down |
| RAP1GAP2 | 0.038804 | 0.068832 | 0.034195 | 0.258566 | 0.153928 | 0.245587 | 1.63E-06 | 2.158608 | up |
| MYL12A | 54.87211 | 59.70246 | 61.33622 | 120.2827 | 120.0136 | 119.7176 | 1.01E-89 | 1.017604 | up |
| SEL1L3 | 3.168938 | 3.058243 | 2.775455 | 6.774423 | 7.191082 | 7.451851 | 1.11E-44 | 1.210413 | up |
| IL1RAP | 0.356472 | 0.457084 | 0.313633 | 0.061304 | 0.090269 | 0.035074 | 4.83E-08 | -2.3237 | down |
| FAT1 | 0.626177 | 0.639464 | 0.815397 | 0.016491 | 0.009689 | 0.009641 | 2.33E-55 | -5.75207 | down |
| DSG2 | 5.554934 | 5.345474 | 5.317841 | 11.55168 | 10.89892 | 12.6693 | 1.12E-60 | 1.092806 | up |
| B3GALNT1 | 1.776551 | 2.128727 | 1.911955 | 0.131824 | 0.115321 | 0.154394 | 1.34E-42 | -3.46999 | down |

| | | | | | | | | | |
|---|---|---|---|---|---|---|---|---|---|
| TMEM233 | 2.884423 | 2.739352 | 2.291529 | 0.760561 | 0.647498 | 0.892674 | 2.81E-22 | -1.80868 | down |
| LAP3 | 83.84583 | 89.01955 | 91.91661 | 42.919 | 41.4931 | 42.69646 | ####### # | -1.08781 | down |
| PTGER3 | 7.615333 | 7.734629 | 7.408654 | 2.328766 | 2.228888 | 2.932746 | 2.91E-61 | -1.51285 | down |
| RAB27A | 5.437626 | 5.253446 | 5.6035 | 10.86869 | 10.99521 | 11.30577 | 5.22E-41 | 1.024043 | up |
| TBC1D2B | 0.697943 | 0.460964 | 0.594547 | 0.192902 | 0.215953 | 0.318286 | 1.12E-06 | -1.2812 | down |
| ACP5 | 6.386271 | 6.376727 | 6.134404 | 0 | 0 | 0.138673 | 1.12E-25 | -7.93474 | down |
| ABCG2 | 0.383909 | 0.549199 | 0.267127 | 2.583209 | 2.525749 | 2.209992 | 3.28E-44 | 2.51898 | up |
| ZNF493 | 0.192331 | 0.274417 | 0.447732 | 0.923358 | 0.815638 | 0.807093 | 6.67E-10 | 1.710476 | up |
| CASKIN2 | 0.647876 | 0.468627 | 0.659748 | 0.146833 | 0.096245 | 0.194093 | 8.58E-12 | -2.10725 | down |
| MEX3A | 0.153826 | 0.148803 | 0.162516 | 0.052359 | 0.06214 | 0.057903 | 0.001367 | -1.45145 | down |
| CRYBG1 | 4.48752 | 4.680264 | 4.683656 | 10.90954 | 11.29831 | 12.2999 | ####### # | 1.29153 | up |
| CDK6 | 0.748695 | 0.87426 | 0.852566 | 6.204297 | 6.360597 | 7.264614 | ####### # | 2.974768 | up |
| CEP55 | 12.1835 | 12.25243 | 13.34405 | 6.392768 | 5.927825 | 6.037003 | 3.11E-40 | -1.06648 | down |
| ZNF799 | 0.767195 | 0.517723 | 0.867854 | 0.019746 | 0.045013 | 0.087115 | 1.11E-12 | -3.57966 | down |
| TRPV3 | 0.594503 | 0.497677 | 0.629713 | 0.302005 | 0.213279 | 0.246953 | 2.11E-06 | -1.18378 | down |
| FGFR4 | 0.82062 | 0.735633 | 0.54836 | 0.361419 | 0.275474 | 0.231467 | 0.000142 | -1.25394 | down |
| FGFR1 | 0.398247 | 0.288602 | 0.310739 | 1.19534 | 0.880825 | 1.238905 | 1.18E-14 | 1.653049 | up |
| MSTRG.16701 | 1.510946 | 1.334252 | 1.510207 | 0.732485 | 0.717829 | 0.681362 | 7.39E-08 | -1.01789 | down |
| DSEL | 0.389773 | 0.42647 | 0.3065 | 0.06963 | 0.056555 | 0.056472 | 2.89E-18 | -2.61432 | down |
| PDZK1IP1 | 7.849038 | 7.598465 | 7.81111 | 3.97526 | 3.853565 | 3.869193 | 8.51E-10 | -1.01565 | down |
| PSMB8 | 11.24426 | 13.57868 | 12.88678 | 6.594262 | 6.424291 | 6.017381 | 6.37E-18 | -1.0166 | down |
| SPINDOC | 6.896795 | 7.419677 | 6.411756 | 3.659801 | 3.779664 | 3.080529 | 9.19E-18 | -1.08558 | down |
| BIRC3 | 0.091278 | 0.139452 | 0.133687 | 0.491562 | 0.42943 | 0.751775 | 3.53E-13 | 2.014158 | up |
| ANKRD12 | 2.607215 | 2.907668 | 1.990481 | 5.009284 | 5.210824 | 5.778513 | 6.89E-47 | 1.088931 | up |
| NUTM2A | 1.76174 | 1.191919 | 1.407519 | 0.663505 | 0.771593 | 0.731829 | 1.12E-06 | -1.02262 | down |
| NHLRC2 | 1.364146 | 1.52918 | 1.352972 | 2.987083 | 2.823051 | 3.303987 | 1.04E-36 | 1.077106 | up |
| LYZ | 0.46596 | 0.582754 | 0.871717 | 1.560372 | 1.379625 | 1.424353 | 0.00024 | 1.159042 | up |
| SLC7A8 | 14.34972 | 14.95927 | 13.36665 | 3.648404 | 4.652657 | 3.207067 | ####### # | -2.15555 | down |
| PLEKHA2 | 0.107903 | 0.150346 | 0.142848 | 0.627261 | 0.731639 | 0.727247 | 3.94E-18 | 2.321662 | up |
| TNFAIP3 | 0.695206 | 0.592761 | 1.378687 | 2.53788 | 2.293568 | 1.776826 | 4.81E-09 | 1.110806 | up |
| PDGFD | 0.267372 | 0.289616 | 0.305736 | 0.105098 | 0.133313 | 0.020147 | 0.000778 | -1.66965 | down |
| SMCHD1 | 4.841932 | 5.228071 | 5.569763 | 11.18187 | 11.65166 | 13.47235 | 6.57E-76 | 1.150131 | up |
| SCPEP1 | 3.664247 | 3.535183 | 3.816023 | 12.29985 | 13.37714 | 12.16453 | 4.44E-67 | 1.748696 | up |
| PAQR7 | 1.199779 | 1.231104 | 1.365982 | 0.456158 | 0.47617 | 0.734243 | 3.07E-06 | -1.14696 | down |
| FERMT1 | 0.699901 | 0.757778 | 0.924808 | 0.14122 | 0.149875 | 0.085119 | 3.05E-19 | -2.65262 | down |
| WDR97 | 0.323567 | 0.300728 | 0.210087 | 0.07993 | 0.116931 | 0.101483 | 1.60E-05 | -1.47834 | down |
| MSTRG.2653 | 3.181705 | 3.061546 | 2.919592 | 1.213178 | 1.448639 | 1.856546 | 8.31E-08 | -1.00338 | down |
| TMEM121 | 3.074098 | 3.157713 | 2.576917 | 0.788445 | 1.209597 | 1.062739 | 2.36E-11 | -1.523 | down |
| USP14 | 14.59116 | 15.44615 | 14.79247 | 29.48013 | 30.36155 | 31.68441 | ####### # | 1.006394 | up |
| CEP76 | 2.994903 | 2.940462 | 3.239084 | 6.472185 | 5.935351 | 6.422942 | 3.31E-22 | 1.011596 | up |
| ZNF519 | 0.520516 | 0.570349 | 0.548045 | 1.176048 | 1.144794 | 1.406788 | 3.55E-12 | 1.179177 | up |
| ZNF516 | 0.108683 | 0.152655 | 0.118142 | 0.434386 | 0.403644 | 0.441793 | 9.29E-11 | 1.647688 | up |
| KLRG2 | 6.306049 | 7.329172 | 6.243232 | 2.440433 | 2.313173 | 2.194139 | 2.37E-33 | -1.51979 | down |
| AFDN | 3.689071 | 3.308131 | 4.131203 | 0.378458 | 0.47733 | 0.546018 | 2.29E-60 | -2.50585 | down |
| ARMCX4 | 0.287083 | 0.364996 | 0.353736 | 0 | 0.003319 | 0.01024 | 2.55E-13 | -5.91394 | down |
| FUT9 | 2.416922 | 2.36908 | 2.703565 | 0.881583 | 0.85061 | 1.046327 | 1.54E-55 | -1.45385 | down |
| RNF207 | 2.526584 | 2.579612 | 2.954072 | 1.038873 | 1.147627 | 0.895982 | 4.66E-22 | -1.40512 | down |
| EPOP | 5.342731 | 5.543163 | 5.200663 | 2.561631 | 2.724496 | 2.789837 | 6.81E-23 | -1.01682 | down |
| MSTRG.16573 | 0.322188 | 0.263256 | 0.230758 | 0.808385 | 0.780148 | 0.747741 | 0.000442 | 1.484862 | up |
| SATB1 | 1.153029 | 1.126861 | 1.221606 | 0 | 0 | 0 | 4.73E-18 | -10.6739 | down |
| CAPRIN2 | 4.993067 | 5.03978 | 5.74305 | 11.54734 | 11.04927 | 12.08264 | 5.21E-52 | 1.070414 | up |

| | | | | | | | | | |
|---|---|---|---|---|---|---|---|---|---|
| LRRC6 | 0.727487 | 0.607234 | 0.655432 | 0.198415 | 0.282108 | 0.28535 | 0.000131 | -1.24709 | down |
| DOCK6 | 1.39321 | 1.598522 | 1.511141 | 0.498978 | 0.544351 | 0.520805 | 1.19E-23 | -1.50817 | down |
| SCNN1D | 0.658071 | 0.573966 | 0.602358 | 0.267115 | 0.345788 | 0.150684 | 0.000773 | -1.21492 | down |
| ARHGEF33 | 1.567848 | 1.231737 | 1.273694 | 0.586691 | 0.74066 | 0.511005 | 1.01E-07 | -1.13242 | down |
| FAM184B | 0.939531 | 0.957578 | 1.067809 | 0.359832 | 0.378577 | 0.425953 | 3.63E-15 | -1.36697 | down |
| MYEF2 | 1.304354 | 1.980988 | 2.128902 | 0.004738 | 0 | 0.005335 | 5.43E-23 | -7.53407 | down |
| RUSC2 | 0.67159 | 0.592672 | 0.722964 | 0.309665 | 0.257053 | 0.27073 | 3.36E-07 | -1.23164 | down |
| WASF3 | 0.857546 | 0.935042 | 0.734781 | 0.047737 | 0.178929 | 0.143327 | 1.09E-19 | -2.78853 | down |
| RAVER2 | 1.058619 | 0.847079 | 1.095798 | 0.031148 | 0.082168 | 0.045058 | 1.12E-29 | -4.15754 | down |
| MSTRG.8698 | 1.02832 | 1.24667 | 0.970787 | 0.380872 | 0.392976 | 0.310949 | 2.60E-22 | -1.783 | down |
| SP140L | 1.460809 | 1.468258 | 1.745285 | 3.65809 | 3.035501 | 3.036344 | 8.94E-11 | 1.033608 | up |
| PCBP4 | 1.109212 | 0.583149 | 1.030935 | 0.39087 | 0.35947 | 0.366053 | 0.000385 | -1.24138 | down |
| RASD1 | 1.389189 | 1.568641 | 1.265754 | 0.430021 | 0.484781 | 0.249034 | 7.32E-09 | -1.85153 | down |
| FGF14 | 0.428665 | 0.487942 | 0.498422 | 0.260427 | 0.191789 | 0.265984 | 3.77E-08 | -1.00299 | down |
| RNF157 | 0.407527 | 0.495462 | 0.413488 | 1.22865 | 1.513099 | 1.352559 | 1.96E-16 | 1.561785 | up |
| SYCP2L | 20.46055 | 21.54089 | 20.95659 | 7.940658 | 8.137337 | 9.046099 | 2.53E-97 | -1.34933 | down |
| ZNF254 | 0.844075 | 0.728428 | 0.647349 | 1.43342 | 1.361203 | 1.753039 | 2.56E-09 | 1.085893 | up |
| PGAP4 | 11.89478 | 11.7285 | 12.21343 | 4.728279 | 3.908991 | 5.003221 | 1.48E-71 | -1.41869 | down |
| CIDEB | 0.445732 | 0.429131 | 0.539757 | 0.211402 | 0.165532 | 0.165046 | 0.002875 | -1.38764 | down |
| AKTIP | 0.443421 | 0.721549 | 1.136503 | 0.406251 | 0.317816 | 0.369689 | 0.004511 | -1.06154 | down |
| RIMKLB | 0.358094 | 0.358252 | 0.520039 | 2.655794 | 3.329216 | 3.451498 | 1.26E-57 | 2.925824 | up |
| CRYL1 | 0.446183 | 0.549306 | 0.08367 | 1.492479 | 1.959548 | 1.659973 | 1.91E-07 | 1.964067 | up |
| ITGB4 | 2.643718 | 2.610042 | 2.536088 | 1.06652 | 0.976575 | 0.984577 | 1.29E-30 | -1.38331 | down |
| ZFPM2 | 0.415702 | 0.361137 | 0.278936 | 2.044996 | 1.940602 | 2.281921 | 2.54E-46 | 2.515116 | up |
| SLC12A8 | 1.661998 | 1.4087 | 1.567329 | 0.742442 | 0.620876 | 0.781937 | 5.58E-09 | -1.14692 | down |
| MSTRG.19106 | 1.604714 | 1.7681 | 1.863342 | 0.665312 | 0.905037 | 0.744653 | 6.83E-07 | -1.10245 | down |
| DOCK11 | 1.76254 | 1.489397 | 1.564878 | 3.893702 | 3.714638 | 4.215041 | 1.06E-37 | 1.306068 | up |
| AKR1C1 | 0.430177 | 0.501269 | 0.551915 | 0.135263 | 0.186133 | 0.114113 | 8.98E-11 | -1.78938 | down |
| LAMP3 | 0.58814 | 0.696675 | 0.817441 | 0.018537 | 0.051882 | 0.125413 | 1.33E-12 | -3.19625 | down |
| SLC9A5 | 1.470113 | 1.919844 | 1.706587 | 0.811437 | 0.868115 | 0.751989 | 1.02E-09 | -1.09465 | down |
| HMGA2 | 0.031041 | 0.042846 | 0.034493 | 0.583751 | 0.724382 | 0.600554 | 4.07E-18 | 3.821602 | up |
| MSTRG.11895 | 0.514229 | 0.470253 | 0.522464 | 0.152497 | 0.046965 | 0.182895 | 3.89E-05 | -1.89021 | down |
| SLC9A2 | 0.668519 | 0.665008 | 0.618735 | 2.105637 | 2.319618 | 2.361522 | 3.43E-39 | 1.769943 | up |
| TLX3 | 0.274004 | 0.267418 | 0.393574 | 1.275643 | 1.337442 | 0.867556 | 1.39E-06 | 1.846171 | up |
| SLC30A1 | 0.206234 | 0.142183 | 0.246003 | 0.724543 | 0.745086 | 0.73748 | 1.42E-14 | 1.851574 | up |
| CNN1 | 0.890884 | 0.596849 | 0.779446 | 0.17776 | 0.144951 | 0.131918 | 1.09E-05 | -2.22457 | down |
| CNN3 | 1.646718 | 1.771516 | 2.030756 | 0.621072 | 0.224132 | 0.48607 | 1.46E-12 | -2.04336 | down |
| PSMB9 | 2.671328 | 2.93223 | 3.173613 | 1.612481 | 1.275313 | 1.38163 | 3.62E-05 | -1.05193 | down |
| CCR7 | 0.457849 | 0.23929 | 0.349876 | 1.622791 | 1.817609 | 1.802552 | 1.01E-16 | 2.285129 | up |
| GALNT14 | 5.367072 | 4.763074 | 4.983407 | 2.281069 | 1.915833 | 1.968639 | 2.10E-22 | -1.3073 | down |
| LRP4 | 0.942571 | 0.892236 | 1.195953 | 0.445651 | 0.343151 | 0.330113 | 2.89E-16 | -1.45609 | down |
| PTPN21 | 0.49196 | 0.547273 | 0.476236 | 0.058348 | 0.051472 | 0.099012 | 5.35E-19 | -2.85033 | down |
| AFG3L2 | 17.63499 | 20.80954 | 20.6509 | 38.70282 | 39.35862 | 43.42877 | 5.00E-63 | 1.015533 | up |
| MSTRG.10531 | 1.806799 | 1.353671 | 1.516761 | 0.565989 | 0.30814 | 0.655849 | 2.26E-13 | -1.62927 | down |
| PTGES3L-AARSD1 | 4.811263 | 0.9045 | 1.01616 | 0.27089 | 0.291554 | 0.036805 | 0.000229 | -4.13796 | down |
| DENND5A | 2.983475 | 2.850676 | 3.144099 | 1.59211 | 1.218536 | 1.313926 | 1.20E-28 | -1.34922 | down |
| RAB3A | 0.97814 | 0.856835 | 0.859808 | 0.165512 | 0.466456 | 0.314234 | 0.000695 | -1.44983 | down |
| SORT1 | 4.552843 | 4.522188 | 4.213125 | 1.920388 | 1.91313 | 2.215377 | 4.68E-40 | -1.14843 | down |
| SIGLEC6 | 5.683784 | 5.969907 | 6.042212 | 3.346707 | 2.577389 | 2.61308 | 6.59E-28 | -1.10201 | down |
| PPP1R14A | 0.49842 | 1.156483 | 0.234374 | 4.261116 | 2.467581 | 3.568473 | 1.54E-07 | 2.118765 | up |
| KSR1 | 40.64444 | 40.71631 | 47.92133 | 18.76151 | 17.97427 | 21.08642 | ######## | -1.20609 | down |
| FHDC1 | 1.936668 | 1.768668 | 1.763339 | 4.333801 | 5.225951 | 5.519586 | 1.79E-48 | 1.43059 | up |
| NOL3 | 1.898755 | 1.706632 | 2.143408 | 0.612748 | 0.471732 | 0.604048 | 1.42E-10 | -1.81817 | down |
| MSTRG.13963 | 0.535238 | 0.68853 | 0.859236 | 0.300537 | 0.357529 | 0.274386 | 0.000201 | -1.2298 | down |

| | | | | | | | | | |
|---|---|---|---|---|---|---|---|---|---|
| TMEM52 | 3.632693 | 3.249007 | 3.303286 | 0.96462 | 0.749372 | 0.853175 | 3.51E-13 | -1.89646 | down |
| OAF | 0.905966 | 0.538772 | 0.575455 | 2.892514 | 2.336281 | 2.562147 | 7.50E-18 | 1.910389 | up |
| FAS | 3.282012 | 2.688498 | 2.611813 | 1.646078 | 0.972298 | 1.336749 | 2.21E-15 | -1.18912 | down |
| SYTL3 | 2.660489 | 2.422672 | 2.632066 | 1.293248 | 0.916694 | 1.17268 | 2.48E-12 | -1.20652 | down |
| EPHX2 | 1.820383 | 2.085458 | 1.637316 | 0.601359 | 0.71531 | 0.771748 | 4.89E-13 | -1.54071 | down |
| TEX19 | 0.638799 | 0.744886 | 0.735186 | 0.136913 | 0.030532 | 0.115384 | 1.39E-08 | -2.81468 | down |
| LRRC3B | 0.357065 | 0.169887 | 0.192726 | 3.142292 | 2.830563 | 2.715503 | 3.00E-77 | 3.472183 | up |
| ARID3A | 0.867086 | 1.21906 | 0.851585 | 0.322439 | 0.447459 | 0.610126 | 1.20E-07 | -1.1573 | down |
| CAVIN2 | 0.456416 | 0.467913 | 0.426286 | 0.061935 | 0.050748 | 0.04484 | 2.50E-09 | -2.97878 | down |
| C20orf194 | 0.392767 | 0.56519 | 0.578512 | 0.161083 | 0.24008 | 0.236829 | 5.89E-07 | -1.28476 | down |
| NINJ1 | 0.264743 | 0.135312 | 0.226039 | 1.244439 | 1.073175 | 0.851972 | 1.87E-06 | 2.236417 | up |
| SLC16A9 | 0.04061 | 0.05559 | 0 | 0.267302 | 0.313875 | 0.236226 | 3.34E-07 | 3.041228 | up |
| ARHGAP32 | 0.231432 | 0.275535 | 0.310076 | 0.134357 | 0.070176 | 0.094584 | 1.97E-06 | -1.40541 | down |
| ARHGAP39 | 0.221386 | 0.193318 | 0.246547 | 0.069755 | 0.05304 | 0.075386 | 0.00025 | -1.71943 | down |
| CMKLR1 | 0.080548 | 0.086498 | 0.02863 | 0.464525 | 0.395156 | 0.462238 | 3.36E-11 | 2.538127 | up |
| NPY1R | 5.450618 | 5.804647 | 5.416383 | 11.55771 | 11.38683 | 12.49966 | 1.41E-38 | 1.063503 | up |
| H4C8 | 1.116912 | 1.011236 | 0.874784 | 0.567329 | 0.654035 | 0.552238 | 0.002661 | -1.03321 | down |
| PPM1N | 4.464601 | 5.041793 | 3.877671 | 1.462603 | 1.866693 | 1.796574 | 1.21E-15 | -1.42034 | down |
| MS4A2 | 0.463349 | 0.267309 | 0.471492 | 0.164021 | 0.083944 | 0.166422 | 0.000296 | -1.51147 | down |
| DDR1 | 1.168949 | 1.279888 | 1.031649 | 0.269455 | 0.377619 | 0.368071 | 2.28E-14 | -1.77156 | down |
| FBXO41 | 0.262112 | 0.226293 | 0.22024 | 0.035427 | 0.016107 | 0.038029 | 1.01E-10 | -2.92868 | down |
| COL1A1 | 0.331882 | 0.456103 | 0.509549 | 0 | 0 | 0 | 2.35E-12 | -8.89782 | down |
| KIAA1217 | 0.127586 | 0.160373 | 0.095969 | 1.962848 | 0.949387 | 1.153712 | 9.48E-42 | 2.941511 | up |
| DPY19L2 | 6.321462 | 8.456165 | 7.166116 | 2.626169 | 2.852307 | 2.951665 | 3.42E-39 | -1.41374 | down |
| MSTRG.9752 | 17.47152 | 17.30853 | 18.58722 | 2.107839 | 2.284685 | 2.751768 | 0 | -2.83677 | down |
| NCKAP5 | 0.304535 | 0.193219 | 0.153875 | 0.058379 | 0.024306 | 0.008047 | 1.80E-09 | -3.08469 | down |
| IL17RB | 0.983384 | 0.935744 | 0.958008 | 0.295433 | 0.409246 | 0.528027 | 0.000143 | -1.24536 | down |
| GSTP1 | 15.32748 | 15.4328 | 16.42393 | 3.570081 | 4.012439 | 3.321595 | 2.90E-47 | -2.12563 | down |
| OSBPL6 | 1.332767 | 0.465815 | 1.152599 | 0.089765 | 0.097985 | 0.163227 | 1.43E-22 | -2.3918 | down |
| CD3EAP | 13.37334 | 13.6115 | 12.82251 | 6.512889 | 6.550947 | 6.956608 | 4.62E-45 | -1.06059 | down |
| LIN28B | 0.90737 | 0.865375 | 0.869184 | 0.183995 | 0.156974 | 0.150571 | 1.31E-23 | -2.42162 | down |
| MSTRG.1930 | 0.461334 | 0.407954 | 0.319 | 0.179763 | 0.186217 | 0.190884 | 2.16E-07 | -1.1179 | down |
| METTL27 | 1.047328 | 0.996348 | 1.064889 | 0.329831 | 0.221086 | 0.230875 | 9.51E-05 | -1.99476 | down |
| ERMP1 | 0.800439 | 1.08011 | 0.969411 | 2.069005 | 2.23318 | 2.445786 | 4.05E-19 | 1.217944 | up |
| BEX4 | 26.7255 | 26.9641 | 26.91961 | 11.14523 | 10.56542 | 10.34124 | 2.23E-65 | -1.34918 | down |
| PLEK | 1.931754 | 1.781362 | 2.103446 | 0.111035 | 0.05701 | 0.060042 | 8.01E-37 | -4.57313 | down |
| EEF1A2 | 0.247086 | 0.302888 | 0.228316 | 0.783947 | 0.720049 | 0.694356 | 0.002277 | 1.245259 | up |
| HLA-C | 8.03513 | 8.024138 | 8.209236 | 3.514409 | 3.048663 | 2.943986 | 1.68E-26 | -1.37164 | down |
| LMO3 | 17.30091 | 18.32623 | 17.19018 | 1.928774 | 2.318062 | 1.972222 | ####### # | -2.96495 | down |
| BEX1 | 11.14742 | 10.19721 | 10.41901 | 3.023434 | 3.492257 | 3.465961 | 1.67E-25 | -1.69011 | down |
| KRBA1 | 0.746927 | 0.576652 | 0.714648 | 0.079807 | 0.341457 | 0.147647 | 8.13E-10 | -2.31832 | down |
| TRIP10 | 12.00928 | 11.653 | 11.64811 | 4.358864 | 4.645727 | 3.95227 | 5.62E-50 | -1.46526 | down |
| KIF4B | 0.484996 | 0.464234 | 0.537583 | 0.236756 | 0.21381 | 0.143142 | 2.31E-05 | -1.32836 | down |
| SPATC1L | 25.12185 | 22.87616 | 20.47175 | 6.485797 | 6.022359 | 4.54717 | 2.61E-61 | -2.03163 | down |
| MSTRG.18409 | 0.65407 | 0.752268 | 0.628341 | 0.218805 | 0.271788 | 0.391483 | 0.00073 | -1.22388 | down |
| CA11 | 2.43924 | 3.080915 | 3.454621 | 1.000057 | 1.006035 | 0.949685 | 9.71E-14 | -1.62404 | down |
| DNAJB2 | 4.174417 | 4.445025 | 3.773543 | 1.92422 | 1.88157 | 1.727477 | 1.05E-20 | -1.3525 | down |
| MSTRG.15720 | 2.823233 | 2.542164 | 4.385881 | 1.795627 | 1.099913 | 1.529292 | 0.000896 | -1.15019 | down |
| PROB1 | 0.48301 | 0.656259 | 0.517814 | 0.006498 | 0.054744 | 0.045628 | 3.62E-17 | -3.90817 | down |
| PACSIN3 | 0.7248 | 1.096147 | 0.728689 | 0.130516 | 0.062123 | 0.28671 | 2.40E-07 | -2.41313 | down |
| SYBU | 2.621027 | 2.351486 | 2.296152 | 1.172682 | 0.881641 | 0.898934 | 1.14E-13 | -1.23242 | down |
| RTL6 | 1.453748 | 1.60041 | 1.454348 | 0.493576 | 0.483953 | 0.516706 | 1.35E-23 | -1.61259 | down |
| CHPF | 3.52321 | 4.018194 | 3.478299 | 1.341308 | 1.386375 | 1.530365 | 1.21E-25 | -1.4599 | down |
| ZNF619 | 0.42343 | 0.480496 | 0.552012 | 0.941382 | 1.015861 | 1.406796 | 8.12E-08 | 1.098351 | up |
| SCX | 1.633374 | 1.934441 | 1.641294 | 0.796199 | 0.746598 | 0.455037 | 3.77E-05 | -1.39477 | down |

| | | | | | | | | | | |
|---|---|---|---|---|---|---|---|---|---|---|
| FOS | 42.68523 | 46.18968 | 47.71665 | 116.3826 | 118.8548 | 110.7317 | ######## | 1.287425 | up |
| GFOD1 | 1.156159 | 1.31977 | 1.236654 | 0.285131 | 0.261522 | 0.195193 | 5.69E-42 | -2.56992 | down |
| MSTRG.19513 | 1.100123 | 1.312833 | 0.978625 | 2.27755 | 2.58262 | 3.034313 | 5.84E-06 | 1.183613 | up |
| GSTM2 | 2.333232 | 2.655015 | 2.350664 | 0.497234 | 0.673052 | 0.711736 | 1.45E-11 | -1.94093 | down |
| LPAR2 | 0.527721 | 0.691449 | 0.515062 | 0.200768 | 0.268341 | 0.189018 | 0.009722 | -1.09892 | down |
| ANXA6 | 8.73338 | 8.956298 | 9.584039 | 3.291873 | 3.513933 | 3.698345 | 8.62E-54 | -1.41259 | down |
| PWWP3B | 0.985798 | 1.1756 | 1.183414 | 0 | 0 | 0 | 5.24E-15 | -9.75107 | down |
| MSTRG.14469 | 1.181194 | 1.120461 | 1.024483 | 0.501774 | 0.589492 | 0.502965 | 2.21E-06 | -1.07649 | down |
| PYGL | 14.41373 | 14.59537 | 15.20703 | 0.393158 | 0.315414 | 0.348379 | ######## | -5.39518 | down |
| NEO1 | 2.674179 | 2.487639 | 2.713801 | 0.803203 | 0.78425 | 0.726554 | 4.93E-39 | -1.61063 | down |
| SERINC2 | 0.546089 | 0.420761 | 0.641352 | 0.199577 | 0.158481 | 0.309885 | 0.006364 | -1.22198 | down |
| KCTD6 | 2.1903 | 1.896817 | 1.552298 | 3.844261 | 3.752622 | 4.597432 | 2.58E-08 | 1.003975 | up |
| EXOC6B | 1.020335 | 0.936793 | 0.908843 | 0.26249 | 0.311688 | 0.372276 | 8.37E-16 | -1.60658 | down |
| TNS4 | 0.01967 | 0.014387 | 0.04538 | 0.740152 | 0.708683 | 0.556534 | 1.86E-17 | 4.377472 | up |
| IGSF3 | 0.071016 | 0.028278 | 0.071093 | 0.201715 | 0.198813 | 0.160961 | 0.000103 | 1.658157 | up |
| PRG2 | 153.5413 | 155.0706 | 160.1356 | 49.26708 | 45.58625 | 43.15459 | ######## | -1.78474 | down |
| TREML2 | 0.087879 | 0.070217 | 0.070318 | 0.420379 | 0.430545 | 0.339583 | 1.21E-07 | 2.283519 | up |
| MSTRG.5964 | 0.271232 | 0.302408 | 0.319441 | 1.427282 | 0.72485 | 1.007176 | 0.000273 | 1.525002 | up |
| ZNF594 | 0.384111 | 0.681345 | 0.328354 | 0.807843 | 1.035496 | 1.110736 | 4.67E-08 | 1.222009 | up |
| ZNF350 | 1.180994 | 1.273421 | 1.626375 | 3.307871 | 2.873322 | 3.514033 | 1.31E-11 | 1.155849 | up |
| KLHL6 | 5.157192 | 4.992074 | 5.392796 | 1.776899 | 1.943759 | 2.301084 | 1.66E-52 | -1.34051 | down |
| PEAR1 | 0.392575 | 0.458481 | 0.249326 | 0.856725 | 0.832391 | 0.715526 | 5.95E-06 | 1.106396 | up |
| CRISP3 | 0.467738 | 0.622081 | 0.487242 | 0.093369 | 0.20651 | 0.176058 | 0.000208 | -1.70492 | down |
| PCSK4 | 0.789348 | 1.000543 | 1.006385 | 0.377731 | 0.40337 | 0.323187 | 2.42E-06 | -1.38069 | down |
| CALB2 | 20.69469 | 22.00421 | 20.40786 | 1.601202 | 1.001522 | 1.276577 | ######## | -4.03072 | down |
| CSF1 | 0.876035 | 0.976063 | 0.931334 | 3.37176 | 3.854387 | 3.565994 | 3.27E-46 | 1.951326 | up |
| GPR68 | 0.259697 | 0.113006 | 0.080552 | 0.442406 | 0.275485 | 0.479023 | 0.004981 | 1.344672 | up |
| CTSF | 3.163695 | 3.16573 | 3.834803 | 1.638818 | 1.616528 | 1.510661 | 2.76E-10 | -1.08473 | down |
| TBX18 | 2.330697 | 2.03227 | 2.021513 | 0.080662 | 0.056813 | 0.03504 | 3.37E-76 | -4.96527 | down |
| C11orf80 | 1.212407 | 0.809634 | 1.17209 | 0.533337 | 0.696181 | 0.405057 | 0.001855 | -1.01732 | down |
| TGFBR2 | 2.205275 | 1.952128 | 2.105989 | 4.372095 | 4.691333 | 4.692624 | 6.29E-29 | 1.110267 | up |
| REC8 | 23.31821 | 24.21133 | 22.88656 | 0.193828 | 0.25928 | 0.20315 | ######## | -6.6175 | down |
| HCLS1 | 1.784363 | 1.957971 | 1.77719 | 0.25443 | 0.193669 | 0.314015 | 3.59E-21 | -2.80361 | down |
| H2BC12 | 31.2743 | 30.77929 | 30.22663 | 14.57024 | 12.2607 | 12.79751 | 1.50E-26 | -1.24241 | down |
| H2AC7 | 0.33032 | 0.759214 | 0.605291 | 2.324852 | 1.774317 | 1.667886 | 0.000858 | 1.683178 | up |
| H2AC6 | 5.271385 | 4.802408 | 5.486813 | 1.556694 | 1.7499 | 2.098607 | 4.15E-24 | -1.64333 | down |
| LAMC3 | 0.220747 | 0.358182 | 0.300661 | 0.077629 | 0.058961 | 0.08355 | 1.03E-08 | -1.99926 | down |
| ICAM3 | 7.982127 | 7.034777 | 7.483072 | 2.442765 | 3.580228 | 2.986518 | 2.25E-24 | -1.38496 | down |
| UCHL1 | 4.531877 | 3.666813 | 5.650992 | 0.46823 | 0.397735 | 0.883269 | 3.32E-23 | -2.84924 | down |
| MSTRG.20685 | 1.802805 | 2.639851 | 2.723552 | 0.474829 | 0.614163 | 0.53479 | 1.31E-13 | -2.13486 | down |
| CCNI2 | 1.038506 | 0.683909 | 0.721842 | 0.206988 | 0.228971 | 0.258309 | 1.34E-07 | -1.79933 | down |
| ZNF365 | 0.624185 | 0.438695 | 0.658755 | 0.043827 | 0.068542 | 0 | 7.64E-15 | -4.0849 | down |
| CASP4 | 2.066028 | 2.086691 | 2.517444 | 1.320552 | 0.838328 | 1.164507 | 0.000103 | -1.03312 | down |
| PRXL2A | 6.9407 | 7.468071 | 7.901923 | 3.448437 | 3.587659 | 4.306851 | 9.75E-37 | -1.08067 | down |
| SLC4A7 | 4.116512 | 4.927092 | 5.758669 | 2.266015 | 1.995621 | 3.211932 | 1.05E-18 | -1.0051 | down |
| HUNK | 0.160999 | 0.165806 | 0.177786 | 0.087008 | 0.079234 | 0.045308 | 0.002179 | -1.2466 | down |
| NCF4 | 17.09448 | 16.81987 | 16.15678 | 6.364906 | 5.760876 | 6.113286 | 1.90E-52 | -1.45077 | down |
| MSTRG.19234 | 0.595878 | 0.236754 | 0.702609 | 1.410148 | 1.518819 | 1.133141 | 0.007306 | 1.272262 | up |
| MEAK7 | 0.771846 | 0.808136 | 0.888087 | 0.381998 | 0.41534 | 0.405631 | 9.52E-07 | -1.09516 | down |
| AC004997.1 | 1.135323 | 1.511379 | 1.480072 | 0.096465 | 0.308375 | 0.049976 | 3.37E-05 | -3.11354 | down |
| RNF24 | 3.136226 | 3.515763 | 3.703365 | 7.080138 | 6.94411 | 7.831127 | 1.80E-53 | 1.056284 | up |

| | | | | | | | | | |
|---|---|---|---|---|---|---|---|---|---|
| AC104389.5 | 0.077126 | 0 | 0.21849 | 1.633758 | 1.870826 | 1.381109 | 0.00034 | 3.997936 | up |
| GABBR1 | 1.052064 | 1.074041 | 1.08554 | 0.147804 | 0.191068 | 0.218156 | 1.01E-23 | -2.49264 | down |
| NXF3 | 1.127355 | 0.950069 | 0.933372 | 0.45785 | 0.423363 | 0.404464 | 0.000107 | -1.23494 | down |
| EPPK1 | 0.07764 | 0.101394 | 0.081229 | 0.011571 | 0.030572 | 0.033391 | 5.31E-05 | -1.75783 | down |
| GPR157 | 0.441204 | 0.271826 | 0.372215 | 0.103696 | 0.131736 | 0.176085 | 7.01E-05 | -1.4013 | down |
| PPFIA3 | 3.780045 | 3.567491 | 4.289648 | 1.370527 | 1.305168 | 1.331119 | 1.77E-30 | -1.51247 | down |
| LMCD1 | 3.669775 | 4.386709 | 3.550988 | 0.959941 | 1.223053 | 1.200327 | 3.34E-73 | -1.89653 | down |
| HSD17B6 | 0.720259 | 0.731077 | 1.024232 | 0.290336 | 0.217852 | 0.241472 | 0.000419 | -1.52788 | down |
| RTN2 | 3.388118 | 2.907334 | 2.591271 | 1.808295 | 1.884185 | 1.811805 | 8.39E-08 | -1.02598 | down |
| ZNF395 | 3.399721 | 3.200216 | 4.099328 | 6.618684 | 6.594392 | 7.216286 | 2.53E-33 | 1.05831 | up |
| PKP3 | 0.162508 | 0.177419 | 0.097992 | 0.312589 | 0.539022 | 0.464919 | 0.000605 | 1.498924 | up |
| MSTRG.4994 | 0.738354 | 0.47427 | 0.62805 | 1.584041 | 1.581909 | 1.735999 | 2.37E-32 | 1.639239 | up |
| CEP192 | 2.470765 | 2.474329 | 3.01935 | 5.595842 | 5.569943 | 5.576032 | 1.49E-49 | 1.085401 | up |
| TNFRSF25 | 2.443659 | 2.325089 | 2.189715 | 1.199597 | 1.000193 | 0.655909 | 9.88E-08 | -1.31682 | down |
| RNMT | 4.178441 | 4.311734 | 4.189644 | 9.160765 | 8.346037 | 12.03554 | 1.37E-46 | 1.067318 | up |
| NOS3 | 7.441088 | 6.752849 | 6.031613 | 3.654981 | 3.595641 | 3.585712 | 2.55E-24 | -1.0908 | down |
| ARL4D | 0.926764 | 1.270066 | 1.082216 | 0.455392 | 0.39243 | 0.12377 | 1.50E-05 | -1.74846 | down |
| CBX7 | 0.773453 | 1.151776 | 1.020102 | 0.408309 | 0.253554 | 0.253976 | 5.58E-11 | -1.69339 | down |
| IGF2BP1 | 2.674337 | 2.894127 | 2.611171 | 10.48937 | 10.38318 | 11.25169 | ######## | 1.948778 | up |
| CTTNBP2NL | 0.321912 | 0.258226 | 0.293856 | 0.077938 | 0.102865 | 0.085441 | 2.96E-06 | -1.70649 | down |
| CASTOR1 | 8.891679 | 8.483601 | 9.895023 | 2.592234 | 2.175715 | 2.296875 | 4.76E-45 | -1.94046 | down |
| MAGI1 | 13.56032 | 13.88626 | 13.89226 | 7.00735 | 8.202422 | 7.185069 | ######## | -1.096 | down |
| APOE | 1.412573 | 1.11639 | 0.915894 | 0.495102 | 0.34175 | 0.674729 | 0.00378 | -1.19298 | down |
| PITPNM1 | 1.758237 | 1.954968 | 1.894884 | 0.57624 | 0.549497 | 0.555387 | 4.44E-24 | -1.70726 | down |
| MSTRG.1681 | 1.802106 | 1.874806 | 1.768517 | 0.61586 | 0.738574 | 0.6085 | 3.07E-20 | -1.46969 | down |
| GAA | 0.805961 | 0.754813 | 0.681577 | 0.506733 | 0.455447 | 0.217478 | 0.000717 | -1.01105 | down |
| TAF7L | 1.817366 | 1.696304 | 1.942109 | 0.407607 | 0.286399 | 0.320136 | 5.42E-16 | -2.19399 | down |
| PNMA6A | 1.569564 | 1.780734 | 1.802527 | 0.869488 | 0.814697 | 0.814786 | 1.18E-06 | -1.06461 | down |
| SPINT1 | 0.867228 | 0.921093 | 0.88501 | 0.071942 | 0.071147 | 0.094095 | 1.11E-15 | -3.21378 | down |
| ZNF667 | 0.423713 | 0.770347 | 0.44586 | 0.320851 | 0.245729 | 0.190463 | 8.68E-05 | -1.12044 | down |
| TSPAN4 | 13.37434 | 15.04977 | 13.33752 | 7.32617 | 6.295057 | 6.761414 | 1.58E-23 | -1.0515 | down |
| TSPAN5 | 0.643576 | 0.536925 | 0.473632 | 0.150427 | 0.168278 | 0.234023 | 8.31E-06 | -1.56405 | down |
| ZNF440 | 0.103432 | 0.146403 | 0.178589 | 0.404819 | 0.256114 | 0.512773 | 0.000917 | 1.376863 | up |
| CPA3 | 0.694569 | 0.564184 | 0.739088 | 0 | 0.15189 | 0.026756 | 0.003653 | -3.33473 | down |
| EHD4 | 1.987814 | 1.892321 | 1.859538 | 0.88044 | 0.888337 | 0.943278 | 3.49E-19 | -1.10614 | down |
| HES1 | 5.263922 | 5.706407 | 4.965058 | 1.604845 | 1.838905 | 1.783046 | 8.52E-24 | -1.62611 | down |
| MSTRG.18989 | 0.080857 | 0.049985 | 0.115401 | 0.924668 | 0.774292 | 0.882355 | 2.52E-27 | 3.268473 | up |
| ISG20 | 2.153229 | 1.256297 | 1.542401 | 0.444765 | 0.57351 | 1.19056 | 0.000911 | -1.16477 | down |
| RAB32 | 0.635957 | 0.638413 | 0.493316 | 2.075999 | 2.392768 | 2.370631 | 1.60E-09 | 1.905455 | up |
| DOK2 | 0.453869 | 0.523396 | 0.458852 | 1.116755 | 1.076602 | 1.22155 | 0.000304 | 1.147134 | up |
| RAB3D | 0.5418 | 0.548743 | 0.520609 | 0.191177 | 0.126125 | 0.166485 | 4.46E-08 | -1.72313 | down |
| CCDC120 | 1.515257 | 1.984223 | 1.494127 | 0.156919 | 0.041075 | 0.136155 | 4.45E-37 | -3.94465 | down |
| CD226 | 0.036717 | 0.038282 | 0.030367 | 0.104671 | 0.082136 | 0.075125 | 0.004459 | 1.261488 | up |
| IRX4 | 4.126631 | 3.604424 | 4.176916 | 0.063151 | 0 | 0.046256 | 3.81E-41 | -6.32154 | down |
| GPRASP2 | 0.362401 | 0.364217 | 0.367738 | 0.171584 | 0.1082 | 0.188937 | 0.001924 | -1.19082 | down |
| TP53I13 | 4.007467 | 4.029961 | 3.350826 | 1.596719 | 2.015723 | 1.875802 | 5.08E-08 | -1.11257 | down |
| TMEM198 | 0.545053 | 0.463512 | 0.488464 | 0.289204 | 0.175919 | 0.098914 | 0.002219 | -1.38939 | down |
| ALDH1A2 | 3.073086 | 3.227006 | 3.256326 | 0 | 0 | 0 | 5.15E-19 | -10.9563 | down |
| THAP8 | 3.410008 | 2.772104 | 2.786932 | 1.602977 | 1.409343 | 1.232961 | 3.19E-06 | -1.05439 | down |
| EYA4 | 0.230103 | 0.328129 | 0.337055 | 0 | 0 | 0.029568 | 1.03E-08 | -5.53993 | down |
| DBP | 1.850352 | 2.015462 | 1.377125 | 0.603837 | 0.608151 | 0.597859 | 2.19E-07 | -1.34725 | down |
| EPS8L2 | 1.652871 | 1.852038 | 1.634704 | 0.639953 | 0.602669 | 0.917704 | 3.20E-09 | -1.26799 | down |
| ABTB1 | 0.588387 | 0.472953 | 0.441118 | 0.154124 | 0.303045 | 0.08025 | 0.005619 | -1.40743 | down |
| ABTB2 | 0.177304 | 0.245679 | 0.320709 | 0.563591 | 0.53808 | 0.625546 | 1.61E-05 | 1.193687 | up |

| | | | | | | | | | |
|---|---|---|---|---|---|---|---|---|---|
| AREG | 0.240897 | 0.371821 | 0.34349 | 3.324736 | 3.378003 | 3.250955 | 8.23E-27 | 3.289506 | up |
| ARHGEF37 | 0.079044 | 0.278757 | 0.154757 | 0.500381 | 0.338707 | 0.462244 | 2.43E-05 | 1.787984 | up |
| NPTX2 | 7.496562 | 8.010839 | 7.853568 | 0.594829 | 0.443514 | 0.544249 | ######## | -3.89524 | down |
| OTUB2 | 0.433519 | 0.345013 | 0.465068 | 0.087329 | 0.035784 | 0.078418 | 3.29E-08 | -2.46773 | down |
| MSTRG.9545 | 0.591068 | 0.6096 | 0.459725 | 1.566701 | 1.399932 | 1.545977 | 1.06E-11 | 1.406216 | up |
| H1-0 | 0.533319 | 0.861412 | 1.004699 | 0.079771 | 0.166854 | 0.190763 | 6.33E-09 | -2.42035 | down |
| CD3D | 0.737387 | 0.785941 | 0.692761 | 2.217075 | 1.951339 | 1.496853 | 0.002934 | 1.235335 | up |
| MSTRG.15576 | 1.477412 | 1.839868 | 1.693587 | 0.257974 | 0.443866 | 0.136904 | 4.61E-12 | -2.4066 | down |
| NANOS1 | 0.502532 | 0.624166 | 0.285746 | 0.253137 | 0.225702 | 0.381644 | 0.003607 | -1.31535 | down |
| ALDH1A1 | 1.05997 | 1.013363 | 1.09884 | 3.57713 | 3.566899 | 4.003297 | 1.40E-25 | 1.784097 | up |
| RBP5 | 0.440043 | 0.631781 | 1.028496 | 1.617884 | 1.602251 | 1.904243 | 0.003647 | 1.190308 | up |
| CCR10 | 2.559266 | 2.292126 | 2.112856 | 0.814107 | 0.918007 | 0.599238 | 1.44E-11 | -1.59902 | down |
| ADAM8 | 1.472395 | 1.768053 | 1.474352 | 0.705355 | 0.744669 | 0.537043 | 3.13E-10 | -1.29622 | down |
| EGFL7 | 17.14119 | 16.96398 | 18.71966 | 5.052181 | 5.596534 | 3.725777 | 3.59E-67 | -1.93734 | down |
| CRIP2 | 2.033604 | 1.997252 | 2.181088 | 1.168365 | 0.687744 | 0.71568 | 1.24E-06 | -1.46547 | down |
| MSTRG.1188 | 1.442734 | 1.692523 | 1.583373 | 0.160282 | 0.206457 | 0.079854 | 3.69E-22 | -3.37782 | down |
| ZNF629 | 0.560964 | 0.521904 | 0.597587 | 1.70267 | 1.66549 | 1.710843 | 1.38E-27 | 1.566232 | up |
| OR2A4 | 1.983115 | 1.997924 | 1.762606 | 0.514063 | 0.566431 | 0.48467 | 6.67E-13 | -1.89211 | down |
| OR2A7 | 1.300877 | 1.240868 | 1.228978 | 0.502735 | 0.351585 | 0.276993 | 2.23E-08 | -1.71889 | down |
| TRAF3 | 1.745794 | 2.063969 | 1.779812 | 4.16411 | 4.414485 | 4.426956 | 4.32E-47 | 1.197386 | up |
| HOOK2 | 2.348136 | 2.2976 | 2.201999 | 1.060812 | 1.27389 | 0.892889 | 2.10E-08 | -1.08806 | down |
| TBC1D10A | 8.645314 | 7.872583 | 8.882923 | 3.840699 | 4.3178 | 3.739477 | 7.11E-23 | -1.10451 | down |
| MSTRG.19364 | 3.579552 | 3.4504 | 4.01685 | 1.911142 | 1.252486 | 1.291366 | 3.98E-11 | -1.33115 | down |
| PPARD | 2.008094 | 1.808612 | 1.689824 | 4.535343 | 3.725159 | 3.693337 | 3.81E-17 | 1.084722 | up |
| DEPTOR | 1.217223 | 1.460506 | 1.07428 | 0.588008 | 0.598163 | 0.532417 | 7.80E-06 | -1.17978 | down |
| PRRG2 | 0.639208 | 1.098324 | 0.757067 | 0.239664 | 0.409925 | 0.182187 | 0.001063 | -1.52213 | down |
| MSTRG.12897 | 0.12596 | 0.213128 | 0.144182 | 0.313449 | 0.38659 | 0.601756 | 0.001248 | 1.375316 | up |
| AC010442.3 | 0.089688 | 0.045825 | 0.055099 | 0.172527 | 0.190791 | 0.197003 | 0.00109 | 1.497818 | up |
| PTGES | 1.920148 | 1.977197 | 1.859496 | 0.523097 | 0.473041 | 0.493485 | 1.93E-13 | -1.93481 | down |
| CSGALNACT1 | 0.314388 | 0.315576 | 0.280413 | 0.073856 | 0.133413 | 0.170713 | 0.002453 | -1.26013 | down |
| LPIN2 | 2.145308 | 2.118717 | 2.500789 | 4.724321 | 4.761909 | 4.668308 | 3.23E-34 | 1.062024 | up |
| N4BP3 | 0.265207 | 0.241385 | 0.238618 | 0.89096 | 0.782035 | 0.709605 | 9.27E-14 | 1.636849 | up |
| MAP3K10 | 0.625176 | 0.729685 | 0.461131 | 0.208028 | 0.271319 | 0.269267 | 0.000139 | -1.20396 | down |
| ZNF541 | 0.48691 | 0.474643 | 0.636112 | 0.091414 | 0.111008 | 0.133323 | 1.23E-11 | -2.25765 | down |
| C2orf74 | 3.864296 | 4.945537 | 5.676866 | 1.742472 | 1.484246 | 1.072219 | 1.84E-10 | -1.60094 | down |
| JAKMIP1 | 5.473237 | 5.526461 | 5.5578 | 2.375454 | 2.324672 | 2.255177 | 7.33E-28 | -1.27134 | down |
| IMPA2 | 1.475745 | 1.005949 | 0.863058 | 3.015379 | 3.400494 | 2.911072 | 2.51E-10 | 1.433644 | up |
| NMT2 | 5.454844 | 5.495759 | 5.372087 | 1.727777 | 1.756504 | 1.659118 | 1.17E-60 | -1.63374 | down |
| SNAI1 | 1.434303 | 1.053665 | 1.277365 | 0.345281 | 0.33669 | 0.388418 | 3.94E-08 | -1.83087 | down |
| SLC2A3 | 0.937636 | 0.841804 | 0.659397 | 4.306334 | 3.883935 | 4.674626 | 4.21E-63 | 2.364517 | up |
| HPGD | 247.5358 | 251.5395 | 251.4335 | 78.68358 | 80.82981 | 87.4227 | 0 | -1.66474 | down |
| SLC46A3 | 0.067535 | 0.0498 | 0.074851 | 0.580134 | 0.607435 | 0.587406 | 1.16E-12 | 3.074549 | up |
| MOCOS | 0.592494 | 0.57892 | 0.517916 | 0.122355 | 0.148256 | 0.109371 | 3.39E-15 | -2.1373 | down |
| FAM81A | 0.134002 | 0.059116 | 0.437653 | 0.938163 | 1.100568 | 0.700152 | 5.83E-13 | 2.592116 | up |
| SLFN14 | 1.537168 | 1.632961 | 1.734592 | 0.664552 | 0.763249 | 0.855735 | 2.97E-17 | -1.13167 | down |
| PGGHG | 0.418908 | 0.771776 | 0.663188 | 0.323176 | 0.379893 | 0.174691 | 0.000932 | -1.16039 | down |
| NRARP | 1.334623 | 1.043283 | 0.835549 | 0.422492 | 0.539493 | 0.330274 | 7.45E-06 | -1.32375 | down |
| CYP17A1 | 1.335329 | 1.769865 | 1.921863 | 0.059947 | 0 | 0.033586 | 3.31E-17 | -5.64092 | down |
| ARMH4 | 1.563843 | 1.572531 | 1.70202 | 0.759665 | 0.710181 | 0.641001 | 1.18E-16 | -1.21507 | down |
| TWSG1 | 4.381929 | 4.757864 | 5.491841 | 8.902317 | 10.35115 | 10.86926 | 2.79E-29 | 1.02041 | up |
| PPP4R1 | 5.800225 | 6.165498 | 5.766564 | 11.9365 | 11.76611 | 12.59222 | 1.28E-47 | 1.007746 | up |
| APOC1 | 52.50764 | 52.88075 | 53.25463 | 17.07496 | 18.94395 | 15.76276 | 3.73E-83 | -1.66896 | down |
| SPR | 8.665058 | 9.514974 | 8.112816 | 4.195733 | 3.498015 | 3.483251 | 3.29E-21 | -1.25619 | down |
| TMEM45A | 1.363064 | 1.211148 | 1.595747 | 0.547195 | 0.78057 | 0.612529 | 0.00073 | -1.00322 | down |
| AKNA | 0.27251 | 0.190974 | 0.174814 | 0.082092 | 0.139496 | 0.062698 | 0.003633 | -1.14317 | down |

| | | | | | | | | | |
|---|---|---|---|---|---|---|---|---|---|
| MSTRG.17120 | 0.374523 | 0.371094 | 0.266644 | 0.57044 | 0.680971 | 0.876969 | 0.001459 | 1.026989 | up |
| ST8SIA1 | 0.215834 | 0.383156 | 0.409614 | 0.979277 | 1.004465 | 0.636381 | 0.000317 | 1.193448 | up |
| STX1B | 0.348896 | 0.387966 | 0.31321 | 0.178591 | 0.175114 | 0.126406 | 0.001179 | -1.1347 | down |
| LIPA | 26.90243 | 30.48993 | 28.73023 | 5.218069 | 5.698128 | 6.273712 | ######## | -2.34995 | down |
| SCN2A | 8.051696 | 8.773763 | 9.500619 | 0.088633 | 0.093692 | 0.092351 | ######## | -6.60418 | down |
| MSTRG.10290 | 0.586069 | 0.784236 | 0.649215 | 0.307524 | 0.21579 | 0.141071 | 0.0017 | -1.55029 | down |
| RNASEL | 1.330784 | 1.222212 | 1.322956 | 0.763238 | 0.461276 | 0.683437 | 2.71E-07 | -1.03776 | down |
| ROBO2 | 3.558916 | 4.287624 | 4.050662 | 0 | 0.013186 | 0.010266 | 2.21E-56 | -8.34966 | down |
| ELOVL2 | 11.79752 | 12.29813 | 12.11495 | 4.694028 | 5.250792 | 5.032494 | 7.41E-77 | -1.29701 | down |
| MFSD13A | 3.055695 | 3.000912 | 2.878558 | 1.348472 | 1.328779 | 1.351988 | 1.65E-15 | -1.27275 | down |
| HSPA2 | 2.310127 | 2.054026 | 2.543667 | 1.230887 | 1.082953 | 0.798708 | 1.20E-09 | -1.16502 | down |
| TMEM35B | 4.55697 | 4.636976 | 4.10596 | 1.966049 | 1.688317 | 2.385597 | 1.45E-07 | -1.1619 | down |
| DUSP2 | 19.17028 | 18.82067 | 20.01949 | 8.712877 | 8.179624 | 7.768998 | 2.49E-51 | -1.25643 | down |
| HDX | 0.384752 | 0.407897 | 0.659025 | 0 | 0 | 0 | 2.51E-12 | -8.87636 | down |
| TBC1D12 | 3.425152 | 3.517809 | 3.623005 | 1.607075 | 1.763315 | 1.963199 | 8.43E-24 | -1.01122 | down |
| HSPA1A | 58.71656 | 59.7047 | 54.61722 | 25.86122 | 25.16449 | 24.30784 | ######## | -1.22362 | down |
| HSPA1L | 1.135486 | 0.905257 | 0.703196 | 0.27942 | 0.279212 | 0.232988 | 2.15E-08 | -1.77443 | down |
| AHNAK2 | 1.89544 | 2.059594 | 1.731264 | 0.102766 | 0.147953 | 0.217283 | 3.82E-42 | -2.24549 | down |
| SEMA4A | 0.492674 | 0.529111 | 0.394749 | 0.263416 | 0.134387 | 0.127639 | 0.000234 | -1.4553 | down |
| CTAGE9 | 0.395183 | 0.477024 | 0.490754 | 0.162974 | 0.147825 | 0.226613 | 0.001804 | -1.32892 | down |
| MSTRG.12441 | 0.502219 | 0.43283 | 0.416061 | 0.171119 | 0.096578 | 0.180053 | 9.96E-09 | -1.55258 | down |
| CDC42BPB | 0.331488 | 0.427532 | 0.458336 | 0.184639 | 0.164123 | 0.179375 | 9.75E-06 | -1.19551 | down |
| NPW | 13.48276 | 12.86085 | 11.0876 | 2.165079 | 2.937076 | 1.720831 | 8.25E-39 | -2.46051 | down |
| KYAT1 | 6.665436 | 7.029122 | 3.880889 | 2.970968 | 2.519122 | 3.034663 | 6.34E-09 | -1.06916 | down |
| ZNF229 | 0.752832 | 0.98523 | 0.867412 | 0.391092 | 0.385481 | 0.355176 | 5.04E-08 | -1.13834 | down |
| AKAP12 | 0.214084 | 0.270611 | 0.367701 | 0.108293 | 0.07546 | 0.119615 | 9.19E-06 | -1.40525 | down |
| MSTRG.4018 | 0.466113 | 0.697702 | 0.785631 | 1.570674 | 1.494323 | 1.70796 | 1.47E-07 | 1.263101 | up |
| AACS | 9.112885 | 10.2298 | 9.64482 | 3.735671 | 3.928445 | 3.726928 | 8.82E-59 | -1.3692 | down |
| NPR3 | 4.632201 | 4.264946 | 4.960425 | 0 | 0.009819 | 0 | 2.54E-30 | -9.85777 | down |
| ANK3 | 0.334837 | 0.144854 | 0.042195 | 0.536955 | 0.383152 | 0.087658 | 0.002824 | 1.025873 | up |
| MGAT4A | 0.20529 | 0.054066 | 0.069184 | 1.221095 | 1.316256 | 1.218523 | 6.56E-48 | 3.639673 | up |
| LRRC4 | 1.138194 | 1.233962 | 1.17425 | 0.585966 | 0.513949 | 0.298075 | 2.21E-09 | -1.40032 | down |
| AC022415.2 | 0.212629 | 0.165442 | 0.190613 | 0.048253 | 0.03688 | 0.044244 | 3.95E-05 | -2.0976 | down |
| CDH6 | 8.204275 | 8.862067 | 8.60423 | 26.42699 | 25.41411 | 27.88126 | ######## | 1.567631 | up |
| BHLHE40 | 0.906791 | 0.689468 | 0.886145 | 0.353749 | 0.255349 | 0.281069 | 3.36E-07 | -1.49576 | down |
| PLEKHA5 | 1.044614 | 0.986214 | 1.063427 | 2.58517 | 2.311612 | 2.807965 | 1.34E-15 | 1.105376 | up |
| PGBD1 | 1.799775 | 2.217155 | 2.686175 | 4.687742 | 5.254959 | 5.092024 | 1.42E-18 | 1.141265 | up |
| CILP2 | 2.15554 | 2.098464 | 2.04514 | 0.224717 | 0.166466 | 0.149381 | 1.79E-58 | -3.53444 | down |
| MSTRG.5183 | 1.064159 | 0.554948 | 0.899022 | 1.495135 | 1.934891 | 1.83718 | 0.00494 | 1.005943 | up |
| AC023055.1 | 2.725593 | 3.591329 | 6.77529 | 0.249795 | 0.104512 | 0.114071 | 4.83E-18 | -5.61129 | down |
| MACC1 | 0.519619 | 0.65272 | 0.562368 | 1.29518 | 1.257003 | 1.712341 | 3.28E-19 | 1.185145 | up |
| GDF15 | 3.343657 | 3.721098 | 2.992183 | 1.339831 | 1.024374 | 1.129867 | 3.50E-10 | -1.47548 | down |
| CNNM1 | 3.086127 | 3.204613 | 3.139798 | 0 | 0 | 0 | 1.18E-21 | -11.696 | down |
| TSLP | 0.69611 | 0.515719 | 0.548099 | 0.290626 | 0.213186 | 0.136536 | 0.000333 | -1.4637 | down |
| IRF6 | 7.789133 | 8.166913 | 7.564852 | 2.096407 | 2.027834 | 2.327651 | ######## | -1.97933 | down |
| FMNL2 | 1.41514 | 1.424474 | 1.212559 | 0.113306 | 0.053798 | 0.088449 | 4.82E-51 | -3.95758 | down |
| STAP2 | 1.162067 | 1.789132 | 1.49184 | 0.577266 | 0.868403 | 0.848683 | 0.001041 | -1.00065 | down |
| IFI6 | 3.188612 | 2.698312 | 3.075517 | 1.211054 | 1.425937 | 1.473074 | 4.32E-05 | -1.14125 | down |
| GUCY1B1 | 0.364071 | 0.512553 | 0.362404 | 0.023226 | 0.024148 | 0.054583 | 2.94E-09 | -3.40236 | down |
| FNBP1L | 2.972663 | 3.325974 | 3.295444 | 1.479967 | 1.546473 | 1.661574 | 1.72E-22 | -1.04053 | down |
| PARP3 | 0.985912 | 1.246105 | 0.841937 | 0.248142 | 0.31644 | 0.27657 | 4.65E-09 | -1.86086 | down |

| | | | | | | | | | |
|---|---|---|---|---|---|---|---|---|---|
| GSPT2 | 0.020948 | 0 | 0 | 0.522846 | 0.316036 | 0.30024 | 1.18E-06 | 5.74347 | up |
| ZNF35 | 1.438043 | 1.528067 | 1.326335 | 3.301101 | 3.928521 | 3.842378 | 1.30E-19 | 1.343682 | up |
| ITPRIPL1 | 0.654898 | 0.519906 | 0.500768 | 0.260191 | 0.261926 | 0.271914 | 0.000504 | -1.08023 | down |
| YES1 | 2.375032 | 2.439387 | 2.544771 | 6.327854 | 6.611255 | 7.794592 | 2.24E-56 | 1.468556 | up |
| INA | 0.326445 | 0.403085 | 0.452375 | 0.029909 | 0 | 0.061081 | 4.43E-09 | -3.57986 | down |
| MSTRG.7291 | 7.140353 | 6.792396 | 6.641823 | 0.622879 | 0.746046 | 0.670176 | 1.34E-71 | -3.28851 | down |
| MAMDC4 | 1.699143 | 1.869945 | 1.747362 | 0.532211 | 0.984199 | 0.703538 | 4.10E-10 | -1.20412 | down |
| HBG2 | 29.43483 | 34.8503 | 34.58412 | 4845.995 | 4838.196 | 4376.545 | 0 | 7.127138 | up |
| CARMIL3 | 0.291483 | 0.222138 | 0.243851 | 0.058846 | 0.101406 | 0.043361 | 7.33E-05 | -1.86201 | down |
| CARMIL2 | 0.898875 | 0.895259 | 1.014327 | 0.14703 | 0.154503 | 0.159652 | 5.45E-21 | -2.56469 | down |
| ADGRA3 | 0.563677 | 0.586275 | 0.569137 | 0 | 0 | 0 | 2.00E-12 | -8.8996 | down |
| AGTRAP | 70.10504 | 72.41514 | 70.41352 | 34.74168 | 34.08847 | 33.31169 | 2.58E-80 | -1.08075 | down |
| UBASH3A | 0.721518 | 0.83845 | 0.621297 | 5.910276 | 5.673512 | 4.522458 | 1.51E-61 | 2.832841 | up |
| MSTRG.13780 | 2.686403 | 2.441551 | 2.639339 | 0.598271 | 0.519465 | 0.510475 | 1.24E-28 | -2.25375 | down |
| SMPX | 5.660929 | 3.883615 | 3.821659 | 0 | 0 | 0 | 4.14E-14 | -9.48748 | down |
| LTB4R2 | 0.483062 | 0.396287 | 0.6022 | 1.019321 | 1.011325 | 0.981866 | 0.000158 | 1.029418 | up |
| ZNF285 | 0.364323 | 0.663762 | 0.52797 | 0.113996 | 0.077976 | 0.091095 | 7.51E-11 | -2.12102 | down |
| ARNTL | 1.508272 | 1.378387 | 1.528449 | 3.593871 | 3.334182 | 3.841437 | 8.86E-19 | 1.262082 | up |
| GPX3 | 0.10583 | 0.081183 | 0.133909 | 0.706356 | 1.110427 | 0.86669 | 5.73E-09 | 2.896886 | up |
| TUBB6 | 41.85295 | 40.44367 | 40.01001 | 95.73277 | 85.84609 | 80.05589 | 3.77E-81 | 1.036652 | up |
| RELL2 | 9.177144 | 8.573028 | 8.698606 | 4.354512 | 4.089083 | 4.112115 | 2.91E-29 | -1.13765 | down |
| ABCA3 | 0.662332 | 0.456562 | 0.548916 | 0.187315 | 0.089186 | 0.081924 | 1.48E-10 | -2.00652 | down |
| ZNF14 | 0.285446 | 0.51721 | 0.623971 | 0.080909 | 0.092119 | 0.129571 | 2.28E-06 | -2.18456 | down |
| RTN4RL2 | 2.446352 | 2.881423 | 2.435413 | 1.267544 | 1.137228 | 0.951528 | 8.91E-11 | -1.23417 | down |
| MSTRG.8828 | 1.185265 | 1.01115 | 1.145923 | 2.81791 | 2.936435 | 3.328356 | 1.69E-25 | 1.416776 | up |
| ACOT4 | 0.366196 | 0.538449 | 0.638389 | 0.304904 | 0.169335 | 0.225691 | 0.007756 | -1.13942 | down |
| SPTAN1 | 1.60733 | 1.970492 | 1.944965 | 0.69459 | 0.539876 | 0.59199 | 4.21E-41 | -1.95092 | down |
| PRR29 | 0.425267 | 0.529347 | 1.032922 | 0.313568 | 0.26526 | 0.373989 | 0.004219 | -1.05457 | down |
| NCR3 | 0.446695 | 0.716232 | 0.495462 | 1.888604 | 1.388979 | 1.111688 | 0.000812 | 1.323637 | up |
| PRSS16 | 0.719335 | 0.592536 | 0.736906 | 0.214415 | 0.265486 | 0.275199 | 7.05E-06 | -1.47791 | down |
| MSTRG.18145 | 2.044108 | 2.686001 | 2.740088 | 6.037628 | 6.251568 | 5.995486 | 4.32E-16 | 1.242255 | up |
| FAM95C | 0.343099 | 0.482987 | 0.330039 | 1.791793 | 2.040717 | 1.973604 | 2.48E-13 | 2.166585 | up |
| SYT11 | 0.670518 | 0.736556 | 0.698512 | 0.239917 | 0.192753 | 0.262981 | 4.46E-11 | -1.60866 | down |
| NDC80 | 10.43923 | 11.25934 | 13.0312 | 25.06137 | 24.87985 | 27.66648 | 1.00E-49 | 1.120032 | up |
| MSTRG.4028 | 0.781994 | 0.881746 | 0.944722 | 0.181334 | 0.273797 | 0.264526 | 4.36E-10 | -1.76829 | down |
| SOX7 | 0.496103 | 0.364716 | 0.543118 | 0.32845 | 0.208203 | 0.169425 | 0.009649 | -1.00154 | down |
| SOX4 | 1.110879 | 1.228306 | 1.101223 | 0.434964 | 0.298128 | 0.324045 | 6.98E-17 | -1.71535 | down |
| ZNF724 | 0.411605 | 0.360503 | 0.373777 | 0.975308 | 1.211006 | 1.382142 | 1.05E-09 | 1.606323 | up |
| CD69 | 0.933421 | 0.819761 | 1.345038 | 3.765173 | 2.742748 | 2.684086 | 3.23E-10 | 1.460229 | up |
| TNFRSF11A | 0.118862 | 0.065467 | 0.104409 | 0.248562 | 0.171868 | 0.211392 | 0.000534 | 1.26069 | up |
| TRIO | 0.23742 | 0.276607 | 0.193947 | 0.010047 | 0.014211 | 0.029152 | 3.69E-18 | -3.69232 | down |
| MSTRG.3844 | 1.645332 | 1.673451 | 1.431618 | 0.665631 | 0.417995 | 0.766917 | 0.000609 | -1.26013 | down |
| FES | 3.871545 | 4.297816 | 4.375863 | 1.8082 | 2.437447 | 1.886466 | 4.74E-12 | -1.04798 | down |
| CERS4 | 2.017808 | 1.655905 | 1.875869 | 0.859579 | 0.787192 | 1.054251 | 9.94E-05 | -1.00995 | down |
| LINGO2 | 2.086207 | 2.080107 | 1.967135 | 0.644338 | 0.818522 | 1.054009 | 9.21E-12 | -1.29941 | down |
| SNTG1 | 1.887899 | 1.565134 | 1.766143 | 0.032453 | 0.034201 | 0.019302 | 2.16E-20 | -4.10286 | down |
| FAM13A | 0.314022 | 0.286406 | 0.339519 | 1.618774 | 1.536285 | 1.420076 | 2.06E-25 | 1.995152 | up |
| PIK3IP1 | 0.555095 | 0.458971 | 0.576234 | 0.328112 | 0.160394 | 0.247807 | 0.004674 | -1.16059 | down |
| AP001931.2 | 0.632037 | 0.577433 | 0.405108 | 1.016887 | 1.317763 | 1.593572 | 4.13E-10 | 1.25685 | up |
| DNAAF3 | 6.917484 | 6.538673 | 3.602261 | 1.299205 | 1.429496 | 2.066925 | 1.09E-20 | -1.48126 | down |
| DNAAF4 | 5.460553 | 5.403705 | 5.158679 | 2.315126 | 2.454746 | 2.402214 | 5.26E-14 | -1.10069 | down |
| CHRNB1 | 0.692593 | 0.699637 | 0.753928 | 0.378731 | 0.317532 | 0.328701 | 0.000345 | -1.40197 | down |
| GPHN | 2.939924 | 3.018237 | 3.09366 | 6.729691 | 6.700907 | 6.503669 | 3.05E-34 | 1.11389 | up |
| RHEX | 149.9834 | 155.5868 | 153.9401 | 41.01562 | 40.48867 | 40.92666 | 0 | -1.92794 | down |
| GABRB2 | 0.234357 | 0.19698 | 0.163045 | 0.02458 | 0.032318 | 0.028894 | 1.11E-08 | -2.67629 | down |
| CD274 | 0.72091 | 0.665662 | 0.679954 | 1.438551 | 1.906415 | 1.557971 | 6.11E-11 | 1.253442 | up |

| | | | | | | | | | |
|---|---|---|---|---|---|---|---|---|---|
| LTB4R | 1.303716 | 1.707522 | 1.63136 | 3.047499 | 3.448478 | 3.260289 | 2.42E-15 | 1.008497 | up |
| ZNF71 | 0.196061 | 0.25637 | 0.201092 | 0.099573 | 0.072997 | 0.050833 | 0.004686 | -1.29815 | down |
| ZNF77 | 0.31189 | 0.498887 | 0.4303 | 0.862075 | 1.257567 | 1.242911 | 1.21E-05 | 1.395859 | up |
| SOCS3 | 0.150921 | 0.182734 | 0.103357 | 0.539794 | 0.4574 | 0.588441 | 9.79E-06 | 1.824294 | up |
| DISP2 | 0.096912 | 0.075791 | 0.110427 | 0.01584 | 0.0381 | 0.037186 | 0.000401 | -1.61815 | down |
| HBG1 | 11.02111 | 10.34227 | 12.05765 | 1261.613 | 1223.784 | 1162.746 | 0 | 6.742997 | up |
| SLC2A6 | 1.085948 | 1.115143 | 1.149278 | 0.162539 | 0.42748 | 0.169346 | 1.29E-09 | -2.08004 | down |
| PLPPR2 | 2.274879 | 2.303571 | 1.762638 | 0.639377 | 0.524134 | 0.592625 | 6.38E-19 | -1.86268 | down |
| CPLANE2 | 1.235711 | 1.08725 | 0.840126 | 0.440418 | 0.686133 | 0.384829 | 0.003528 | -1.07231 | down |
| CPLANE1 | 0.053464 | 0.080067 | 0.074864 | 0.246104 | 0.269831 | 0.297362 | 9.58E-11 | 1.873956 | up |
| IKZF3 | 3.209284 | 3.188093 | 3.205368 | 6.305988 | 6.829239 | 7.314874 | 1.11E-60 | 1.063253 | up |
| IKZF2 | 0.494192 | 0.477958 | 0.593804 | 0.158688 | 0.040018 | 0.044025 | 8.49E-27 | -3.19414 | down |
| SLC14A1 | 13.13861 | 12.09363 | 12.41861 | 5.098934 | 5.829667 | 5.809519 | 3.51E-69 | -1.27131 | down |
| TNFRSF10D | 0.747109 | 0.774503 | 0.774909 | 0.240533 | 0.23058 | 0.265396 | 3.39E-09 | -1.66276 | down |
| TCEAL9 | 2.612692 | 2.754272 | 2.35218 | 1.073771 | 1.045762 | 1.38563 | 0.000153 | -1.03663 | down |
| RGS16 | 1.233758 | 1.335426 | 1.637224 | 0.708097 | 0.606089 | 0.745822 | 1.30E-05 | -1.05041 | down |
| CRYAB | 3.033526 | 2.82314 | 3.083594 | 0.064533 | 0.375813 | 0.210375 | 1.36E-22 | -3.86545 | down |
| CHDH | 0.188226 | 0.180941 | 0.212904 | 0.051189 | 0.068055 | 0.043713 | 4.23E-06 | -1.84051 | down |
| LDHD | 3.941602 | 3.650342 | 3.540739 | 0.212537 | 0.33889 | 0.308051 | 4.75E-51 | -3.68551 | down |
| SLC7A11 | 1.557123 | 1.675283 | 1.685714 | 3.306306 | 3.244897 | 3.862816 | 6.78E-35 | 1.054486 | up |
| SERPINH1 | 29.3975 | 44.22236 | 31.49992 | 15.34231 | 20.80284 | 19.60757 | 1.03E-75 | -1.09353 | down |

Table S5. Differentially expression genes of proteomics.

| Differentially expression genes of proteomics | | | | | | | | | | |
|---|---|---|---|---|---|---|---|---|---|---|
| Gene | WT_rep.1 | WT_rep.2 | WT_rep.3 | HBB.KO_rep.1 | HBB.KO_rep.2 | HBB.KO_rep.3 | FC(HBB-KO vs WT) | Pvalue | log10Pvalue | log2FC | sig |
| HBG2 | 43.8 | 46.8 | 49.1 | 153.7 | 155.6 | 151 | 3.294918 | 7.85E-07 | 6.10513 | 1.720242 | up |
| HBG1 | 41.6 | 46.3 | 50.1 | 155.2 | 152.1 | 154.7 | 3.347826 | 2.13E-06 | 5.67162 | 1.743225 | up |
| SLC4A1 | 62.6 | 61.5 | 61 | 135.6 | 141.1 | 138.1 | 2.240951 | 1.32E-06 | 5.879426 | 1.164111 | up |
| EPB42 | 68.7 | 64.2 | 59.9 | 134.3 | 137.7 | 135.2 | 2.112033 | 1.28E-05 | 4.89279 | 1.078633 | up |
| EHD3 | 49.4 | 44.3 | 44.9 | 142.4 | 161.1 | 157.8 | 3.328283 | 5.63E-05 | 4.249492 | 1.734778 | up |
| HBE1 | 45.2 | 46 | 48.5 | 158.6 | 149.1 | 152.5 | 3.294202 | 3.48E-06 | 5.458421 | 1.719929 | up |
| PKP3 | 61.4 | 61.4 | 61.9 | 146.5 | 132.3 | 136.6 | 2.249053 | 5.27E-05 | 4.278189 | 1.169317 | up |
| ISG20 | 55.5 | 48.1 | 40.3 | 122.8 | 171 | 162.4 | 3.170257 | 0.002545 | 2.594277 | 1.6646 | up |
| HBM | 48 | 53.3 | 48.6 | 157 | 141.8 | 151.2 | 3.002001 | 2.97E-05 | 4.527244 | 1.585925 | up |
| TNS1 | 62.1 | 67.6 | 66.5 | 119.9 | 144.9 | 139 | 2.058104 | 0.000861 | 3.064933 | 1.041316 | up |
| ARG1 | 49.2 | 54.2 | 48.8 | 118.2 | 168.7 | 161.1 | 2.943495 | 0.003373 | 2.472001 | 1.55753 | up |
| SLC2A3 | 62.9 | 62.4 | 68.7 | 124.3 | 143.3 | 138.5 | 2.093299 | 0.000307 | 3.51288 | 1.065778 | up |
| TPPP3 | 77.7 | 41.9 | 46.1 | 153.6 | 140.7 | 140 | 2.621002 | 0.001798 | 2.745321 | 1.390118 | up |
| CALML3 | 44.4 | 47 | 98.3 | 121.3 | 151.3 | 137.7 | 2.162889 | 0.019837 | 1.702532 | 1.112959 | up |

| | | | | | | | | | | | |
|---|---|---|---|---|---|---|---|---|---|---|---|
| DUS4L | 48.5 | 51.6 | 54.8 | 148.4 | 146.3 | 150.3 | 2.872821 | 1.47E-06 | 5.832683 | 1.522468 | up |
| TFEB | 50 | 60.4 | 71.9 | 114.8 | 149.6 | 153.3 | 2.291278 | 0.004721 | 2.325923 | 1.196153 | up |
| APOA1 | 72.1 | 63.5 | 55.2 | 111.9 | 142.9 | 154.4 | 2.144654 | 0.00587 | 2.231372 | 1.100745 | up |
| SLC35D3 | 69.6 | 26 | 20.7 | 130.1 | 175.3 | 178.3 | 4.159071 | 0.005084 | 2.293815 | 2.056261 | up |
| CSPG4 | 77.3 | 44.1 | 67.2 | 151.2 | 115.4 | 144.9 | 2.181866 | 0.00734 | 2.134306 | 1.125563 | up |
| PPP1R14A | 45.5 | 51.7 | 55.7 | 115.7 | 169 | 162.5 | 2.924787 | 0.004524 | 2.344509 | 1.548332 | up |
| ARHGEF37 | 66.4 | 73.6 | 59.2 | 134 | 119.9 | 147 | 2.01255 | 0.001618 | 2.790975 | 1.009025 | up |
| INS | 68.7 | 64.3 | 52.7 | 88 | 180.4 | 146 | 2.231556 | 0.049607 | 1.304456 | 1.15805 | up |
| RHCE | 41.6 | 37.8 | 28 | 153.8 | 180.1 | 158.7 | 4.586592 | 0.000142 | 3.846749 | 2.197423 | up |
| FRMD4A | 40.8 | 82.6 | 63.2 | 144.3 | 132.9 | 136.1 | 2.214898 | 0.003827 | 2.417144 | 1.14724 | up |
| SER-PINA2 | 7 | 8.1 | 8.5 | 226.8 | 151.5 | 198.1 | 24.42373 | 0.001101 | 2.958237 | 4.610212 | up |
| IQCE | 57.8 | 68.3 | 69.7 | 130.6 | 138.4 | 135.2 | 2.064351 | 9.28E-05 | 4.032452 | 1.045689 | up |
| MARCOL | 70 | 55 | 62 | 105.6 | 153.9 | 153.6 | 2.209091 | 0.01055 | 1.976734 | 1.143453 | up |
| HBB | 171.2 | 162.1 | 166.2 | 33 | 33.7 | 33.8 | 0.201201 | 9.34E-07 | 6.029653 | -2.31329 | down |
| PYGL | 123.9 | 158.5 | 150.2 | 56.2 | 56.1 | 55.1 | 0.386963 | 0.001064 | 2.973011 | -1.36973 | down |
| SER-PINB1 | 142.7 | 152.3 | 148 | 54.4 | 53.6 | 49 | 0.354402 | 8.01E-06 | 5.096367 | -1.49654 | down |
| AKR1B1 | 130.1 | 140.8 | 133 | 67 | 63.6 | 65.6 | 0.485764 | 3.21E-05 | 4.493495 | -1.04167 | down |
| PFKFB2 | 149.6 | 146 | 136.3 | 53.6 | 57.3 | 57.2 | 0.38921 | 2.94E-05 | 4.531653 | -1.36138 | down |
| ROBO2 | 143.4 | 149.2 | 138.6 | 53.4 | 55.9 | 59.5 | 0.391466 | 1.59E-05 | 4.798603 | -1.35304 | down |
| SLFN11 | 109.3 | 148.3 | 145.2 | 63.6 | 66.7 | 66.9 | 0.489573 | 0.005484 | 2.260874 | -1.0304 | down |
| NUDT12 | 133.1 | 147.5 | 133.3 | 64.4 | 60 | 61.6 | 0.449384 | 0.000104 | 3.982487 | -1.15398 | down |
| HCLS1 | 122.2 | 132.4 | 146.9 | 62.8 | 68.9 | 66.8 | 0.494396 | 0.000788 | 3.103403 | -1.01626 | down |
| MAGED1 | 130.2 | 139.9 | 170.3 | 46.7 | 56.8 | 56.1 | 0.362398 | 0.001706 | 2.767916 | -1.46435 | down |
| PTGES | 133.7 | 163.6 | 134.8 | 56 | 55.7 | 56.2 | 0.388567 | 0.000845 | 3.072997 | -1.36376 | down |
| KLRG2 | 100.8 | 156.3 | 147.8 | 63.2 | 64.9 | 66.9 | 0.4816 | 0.015524 | 1.809004 | -1.05409 | down |
| FAT1 | 144.5 | 155.7 | 208.1 | 24.4 | 28.6 | 38.6 | 0.180209 | 0.002279 | 2.64231 | -2.47226 | down |
| MYEF2 | 148.3 | 141.6 | 140.3 | 54.2 | 59.5 | 55.9 | 0.394235 | 7.71E-06 | 5.112946 | -1.34287 | down |
| ZNF234 | 132.2 | 140.8 | 150.6 | 61.3 | 54.4 | 60.9 | 0.416903 | 0.00014 | 3.854524 | -1.26222 | down |
| ARHGAP9 | 105.1 | 157.1 | 167 | 65.8 | 50.2 | 54.7 | 0.397717 | 0.012033 | 1.919624 | -1.33019 | down |
| ZNF648 | 130.1 | 136.3 | 157.8 | 56.4 | 61.4 | 58 | 0.414427 | 0.000628 | 3.201959 | -1.27081 | down |
| TPGS2 | 110.8 | 153.3 | 144.3 | 76.5 | 51.5 | 63.7 | 0.469393 | 0.008173 | 2.087611 | -1.09113 | down |

| | | | | | | | | | | | |
|---|---|---|---|---|---|---|---|---|---|---|---|
| ADAM8 | 119.4 | 141.7 | 156.7 | 52.1 | 73.4 | 56.6 | 0.435854 | 0.003397 | 2.468967 | -1.19808 | down |
| CRYAB | 186.1 | 116.5 | 115.9 | 62.2 | 59.6 | 59.7 | 0.433692 | 0.027576 | 1.559475 | -1.20526 | down |
| RAVER2 | 157.2 | 165.7 | 190.8 | 32.6 | 29.6 | 24.2 | 0.168192 | 0.000163 | 3.78649 | -2.57182 | down |
| IRAG2 | 119.8 | 189.7 | 194.7 | 34.9 | 28.7 | 32.1 | 0.189806 | 0.004938 | 2.30642 | -2.39741 | down |
| LMO3 | 147.4 | 167.1 | 149.2 | 34.8 | 44 | 57.4 | 0.293724 | 0.000275 | 3.560112 | -1.76747 | down |

## Table S6. Differentially expression phosphorylated peptides of phosphoproteomics.

Differentially expression phosphorylated peptides of phosphoproteomics

| WT_rep.1 | WT_rep.2 | WT_rep.3 | HBB.KO_rep.1 | HBB.KO_rep.2 | HBB.KO_rep.3 | FC(HBB-KO vs WT) | Pvalue | Amino.acid | Localization.prob | Position | Proteins | Sequence.window | Phospho..STY..Probabilities | PhosphoSitePlus.window | log10Pvalue | log2FC | sig |
|---|---|---|---|---|---|---|---|---|---|---|---|---|---|---|---|---|---|
| 70387 | 38035 | 37082 | 112710 | 148510 | 139400 | 2.753326 | 0.0051774 | S | 0.96148 | 572 | P06396 | QTAPASTRLFQVRANSAGATRAVEVLPKAGA | ANS(0.961)AGAT(0.039)R | | 2.28616 | 1.461176 | up |
| 60522 | 47827 | 50330 | 170200 | 171310 | 186790 | 3.329363 | 4.89E-05 | S | 1 | 4 | P69905 | ____________MVLSPADKTNVKAAWGKVG | VLS(1)PADK | ____MVLsPADktNV | 4.310691 | 1.735246 | up |
| 20882 | 16354 | 14189 | 37317 | 54611 | 49189 | 2.744132 | 0.0054665 | S | 1 | 142 | P30043 | AVTDDHIRMHKVLRESGLKYVAVMPPHIGDQ | VLRES(1)GLK | MHkVLREsGLKyVAV | 2.262412 | 1.45635 | up |
| 14295 | 7932.6 | 9192.3 | 22991 | 20165 | 22227 | 2.080942 | 0.0059928 | S | 0.833597 | 204 | Q9UIG0 | SINDRARRSPRKLPTSLKKGERKWAPPKFLP | KLPT(0.166)S(0.834)LK | sPRKLPtsLKKGERK | 2.227061 | 1.057237 | up |
| 13924 | 4285 | 7263.2 | 16686 | 17823 | 17215 | 2.030606 | 0.0379999 | S | 0.992833 | 200 | Q15555 | GEQIFNLPKKSHHANSPTAGAAKSSPAAKPG | KSHHANS(0.993)PT(0.007)AGAAK | kkSHHANsPTAGAAk | 1.420232 | 1.02191 | up |
| 12995 | 9361.6 | 11863 | 30496 | 20397 | 26065 | 2.248945 | 0.0102177 | S | 0.964454 | 97 | P23193 | TEKDLDEKKKEPAITSQNSPEAREESTSSGN | KKEPAIT(0.035)S(0.964)QNSPEAR | KKEPAItsQNsPEAR | 1.99066 | 1.169248 | up |
| 12995 | 9361.6 | 11863 | 30496 | 20397 | 26065 | 2.248945 | 0.0102177 | S | 0.999999 | 100 | P23193 | DLDEKKKEPAITSQNSPEAREESTSSGNVSN | EPAIT(0.394)S(0.606)QNS(1)PEAR | PAItsQNsPEAREES | 1.99066 | 1.169248 | up |
| 11336 | 4029.1 | 2263.5 | 13351 | 13898 | 14368 | 2.360766 | 0.0457774 | S | 0.978309 | 307 | Q99442 | DLKKDEKSETKKQQKSDSEEKSDSEKKEDEE | S(0.978)DS(0.026)EEKS(0.171)DS(0.824)EK | ETKKQQKsDsEEKsD | 1.339378 | 1.239255 | up |
| 10805 | 8008.4 | 6184.4 | 20411 | 18111 | 18578 | 2.284201 | 0.0021244 | S | 0.784601 | 137 | Q86YP4 | TVALKETSTEALMKSSPEERERMIKQLKEEL | ETSTEALMKS(0.215)S(0.785)PEER | TEALMKssPEERERM | 2.672747 | 1.19169 | up |
| 10384 | 8813.6 | 9986.3 | 16457 | 24661 | 27440 | 2.349172 | 0.0169948 | S | 0.931994 | 41 | P10412;P16402;P16403 | AGATAGKRKASGPPVSELITKAVAASKERSG;KKAGGTPRKASGPPVSELITKAVAASKERSG;KSAGAAKRKASGPPVSELITKAVAASKERSG | KAS(0.046)GPPVS(0.932)ELIT(0.022)K | kAsGPPVsELItkAV | 1.770878 | 1.232152 | up |

| | | | | | | | | | | | | | | | | | |
|---|---|---|---|---|---|---|---|---|---|---|---|---|---|---|---|---|---|
| 10378 | 8129.1 | 9541.1 | 15756 | 30383 | 29252 | 2.687909 | 0.00292023 | S | 0.987873 | 530 | Q8IYB3 | GEVGRRRRHSPSRSASPSPRKRQKETSPRGR | S(0.013)AS(0.988)PS(0.999)PR | HsPsRsAsPsPRKRQ | 1.534569 | 1.426484 | up |
| 10249 | 7856.6 | 8967.9 | 20058 | 17887 | 17766 | 2.057769 | 0.0000715 | S | 0.916265 | 319 | P17252 | KFEKAKLGPAGNKVISPSEDRKQP-SNNLDRV | VIS(0.916)PS(0.084)EDR | PAGN-kVIsPsEDRkQ | 3.145622 | 1.041081 | up |
| 9481.6 | 1451.7 | 2530.7 | 14161 | 14802 | 14673 | 3.240939 | 0.0016332 | T | 0.987389 | 1885 | P21333 | AYGPGLTHGVVNKPATFTVNTKDAGEG-GLSL | PAT(0.987)FT(0.99)VNT(0.023)K | | 1.786962 | 1.696412 | up |
| 9481.6 | 1451.7 | 2530.7 | 14161 | 14802 | 14673 | 3.240939 | 0.0016332 | T | 0.989828 | 1887 | P21333 | GPGLTHGVVNKPATFTVNTKDAGEG-GLSLAI | PAT(0.987)FT(0.99)VNT(0.023)K | | 1.786962 | 1.696412 | up |
| 8847.2 | 4503 | 4832.6 | 13961 | 11054 | 12283 | 2.051279 | 0.0017438 | S | 0.975872 | 438 | Q9NZI8 | KKGQHIKQLSRFASASIKIAPPETPDSKVRM | FAS(0.024)AS(0.976)IK | LsRFASAs-IkIAPPE | 1.758499 | 1.036524 | up |
| 8844.2 | 5327.6 | 6921 | 19918 | 11188 | 14597 | 2.166758 | 0.0040012 | S | 0.964454 | 97 | P23193 | TEKDLDEKKKEPAITSQNSPEAREESTSSGN | KKEPAIT(0.035)S(0.964)QNSPEAR | KKEPAItsQNsPEAR | 1.397808 | 1.115538 | up |
| 8324.6 | 6867.8 | 7123.4 | 14797 | 16947 | 17219 | 2.194096 | 0.0000561 | S | 0.989384 | 293 | Q99623 | LVLNLQDESFTRGSDSLIKGKK_________ | GS(0.011)DS(0.989)LIK | sFTrGsD-sLIkGKK_ | 3.250759 | 1.133626 | up |
| 7561.2 | 5996.2 | 6044 | 11153 | 23201 | 19783 | 2.761895 | 0.0033574 | S | 1 | 33 | P63261;P60709;P63267;P68133;P68032;P62736;Q6S8J3;P0CG38;A5A3E0;Q9BYX7 | KAGFAGDDAPRAVFPSIVGRPRHQGVMVGMG | AVFPS(1)IVGRPR | APRAVFPsIVGRPRH;APRAVFPsIVGRPRQ | 1.474003 | 1.465658 | up |
| 6776.1 | 3937.7 | 5717.1 | 14513 | 10283 | 10548 | 2.151069 | 0.0016925 | S | 0.821544 | 1126 | O95239 | CCDPTKCRN-RQQGKDSLGTVERTQDSEGSFK | QQGKDS(0.822)LGT(0.178)VER | NRQQGKDsLGTVERt | 1.771465 | 1.105054 | up |
| 6628.5 | 2576.5 | 2515.8 | 10162 | 7906.5 | 8667.1 | 2.281039 | 0.0029747 | S | 1 | 427 | Q8TB61 | RGRLKQRGKKAVPVESPVQKV__________ | KAVPVES(1)PVQKV | KkAVPVEs-PVQkV__ | 1.526554 | 1.189691 | up |
| 6611.8 | 3016.7 | 4191.3 | 13015 | 8733.6 | 9383 | 2.252681 | 0.0027488 | S | 0.999997 | 347 | P50750 | LTSMFEYLAPPRRKGSQITQQSTNQSRNPAT | RKGS(1)QITQQST-NQSR | APPRR-kGsQItQQst | 1.560852 | 1.171643 | up |
| 6241.7 | 6323.1 | 6586 | 13957 | 14890 | 14938 | 2.286327 | 1.66E-05 | S | 0.993267 | 149 | Q9Y3C6 | QGIGMVNRVGMVETNS-QDRPVDDVKIIKAYP | VGMVET(0.007)NS(0.993)QDRPVDDVK | VGMVEtNs-QDRPVDD | 4.779892 | 1.193032 | up |

| | | | | | | | | | | | | | | | | | |
|---|---|---|---|---|---|---|---|---|---|---|---|---|---|---|---|---|---|
| 6175.1 | 4747.3 | 7145.5 | 10576 | 12725 | 13118 | 2.015674 | 0.004372 | S | 1 | 328 | Q9UKV3 | RVKPEEMMDERPKTRSQEQEVLER-GGRFTRS | S(1)QEQEVLER | DERPK-tRsQEQEVLE | 2.359272 | 1.0112262 | up |
| 5697.3 | 4855.4 | 6086.5 | 11696 | 11129 | 11008 | 2.033331 | 0.000168 | S | 0.995102 | 168 | O60238 | PKEFHFRHPKRSVSLSMRKSGAMKKGGIFSA | SVS(0.005)LS(0.995)MR | PKRsVsLs-MRKSGAM | 3.77452 | 1.023845 | up |
| 5661.8 | 5574.3 | 5266.7 | 22176 | 12214 | 12996 | 2.871392 | 0.03239 | S | 0.984823 | 63 | P50579 | PSAAGEQEPDKESGASVDE-VARQLERSALED | SKGPSAA-GEQEPDKES(0.015)GAS(0.985)VDEVAR | PDkEsGAsVDE-VARQ | 1.489592 | 1.52175 | up |
| 5638.9 | 2778.6 | 2368.5 | 7380.5 | 6646 | 7845.3 | 2.027795 | 0.027231 | S | 0.99478 | 594 | Q15424 | TSGSKERASKSQDRKSASREKRSVVSFDKVK | S(0.005)QDRKS(0.995)AS(1)R | | 1.564942 | 1.019912 | up |
| 5638.9 | 2778.6 | 2368.5 | 7380.5 | 6646 | 7845.3 | 2.027795 | 0.027231 | S | 0.999868 | 596 | Q15424 | GSKERASKSQDRKSASREKRSVVSFDKVKEP | S(0.005)QDRKS(0.995)AS(1)R | | 1.564942 | 1.019912 | up |
| 5139.1 | 3557.1 | 4297 | 6865.5 | 15945 | 13648 | 2.805968 | 0.047332 | T | 1 | 199 | P08621 | VKGWRPRRLGGGLGGTRRGGADVNIR-HSGRD | LGGGLGGT(1)RR | LGGGLGGtRrG-GADV | 1.324848 | 1.488498 | up |
| 5069.1 | 2691.6 | 4008.5 | 8893.7 | 9790.3 | 10118 | 2.447235 | 0.001883 | S | 0.932591 | 287 | Q08495 | DRTPFHTSLHQGTSKSSSLPAYGRTTLSRLQ | S(0.933)S(0.012)S(0.056)LPAYGR | LHQGTsKsssLPAyG | 2.725089 | 1.291153 | up |
| 4978.9 | 4019.2 | 3433.3 | 6907 | 15340 | 16098 | 3.084528 | 0.044168 | S | 1 | 233 | Q16629 | PKRSRSPSGSPRRSASPERMD__________ | S(1)AS(1)PERMD | GsPRRsAsPErMD__ | 1.354897 | 1.62505 | up |
| 4574.1 | 3690.9 | 4858.6 | 9055.7 | 10421 | 10179 | 2.259723 | 0.000551 | S | 0.892882 | 484 | P47736 | DLAKAAGISLIVPGKSPTRKKSGPFGSRRSS | AA-GIS(0.004)LIVPGKS(0.893)PT(0.103)R | SLIVPGKsPtRK-KsG | 3.25912 | 1.176146 | up |
| 4435 | 3916.1 | 4401.2 | 7563.4 | 8376.3 | 10175 | 2.047842 | 0.004862 | S | 1 | 438 | Q8NI36 | INKKRVKRKGLQNTMSVRLPPITKFAAEEAR | GLQNTMS(1)VR | kGLQNTMsVRL-PPIT | 2.313201 | 1.034105 | up |
| 4253.7 | 4059.8 | 3258.1 | 7354.7 | 8741.6 | 7935.7 | 2.076809 | 0.001187 | S | 1 | 16 | P46776 | MPSRLRKTRKLRGHVSH-GHGRIGKHRKHPGG | GHVS(1)HGHGR | RKLrGHVsH-GHGRIG | 2.925474 | 1.054368 | up |
| 4253.1 | 3262.2 | 2270.5 | 6605 | 6663.8 | 7411 | 2.113246 | 0.004453 | S | 0.809984 | 92 | Q99879;Q99877;Q93079;Q5QNW6;P62807;P58876;P57053;O60814;Q16778;P33778;P23527;P06899;Q6DRA6;Q6DN03;Q99880;Q8N257 | EASRLAHYNKRSTITSREIQTAVRLLLPGEL | S(0.001)T(0.001)IT(0.188)S(0.81)REIQTAVR | NkrstIt-srEIQtAV;NKrstITsrEIQtAV;NkrstITsrEIQtAV | 2.351309 | 1.079461 | up |
| 4087.6 | 2589.7 | 2782.7 | 7578.5 | 12202 | 13911 | 3.561469 | 0.014 | S | 0.999978 | 140 | P16104 | VGPKAPSGGKKATQASQEY____________ | ATQAS(1)QEY | GkkAtQAsQEy___ | 1.843776 | 1.832 | up |

| | | | | | | | | | | | | | | | | | |
|---|---|---|---|---|---|---|---|---|---|---|---|---|---|---|---|---|---|
| | | | | | | | 329 | | | | | | | | | 473 | |
| 3727.2 | 4476 | 4355.2 | 9962.7 | 10409 | 10245 | 2.437946 | 2.27E-05 | S | 0.797612 | 3128 | P46013 | ERIEINRNEKKPMKTSPEMDIQNPDDGARKP | KPMKT(0.202)S(0.798)PEMDIQNPDD-GAR | EKKPMKtsPEMDIQN | 4.643974 | 1.285666 | up |
| 3704.7 | 2631.8 | 3154.9 | 5432.5 | 7148.8 | 8562.7 | 2.227701 | 0.0015341 | S | 0.782336 | 31 | Q9BW19 | ELKRPLIKAPSQLPLSGSRLKKRRPDQMEDGL | AP-SQLPLS(0.782)GS(0.218)R | APsQLPLsGsRL-KRR | 1.814146 | 1.155556 | up |
| 3585.5 | 2440.6 | 2983.5 | 7152.2 | 8954.1 | 8868.1 | 2.771976 | 0.0001388 | S | 0.942428 | 2084 | Q9UQ35 | TRSPPAIRRRSASGSSSDRSRSATPPATRNH | SASGS(0.019)S(0.942)S(0.039)DR | | 2.857509 | 1.470915 | up |
| 3437.8 | 4014.4 | 4240.7 | 9274.2 | 8383.3 | 9548.9 | 2.326745 | 0.0000263 | T | 0.972541 | 121 | Q99878;Q96KK5;Q93077;Q7L7L0;Q6FI13;P20671;P0C0S8;P04908 | GGVLPNIQAVLLPKKTESHHKTK________ | KT(0.973)ES(0.027)HHK | AVLL-PkktEsHHkTk | 3.580684 | 1.218313 | up |
| 3371.5 | 1386.6 | 1852.9 | 4606.7 | 4196.5 | 4633.3 | 2.032446 | 0.002092 | S | 0.95085 | 262 | Q05519 | RHSRSRSRSRRRRTPSSSRHRRSRSRSRRRS | RT(0.024)PS(0.951)S(0.022)S(0.004)R | SRRRRtPsSsRHRRS | 1.679431 | 1.023217 | up |
| 3320.8 | 2617 | 3127.1 | 6658.3 | 12147 | 10974 | 3.285122 | 0.0014789 | S | 0.882274 | 307 | P35659 | KNQNSSKKESESEDSSDDEPLIKKLKKPPTD | ESESEDS(0.118)S(0.882)DDEPLIKK | EsEsEDssDDEP-LIK | 1.830059 | 1.715947 | up |
| 3209.9 | 2611.8 | 2709.4 | 5883.6 | 5690.4 | 5969.5 | 2.056417 | 0.0000121 | S | 1 | 919 | Q15042 | AAAMTPPEEELKRMGSPEERRQNSVSDFPPP | MGS(1)PEER | EELkRMG-sPEERRQN | 3.917477 | 1.040133 | up |
| 2933.4 | 2489.6 | 2375 | 5411.7 | 7264.2 | 7946.2 | 2.644537 | 0.0005301 | S | 0.999726 | 918 | Q9H3U1 | SEMMEILSVLA-KGDHSPVTRAAAACLDKAVE | GDHS(1)PVTR | | 2.275645 | 1.403015 | up |
| 2896 | 200.79 | 284.17 | 7121.1 | 9050.2 | 10470 | 7.879803 | 0.0004119 | S | 0.955784 | 58 | P62995 | SRRSRSKSRSRSESRSRSRRSSRRHYTRSRS | S(0.383)RS(0.596)ES(0.066)RS(0.956)R | | 2.3852 | 2.97816 | up |
| 2633.3 | 549.63 | 659.56 | 3177.7 | 6394.8 | 6243.2 | 4.116003 | 0.0032936 | S | 0.99287 | 707 | Q8IYB3 | QTSSSPPPVRRGASSSPQRRQSPSPSTRPIR | GAS(0.642)S(0.365)S(0.993)PQR | VRR-GAsssPQRRQsP | 1.482324 | 2.041244 | up |
| 2442.6 | 2153.7 | 2638 | 7712.7 | 7496.3 | 7657.8 | 3.160886 | 4.66E-06 | T | 1 | 55 | Q12834 | PSPMRAANRSHSAGRTPGRTPGKSSSKVQTT | T(1)PGRT(1)PGK | RsH-sAGRtPGRtPGk | 5.331614 | 1.660329 | up |
| 2442.6 | 2153.7 | 2638 | 7712.7 | 7496.3 | 7657.8 | 3.160886 | 4.66E-06 | T | 1 | 59 | Q12834 | RAANRSHSAGRTPGRTPGKSSSKVQTTPSKP | T(1)PGRT(1)PGK | AGRtPGRtPGkSSSk | 5.331614 | 1.660329 | up |

| | | | | | | | | | | | | | | | | | |
|---|---|---|---|---|---|---|---|---|---|---|---|---|---|---|---|---|---|
| 2235 | 1613.9 | 1876.1 | 3532 | 4536.2 | 4544.1 | 2.203022 | 0.003831 | S | 0.999949 | 86 | P49792;Q7Z3J3;Q99666;O14715 | ELEENTDKAVECYRRSVELNPTQKDLVLKIA;ELEENTEKAVECYRRSVELNPTQKDLVLKIA | S(1)VELNPTQK | AVECYRRsVELNPtQ | 2.416682 | 1.139484 | up |
| 2176.7 | 2433.4 | 2744.8 | 5471.2 | 4708.2 | 5905.5 | 2.186964 | 0.001668 | S | 0.99997 | 222 | Q00653 | LPLKPVISQPIHDSKSPGASNLKISRMDKTA | S(1)PGASNLK | QPIHDSKsPGASNLk | 2.777931 | 1.128929 | up |
| 2089.4 | 2100.2 | 2285.7 | 4280.6 | 5143.5 | 5777.4 | 2.347613 | 0.002678 | S | 0.998527 | 647 | Q8N1F7 | AKNADKVLELMNKLLSPVVPQISAP-QSNKER | LLS(0.999)PVVPQIS(0.001)APQSNK | ELMNkLL-sPVVPQIs | 2.572156 | 1.231195 | up |
| 1930.7 | 1625.9 | 1855.7 | 3508.7 | 4815.4 | 4293.9 | 2.331356 | 0.00355 | S | 0.961401 | 2 | P16401 | ______________MSETAPAETATPAPVEK | S(0.961)ET(0.964)APAET(0.05)AT(0.021)PAPVEKS(0.003)PAK | ______MsEtAPAEt | 2.449764 | 1.22117 | up |
| 1930.7 | 1625.9 | 1855.7 | 3508.7 | 4815.4 | 4293.9 | 2.331356 | 0.00355 | T | 0.964308 | 4 | P16401 | ____________MSETAPAETATPAPVEKSP | S(0.036)ET(0.964)APAETATPAPVEK | ____MsEtAPAE-tAt | 2.449764 | 1.22117 | up |
| 1543.8 | 1497.7 | 1536.4 | 4282.9 | 4289.9 | 4467.8 | 2.848599 | 1.41E-06 | T | 0.986148 | 478 | P11166 | IASGFRQGGASQSDKTPEELFHPLGADSQV_ | QGGASQS(0.008)DKT(0.986)PEELF-HPL-GADS(0.005)QV | GAsQSDktPEELFHP | 5.850781 | 1.510252 | up |
| 1435.1 | 787.55 | 1143.6 | 2000.7 | 4176.9 | 4100.5 | 3.053279 | 0.0353344 | S | 0.999994 | 154 | O43395;Q9Y2W1 | EEGRSSRHSKSSSDRS-RKRELKEVFGDDSEI;SRSHSRNSDKSSSDRS-RRSSSSRSSSNHSRV | SSSDRS(1)R | DKsssDRs-RRSsSSR | 1.451687 | 1.610359 | up |
| 1019.3 | 583.11 | 902.17 | 1629.5 | 2515.2 | 2209.4 | 2.536992 | 0.01155 | S | 0.993556 | 1097 | Q6PD62 | DQDSDSDQPSRKRRPSGSEQSDNESVQSGRS | RRPS(0.994)GS(0.006)EQSDNESVQSGR | PsRKRRPsGs-EQsDN | 1.937422 | 1.343119 | up |
| 899.15 | 425.66 | 493.79 | 1497.8 | 1226.1 | 1586.6 | 2.37023 | 0.010566 | S | 1 | 751 | Q96D71 | SIPRSVGKDKKAIQASIRRNKETNTVLARLN | AIQAS(1)IRR | DKKAIQAsIRRNKEt | 1.97609 | 1.245027 | up |
| 898.61 | 618.81 | 566.96 | 1785.8 | 2063 | 2210.6 | 2.907052 | 0.001205 | S | 0.999997 | 2672 | Q6KC79 | GSPKNNTAAETEDDESDGEDRGGGTSGSLRR | NNTAAETED-DES(1)DGEDR | AEtEDDEsDGEDRGG | 2.918863 | 1.539557 | up |
| 644.99 | 359.37 | 811.56 | 1458.8 | 1279.9 | 1084.9 | 2.105599 | 0.017194 | S | 0.974756 | 420 | O60296 | FDTVRIANDTRGRSISFPALLPIPGSNRSSV | S(0.025)IS(0.975)FPALLPIPGSNR | DTRGR-SIsFPALLPI | 1.764626 | 1.074231 | up |
| 265.07 | 393.28 | 367.03 | 591.32 | 696.82 | 790.06 | 2.026761 | 0.007219 | S | 0.992034 | 759 | P16157 | QGHTDIVTLLLKNGASPNEVSSDGTTPLAIA | NGAS(0.992)PNEVS(0.002)S(0.002)DGT(0.002)T(0.002)PLAIAK | LLLKNGAsP-NEVSSD | 2.141531 | 1.019176 | up |
| 16579 | 24548 | 26377 | 7708.1 | 8712.3 | 9060.7 | 0.377475 | 0.009 | S | 0.999801 | 84 | Q9Y6M7 | RRRKDKESDKEDGRESPSYDTPSQRVQFILG | ESDKED-GRES(1)PSYDTP-SQR | DKED-GREsPsyDtPs | 2.003645 | -1.4 | do |

| | | | | | | | | | | | | | | | | | |
|---|---|---|---|---|---|---|---|---|---|---|---|---|---|---|---|---|---|
| | | | | | | | 916 | | | | | | | | | 0555 | wn |
| 14559 | 12625 | 12127 | 5508.7 | 4539.4 | 4289.4 | 0.36472 | 0.000555 | S | 1 | 83 | P10644 | LQKAGTRTDSREDEISPPPPNPVVKGRRRRG | EDEIS(1)PPPPNPVVK | DsREDE-IsPPPPNPV | 3.255792 | -1.45514 | down |
| 3311.3 | 3379.9 | 3965.5 | 1717.1 | 1518.2 | 1812.5 | 0.473674 | 0.001145 | S | 1 | 286 | O95072 | MEVTPPEELRLPAPPSPERRPPVPPPPRRRR | LPAPPS(1)PER | LRLPAPPsPER-RPPV | 2.941286 | -1.07803 | down |
| 2718.9 | 4741.9 | 5159.1 | 1868.2 | 2013.2 | 2139.3 | 0.47708 | 0.043963 | S | 0.941516 | 556 | Q9Y6M7 | VPSQEKRKIPVFHNGSTPTLGETPKEAAHHA | IPVFHNGS(0.942)T(0.039)PT(0.02)LGETPK | IPVFHNGstPt-LGEt | 1.356911 | -1.0677 | down |

Table S7. RT-qPCR primers

| | RT-qPCR primers: | |
|---|---|---|
| Name | forward(5' - 3') | reverse(5' - 3') |
| HBA(??-globin) | AACTTCAAGCTCCTAAGCCA | CAGGAACTTGTCCAGGGAG |
| HBG1/2(??-globin) | GAACTTCAAGCTCCTGGGA | AGTCACCATCTTCTGCCAG |
| HBB(??-globin) | GAGAACTTCAGGCTCCTGG | ACTTTCTGATAGGCAGCCTG |
| GAPDH | TCATTTCCTGGTATGACAACGA | GTCTTACTCCTTGGAGGCC |
| BCL11A | GGGTATTTGTAAAGATGAGCCC | TGCAAGAGAAACCATGCAC |
| ZBTB7A | GTGCAACATCTGCAAGGTC | TTCAGGTCGTAGTTGTGGG |
| Band3 | ACCTCTCTCACCTCACCTTCTG | AACCTGTCTAGCAGTTGGTTGG |
| KLF1 | GGTTGCGGCAAGAGCTACA | GTCAGAGCGCGAAAAAGCAC |
| KLF3 | TGTCTCAGTGTCATACCCATCT | CCTTCTGGGGTCTGAAAGAACTT |
| FOG1 | CGTGCTTCGAGTGCGAGAT | GGCCTGAACAGTAGAGGCG |
| AHSP | GGATCTCATTTCCGCAGGATTG | CTGCTGCCTGTAATAGTTGATGT |
| NFE2 | CCACCACCCACAACTTACTG | GAGGGCTAAGGGGTCTTGGA |
| EPOR | TGGAGGACTTGGTGTGTTTCT | GCAACTCTAGGGGCACGAA |
| GFI1B | GCAGGAAGATGAACCGCTCT | CCAGGCACTGGTTTGGGAA |
